# A starting guide to root ecology: strengthening ecological concepts and standardising root classification, sampling, processing and trait measurements

**DOI:** 10.1111/nph.17572

**Published:** 2021-10-05

**Authors:** Grégoire T. Freschet, Loïc Pagès, Colleen M. Iversen, Louise H. Comas, Boris Rewald, Catherine Roumet, Jitka Klimešová, Marcin Zadworny, Hendrik Poorter, Johannes A. Postma, Thomas S. Adams, Agnieszka Bagniewska‐Zadworna, A. Glyn Bengough, Elison B. Blancaflor, Ivano Brunner, Johannes H. C. Cornelissen, Eric Garnier, Arthur Gessler, Sarah E. Hobbie, Ina C. Meier, Liesje Mommer, Catherine Picon‐Cochard, Laura Rose, Peter Ryser, Michael Scherer‐Lorenzen, Nadejda A. Soudzilovskaia, Alexia Stokes, Tao Sun, Oscar J. Valverde‐Barrantes, Monique Weemstra, Alexandra Weigelt, Nina Wurzburger, Larry M. York, Sarah A. Batterman, Moemy Gomes de Moraes, Štěpán Janeček, Hans Lambers, Verity Salmon, Nishanth Tharayil, M. Luke McCormack

**Affiliations:** ^1^ CEFE Univ Montpellier, CNRS, EPHE, IRD 1919 route de Mende Montpellier 34293 France; ^2^ Station d’Ecologie Théorique et Expérimentale CNRS 2 route du CNRS 09200 Moulis France; ^3^ UR 1115 PSH Centre PACA, site Agroparc INRAE 84914 Avignon cedex 9 France; ^4^ Environmental Sciences Division and Climate Change Science Institute Oak Ridge National Laboratory Oak Ridge TN 37831 USA; ^5^ USDA‐ARS Water Management Research Unit 2150 Centre Avenue, Bldg D, Suite 320 Fort Collins CO 80526 USA; ^6^ Department of Forest and Soil Sciences University of Natural Resources and Life Sciences Vienna 1190 Austria; ^7^ Department of Functional Ecology Institute of Botany CAS Dukelska 135 37901 Trebon Czech Republic; ^8^ Institute of Dendrology Polish Academy of Sciences Parkowa 5 62‐035 Kórnik Poland; ^9^ Plant Sciences (IBG‐2) Forschungszentrum Jülich GmbH D‐52425 Jülich Germany; ^10^ Department of Biological Sciences Macquarie University North Ryde NSW 2109 Australia; ^11^ Department of Plant Sciences The Pennsylvania State University University Park PA 16802 USA; ^12^ Department of General Botany Institute of Experimental Biology Faculty of Biology Adam Mickiewicz University Uniwersytetu Poznańskiego 6 61-614 Poznań Poland; ^13^ The James Hutton Institute Invergowrie, Dundee, DD2 5DA UK; ^14^ School of Science and Engineering University of Dundee Dundee, DD1 4HN UK; ^15^ Noble Research Institute, LLC 2510 Sam Noble Parkway Ardmore OK 73401 USA; ^16^ Forest Soils and Biogeochemistry Swiss Federal Research Institute WSL Zürcherstr. 111 8903 Birmensdorf Switzerland; ^17^ Department of Ecological Science Faculty of Science Vrije Universiteit Amsterdam De Boelelaan 1085 Amsterdam 1081 HV the Netherlands; ^18^ Forest Dynamics Swiss Federal Research Institute WSL Zürcherstr. 111 8903 Birmensdorf Switzerland; ^19^ Institute of Terrestrial Ecosystems ETH Zurich 8092 Zurich Switzerland; ^20^ Department of Ecology, Evolution and Behavior University of Minnesota St Paul MN 55108 USA; ^21^ Functional Forest Ecology University of Hamburg Haidkrugsweg 1 22885 Barsbütel Germany; ^22^ Plant Ecology and Nature Conservation Group Department of Environmental Sciences Wageningen University and Research PO Box 47 6700 AA Wageningen the Netherlands; ^23^ Université Clermont Auvergne INRAE VetAgro Sup UREP 63000 Clermont-Ferrand France; ^24^ Senckenberg Biodiversity and Climate Research Centre (BiK-F) Senckenberganlage 25 60325 Frankfurt am Main Germany; ^25^ Laurentian University 935 Ramsey Lake Road Sudbury ON P3E 2C6 Canada; ^26^ Geobotany Faculty of Biology University of Freiburg Schänzlestr. 1 79104 Freiburg Germany; ^27^ Environmental Biology Department Institute of Environmental Sciences CML Leiden University Leiden 2300 RA the Netherlands; ^28^ INRAE AMAP CIRAD, IRD CNRS University of Montpellier Montpellier 34000 France; ^29^ Institute of Applied Ecology Chinese Academy of Sciences Shenyang 110016 China; ^30^ International Center for Tropical Botany Department of Biological Sciences Florida International University Miami FL 33199 USA; ^31^ Systematic Botany and Functional Biodiversity Institute of Biology Leipzig University Johannisallee 21-23 Leipzig 04103 Germany; ^32^ Odum School of Ecology University of Georgia 140 E. Green Street Athens GA 30602 USA; ^33^ Biosciences Division and Center for Bioenergy Innovation Oak Ridge National Laboratory Oak Ridge TN 37831 USA; ^34^ School of Geography and Priestley International Centre for Climate University of Leeds Leeds LS2 9JT UK; ^35^ Cary Institute of Ecosystem Studies Millbrook NY 12545 USA; ^36^ Department of Botany Institute of Biological Sciences Federal University of Goiás 19 74690-900 Goiânia, Goiás Brazil; ^37^ School of Biological Sciences The University of Western Australia 35 Stirling Highway Crawley (Perth) WA 6009 Australia; ^38^ School of Biological Sciences The University of Western Australia Crawley (Perth) WA Australia; ^39^ Environmental Sciences Division and Climate Change Science Institute Oak Ridge National Laboratory Oak Ridge TN 37831 USA; ^40^ Department of Plant and Environmental Sciences Clemson University Clemson SC 29634 USA; ^41^ Center for Tree Science Morton Arboretum, 4100 Illinois Rt. 53 Lisle IL 60532 USA

**Keywords:** below‐ground ecology, handbook, plant root functions, protocol, root classification, root ecology, root traits, trait measurements

## Abstract

In the context of a recent massive increase in research on plant root functions and their impact on the environment, root ecologists currently face many important challenges to keep on generating cutting‐edge, meaningful and integrated knowledge. Consideration of the below‐ground components in plant and ecosystem studies has been consistently called for in recent decades, but methodology is disparate and sometimes inappropriate. This handbook, based on the collective effort of a large team of experts, will improve trait comparisons across studies and integration of information across databases by providing standardised methods and controlled vocabularies. It is meant to be used not only as starting point by students and scientists who desire working on below‐ground ecosystems, but also by experts for consolidating and broadening their views on multiple aspects of root ecology. Beyond the classical compilation of measurement protocols, we have synthesised recommendations from the literature to provide key background knowledge useful for: (1) defining below‐ground plant entities and giving keys for their meaningful dissection, classification and naming beyond the classical fine‐root vs coarse‐root approach; (2) considering the specificity of root research to produce sound laboratory and field data; (3) describing typical, but overlooked steps for studying roots (e.g. root handling, cleaning and storage); and (4) gathering metadata necessary for the interpretation of results and their reuse. Most importantly, all root traits have been introduced with some degree of ecological context that will be a foundation for understanding their ecological meaning, their typical use and uncertainties, and some methodological and conceptual perspectives for future research. Considering all of this, we urge readers not to solely extract protocol recommendations for trait measurements from this work, but to take a moment to read and reflect on the extensive information contained in this broader guide to root ecology, including sections I–VII and the many introductions to each section and root trait description. Finally, it is critical to understand that a major aim of this guide is to help break down barriers between the many subdisciplines of root ecology and ecophysiology, broaden researchers’ views on the multiple aspects of root study and create favourable conditions for the inception of comprehensive experiments on the role of roots in plant and ecosystem functioning.

**Table:** 

**Contents**		
	Summary	974
I.	Introduction: continuing to face up to root ecology's challenges	975
II.	Semantics: defining concepts for better understanding and communication	977
III.	Species‐level vs ecosystem‐level measurements	978
IV.	Below‐ground plant entities and root classifications	979
V.	Contextualisation and reuse of data	988
VI.	Experimentation and sampling in laboratory and field	989
VII.	Root washing, sorting and storage	1001
VIII.	Horizontal plant mobility	1004
IX.	Below‐ground allocation	1007
X.	Root system architecture	1013
XI.	Root spatial distribution	1017
XII.	Root morphology	1021
XIII.	Root anatomy	1028
XIV.	Root chemistry	1037
XV.	Root mechanics	1046
XVI.	Root dynamics	1050
XVII.	Root respiration and exudation	1056
XVIII.	Physiology of resource uptake	1063
XIX.	Mycorrhizal associations	1070
XX.	Nitrogen‐fixing symbioses	1075
XXI.	Root tip morphology and elongation	1080
XXII.	Root hair morphology and development	1084
XXIII.	Root decomposition	1090
	Acknowledgements	1094
	References	1095

## 1 Introduction: continuing to face up to root ecology's challenges

### Root ecology is currently facing a number of challenges

Below‐ground parts of plants play key roles in plant functioning and performance and affect many ecosystem processes and functions (Gregory, 2006; Bardgett *et al*., 2014; Freschet *et al*., 2021). The fields of root functional ecology and ecophysiology have recently attracted much interest and the number of studies integrating aspects of below‐ground parts of plants is rapidly rising. Such rapid developments have benefited from the critical perspectives opened by multidimensional characterisations of plant strategies (*sensu* Grime *et al*., 1997) and the popularisation of a few standardised, easily measurable root morphological and chemical traits (Cornelissen *et al*., 2003). However, in the context of an exponentially increasing interest for root functions, root ecology currently faces many important challenges.

A first challenge lies in the difficulty to define a common, unambiguous language to accurately communicate among disciplines of root science and with the broader fields of ecology, agronomy, horticulture, forestry, etc. (Garnier *et al*., 2017; see under section **II. Semantics**). In the same way that grammar structures our language, semantics can help distinguish between the major elements that define a trait measurement – quality, entity and protocol – to precisely define the terms commonly used and help us evaluate the homogeneity of measurements made across numerous studies. In this context, it is also critical to elucidate the ecological foundation of typical classification practices of below‐ground plant entities (see under section **IV. Below‐ground plant entities and root classifications**). Indeed, root systems are continuums of root segments that vary in anatomy, morphology, physiology, mechanical properties, etc. (Pregitzer *et al*., 2002; Wells & Eissenstat, 2002). Such segments or group of segments vary in their contribution to different plant and ecosystem functions (McCormack *et al*., 2015a).

A second challenge rests in the better accounting of spatial and temporal variability of root traits (Shipley *et al*., 2016). Below‐ground parts of plants vary in anatomy, morphology and physiology throughout the life of a root, and the time chosen for root sampling influences the value and meaning of trait measurements. Similarly, they vary depending on the plant environment (and particularly soil properties) across all spatial scales and even at the level of a single plant. Acknowledging this variation implies both establishing guidelines for a minimal characterisation and contextualisation of plant measurements (see section **VI. Experimentation and sampling in laboratory and field**) and improving our understanding of general patterns of below‐ground trait variation across environmental gradients and temporal cycles (Freschet *et al*., 2021).

A third challenge is linked to the high number of technical and practical choices associated with both laboratory and field studies (see section **VI. Experimentation and sampling in laboratory and field**) that have important consequences for the value and interpretation made of trait measurements (e.g. Poorter *et al*., 2012a, 2016). Designing sound experiments requires for instance anticipating the methodological bias induced by inadequate experimental features or sampling location, finding the right balance between exhaustively sampling and cleaning of roots and minimising root damages and labour, or even using appropriate methods for storing roots over short to long periods of time (see section **VII. Root washing, sorting and storage**). Much of this knowledge must be adapted to specific environmental and experimental contexts (e.g. following climate, soil type, research questions) and many gaps remain in our capacities to anticipate issues related to root sampling and measurements. However, generalist knowledge exists and keys of reflection can be further proposed to guide researchers into sound practices.

A fourth challenge lies in improving the soundness and reproducibility of trait measurement methods (Iversen *et al*., 2017). Several commentaries and methodological assessments have for instance highlighted concerns about common methodological biases and pitfalls in specific trait measurements (e.g. Birouste *et al*., 2014; Delory *et al*., 2017; Rose, 2017), without reaching enough visibility or consensus. Also, a better accounting of root associations with symbiotic organisms and the consequences for root trait measurement and root functions is critically needed. Providing a core set of well established methods, and raising awareness of measurement bias and inaccuracies, is thus critical to improve measurement quality, consistency and interpretation (see sections **VIII**–**XXIII**).

A fifth challenge is to strengthen the general understanding of root trait ecological meaning, and emphasise current limitations and promises in the use of these traits as proxies for plant and ecosystem functioning (Laliberté, 2016; Freschet *et al*., 2021). Root ecology is not a young science, but the knowledge gaps regarding linkages between root traits and functions remain to date numerous and hinder adequate quantification of plant and ecosystem functions. Assembling basic common knowledge about the ecological value of traits and highlighting potential future research directions should help to strengthen the foundation of below‐ground functional ecology (see sections **VIII**–**XXIII**).

**Fig. 1:**
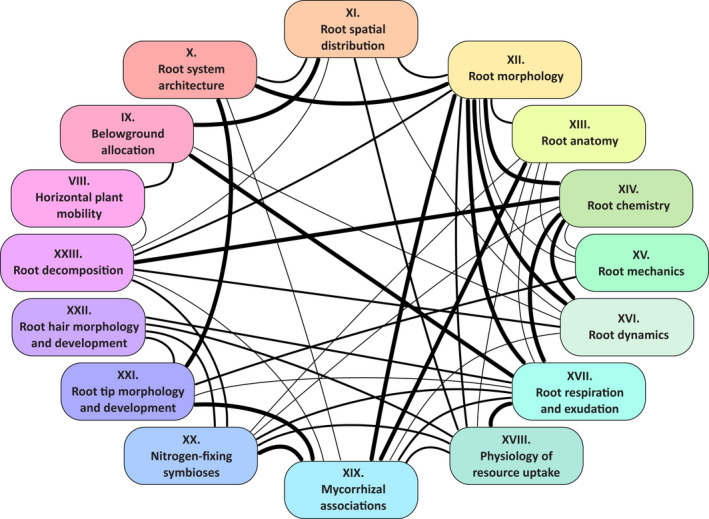
Map of trait categories included in this guide and approximate frequency at which these categories have been studied together. While not all categories are necessarily relevant to study together, this diagram can be used to identify the (lack of) connections between these ‘fields’ of research. The width of connectors depicts weak‐to‐strong linkages between categories. No connector, no or very few studies looking at both fields jointly; thin connectors, few studies; medium connectors, fields sharing substantial number of studies; thick connectors, fields that are frequently studied together. This diagram represents the authors’ expert assessment only and is imperfect as no exhaustive review of the literature was carried out.

A sixth challenge is to shed light on a number of trait categories and traits usually not known or considered by nonspecialist root researchers and help create bridges among different disciplines of root ecology. Coupling measurements from several fields of root ecology is often needed to adequately capture specific plant and ecosystem functions (e.g. McCormack *et al*., 2017; Freschet *et al*., 2018), sometimes across different below‐ground plant entities (Freschet & Roumet, 2017). Therefore, only multidisciplinary root ecology science will be able to capture adequately the integrative response of plants to environmental variations and the effects of roots on ecosystem processes (Fig. 1; see Sections **VIII**–**XXIII**).

As mentioned earlier, root systems can be defined as continuums of root segments that vary in anatomy, morphology, architecture and physiology, both spatially (e.g. different parts of the same root system and different position in soil) and temporally (e.g. plastic changes, root ageing) (Pregitzer *et al*., 2002; Wells & Eissenstat, 2002). As a result, they perform multiple functions varying across space and time. In this context, adequately characterising root functions requires *a priori* knowledge of when (e.g. along seasonal cycles, daily cycles), where (e.g. across soil layers) and what below‐ground entities (e.g. root orders) to sample, and what type of trait (e.g. morphological, architectural) to measure (Freschet *et al*., 2021). Setting such measurements in a well characterised context and allowing their comparability and further use in global assessments require additional care. In this context, this handbook represents a collective effort to assemble, sort through and summarise a core of current knowledge on root functional ecology, to navigate ourselves and future root ecologists into this complexity. We hope that this major undertaking will be instrumental in strengthening the foundations of root ecology.

### Conceptual organisation of the handbook

The conception of this handbook was largely inspired by previous handbooks of plant traits, particularly Cornelissen *et al*. (2003) and its later update by Pérez‐Harguindeguy *et al*. (2013) and follows the same principles. In line with these manuals, we considered plant traits as ‘any morphological, physiological or phenological feature, measurable for individual plants, at the cell to the whole organism level, which potentially affects its fitness (cf. McGill *et al*., 2006; Lavorel *et al*., 2007; Violle *et al*., 2007) or its environment (Lavorel & Garnier, 2002)’ and ‘call the particular value or modality taken by the trait at any place and time an ‘attribute’ (Lavorel *et al*., 2007)’.’ To clarify further the choice of terms made in this handbook, a section was specifically dedicated to trait semantics (see section **II. Semantics**). Additionally, we considered in some cases characteristics of plant communities scaled up from plant traits or measured at the community or ecosystem level. These are referred to as ‘community functional parameters’ (CFP; Violle *et al*., 2007) or ‘community traits’ when considering the mean value of the community characteristics, as discussed under section **III. Species‐level vs ecosystem‐level measurements**. The present focus on below‐ground plant traits implies that selected traits for this handbook relate to a range of plant organs located under the soil surface, including structures such as roots (whether coarse or fine), root hairs, rhizomes, bulbs or tubers, as defined under section **IV. Below‐ground plant entities and root classifications**. Importantly, section **IV. Below‐ground plant entities and root classifications** also elucidates the most common root classification systems and discusses the advantages and disadvantages of their use in root research. To raise awareness of the growing need of metadata in analyses of global trait patterns and dynamic biogeochemical modelling, a section describes key aspects of data gathering and management (see section **V. Contextualisation and reuse of data** **)**. Most importantly, a core description of general sound practices in section **VI. Experimentation and sampling in laboratory and field** considers the specificity of root studies. It is followed by a discussion of common knowledge on section **VII. Root washing, sorting and storage**, to improve the soundness and reproducibility of root studies.

These six sections are followed by the core of the handbook, where traits are organised into 16 broad categories reflecting different disciplines of root ecology (Fig. 1; see sections **VIII**–**XXIII**). The selection of traits was based on similar principles as previous plant trait handbooks (Cornelissen *et al*., 2003; Pérez‐Harguindeguy *et al*., 2013), although with a particular focus on two requirements: demonstrating a substantial link between the trait and plant or ecosystem functioning and covering a set of traits responsible for a range of functions. The final list of 76 traits represents a nonexhaustive, subjective selection of traits that the authors considered valuable to highlight. Each trait section includes an unequivocal definition of the trait, a general description of the trait ecological value, specific recommendations for root experimentation and sampling, as well as root storage and processing, and a step‐by‐step description of the proposed measurement methodology. When relevant, perspectives for better trait contextualisation and methodological improvements were discussed.

## 2 Semantics: defining concepts for better understanding and communication

Every single day of our working life, we are using and dealing with a range of concepts that are insufficiently defined. Try to ask the meaning of a widely used concept (say ‘root nutrient uptake’) to a naïve but interested audience. Each person will bear a personal view on the topic, and you will soon be confronted with questions that either shake the basis of your definition or call for additional boundaries or extensions of the concept. Very soon, someone might also point out that the ‘name’ of your concept, or the terms that you are using to define it, is causing confusion to him/her. We all use terms that represent concepts imperfectly, and we all use terms that do not correspond to the understanding of other colleagues, be they at the other side of the planet or next door. Such semantic heterogeneity, defined here as differences in the meanings of terms and concepts, is a potential source of confusion for data interpretation and integration in science, at a global level.

Regarding plant science, a first step towards achieving controlled vocabularies, which allow the integration of data across disciplines, was taken by the plant ontology consortium (http://www.plantontology.org/; Cooper *et al*., 2012; Garnier *et al*., 2016). More recently, the *Thesaurus of Plant Characteristics* (TOP) was further released to help solve heterogeneity in the field of plant functional ecology (Garnier *et al*., 2017; http://top‐thesaurus.org). The TOP provides names, definitions, synonyms and related terms for *c*. 850 plant characteristics, and most particularly plant traits. The work conducted during the preparation of this handbook, and particularly the work made on defining and conceptualising the dozens of root traits included in this handbook, will be used to enrich the TOP with new concepts pertaining to root structure and function. All root trait definitions provided here will be progressively subjected to the critical assessment of a range of root scientists, and are therefore likely to be gradually improved via the online TOP interface following the handbook publication.

### Semantic information provided in the handbook

For each trait dealt within this handbook, and before describing protocols, we provide some information to reduce the semantic heterogeneity in the fields covered. This is:

(**1**) *A common trait name*: a name that is generally preferred and used by the researchers’ community.
(**2**) *A formalised trait name*, but only in cases when it differs from the common trait name: as for the characteristics previously defined in the TOP, root traits are modelled based on the Entity‐Quality model, used for the description of phenotypes in the field of genetics (see e.g. Mungall *et al*., 2010). These descriptions consist of the entity that is observed (for example, a root of a given order), and the specific quality of that entity (for example, diameter, length). A trait is therefore composed of a combination of at least one ‘entity’ and one ‘quality’, and is defined as ‘an entity having a quality’ (for instance ‘root tip diameter’ (root tip (entity) diameter (quality)), see Table 1).

**Table 1:** Examples of root traits modelled using the Entity‐Quality model (‘EQ’ model).

Common trait name	Formalised trait name	Entity	Quality	Frequent abbreviation	Commonly used unit
Specific root length	Root specific length	Root	Specific length	SRL	m g^−1^
Root nitrogen concentration	Root nitrogen content per unit mass	Root	Nitrogen content per unit mass	RNC	mg g^−1^
Root hair density	Root hair density	Root	Hair density	–	mm^−1^
Vertical root mass distribution index	Root vertical mass distribution index	Root	Vertical mass distribution index	β	no units
Nitrogen‐fixation ability	Root nitrogen‐fixation ability	Root	Nitrogen‐fixation ability	–	categories: N_2_ fixing, non‐N_2_ fixing

Note that the generic entity ‘root’ is used in these examples, but that most qualities could be associated to different entities such as ‘first‐order root’ or ‘transport root’.

For below‐ground organs of plants, the same quality can be associated with many types of entities (e.g. different organs, different root orders or different root diameter thresholds). Therefore, to avoid multiplying the definitions of traits across all potential entities, we have only used the generic term of ‘root’ (such as in ‘root’ nitrogen concentration). Nonetheless, exceptions were made in the few cases for which traits were typically connected to only one specific organ (e.g. ‘root tip’ diameter).

(**3**) *A definition*: the definition of a trait follows the formal name providing the entities, qualities and their relationships. Whenever possible, the definitions are based on concepts of entities and qualities from existing vocabularies or concepts. The definition given for a concept is free of any information pertaining to measurement protocol or methodological information. For example, the trait ‘root dry mass’ consists of the entity ‘root’ and the quality ‘dry mass’, and the definition for this characteristic is: ‘the mass of a root being dried’, and not ‘the mass of a root being dried at 65°C for 1 h in the oven’, which would then include measurement standards and protocol information.
(**4**) *Additional information*: this includes element of context for this trait measurement, the typical units most commonly used and the most frequent abbreviation.

### A note on abbreviations

In root ecology, the common use of a range of root entities calls for a homogeneous system of abbreviation for root trait names (i.e. entity + quality) that takes into account this diversity of entities. Currently, most trait names are abbreviated based on root quality and the generic entity ‘root’, which does not differentiate between below‐ground plant entities and therefore introduces confusion by indifferently referring to several potential traits. For instance, root N concentration (RNC) can refer to ‘root nitrogen concentration of the first‐order roots’, to ‘root nitrogen concentration of the entire root system’, to ‘root nitrogen concentration of the shoot‐borne roots’, to ‘root nitrogen concentration of the roots < 2 mm in diameter’ or to ‘root nitrogen concentration of the absorptive roots’. As authors increasingly commonly measure traits on several entities even within the same study, and traits measured on different entities often carry different ecological meaning, it is recommendable to integrate the notion of entity within commonly used trait abbreviations.

To be intuitively understandable by readers, and to be readily adopted by researchers, we argue that such abbreviation systems should build on currently acknowledged trait ‘common name’ abbreviations (e.g. specific root length (SRL)). Propositions that would question the current trait ‘common name’ abbreviation (e.g. by proposing an alternative ‘formal name’ abbreviation, e.g. root specific length, RSL; or by inserting the notion of entity within the existing trait abbreviation, for example SR_1st_L) are likely to create much confusion to the readers. Among the large range of trait abbreviations already available, one way to include the notion of entity consistently, unambiguously, and to respect the flow of the trait ‘common name’ enunciation is therefore to ident the entity information at the end of the current trait abbreviation. As such ‘SRL’ could become either of ‘specific root length of the first‐order roots (SRL_1st_)’, ‘specific root length of the whole root system (SRL_wrs_)’, ‘specific root length of the shoot‐borne roots (SRL_sbr_)’, ‘specific root length of the roots < 2 mm in diameter (SRL_<2 mm_)’, ‘specific root length of the absorptive roots (SRL_abr_)’, etc.

## 3 Species‐level vs ecosystem‐level measurements

Typically, functional traits are measured at the level of individual organisms, which are then referred to by their species name. However, it is common and valuable to study organism responses and effects on ecosystem properties at the level of ecosystems. In this context, environmental parameters can be linked to the functional structure of communities formed by multiple organisms, and this functional structure further influences ecosystem properties (*cf*. Enquist *et al*., 2015; Garnier *et al*., 2016). This functional structure is referred to as ‘community functional parameter’ (CFP; Violle *et al*., 2007) or ‘community trait’. There are two main ways to measure CFP of organism traits at the ecosystem scale. First, traits measured at the species level can be scaled up into CFP by multiplying trait values of organisms present in a community by the biomass (or the area for leaves; or length for roots) of organs on which the trait values are measured expressed per unit ground area (Lavorel & Garnier, 2002; Violle *et al*., 2007; Garnier *et al*., 2016). Second, CFPs can be directly measured at the ecosystem scale using measurements per ground area (e.g. remote sensing measurements; measurements averaged across a spatially explicit sampling scheme). With respect to root sampling, the latter method is increasingly used due to the difficulties associated to separating root by species in soil samples taken from ecosystem showing a diversity of species. The main practical difference with trait measurements is that root samples are treated as one homogeneous sample rather than sorted out by species. Discussing the practical consequences of each approach to scaling up traits to CFP is beyond the scope of this handbook as such debate is currently not resolved. Nonetheless, it is becoming clear that organism community structure and its influence on ecosystem properties often cannot be reduced to one simple measure of CFP (Enquist *et al*., 2015; Garnier *et al*., 2016). CFP values contain, for instance, no information with regard to the range of trait values within the community, the dominance of some values over others, or the presence of several groups of trait values (such as in a bimodal distribution), all of which can have major consequences for the properties of ecosystems (e.g. Valencia *et al*., 2015; Violle *et al*., 2017).

Although mean, variance and several other indices of functional diversity (e.g. functional richness, evenness and dispersion) are the most commonly used, four moments of the community trait distribution (mean, variance, skewness, kurtosis) have also been more recently highlighted to assess the links between environmental parameters, community functional structure and ecosystem properties (Enquist *et al*., 2015). Such an approach can only be based on the measurement of traits at the organism (or organ, e.g. roots) level and the quantification of organism (or organ) relative abundance (in % mass or cover) per ground area on a large range of organisms, rather than via direct measurement of CFP.

Measurement of CFP based on species‐level measurements typically require that sampled species represent at least 80% of the plant community biomass (Pakeman & Quested, 2007). This minimum threshold is sometimes translated as above‐ground cover, but such estimate would need to be calculated on the total cover of the vegetation, which often exceeds 100% cover as vegetation strata overlay each other. However, estimating functional diversity indices, variance, skewness or kurtosis requires more thorough sampling of species (typically higher than 95% of community biomass or cover). Due to the difficulty in assessing root abundance for each species *in situ*, above‐ground plant cover (or biomass) is often used as a surrogate for root abundance, but this cannot be widely recommended as it is a major source of error due to large variation in species leaf, stem and root mass fractions (Poorter *et al*., 2012b) or fine‐root mass fraction (Freschet *et al*., 2015a). Manually sorting out roots by species is tedious or often impossible, but several other methods exist (see section **VI. 4. Separating roots by species**).

Direct measurements of CFP require the sampling of a large number of soil cores to represent the community heterogeneity. Multiple cores can be pooled to obtain a lower number of composite soil samples representative of the plant community but, in such instances, information on spatial heterogeneity is lost. Again, parameters related to the functional diversity cannot be determined.

## 4 Below‐ground plant entities and root classifications

All studies of below‐ground plant parts face a common challenge of defining which below‐ground entities and what type of roots should be sampled and measured. Depending on the species studied and the specific question being addressed, it may be most appropriate to collect and measure the whole root system as one entity, to focus measurements on coarse roots or fine roots only, or to further subdivide and classify roots and other below‐ground plant entities into precise categories (see Box 1). However, how to decide on appropriate subdivisions is not always clear and has been an important topic of discussion stretching back decades (Cannon, 1949; Böhm, 1979; Sutton & Tinus, 1983; Fitter, 1987; Pagès & Kervella, 1990; Berntson, 1997; Hishi, 2007; Zobel, 2011; McCormack *et al*., 2015a).

Box 1 **Definition of selected below‐ground plant entities and root classification schemes**

From the entire below‐ground system of plants, several main entities can be distinguished that show characteristic growth patterns and/or have a different developmental origin. As these entities often serve different functions, and recent work has shown that many of the main entities are differentially genetically regulated, they are usually considered separately in below‐ground studies with regard to many trait measurements:

**Absorptive root**: fine root with dominantly absorptive function. Synonym to noncambial root; antonym to transport root.

**Fig. 2:**
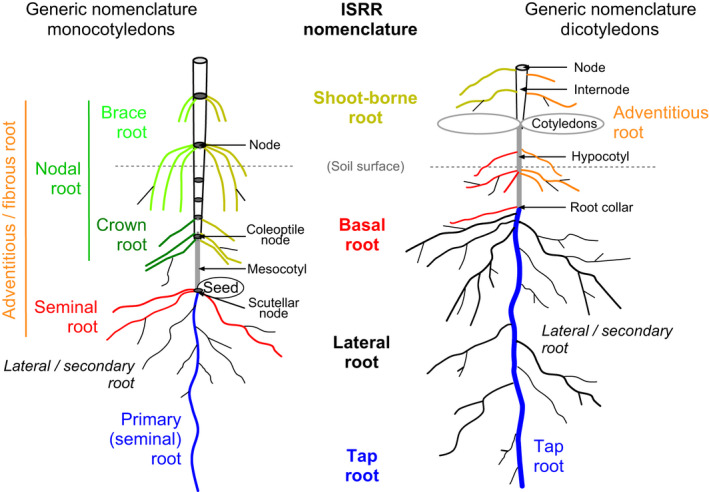
Schematic presentations of generic root nomenclature in monocotyledonous (left side) and dicotyledonous (right side) plants and the corresponding nomenclature as proposed by the International Society of Root Research (ISRR; centre); colours of nomenclature match the respective roots in the drawing; lines indicate superordinate terms. Roots potentially originating from the scutellar node (e.g. in wheat) are not drawn. See Box 1 for further information on root entities and synonyms; drawing not to scale.

**Adventitious root**: root formed from any nonroot tissue; encompasses basal and shoot‐borne roots (Fig. 2).

**Basal root**: root originating from the hypocotyl (or mesocotyl in monocots; Fig. 2); except for the primary (seminal) root, the seminal roots of monocots are considered basal roots.

**Fig. 3:**
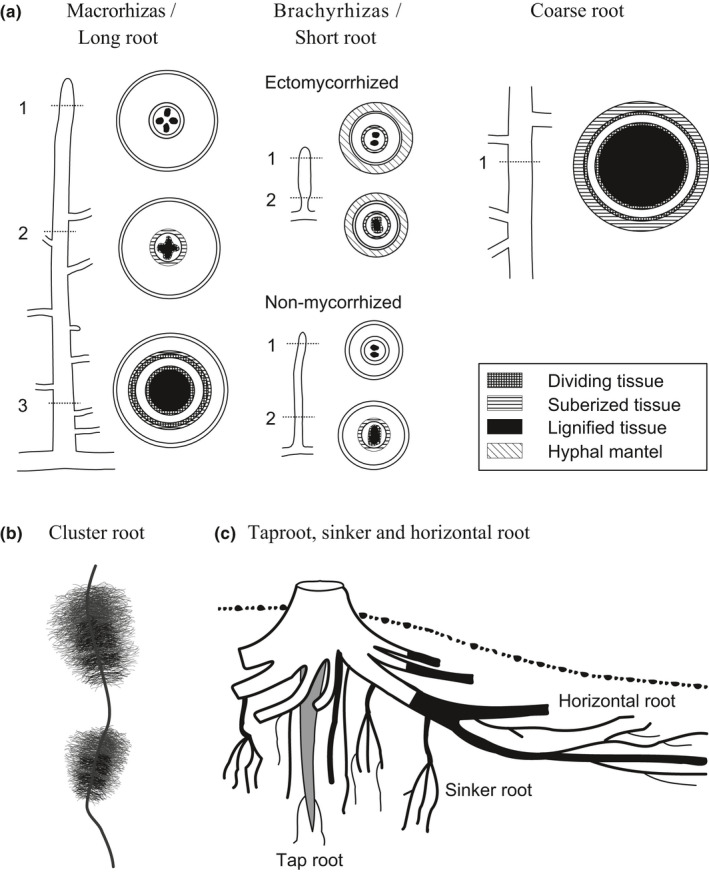
Schematic presentations of common root nomenclatures related to specific root morphological and anatomical traits. (a) Macrorhiza and brachyrhiza (fine roots) and woody coarse roots (example: *Tilia* sp.; modified after Kubíková, 1967). *Macrorhiza*, description of layers from periphery to centre: (1) rhizodermis, cortex, stage 0 endodermis, stele with four protoxylem groups; (2) rhizodermis, cortex, stage I endodermis, stele with first metaphloem and ‐xylem; (3) rhizodermis, cortex, stage I–II endodermis, pericycle, phloem with parenchyma, cambium, xylem; *Brachyrhiza with ectomycorrhizal symbiont*: (1) mycorrhizal mantle, rhizodermis, cortex, stage I endodermis, stele with two protoxylem groups; (2) mycorrhizal mantle, rhizodermis, cortex, stage I–II endodermis, phloem, cambium, xylem; *Nonmycorrhized brachyrhiza*: (1) rhizodermis, cortex, stage 0 endodermis, stele with two protoxylem groups; (2) rhizodermis, cortex, stage 1 endodermis, phloem, cambium, xylem. *Woody coarse root*: l) periderm, phellogen, secondary phloem, vascular cambium, secondary xylem. Dividing (cross‐hatch), lignified (filled) or suberised (horizontal hatch) tissues and hyphal mantel (diagonal hatch) are indicated. (b) Cluster root with two groups of abundant, short lateral roots (rootlets) with root hairs. (c) Taproot, sinker and horizontal roots in a schematic tree root system. See Box 1 and text for further information on root entities and synonyms; drawings not to scale.

**Brachyrhiza**: short, thin root with a determinate growth, often colonised by mycorrhizal fungi (Fig. 3a); synonym of short root, feeder root, antonym to macrorhiza.

**Bulb**: unit for vegetative propagation with short/flattened stem featuring fleshy leaves; adventitious roots develop from the stem.

**Cluster root**: bottle‐brush‐like or Christmas‐tree‐like structure of short lateral roots (‘rootlets’) on a main axis with a dense packing of (short‐lived) root hairs (Fig. 3b). This structure often releases carboxylates into the rhizosphere, therefore solubilising poorly available nutrients (e.g. P) within the soil. Synonym of proteoid root, dauciform root.

**Coarse root**: root with a relatively large diameter, often operationally defined as all roots > 2 mm in diameter that are generally woody, that is lignified, with clear secondary development (Fig. 3a).

**Contractile root**: root with the ability to contract, pulling the shoot closer to the ground or bulbs deeper into soil.

**Crown root**: synonym for nodal root of monocots, often further separated into crown root (on the coleoptile node or other leaf nodes below ground) and brace root (on upper leaf nodes above ground; Fig. 2).

**Feeder root**: synonym to brachyrhiza or short root in woody plants.

**Fibrous root**: Basal and shoot‐borne roots of monocotyledonous plants; synonym to adventitious root. In woody roots often used for thinner absorptive roots and to contrast coarse roots.

**Fine root**: root with a relatively small diameter, often operationally defined as all roots ≤ 2 mm in diameter, although other diameter thresholds are used as well. These roots are generally considered to be those that lack a lignified structure (although not always) and are expected to be more active in resource acquisition than coarse roots.

**First‐order root**: term used contradictorily in several root classifications (see below). In this handbook, first‐order roots are typically referring to the most distal root of a morphometric classification (i.e. ‘root tip’; Fig. 3b).

**Hair root**: root of Ericaceous plants characterised by a reduction of vascular and cortical tissues, by the absence of root hairs, and by the presence of swollen epidermal cells occupied by mycorrhizal fungi; often forming rhizosheaths.

**Haustorial root**: intrusive cells develop at the root tip, which penetrate the cortex and endodermis of the host root to establish haustoria by the parasite. Synonym to parasitic root.

**Lateral root**: any root branching from another root (Fig. 2); frequently further divided into branching orders (1^st^ order laterals, 2^nd^ order laterals etc., Fig. 3a). Synonym to secondary root, branch root.

**Long root**: main growing axis; synonym to pioneer root, explorer root, macrorhiza, framework root in woody plants.

**Macrorhiza**: root with a thick tip and polyarch structure possessing the potential for indeterminate elongation and radial growth (Fig. 3a). Synonym of long root, pioneer root, explorer root, framework root, antonym to brachyrhiza.

**Mycorrhizal root**: roots forming a symbiotic association with a fungus; most frequently used for ectomycorrhizal root segments folding a hyphal mantel (Fig. 3a).

**Nodal root**: shoot‐borne root developing on coleoptile or upper leaf nodes (Fig. 2).

**Pioneer root**: exploratory root that sometimes develops into the framework of a root system as opposed to short or exploitative roots considered to be more absorptive. Synonym of long root, explorer root, macrorhiza or framework root.

**Primary root**: first root developing from the embryo; develops into the tap root or disintegrates (monocotyledons, Fig. 2). Synonym of radicle, embryonic root, tap root or primary seminal root.

**Rhizome**: shoot axis (sometimes swollen) that grows horizontally at or below the substrate surface and produces shoots above and adventitious roots below.

**Root**: an axis made by one subapical meristem and an anatomical structure distinct from other plant organs. It usually has a monopodial structure, but by extension it can have a sympodial construction when it is made by successive equivalent meristems.

**Root hair**: root epidermal cell that develops from a trichoblast, generally extending outward from the root axis increasing absorptive surface area.

**Root nodule**: organ part that is an outgrowth of a root and inhabited by nitrogen‐fixing bacteria.

**Root tip**: organ part which is the apical portion of the root, and includes the root apical meristem (and root cap). Synonym of root apex and first‐order root (in a centripetal classification).

**Secondary root**: synonymously used for lateral root or branch root (Fig. 2).

**Seminal root**: root that originates from the embryonic plant in monocotyledons (Fig. 2).

**Shoot‐bearing root**: root that is able to produce adventitious buds (outside of stem and not derived from stem apical meristem) that sprout spontaneously or after injury to form new above‐ground parts.

**Sinker root**: root that penetrates deeply and vertically into the soil (Fig. 3c).

**Shoot‐borne root**: root originating from a shoot axis, encompassing nodal and internodal roots (Fig. 2); together with basal roots a synonym of adventitious root.

**Storage root**: root axis that is radially enlarged for storage and asexual propagation; develops from a tap root, adventitious root or their laterals.

**Tap root**: first root to emerge from the seed that usually forms the central axis of the root system (Figs 2, 3c); synonym of radicle and primary (seminal) root.

**Transport root**: fine root with reduced absorptive functionality and dominantly transport function; synonym to cambial root, antonym to absorptive root.

**Tuber**: shoot axis that is radially enlarged.

**Fig. 4:**
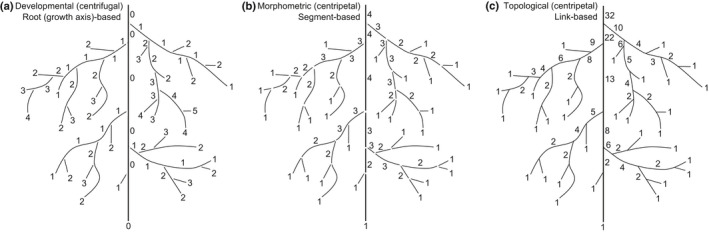
Schematic presentations of systematic root classification approaches. (a) *Developmental approach*; (b) *morphometric approach*; and (c) *topological approach*. Root growth axis (a) and root order (b, c) levels are indicated by adjacent numbers; note that highest root orders are 5, 4 and 32, respectively (a–c), corresponding to 6, 4 and 12 classification levels (a–c). The ‘0’ root order in (a) can be replaced by root class (e.g. basal root, tap root); lower root order in (b) can be refined further, for example by adding information on the mycorrhizal status; the highest order number in (c) represents the numbers of root tips. See Box 1 and text for further information on systematic root classification approaches.

Among roots, a range of classifications have been described that are useful in different contexts (Fig. 4):

**Centrifugal classification**: see developmental classification (Fig. 4a).

**Centripetal classification**: see morphometric classification (Fig. 4b).

**Developmental classification**: a root‐based (i.e. growth axis‐based) approach classifying the root‐branch hierarchy (Fig. 4a). In this context, lateral roots are referred to as first‐order laterals arising from the three major classes tap, basal or shoot‐borne root (i.e. order ‘0’); second‐order laterals arising from the first‐order lateral, and continuing in such a way that the highest order roots are the most distal (Fig. 4a). We note that alternative developmental classifications sometimes use a strict numbering system (e.g. 1^st^, 2^nd^, 3^rd^ or primary, secondary, tertiary etc.) without reference to root classes. Synonym of centrifugal classification.

**Fig. 5:**
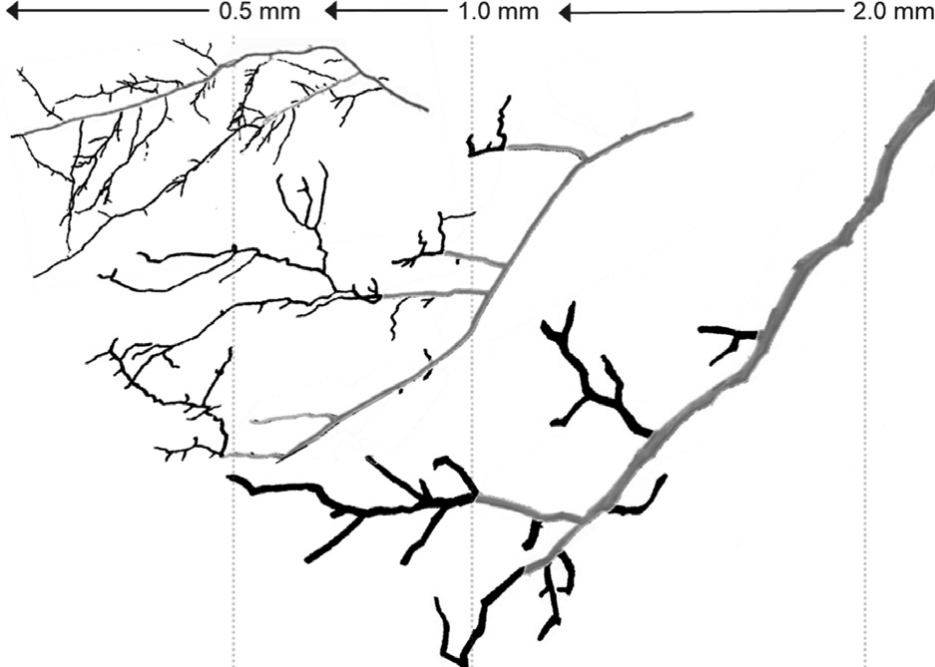
Root branches of three architecturally diverse, co‐occurring subtropical species, demonstrating the functional classification approach (i.e. absorptive and transport fine roots) and the variable number of morphometric (centripetal) fine‐root orders that fall below different diameter cut‐offs (0.5, 1.0, and 2.0 mm). *Schima superba* (top left) with up to five root orders ≤ 0.5 mm, including both absorptive and transport fine roots, *Choerospondias axillaris* (middle) with three root orders ≤ 0.5 mm and only including absorptive fine roots, and *Cinnamomum austrosinense* (bottom right) with no roots ≤ 0.5 mm. Black, absorptive fine roots; grey, transport fine roots (modified after McCormack *et al*., 2015a).

**Functional classification**: a classification system whereby the broad category of fine roots is subdivided into functionally similar pools of roots (i.e. absorptive roots and transport roots; Fig. 5), combing functionally similar root orders (as defined by the morphometric classification).

**Morphometric classification**: a segment order‐based approach to classify the root‐branch hierarchy where distal root segments are first‐order and parent root segments are higher order (e.g. second‐order roots, third‐order roots, etc.; Fig. 4b). Synonym of stream‐order (‘Strahler’) classification and centripetal classification.

**Topological classification**: centrifugal or centripetal, link‐based classification systems based on mathematical trees, combining aspects of developmental and morphometric classifications, emphasising the hierarchical description of the connection of root segments to one another (Fig. 4c).

Regarding roots in particular, there are several common practices used to classify roots and each may have certain advantages and relevance, depending on the specific questions being addressed and traits being measured. In some cases, it may be beneficial to measure the same trait across multiple root classifications as each may provide different, useful information on different aspects of root and root system function (Freschet & Roumet, 2017). In other cases, limiting measurements to a specific root segment that can be repeated precisely may facilitate broader comparisons across species and environments for some root traits (Pregitzer *et al*., 2002). Below, we discuss common practices for classifying roots (see also Box 1) followed by additional considerations of potential functional variation within a classification and along the longitudinal axis of a root segment. For discussion of different classifications of entire root systems, see section **X. Root system architecture**.

Additionally, despite the general focus of plant ecologists on roots, it is important to stress that plants harbour other below‐ground organs, including leaf‐derived and stem‐derived organs (e.g. bulbs, rhizomes). Including such organs in one’s study may be relevant in some cases, as these can account for a large proportion of plant biomass allocated below ground (e.g. Ringselle *et al*., 2017) and play important functional roles (Klimešová *et al*., 2018). For example, rhizomes, tubers and bulbs provide plants with the ability to grow clonally and regrow after disturbance (see section **VIII. Horizontal plant mobility**) and can play critical roles in resource storage and plant anchorage (see section **IX. Below‐ground allocation**).

Finally, the location of below‐ground organs in soil has critical consequences for plant and ecosystem functioning (see section **XI. Root spatial distribution**) and, regardless of whether considering all plant organs or specific root entities, we urge researchers to carefully consider and record ‘where’ in the soil they sample. Although even more overlooked, the notion of ‘when’ to sample may also be critical. Soil is often a highly heterogeneous medium both in space and time, and plant below‐ground plant phenology does not necessarily match with above‐ground phenology (see section **XVI. Root dynamics**).

### 1. Considering whole root systems

Measurements made on whole root systems, irrespective of root diameter or topology, can be useful to describe whole‐plant functioning (e.g. resource acquisition, soil exploration strategy, anchorage) and root impacts on ecosystem processes (e.g. carbon cycling, soil reinforcement) (Freschet & Roumet, 2017). For instance, for both woody and herbaceous species, whole root systems include roots of high longevity or growing over long distances that play critical roles in soil stabilisation (e.g. bridging soil layers and preventing landslides; Stokes *et al*., 2009) and water and nutrient transport on the large scale (Lynch, 2011; Comas *et al*., 2013). Nevertheless, in the field, sampling of whole root systems can often not be easily implemented (especially for large and deep‐rooted species), and therefore complicates trait comparisons. Whether the sampling of whole root systems is feasible should be assessed on a case‐by‐case basis, depending on field site, available resources, as well as the research question. In particular, it is important to consider that obtaining estimates of some traits considered on the whole root system does not necessarily require the excavation of the entire plant. Instead, this can be achieved by conducting measurements on a subset of a plant (e.g. main roots for root growth angle; see section **X. 2. Root growth angles**) or using specific sampling schemes (e.g. root vertical or horizontal distribution; see section **XI. Root spatial distribution**). To improve the comparability of results among species and studies and the interpretation of measured trait values, sampling methodologies should include explicit description of the root entities harvested (see Box 1; Fig. 2) and basic traits such as the root diameter range included. One must further consider that most traits measured on whole root systems are strongly dependent on plant ontogenetic stage and may therefore exhibit large within‐species variation, seasonally and among individuals, further highlighting the importance of providing a range of metadata within scientific reports (see section **V. Contextualisation and reuse of data**).

Measurements made on or representing entire root systems are not appropriate in a range of situations. Indeed, there is some degree of specialisation within a root system (Waisel & Eshel, 2002) and multiple changes in root trait values occur along root axes and among segments (Fig. 3). For example, in short‐term C cycling, the nonlignified tissues of herbaceous root systems suggest generally high turnover rates and decomposition rates throughout the root system (despite variations among root orders; for example Xiong *et al*., 2013), whereas only the most distal orders of woody species show high turnover rates (McCormack *et al*., 2015a). Among other root entities, fine roots have been the focus of most research as they are generally considered as critical for some root functions, including nutrient and water acquisition, association with symbionts, and carbon exudation (Hodge *et al*., 2009; Freschet & Roumet, 2017), and these can be further refined into more precise functional categories, as discussed below. Therefore, while several functions of roots can be adequately measured on whole root systems, measurement of many root functions will require the use of additional root classifications to be precisely represented and compared across plant individuals and species.

### 2. Ontogenetic and developmental classification of roots

Several different points of view taken on the root systems have led to several classifications of roots, and a profusion of terms in the general vocabulary.

#### Origin of roots in the developmental schema of the plant

Many authors have based their classification on the place and time of emergence of roots. For example, the developmental genetic classification adopted by the ISRR (International Society of Root Research) and presented by Zobel and Waisel (2010) defined a framework of four different classes of roots: **tap root,** derived from the central embryonic root (the radicle); **basal root,** originating from the hypocotyl or mesocotyl; **shoot‐borne root**, inserted from leaf nodes or internodal sections; **lateral root**, originating from each of three previously defined root classes (Box 1; Fig. 2). Based on genetic evidence (e.g. Hochholdinger *et al*., 2004; Zobel, 2016a), the four major classes have been (preliminary) divided further into eight subclasses (Zobel, 2011). This classification, based on evidence of different genetic regulation, is especially key for any breeding approach addressing root classes independently. Indeed, some functional differences, for example differences in nutrient uptake rates, have been reported between such root classes (Waisel & Eshel, 1992; Lynch & Brown, 2001) that can be more easily traced back to genetic adaptations. Additionally, this approach allows the structuration of models of plant architectural development on common principles.

Other generic developmental classifications are close to the ISRR one, but some different terms have been defined, largely differing between monocotyledonous and dicotyledonous plants and using species‐specific terms for important crop species (Fig. 2). To keep reasonable length and focus we do not present an exhaustive list of these terms, but just included the main ones and tried to clarify the synonymy.

Starting from the plant embryo, **seminal roots** have been defined as those roots that already exist as parts of the embryonic plant, in the seed. The number of such roots can vary from 1, in many dicot species, to higher values, in Poaceae species for instance (e.g. 3–7 in maize). Among seminal roots, the single or central one is often called the **radicle** (or the **primary (seminal) root**, or the **tap root**). This root develops early and holds a central position in the root system. Various authors use the term tap root only when this particular root keeps a central and dominant position; this is usually not the case in monocots. The additional (not central) seminal roots were considered as basal roots in the classification by Zobel & Waisel (2010).

In most plant species, roots branch and give **lateral roots**, secondary roots or branch roots. The developmental orders that are defined in the section below are based on this branching process (Fig. 4). Lateral roots can form from root primordia on any type of root, even on preceding or ‘parent’ lateral roots and woody roots (Chiatante *et al*., 2010).

Many plant species, between both monocots and dicots, also develop roots directly on their shoot system. These roots that originate directly from shoots have several names: for example **adventitious roots** (Hayward, 1938), or **shoot‐borne roots**, **crown roots** or **nodal roots** (see also Box 1; Fig. 2). They can appear on various shoots and at various positions (e.g. along cuttings, on coleoptile or upper leaf nodes and internodes at the base of erected shoots, on rhizomes or stolons). These variations often require the use of specific classification systems and have led to a plethora of (species‐specific) subdivisions.

#### Specific properties, morphology and anatomy of roots

Some classifications and terms rely on developmental characteristics and anatomy of roots (Fig. 3). Therefore, the differences between root entities have been qualified by a broad nomenclature (Kubíková, 1967; Sutton & Tinus, 1983). Among these terms, some qualify the growth level and associated functional characteristics: **macro‐/brachyrhiza**; **long/short** roots (Fig. 3a); **pioneer/exploitative** roots; **indeterminate/determinate** roots; **perennial/ephemeral** roots. For example, the long roots having a continuous and high elongation rate were usually associated to soil exploration, while the short, determinate and ephemeral roots were associated with the local and transient exploitation of soil resources. Other terms intended to qualify the growth direction that eventually impact root distribution: **root sinkers**/**horizontal roots** (Fig. 3c). A common distinction has also been made by several authors relying on anatomy that also impact functional attributes: **coarse roots**, **lignified roots**, **suberised root, skeleton root** vs **fine root** without/limited secondary development (Fig. 3a). Finally, we note that some species have roots with very clear specialisations towards anchorage (e.g. **buttresses**), storage (**tuberised root**), aeration (**pneumatophore**) or resource acquisition via soil mining (**cluster root**; Fig. 3b) and parasitism (**haustorial root**). Each can be an important adaptation by plants to specific circumstances. However, they are not explicitly detailed further in the more general classifications that follow.

### 3. Root order‐based classifications

Within the root‐branch hierarchy, from the most distal root to the proximal root attached to the plant stem, there exist tremendous trait and functional diversity. Distal root orders generally display thinner, N‐rich tissues that support mycorrhizal colonisation and perform soil resource mobilisation and uptake (Guo *et al*., 2008a; Valenzuela‐Estrada *et al*., 2008; Jia *et al*., 2013). By contrast, more proximal root orders are thicker and longer lived, and generally perform transport and storage functions, especially in perennial plants with a distinct secondary growth (Valenzuela‐Estrada *et al*., 2008; Rewald *et al*., 2011). Given these notable differences, it is important that researchers be able to consistently identify roots positioned throughout the branching hierarchy. Order‐based classifications represent common and highly useful approaches to conduct repeatable and translatable assessments of root trait variation within and among plant species (Fig. 4).

There are several different names and approaches used that are considered as root‐based (growth axis‐based) or order‐based classifications (Box 1; Fig. 4), however, most of these are largely based on either counting roots or root segments (i.e. longitudinal parts of a root between branching points (‘links’)) from the most distal roots inward or starting from the most basal root and counting outward (Berntson, 1997). The **morphometric approach** (Fig. 4b) considers root segments rather than entire roots (i.e. a single longitudinal axis from its point of initiation to its tip). In this approach (Fitter, 1982), also known as **stream‐order‐based** or **centripetal approach**, the most distal roots are considered as first‐order roots while the parent root from which first‐order roots arise is called a second‐order root up to the point where two second‐order roots meet to ‘form’ a third‐order root (Fig. 4b) and so on up the branching hierarchy. By contrast, in the **developmental approach** (Fig. 4a) the most proximal roots arising from the embryo, hypocotyl/mesocotyl or shoot (i.e. tap, basal or shoot‐borne root) are typically considered as zero‐order (or first‐order) roots, while the most distal roots in the system would then be the highest order roots. The counting associated with the developmental approach attempts to follow root growth axes and patterns of root development associated with architectural and ontogenetic changes (see section **IV. 2. Ontogenetic and developmental classification of roots**). In addition, root order numbers occasionally follow nonlinear, **topological classification** (‘centripetal, link‐based approach’; Fig. 4c) schemes based on links (Fitter, 1987; Berntson, 1997) rather than segments. In this approach, the most distal roots are considered as first‐order roots similar to the morphological approach, however subsequent parental orders are assigned an order equal to the sum of orders of the two distal links. Therefore, the highest ordering number at the base of the root system equals the number of root tips.

Within the developmental approach, the naming may also be more descriptive with for example basal roots specifically referred to as basal (instead of order ‘0’; Fig. 4a) while the next roots that branch are called primary roots, then secondary roots, etc. However, it should be noted that some studies also use the terms ‘primary root’ in reference to the radical (initial embryonic root) and ‘secondary roots’ to address all lateral roots. Future studies should consider using an explicit nomenclature whenever feasible, for example 1^st^ order laterals of a basal root, combining both information on root classes (see above) and root branching orders. A unifying nomenclature and abbreviations, for example LTRT2 for secondary lateral (L) of the tap root (TRT), has been suggested by the ISRR (Zobel, 2011). Importantly, while the terminology may change, the ordering approach should be consistent and defined clearly for each study.

The developmental approach has traditionally been favoured in studies focused on young plants, herbaceous plants and cropping systems in which extraction and observation of entire (seedling) root systems is frequently possible. By contrast, the morphometric approach has often been used in studies focused on woody plants in which entire root systems often cannot be studied (as discussed above) and in herbaceous systems, in cases in which identifying the developmental branching order is difficult (Fitter, 1982). Additionally, there tends to be many more root orders in woody species, which makes accurate counting from basal to distal root challenging and highly variable. For example, Zadworny *et al*. (2017) encountered nine or 10 orders of roots in *Pinus sylvestris* before average diameters began to exceed 2 mm. Continued use of these different order‐based classifications among studies has led to some confusion. Because a developmental approach cannot be meaningfully applied to mature woody plants, or to larger plants in general, it may be argued that the developmental approach should be abandoned in favour of the morphometric approach, which, in principle, may be applied to both woody and herbaceous plants. However, the morphometric approach can lead to inconsistent identifications of basal roots. For example, if a basal or shoot‐borne root has no apparent laterals attached it would technically be counted as distal, 1^st^ order unless given further consideration. Finally, the link‐based topological approach might be most usefully applied to in‐depth analyses of branching patterns (‘magnitude’; Fitter, 1987), but may also be useful to help identify root segments with a distinct functionality and to better represent/scale nonlinear traits such as those underlying, for example, root hydraulics.

Given the potential benefits of all approaches, we refrain from dictating that one approach must be used over another. However, it will be useful for research reports to provide some explicit indications of how results from one classification may relate to the other. Moreover, it is always prudent to clearly report what approach was taken during sample collection and processing to enable readers to make appropriate inferences. For further discussion, see Fitter (1982, 1987), Berntson (1997) and Pregitzer *et al*. (2002).

### 4. Root diameter‐based classifications

Most roots, especially ‘fine roots’, have often been considered, at least implicitly, as cylindrical organs. In this perspective, diameter and length are the favoured descriptive variables. This is an efficient approximation, especially when short roots or short root segments are considered. However, it is useful to recognise that root diameter is the result of several successive developmental processes that induce variations along the root and from one root to another. At the tip of the root is the primary and turgescent structure, whose diameter can substantially vary within any root system, in relation to meristem size (Coutts, 1987; Pagès, 1995) and to environmental influences, particularly mechanical constraints (e.g. Konôpka *et al*., 2009; Bengough *et al*., 2011). Later on and further up the root axis, the root may exhibit radial growth and the initial diameter can be drastically increased. Ectomycorrhizal fungi also modify their external diameter (Fig. 3a). Root shrinkage and cortex degradation may also alter diameter in older parts of roots. Therefore, it is very important to specify correctly which diameter is considered when categorising the roots according to their diameter. Roots can be thick because they have experienced radial growth for old proximal parts, or they can be thick because they have originated from large meristems even in young distal parts (e.g. on some monocotyledonous species). Conversely, roots can be fine because their cortex has degraded or even lost, or they can be fine because they have originated from a tiny meristem.

Several authors have proposed to use the diameter as the basis of a simple classification, leading to the terms macrorhiza (thick roots, usually long) and brachyrhiza (fine roots, usually short), as defined by Kubíková (1967) and Sutton and Tinus (1983) (Fig. 3a). This concept, focusing on extreme root categories with two poles (‘heterorhizis’), has been mainly promoted on trees where the differences are clear (e.g. Krasilnikov, 1968; Kahn, 1977; Coutts, 1987), but was first used by Tschirch (1905) for herbaceous roots. macrorhiza have large apical meristems, and they give rise to long roots, up to several metres or several tens of metres. They are equipped to extend the root system and to increase the overall root volume. For this purpose, they exhibit a high growth rate and a continuous (indeterminate) elongation; they also show various forms of gravitropism and the capacity to penetrate dense and compacted soils (e.g. Materechera *et al*., 1992; Bengough *et al*., 2011). Later, these macrorhiza experience radial growth (on dicotyledonous species) and they are the main contributors to anchorage. By contrast, brachyrhiza have different roles: they locally increase the exchange surface and provide an increased number of possible sites for mycorrhizal associations. These roots stay short (several millimetres to centimetres) because they have a small apical meristem allowing a lower elongation rate compared with macrorhiza, and they can only grow during a limited period of time, often less, or much less than 1 decade (e.g. Cahn *et al*., 1989; Pagès, 1995). Therefore, they have a transient role, with opportunistic behaviours (Eissenstat *et al*., 2000). As a result, they have often been compared to leaves because of their exchange role, determinate growth pattern and possible abscission. Beyond these general considerations, it is rather difficult to specify precise values for the diameter of macrorhiza and brachyrhiza, because it depends very much on species. For example, Pagès (2014, 2016) observed eight to 10‐fold variations of minimal and maximal apical diameter across species.

Between the two extreme poles (macrorhiza, brachyrhiza), diameter classes were sometimes used to define root categories in particular species such as maize (Varney *et al*., 1991; Jordan *et al*., 1993; Wu *et al*., 2016) and *Musa* sp. (Lecompte *et al*., 2005). These works have highlighted the diversity of roots even within a single root system, and they have pointed out the relationships between diameter and several other attributes such as anatomical characteristics or functional properties of roots, particularly water conductance (Varney *et al*., 1991; Vercambre *et al*., 2002). These studies have also shown that it is possible to define a continuum of root types with intermediate roots between the two extreme poles macrorhiza and brachyrhiza. Moreover, many studies have demonstrated in different species that even the distal root diameter is not a fixed attribute of the individual root, both in dicotyledonous (Thaler & Pagès, 1996) and monocotyledonous species (Wu *et al*., 2016). It must be considered as a plastic and transient variable during individual root elongation, responding quickly to the local characteristics of the soil (mechanical resistance, oxygen availability, water content) as well as to the whole‐plant status (photoassimilate availability, nutrient status).

In this context, classifying roots solely on the basis of their diameter is prone to many potential biases. The traditional definition of fine roots as a single pool according to a diameter‐based cut‐off, commonly roots ≤ 2 mm, below which root function is primarily uptake (and loss) of resources (Vogt *et al*., 1995) suffers from some important limitations (Fig. 5). The main limitation is that the pool of roots ≤ 2 mm includes several root orders (generally between two and five or more) that differ in structure and function (McCormack *et al*., 2015a) (see section **IV. 3. Root order‐based classifications**). In comparisons among species, the definition of fine roots as all roots with a diameter ≤ 2 mm appears therefore questionable and may be especially problematic in woody species. To improve the comparison of analogous root entities among different species, alternative, lower cut‐off limits at ≤ 1 mm and ≤ 0.5 mm have also been used (e.g. Liu *et al*., 2010; Valverde‐Barrantes *et al*., 2013). However, while lower cut‐offs are likely to be closer to the true absorptive vs transport threshold in a majority of herbaceous species and some woody species, it is also likely to exclude some root entities responsible for resource acquisition, and may still include transport roots in some species, thereby essentially attracting the same criticisms as the more widely used ≤ 2 mm cut‐off. Alternatively, new approaches identifying species × environment‐specific diameter sizes for fine‐root subclasses (Montagnoli *et al*., 2018) could provide a better resolution of corresponding branching orders and root functional classification.

### 5. Root functional classification

While diameter cut‐offs related to a functional classification can be useful to accelerate the sorting of roots within a single species, they often fail when the same diameter breaks are applied across multiple species with different root morphologies (Fig. 5). The functional classification attempts to reconcile traditional approaches to classify fine roots simply by a single diameter cut‐off (e.g. all roots ≤ 2 mm in diameter) with more detailed, but very time‐consuming, designations based on individual root orders. This is done by subdividing the single fine‐root category into functionally similar categories of absorptive fine roots and transport fine roots (Fig. 5). Absorptive roots are identified based on the presence or absence of phellem (i.e. the outer layer of cork periderm; *sensu* Zadworny *et al*., 2016). The loss of phellem is associated with a decline in root absorptive function due to the expanded suberised layer within the root which limits the uptake of ions and water. Therefore, the preferred approach to identifying functional categories and standard breaks within a species should be based on prior anatomical observations. These designations may then approximately parallel either order‐based or fine‐scale diameter‐based classifications. For example, in many woody species, it is often the most distal two or three root orders that are considered most absorptive and may be grouped together (McCormack *et al*., 2015a). Alternatively, a common diameter cut‐off (e.g. 0.5 mm) may be used to separate most absorptive roots from transport roots in some cases provided prior anatomical observations corroborate this approximate division. The proportion of fine‐root mass ≤ 2 mm allocated to absorptive and transport roots varies among species (Pregitzer *et al*., 2002; Guo *et al*., 2008a; Valenzuela‐Estrada *et al*., 2008; Picon‐Cochard *et al*., 2012; Rewald *et al*., 2014). Although few direct assessments have been made, current evidence suggests that between 10 and 60% of all roots ≤ 2 mm are absorptive in woody species while the amount of fine roots classified as absorptive among nonwoody species ranges from 60 to 100% (McCormack *et al*., 2015a).

When the functional classification approach can be accurately and effectively applied, substantial amounts of processing time may be saved, while still approximating a more meaningful designation beyond pooling all roots ≤ 2 mm in diameter together. However, it is important to recognise that this effort only approximates when functional divisions probably occur. Before deciding on the use of the functional classification, several potential limitations to the approach must be considered. First, while the distal root orders and smaller diameter roots of the root system are often the most absorptive, the point within the branching hierarchy where roots transition from a more absorptive role to a more transport‐based role is not always well defined and varies largely between plant functional groups and species. For example, within many small herbaceous plants, all roots may effectively serve clear absorptive function beyond the second‐order or third‐order root, often used to subset absorptive roots in some woody species (Picon‐Cochard *et al*., 2012), or, by contrast, secondary thickening may already occur in lower root orders limiting absorption (Zobel, 2016b). In the monocots, however, second (third)‐order roots (often) do not develop secondary thickening, but do demonstrate rapid maturation that precludes or reduces absorption (McCully, 1987). Furthermore, the approximate functional break can also vary within the same species in response to local environmental conditions such that a separation between third‐order and fourth‐order roots (in woody plants) may represent an effective break in some conditions but not others (e.g. Zadworny *et al*., 2016).

Therefore, the functional classification offers a relatively rapid approach that may be used to approximate functional transitions within single pooled fine‐root group. However, more rigorous methods, for example order‐based approaches or precise developmental designations (as discussed above), are still preferable in any case where they are tractable. The functional approach is most often appropriate when the specific study or question makes an order‐based approach intractable. For example, assessments of root system biomass at either the whole‐plant or ecosystem scale are usually not possible to conduct on individual root orders. Furthermore, some methods of study make it difficult to separate roots orders (e.g. most measurements of root respiration and minirhizotron observations of root lifespan). In these cases, working to tailor measurements to specific functional pools of fine roots can offer tractable ways to make better inferences concerning measured root traits. However, whenever possible, effort should be made to conduct preliminary assessments of root structure and root anatomy (see Zadworny *et al*., 2016) to empirically define probable functional breakpoints for a given species (or even ecotype/cultivar) and environmental conditions. This will ensure that the approach can be applied most effectively. For further discussion, see Hishi (2007), McCormack *et al*. (2015) and Zobel (2016b).

### 6. Additional considerations for root classification

#### Apparent variation within ordering schemes

The goal of all root classification approaches is to provide a meaningful and repeatable way of identifying root entities in a complex root system in a way that measured root traits can be used to understand and compare important aspects of root functioning. While none of the above classifications are perfectly suited to all plant types and systems, they are each broadly applicable to some common circumstances.

However, it is still possible for important variation to exist among roots within a given classification. These differences often occur due to structural and developmental differences that may not always be clear when roots are sampled and classified. We provided one example above that may commonly occur in growing herbaceous species regarding the potential classification of unbranched basal roots as first‐order roots within the morphometric approach (see section **IV. 3. Root order‐based classifications**). In a similar example, some woody plants will occasionally produce macrorhiza/pioneer roots that might grow as unbranched, somewhat larger first‐order roots for relatively long distances. These pioneer roots often occur in response to disturbance and express notable differences from the typical ‘brachyrhiza’, short first‐order roots in terms of their morphology, anatomy, and function (Zadworny & Eissenstat, 2011; Fig 2a). Similarly, significant differences in specific respiration rates have been found between younger ‘white’ and older ‘brown’ first‐order roots of the same plant species (Rewald *et al*., 2014). Species that associate with ectomycorrhizal fungi present an additional challenge to root order classifications. In this symbiosis, the ectomycorrhizal fungus will produce a fungal mantle around the root and in many cases may induce distinct morphologies and branching patterns (Fig. 3a). The mantle itself may substantially alter, for example, the measured diameter of a root according to standard methodologies (i.e. without anatomical assessment) leading to differences in trait values between colonised and nonmycorrhizal roots.

The potential for distinct dimorphism within a single root‐branch order should be acknowledged and treated appropriately when processing roots for trait measurements. It is, however, not always clear if colonised/uncolonised (or white/brown, pioneer/feeder etc.) roots should be treated uniformly as a single root order (e.g. first order), or as its own designation (e.g. mycorrhizal first order), or perhaps as multiple orders that account for the multiple levels of ramification that may occur. When appropriate, researchers are encouraged to use additional information beyond strict order‐based classifications to classify root entities including anatomical assessments (see section **XIII. Root anatomy**), diameter (see sections **IV. 4. Root diameter‐based classifications** and **XII. 1. Mean root diameter and mode of diameter distribution**), length, and broader branching patterns in the root system (Zadworny & Eissenstat, 2011). In either case, it is imperative that researchers clearly report how root structures were sampled and subsequently analysed.

It is also important to recognise that root functions may change within individually identified roots (or root sections), as root tissues develop from the tips towards older and more basal portions of the root segment. The development of individual roots includes several processes: subapical elongation, acropetal branching, tissue maturation, cambial growth, ageing with cortex and root hair degradation. All these processes together contribute to build roots with some morphological and functional gradients, from the base with old tissues to the apex with young tissues (e.g. Gambetta *et al*., 2013). This point has heavy consequences on several functions of the root: resource uptake and exchange capacity, conducting characteristics, mechanical strength and anchorage. We recognise that sampling beyond root orders and identifying subroot segments may be tedious and often unrealistic, particularly in cases when substantial amounts of root material are needed for the trait measurement. Nonetheless, considering a long‐term growing root as a single organ may be comparable with pooling together a tree leaf with the branch on which it has grown. They have neither the same structure nor the same function. Even though there is a developmental continuity between the distal end of the root and the base, it is important to use some developmental indicators (e.g. tip zone, apical nonbranching zone, radial growth zone; see sections **XIII. Root anatomy** and **XXI. Root tip morphology and elongation**) as landmarks to define longitudinal root segments with more homogeneous functions and properties.

#### Species‐specific classification systems

It is worth noting that additional classification schemes do exist and have important relevance to particular species or systems. For example, the cluster roots (Fig. 3b) found in some plant families (e.g. Proteaceae) generally defy categorisation in typical classification approaches but may be described by their branching as either simple or compound (Lambers *et al*., 2008). Similarly, clusters of ectomycorrhizal root tips may not be adequately classified as first‐order roots for the purpose of root trait measurements.

Due to the intense level of study given to many crop species, and the critical importance of describing root function in these systems, many economically important cropping systems have well developed approaches to classifying root types and functions. These systems share similarities to developmental, order‐based approaches or even functional approaches, but they tend to be more precise and rigorous and are well suited to capture changes in roots and the entire root system throughout the developmental trajectory of a plant from seed to senescence. Maize is one important example (Giradin *et al*., 1986), but detailed root classifications also exist for wheat (Klepper *et al*., 1984), rice (Yamauchi *et al*., 1987), leguminous plants and other crop species. The detailed classification approaches used in these systems can provide important insights into how more generic classifications should be interpreted.

#### When is each root classification method appropriate?

There is no single answer to the question of when each method of root classification is most appropriate. Alternatively, a broad understanding of the advantages and drawbacks of each classification, as described above, is the key to choosing a methodology in line with one’s research question. Indeed, the same trait measurement made on different root entities can provide complementary, yet unique information with which plant functional strategies can be interpreted (Freschet & Roumet, 2017). For instance, considering entire root systems is highly relevant to the study of certain plant functions (e.g. soil exploration strategy, anchorage) and root impacts on ecosystem processes (e.g. long‐term carbon cycling, soil reinforcement) that depend on all or most below‐ground parts of plants. By contrast, using root functional classifications separating roots into absorptive fine roots vs transport roots and roots of other functions (e.g. tap roots, pioneer roots, rhizomes) is better adapted to the study of several other key plant (e.g. resource acquisition, resistance to herbivores) and ecosystem (e.g. annual carbon allocation and nutrient cycling) functions that are mainly determined by a smaller part of the root system. In addition, root order‐based classifications on single root segments, such as first‐order roots in morphometric scheme, may be particularly useful for studying the response of plants on fine temporal and spatial scales (e.g. root elongation rate, root‐penetration force in soil), and their effect on soil organisms and properties at the level of soil aggregates (e.g. soil aggregate stability). Developmental, root axis‐based classifications, potentially including information on the origin of the main axis, are key to understanding and potentially modifying (via breeding) root system development during plant ontogeny. Finally, measurements made on root diameter‐based classifications, such as ≤ 2 mm and ≤ 1 mm, appear globally relatively similar to those made on absorptive fine roots (at least for the few traits tested; Freschet & Roumet, 2017), but are increasingly recognised as nonoptimal in interspecific comparisons of root functions, at least among woody species.

## 5 Contextualisation and reuse of data

A primary feature of ecology as a scientific field – and below‐ground functional ecology is no exception to this – is that it produces large volumes of data that span diverse, individual projects. In recent years, increasing connections and collaborations across the international community of below‐ground ecologists have moved us towards a formal culture of data curation and sharing (Reichman *et al*., 2011; Hampton *et al*., 2013). To address large‐scale questions, ecologists must treat data as an enduring outcome of research, which requires that data be organised and archived for reuse beyond primary publication. The development of ecoinformatics (Jones *et al*., 2006; Michener & Jones, 2012) results from the realisation that data sharing and integration should become high priorities in ecology.

After the initial development of a range of specialised (e.g. CLO‐PLA; Klimešová & de Bello, 2009) and generalist (e.g. TRY; Kattge *et al*., 2011) databases including below‐ground plant organs, the most significant effort in this direction has been done by the community working in below‐ground functional ecology, who recently collated root trait data from across the globe into the Fine‐Root Ecology Database (fred). fred includes observations from more than 2000 plant species encompassing more than 300 types of root traits, along with metadata (i.e. information about data, necessary to interpret these data, see Michener *et al*., 1997), are now freely available for download at http://roots.ornl.gov in fred v.2.0 (McCormack *et al*., 2018). Furthermore, each new version of fred is submitted to the TRY database (Kattge *et al*., 2011) to facilitate above‐ground and below‐ground linkages. The fred database was begun to answer important questions on the variation of root traits within and among species, across environmental gradients, and with regard to other root and whole‐plant traits (Iversen *et al*., 2017). However, fred also provides an important framework for understanding the diversity of root traits that are measured by the broader community of root and rhizosphere ecologists, the methodology most commonly used, and where the largest gaps in observations lie (i.e. physiological root traits, plants in tropical ecosystems, plant communities underlain by organic soils). Importantly, fred also provides a house for new observations; this handbook will help to guide measurements in a way that the resulting data can easily find a home in fred (data can be contributed at https://roots.ornl.gov/upload). Ultimately, the more observations that are measured on a range of well described root entities, using the same methodology, the better our understanding of the important processes occurring below ground.

**Fig. 6:**
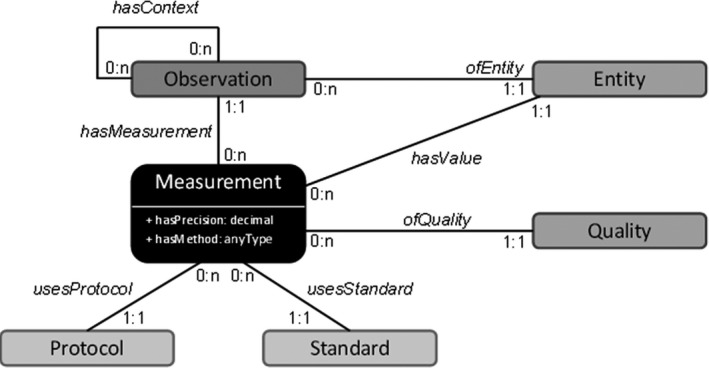
The main classes (rectangles) and properties (connections between the rectangles) of the observation and measurement ontology (OBOE) initially developed by Madin *et al*. (2007), and modified later by Saunders *et al*. (2011). An observation is made of an entity. The quality of an entity can be represented by a measure. Measures establish a relationship between the characteristics and a measurement standard via a value, and are obtained with a certain precision. Measures are carried out using a protocol in a certain place at a certain time. Observations can have multiple measures. Entities, characteristics and measurement standards constitute entry points for domain‐specific ontologies. The notations ‘1 : 1’ and ‘0 : n’ are called multiplicities: they indicate how many objects within a given class can be linked to objects of another class. For example in the relationship ‘of entity’, an Observation will be linked to only one entity (‘1 : 1’), while an entity could be linked to 0 or to n Observations (‘0 : n’).

To be shared and reused in a relevant way using publicly available databases, supplementary information should be given in addition to the primary data at stake (Fig. 6). First, the variables retained (here those describing the different root traits) should be formalised in a relevant semantic framework (see section **II. Semantics**) including variables’ entity and quality. Second, the protocols used for data collection – the main topic of this handbook – must be recorded, enabling end users to establish links between data collected in the same way. Third, measurement standards (i.e. units) must be explicit. And finally, a minimum set of data on the context of observations (location with proper georeferencing, date, species sampled, type of ecosystem, plant growth conditions, relevant environmental factors, etc.) must be given, to enable the assessment of the sources of variations driving the changes in the variables concerned. For example, while fred collects relevant metadata, only 60% of observations in fred 1.0 were georeferenced in their original publication, making it difficult to assess root trait variation across biomes and also develop linkages among databases (Iversen *et al*., 2017). The relevant community should therefore work towards an agreement on a minimum set of metadata, preferably chosen from existing frameworks such as the Ecological Metadata Language (EML; Michener *et al*., 1997). Regarding below‐ground plant traits, it is critical to consider that the resolution of current worldwide databases of soil properties is unlikely to capture the large heterogeneity in soil properties occurring at much smaller scale, stressing further the need to adequately characterise soil conditions for plant growth. Although more specific information is provided on trait–environment relationships under each respective trait section, a range of generic environmental factors have been shown to impact on values of some root traits. Below ground, this includes soil type and texture, bulk density, substrate water and nutrient availability, pH and temperature (see further discussion section **VI. 2. Laboratory experiments and sampling**). Above ground, factors such as light availability, average day and night air temperature and CO_2_ concentrations can also have effects on below‐ground traits. Finally, we note that all four criteria mentioned above are not only useful for data reuse but are also most often critical to allow an informed assessment of the validity of any experiment in answering its set of hypotheses.

## 6 Experimentation and sampling in laboratory and field

### 1. Where to do your study: the choice of laboratory or field experiments

One of the first choices to be made when designing experiments to investigate below‐ground processes is where to carry out your experiments – in the laboratory or in the field – to best meet your research aims. Glasshouses or growth chamber experiments allow for a better control of water and nutrient supply, but also light, temperature and CO_2_. Freezing or low‐temperature stress can be avoided by heaters, and lamps can be used to mitigate periods of low light. However, the daily amount of light will still vary strongly across days and seasons. Plants are mostly, but not necessarily, grown in containers of some sort, often with one plant per pot. The use of controlled and simplified environments allows for relatively easy repetition of experiments over time by the same laboratory or elsewhere (Sasse *et al*., 2019), although true replication remains challenging (Massonnet *et al*., 2010; Milcu *et al*., 2018). Controlled experiments not only benefit from lower variation, but also from easier sampling. By growing one plant per pot, interindividual root competition may be avoided and whole‐plant root systems may be collected relatively easily. Additionally, the planning of experiments is more flexible and reliable as it is less dependent on seasons. However, environmental conditions are often highly simplified and artificial, and may not represent well plant growth conditions in the field. Among other drawbacks, root growth may be rapidly constrained by the volume and shape of the containers used.

A second option is to grow plants in what ecologists would consider ‘common gardens’, and agriculturalists would call ‘field conditions’. Plants are generally sown from seed or planted as equally sized individuals. Common gardens share the advantages and disadvantages of *in situ* experiments, but the researcher has more control over factors such as planting scheme, soil preparation, fertilisation, irrigation, weed and pest management, which is likely to result in more homogeneous growth conditions. Furthermore, common gardens allow easier sampling of species monocultures and mixtures and assessment of species‐specific root traits. However, interannual variability is still likely to be high. For example, Poorter *et al*. (2016) found an average interannual correlation *r* ^2^ of only 0.08 for yield trials with genotypes of different crop species across consecutive years.

As a third option, *in situ* field experiments in existing vegetation have the advantage that they challenge plants with realistic conditions (Poorter *et al*., 2016; Schuman & Baldwin, 2018) for long periods of time and without any artificial soil volume restriction. Drawbacks include potentially high temporal and spatial variation in environmental factors important for plant growth such as light, temperature, nutrient and water availability. Furthermore, field experiments have a risk of disturbance, including herbivory and storm damage, which add additional sources of variability and may interact with the applied treatments. Therefore, it may well be that a repetition of the experiment in 2 different years provides very different results, even when done at the same location. However, environmental variation and disturbance can provide a useful context for understanding variation in below‐ground processes in response to changing environmental conditions, especially if the experiment is run for multiple years or decades (Norby *et al*., 2010).

The choice for any of these options depends on the level of control the researcher wants to achieve, knowing that this control often comes with a loss of realism. Ideally, it would be good to test hypotheses under both laboratory and field or *in situ* conditions.

### 2. Laboratory experiments and sampling

Given the advantages of repeatable testing and the ease with which plant growth can be manipulated and controlled, experiments in glasshouses and growth rooms are continuously popular. There is a wide range of methodologies and options used for optimising root measurement in controlled experiments, all of which have pros and cons. Below, we discuss a number of them.

#### a. Realistic vs artificial environmental conditions

Performing experiments under controlled conditions comes at the risk of losing relevance to the ‘outside world’, where plants are growing in heterogeneous, fluctuating and competitive environments. Plants grown under controlled conditions differ in many aspects from those grown outside. Generally, laboratory‐grown plants are harvested at a young age, have much higher growth rates, thinner leaves, smaller size, higher leaf N concentrations, and lower root tissue density (Poorter *et al*., 2016). Additionally, they may show lower rooting depth (as this is limited by the pot size) and higher SRL (Freschet *et al*., 2017). One may improve the correlation between laboratory and field by altering major environmental factors. First, one option is to change environmental factors, such as temperature and light, to the average levels which plants experience outside during the growing season (Junker *et al*., 2015). Second, one may simulate fluctuations in time, for example by adjusting light frequently during the day and temperature during day and night. Third, variation can also be imposed by changing conditions from day to day (Poorter *et al*., 2016). However, in view of minimising one’s experimental costs and environmental footprint, as well as for maximising the replicability of results in field conditions, researchers should always consider whether a minimal buffering of external conditions, in line with natural seasonal fluctuations, may not be the most appropriate set‐up for their research question.

#### b. Substrate types

The choice of a rooting substrate or medium for growing plants is challenging, as the substrate influences the growth conditions of the roots, as well as the ease with which they can be observed or sampled. ‘Substrates’ that facilitate easy observations are aeroponics (de Dorlodot *et al*., 2005), agar (Xu *et al*., 2013), hydroponics (Hoagland, 1933) and paper pouches (Le Marié *et al*., 2014). These systems, however, form a highly artificial growth environment and often lack sufficient support to grow plants to larger sizes. They generally produce plants with root traits that differ highly from these grown in more realistic soil substrates (Shrestha *et al*., 2014; but see Bengough *et al*., 2004). In horticultural practice, the ideal substrate for plant growth has high water holding capacity, good rehydration after drying, good aeration, stable structure, optimal pH, high cation‐exchange capacity, free of toxic compounds, and is low on microbial activity as well as free of pests and weeds (Gruda *et al*., 2013). Light, porous materials such as peat, bark, compost, rock wool, vermiculite, perlite or zeolite are often recommended, as they give good plant growth and allow easy handling (Handreck *et al*., 2002; Landis *et al*., 2014). For root research, however, these materials might be less desirable as fine roots tend to grow into the pores, which strongly complicates root sampling, cleaning and subsequent measurements. Expanded clay granules (fritted or calcined) provide a very reproducible environment, with a high water holding capacity and good aeration (Van Bavel *et al*., 1978). However, very fine roots may still be attached to the granules, depending on species. A very easy substrate for growing plants and harvesting roots is sand. Sand, however, needs to be sufficiently coarse to allow drainage, needs to be rounded (river sand) to avoid high penetration resistance, and must have a proper pH, as many species do not thrive on calcareous sands with a pH higher than 7. Sand does not hold much water and nutrients, and therefore must be fertigated frequently. Mixing the sand with small amounts (*c*. 10% v/v) of vermiculite or zeolite can greatly improve the growth conditions with respect to many of the above‐mentioned variables but is likely to complicate root cleaning.

Soils collected in a plant’s natural habitat offer the most realistic substrate. A drawback of using field soil, however, might be its variable quality, as the soil could contain weeds and pests, and might have poor aeration, especially when soil structure is lost during preparation (Douglass *et al*., 2009; Landis *et al*., 2014). To mitigate the latter problem, some researchers have used intact soil cores for growing plants. These cores have a natural soil structure of micropores and macropores that is associated with better drainage and aeration (Jakobsen *et al*., 2001). This approach better reflects field conditions, but may also be associated with higher among‐pot variation compared with more uniform substrates and blends (e.g. soil plus sand).

An important consideration when choosing substrate is the strong effect that substrate density and texture has on root development. Generally, dense substrates with coarse textures have high penetration resistance, resulting in slower‐elongating roots with larger diameters and lower SRL (Bengough *et al*., 2011). Moreover, plasticity responses to substrate density and/or texture may vary strongly by genotype and species (Materechera *et al*., 1991; Rogers *et al*., 2016). The ideal substrate for many experiments may actually be a mixture of two or more different substrates. In this way one can take advantage of the various properties of the different materials. A mixture of river sand and field soil, for example, allows both good drainage and some microbial colonisation. Mixing is often best achieved by tumbling with, for example, a cement mixer.

Other preparatory steps of the substrate might involve drying, sieving, mixing, sterilisation, inoculation, filling of pots and compaction. For root research, special care has to be given to how the soil is compacted, as it greatly affects root growth. Filling by weight and compacting the soil to reach a similar volume within each pot is one way to ensure a similar amount of soil and bulk density among replicates. Although intended to have a reproducible and homogeneous medium with known water content and microbial activity, these preparatory steps may damage soil structure, changing soil chemistry and biology. While soil chemistry might equilibrate relatively rapidly after preparation, one may expect temporal peaks in microbial activity, whereas the reestablishment of fungal networks will last longer. It may therefore be necessary to let the soil rest for some time (at least 1 wk) before sowing or planting is started.

#### c. Pot size, shape and material

Pot size has profound effects on plant growth and functioning. Over a large trajectory of pot sizes, each doubling in pot volume will increase plant biomass by *c*. 30–50% (Poorter *et al*., 2012c). Strong reductions in plant biomass might be avoided by estimating the final plant biomass before the start of the experiment and choosing pot volumes such that plant biomass/soil volume remains <1–2 g l^−1^ (Poorter *et al*., 2012c). Regarding root traits, particularly architectural traits, large pots will provide more realistic conditions for root growth and meaningful trait expression (see Kutschera *et al*., 1992; Kutschera & Lichtenegger, 2002 for drawings of a large number of excavated root systems from plants grown in the field). The shape of the pot is also relevant. To avoid roots to become pot bound and circle along the pot wall, with little contact with the rooting substrate, special pots with ridges or fabric pots can be used that cause roots to grow downwards or inwards (Gilman *et al*., 2010; Landis *et al*., 2014). Deep pots will defer the time that roots reach the bottom of the pot and allow root systems to express their potential for deep biomass allocation to some extent. Wide pots will decrease the chance that roots get pot bound on their way down, although this depends strongly on the root angle at which a given species grows. As discussed above, using a substrate with a somewhat higher density may actually improve the relevance of experiments for the field and slow down root growth such that the roots may reach the bottom less quickly. Deep pots may also ensure that the top part of the soil will drain better (Passioura, 2006). When using deep pots, for example tubular ones made of PVC pipes, one may place long plastic sleeves inside the pots before filling them with substrate. If there is no water between the pot walls and the sleeves, they can be pulled out of the pots, allowing good access of the roots and the possibility to study root distribution within the pot (Merchuk‐Ovnat *et al*., 2017).

These days, most pots used for research are made from plastic. Such pots are sturdy and light weight, and therefore practical in use. They are often dark‐grey or black, which implies they may warm up when exposed to sunlight (see temperature section). Compared with clay or fabric pots they have little evaporative cooling (Tauer & Cole, 2009). In all of these containers, plants have to be harvested destructively to follow root development over time. An alternative option is to use pots or rhizotrons which are partly made of transparent material, for example Perspex and a removable nontransparent cover. This allows the recurrent inspection of the roots growing in between the Perspex and the root substrate, enabling an evaluation of the dynamics of root growth and architecture (Nagel *et al*., 2012). Finally, flat rhizotrons spaced with pins allow for removal of the substrate while keeping the spatial distribution of roots intact (Schuurman & Goedewaagen, 1965; Singh *et al*., 2012).

#### d. Temperature

Root temperature is strongly determined by the temperature of the soil substrate. In nature, most soil temperatures will follow air temperature, but with strongly dampened circadian variation (Nobel, 2009; Poorter *et al*., 2016). In glasshouses or growth chambers, pot temperature will follow air temperature, with some delay depending on pot size, pot material, substrate and water content. If sunbeams hit the side wall of dark‐coloured plastic pots, substrate temperature may rise up to 20°C above air temperature (Markham *et al*., 2011; H. Poorter & R. Pieruschka, personal observation). High soil temperatures might be avoided by using clay or fabric pots, white (outer) pots, by packing dark plastic pots in aluminium foil, or by placing pots in premade holes in wooden plates which are constructed above glasshouse tables.

#### e. Watering

Plant species differ in their water requirements. Generally, terrestrial plant species do not like either very wet or very dry conditions. The rate at which pots dry out is determined by the rate of evapotranspiration divided by the amount of water in the pot. Soil water amount is determined by pot size and water holding capacity of the substrate. The evapotranspiration rate is determined by many factors, including plant size, light intensity and air moisture content. Differences in growth can easily cause variation in the rate with which pots dry, which may introduce a confounding factor when comparing treatments or species. Therefore, it is better to water plants based on measurements of water content of individual pots, for example by rewatering to the original weight. Another consideration is that soil drying–wetting cycles may cause significant air movement in the soil, which can improve oxygen levels (Heiskanen, 1995).

Not only amount and frequency, but also quality of the water should be considered. Tap water often has high pH and general hardness (GH) and might be unsuitable, especially in unbuffered substrates. Alternatively, rain or deionised water might be used. The temperature of the water should not be overlooked and can be controlled by watering from a canister inside the glasshouse or growth chamber. pH or nutrient content (see below) of the water can also be easily monitored and adjusted inside this canister. With a simple electric pump and a timer the water might be distributed to the plants using for example drippers or flooding trays. The amount of water delivered by drippers may be quite variable, which can be corrected by regular adjustment of the water content based on pot weight. Alternatively, plants can be positioned on electronic scales and followed in real time (Meurs & Stanghellini, 1991).

Water might be given from the top or from the bottom and this can cause differences in nutrient gradients. Watering from the top can leach mobile nutrients downwards and, when in excess, out of the pot. Watering from the bottom might cause nutrients to move upwards, which will then show up as an accumulation of salts at the soil surface in high‐evaporative environments. High evaporation can be avoided by covering the substrate with – for example – small white plastic balls. They have the additional advantage that growth of algae and mosses will be avoided. If the amount of water given to the plant and the water content of the pot are measured over time, plant transpiration rates might be derived. When using very tall pots (> 0.5 m), water from the bottom might not percolate to the top soil fast enough, causing too dry conditions at the top where most roots reside. Finally, moist conditions below the pot may cause roots to grow out of the pot. This may be avoided by gluing a fine mesh over the drainage holes of the pot.

#### f. Nutrients

Like water, nutrient demand increases with plant size and growth rate. When plants are grown in pots, and the interest of the researcher is only that nutrient levels should not become growth limiting, a simple solution might be to mix soil with slow‐release fertiliser. As a coarse estimate, 5% of the projected final plant biomass can be taken as the amount of N that has to be provided by soil and fertiliser. If better control is needed, more specific fertilisation is required. Many fertilisers are available commercially, but they often differ from country to country and are not always well specified. This makes it difficult for other researchers to repeat an experiment. Furthermore, glasshouse grade fertilisers are often not as pure as reagent grade salts, which may cause precipitates in the nutrient solution, or higher than expected levels of micronutrients. This is avoided when nutrient solutions are made from reagent grade salts. A well known solution is Hoagland’s solution (Hoagland, 1933), or published variants thereof. Full strength of these nutrient solutions provide very high concentrations of nutrients. Especially for wild plant species, it is recommended to reduce the concentration by a factor of 2–10 and rather fertigate, or – for hydroponics – replace the solution more frequently. Also in this case it should be kept in mind that a plant’s demand increases with size, such that greater amounts should be distributed towards the end of the growth period, at least if the aim is to keep the plant’s nutrient status more or less constant.

Solid fertilisers may be preferred over nutrient solutions in certain cases in which, for example, a more buffered/slow release of nutrients is required, or where it is necessary to mimic agronomic practices. Common mistakes when using these products involve the computation of the amount, as these fertilisers typically report N in %w/w of elemental N but, for historical reasons that date back over 200 years, they report P and K on the basis of the equivalent molecular mass of P_2_O_5_ and K_2_O, even though these elements are not present in that form (Lambers & Barrow, 2020). Mistakes are easily made, and it is therefore useful to be familiar with the symptoms of plant nutrient disorders to detect and correct them.

#### g. Increasing the representativeness of an experiment

Although the aim of the researcher is often to solve a general question, the results of a specific experiment are frequently context dependent, and therefore nonrepresentative. One option to improve the generality of controlled experiments may be to include some genetic variability within the species grown (e.g. Milcu *et al*., 2018). This could be done by randomly selecting seeds for example from different locations and/or genotypic background. Seeds of different sources could also be mixed when several individuals are grown together in the same pot.

Introducing environmental variability in a controlled way may also increase the generality of experimental outcomes (S. H. Richter *et al*., 2011). This could be done by introducing a block design, for which the blocks are on purpose treated slightly differently. For example, soils from different origins, different pot volumes or different types of lamps could be used.

#### h. Monitoring environmental conditions

Even in cases in which a specific environmental factor is not the focus of the experiment, it is still key to report the environmental conditions prevailing during such an experiment, for the simple reason that plant performance and plant traits are so dependent on them. Daily amount of light (daily light integral) and average day and night air temperature are strong determinants of growth, and so are soil substrate water and nutrient availability and pH. These variables should be preferably reported in any scientific paper. However, ‘envirotyping’ of the environment could go much further. A full checklist of relevant environmental variables is given in Poorter *et al*. (2012b). With respect to soils, additional variables such as root substrate temperature and bulk density are highly pertinent variables which are easily measured. Additional characterisations could include soil cation‐exchange capacity and total carbon and nutrient concentrations and a water retention curve. Given the rapid development of cheap sensors and controllers, many of these variables can now be characterised for each specific experiment, often at high time resolution (Srbinovska *et al*., 2015).

It could be considered to include some additional pots with plants in the experiment, which can be used to more specifically monitor environmental conditions, or to inspect root development during the experiment.

#### i. Further considerations

There is a wealth of other general measures that the experimenter could make to improve the growth of plants, avoid biases in the data, and increase the relevance of treatments applied under controlled environments for inferences for field performance. For this we refer the reader to, for example Langhans & Tibbits (1997), Poorter *et al*. (2012b), Wilkinson *et al*. (2014), Both *et al*. (2015), Poorter *et al*. (2016) and Schuman & Baldwin (2018).

### 3. Field observations, experiments and sampling

Conducting field observations and experiments is critical for understanding plant and ecosystem functioning in the complex, heterogeneous conditions typical of the natural world, and the realism of *in situ* measurements is an important advantage for studying a range of research questions. Similar to more controlled experiments there are a wide range of approaches to study roots *in situ*, and there are advantages and disadvantages associated with each methodology (summarised in Table 2). Root sampling and measurement are always labour intensive, it is therefore important to choose the methods most appropriate to the research questions; below, we present a selection of methods commonly used for root sampling and root observation. For each method, we discussed the advantages and drawbacks, sampling location and the number of replicates recommended.

**Table 2:** Advantages and disadvantages of common methods used to observe the below‐ground environment or assess below‐ground dynamics (e.g. patterns of root production and standing root biomass).

Technique	Advantages	Disadvantages	Best use	Relevant citation
Soil cores	Easy and cheap to collect. Can be easily scaled across plots as needed	Time consuming. Can cause substantial disturbance in small plots or over longer studies	Spatial or temporal distribution of root biomass in sites where repeated destructive samplings are acceptable	Jackson *et al.* (2009); Brunner *et al.* (2013)
In‐growth cores	Easy and cheap to install. Can be easily scaled across plots as needed	Can disturb and illicit wounding responses in perennial species that maintain root biomass throughout the year	Estimate root production in some herbaceous systems without large standing root biomass through dormant season	See discussion in Sun *et al.* (2016)
Soil trench profile	Possibility to analyse root distribution in relation to soil spatial heterogeneity	Cause disturbance in plots, uneasy to replicate, require calibration with root extraction method to estimate root length or mass density	Map of vertical and horizontal root distribution in 2D dimension	Van Noordwijk *et al.* (2000)
Rhizobox	Relatively inexpensive, easy to replicate, moderately easy to install and use. Large viewing and sampling area	Can be difficult to access deep roots without substantial changes to basic design	Repeated access to easily sample the upper *c*. 30 cm of soil. Good for most terrestrial systems	Zadworny & Eissenstat (2011); Meier *et al.* (2015)
Surficial root window	Inexpensive, fast and easy to install, easy to use	Does not access roots beneath surface horizon, must be installed carefully and at the right time to minimise artefacts	Repeated imaging and sampling of shallow‐rooted herbs and surface roots (e.g. some tropical forests)	Sun *et al.* (2016)
Rhizotron	Very large viewing and sampling area. Can be constructed to observe greater depths (> 1 m)	Can be expensive and labour intensive making true replication (i.e. in different plots or sites) cost prohibitive	Studies where intensive, long term, repeated observations of the soil system in a single site/plot are required	Pregitzer *et al.* (1993); Maeght *et al.* (2013); Mao *et al.* (2013)
Minirhizotron camera systems	Easy to replicate. Allows repeated imaging of soil environment without disturbance once installed	Expensive, not possible to physically sample roots without major disturbance. Limited viewing area	Repeated imaging of soil environment for study of long‐term absorptive roots and potentially fungal dynamics	Pritchard *et al.* (2008); McCormack *et al.* (2012)
Ground‐penetrating radar	Nondestructive. Can be used to spatially map coarse‐root architecture *in situ* across large soil volumes	Expensive and often requires substantial calibration. Cannot resolve smaller roots (< 5 mm diameter)	Repeated observations of coarse roots in relatively homogenous, sandy soil	Stover *et al.* (2007)

It is crucial for studies to select their sampling methods appropriately to ensure sufficient, and meaningful replication to be statistically robust. In this respect, one should carefully consider the use of both biological and technical replicates, as they are key instruments to assess and isolate sources of variation in measurements and limit the effect of spurious variation on hypothesis testing and parameter estimation (Blainey *et al.*, 2014). ‘Biological’ replicates are parallel measurements of biologically distinct samples that capture random biological variation, which may itself be a subject of study or a noise source, whereas ‘technical’ replicates are repeated measurements of the same sample that represent independent measures of the random noise associated with protocols or equipment. While the need for ‘biological’ vs ‘technical’ replicates might vary among studies or systems, it can be advocated that the variation in biological units (e.g. among plant species) will be generally better captured by biological replication (e.g. measurements taken from a range of plant individuals) than by technical replicate (e.g. replication of measurements on the same sample of the same plant) (Blainey *et al.*, 2014). Nonetheless, depending on the research question, several levels of biological replication might need to be considered (e.g. measurements taken from a range of plant individuals and from several roots on each plant individual).

#### a. Overview of sampling methods

##### Excavation of the whole root system (architecture‐guided sampling)

Excavation of the whole root system is a useful method for exploring root architectural, morphological and chemical traits, as well as biomass allocation to roots and other organs. With excavation methods, the root system of an individual plant (or a plant ‘ramet’ in case of clonal plants; see section **VIII. Horizontal plant mobility**) is exposed by removing the surrounding soil manually or with the help of a high‐pressure air or water system (Böhm, 1979).

There are several different methods available for excavation, including trenching in both vertical and horizontal planes. On a vertical plane, a trench is dug close to the centre of the plant. Its depth should extend 20–30 cm below the deepest roots, and a 1‐m width is recommended to facilitate excavation. Following this, soil is carefully removed from the plant side of the trench, starting with the surface soil, and gradually working downward (Böhm, 1979). For herbaceous species with narrow‐diameter root systems, fine picks, sharpened needles and brushes are used to remove the soil. For woody species, use of larger tools such as pickaxes or metal forks may be necessary. The use of a supersonic air stream that blows away the soil particles works quickly on light soils (hundreds of times faster than hand excavation), but more slowly on heavy soils, which are less porous (Nadezhdina & Čermák, 2003). For woody species with horizontally growing root systems, excavations in a horizontal plane using supersonic air stream are preferred (Böhm, 1979). To study their coarse root biomass and architecture, woody species can be uprooted by pulling the stump with a trestle, a mini‐shovel or a lumbering crane (Danjon *et al*., 1999; Danjon & Reubens, 2008).

*Advantages and disadvantages*. The excavation method provides a picture of the coarse‐root system architecture as it exists naturally. It is considered as the best method to accurately estimate coarse‐root biomass in individual trees and shrubs (Addo‐Danso *et al*., 2016). However, the excavation method requires a large amount of physical labour and is very time consuming. More than one specimen is needed to be sure that the root‐system information obtained is representative of the plant species growing on the given site. Because complete recovery of the entire root system is extremely difficult, especially in deep soil (Maeght *et al*., 2013), the excavation method may result in sampling errors as roots become broken or lost during excavation. For this reason, the excavation method is more suitable for studies involving trees and shrubs compared with studies for herbaceous species, as woody roots are generally stronger and more resistant to breaking compared with fibrous roots.

##### Monolith excavation

**Fig. 7:**
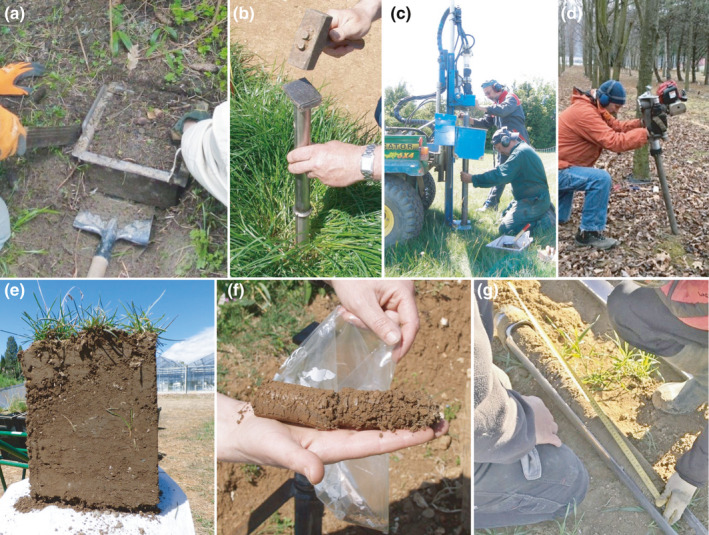
Soil sampling techniques using (a) a metallic frame (0.25 × 0.25 × 0.15 m); (b) a manual core or (c, d) mechanised augers. (e) Monolith excavation provides a large volume of soil, which increases the accuracy of root biomass estimates; (f) manual coring provides small cores usually sampled in successive steps of 10–20 cm depth with a 5–10 cm diameter core; (g) mechanised augers allow deep coring in harsh soils. Image courtesy of C. Roumet (a, b, c, f), M. Zhun (e), F. Khalfallah (g).

The monolith method requires cutting of soil monoliths directly from the soil surface or at different soil depths (Fig. 7a,e). When monoliths are excavated at different depths, trenches are dug before starting the sampling procedure. The size of the monolith typically ranges from 5 × 5 cm to 40 × 40 cm at the soil surface and down to 20 cm depth. In mineral soils, monoliths are taken with shovels, or excavated with thick metallic frames driven into soil with a heavy hammer while, in organic soils, a breadknife can be used to cut a soil block. If metallic frames are used, it is recommended to place a piece of hard wood on the top of the frame to protect the frame during the hammering. When the frame is sunk into the soil, the monolith is lifted out using a shovel underneath the monolith. Soil and roots are then separated by washing or by hand sorting (see section **VII. Root washing, sorting and storage**). This method is frequently used to determine root traits of herbaceous species in the upper soil layers. The ‘shovelomic approach’ is a modified monolith excavation to phenotype root crown architecture of crop species (Trachsel *et al*., 2011).

*Where to sample.* When the objective is to determine the root biomass and traits, the monolith is centred on one isolated individual for herbaceous species or on an isolated coarse root for woody species. For crop species, we recommend a 40 × 40 cm monolith centred on the stem. When the objective is to determine the root traits at the community level, monoliths (generally 25 × 25 × 20 cm deep) are excavated at a place representative of the plant community.

*Number of replications.* In most studies, three to six monoliths are excavated; the coefficients of variation are however often very high due to soil heterogeneity and plant patchiness, which causes wide variation in root mass and distribution. Increasing the number of replicates increases the precision of fine‐root biomass estimates but also requires substantial time for root harvesting and sorting and could considerably damage the field area. In a *Eucalyptus* plantation, Levillain *et al*. (2011) demonstrated that 147 monoliths (25 × 25 × 10 cm deep) were required to achieve 10% sampling precision for fine‐root biomass; this number fell to 14 monoliths for a 30% precision.

*Advantages and disadvantages.* Monoliths provide large soil sample volumes, which increase the accuracy of root biomass estimates (Taylor *et al*., 2013) and reduce the number of replicates needed to secure good estimates of root biomass (Levillain *et al*., 2011). Monoliths are also suited for harvesting an intact shallow root system for analyses of root traits, including morphology and some types of architectural traits. The monolith excavation is relatively easy, however, because monoliths are large, root washing and sorting is time consuming and laborious, especially when monoliths are collected in natural communities where the root density can be very high. Levillain *et al*. (2011) reported that for a monolith (25 × 25 × 10 cm deep), root washing and sorting can take up to 31 person‐days, accounting for 55% of the time spent in the field. In addition, it is difficult to remove all the mineral and organic matter particles from such large samples, and root loss can occur during washing (Oliveira *et al*., 2000; Pierret *et al*., 2005).

##### Soil core sampling

The method is based on sampling a cylindrical volume of undisturbed soil with a soil auger (Fig. 7), where the roots are then separated from the soil core by washing (see section **VII. Root washing, sorting and storage** **)**. The soil core method is commonly used to determine fine‐root biomass density and rooting depth distribution, as well as morphological and chemical traits of fine roots. However, the relatively small diameter of the soil auger is inappropriate for capturing root system architecture and does not adequately sample coarse roots.

Augers typically range from 10 to 100 cm in length, with an inside diameter between 2 and 10 cm. The diameter of the auger should be adjusted to the mean root diameter and root length density in the system and is recommended to be larger in forests (diameter 4–10 cm) compared with herbaceous communities or grasslands (2–5 cm). Entire soil cores can be extracted at once over the complete soil profile in soil of light to medium density, but this would create too much friction in dense soils such as clay soils and compacted soils. Extracting soil cores at once is particularly advisable with dry sandy soils in arid environments, because they present a larger risk of losing soil from the auger, and a small auger diameter is further recommended to avoid soil loss during core extraction (Oliveira *et al*., 2000). If the soil is more compact, sequential coring of different soil layers is preferred to avoid strong compression of the top soil layers in the core due to friction with the core walls. The disadvantage of sequential coring is the higher risk of losing soil material from the core and of soil falling into the hole during coring. An auger diameter less than 4 cm increases the risk of frictional resistance and soil compaction (Schuurmann & Goedewaagen, 1965). The decision for the one over the other method needs some experience and trial and error. Measurements of soil water content and bulk density (generally accounting for rock volume) are needed to extrapolate root biomass to a per unit soil mass, but may not be necessary for soil volume (although accounting for rock volume remains necessary) and ground area metrics.

There are two main types of hand augers. The first, a twist‐auger, is based on twisting a light auger by turning and pushing downward at the same time. It consists of a cylindrical tube (e.g. 15 cm long, 7 cm diameter); a T‐handle at the top of the auger shaft makes it possible to rotate the auger while driving it into the soil and to pull out it again. Twist‐augers often twist roots out of the surrounding soil, which can unfortunately result in overestimation of root biomass per volume of soil. The second type is a heavy hand auger, usually a stainless‐steel cylinder, provided with a striking head, which is driven into the soil with a hammer, slide hammer, or fence post driver (Fig. 7b,f). The steel corer may be lined with a plastic tube to help with soil core retrieval and transport, although this may not work well in all situations. The edges of the cylinder need to be bevelled or well sharpened to cleanly cut the roots rather than pulling them from surrounding soil. Keep in mind that a sharpened thin edge will be damaged more easily by stones than a thicker blunt edge. As sharpening takes a metal file or a smithy, you might want to take extra augers (e.g. up to 10) to the field for larger sampling campaigns in rocky soil conditions. In particularly rocky soils, a powered auger with a diamond‐tipped bit may be used to retrieve cores (Johnson *et al*., 2008).

To sample soil cores by depth increment, intact cores can be placed on a flat surface and cut to length. If extraction of intact cores is not possible, repeated sampling at different depth intervals can be accomplished by replacing the auger in the same borehole after sampling the upper portion of the core. To remove the auger from the shallow soil, a rod is used to turn and lift the corer out of the soil; in deeper soil, a lever or chain may be needed to pull out the auger. Depending on the soil characteristics, coring can be quite difficult. While augers work well in mineral soils, a modified hole saw connected to a battery‐operated drill can be used to slice cleanly through organic soils and retrieve an intact core that is not possible with a normal auger. Alternatively, a sharp breadknife can be used to sample square areas of peat.

The use of mechanised augers attached to a tractor or other large piece of machinery (Fig. 7c,g; see Böhm, 1979; Prior *et al*., 2004) may reduce the time and labour required for taking soil cores, and may be needed in very hard soils or if core samples are to be taken below 1 m soil depth. However, they are expensive and may disturb the surrounding plant communities. Hand‐held, gas‐powered post pounders are less destructive and may also be used in cases in which machine assistance is desired (Fig. 7d). But hand augers will suffice in most cases.

*Where to sample.* If the aim of the study is to measure the traits of a given species within a plant community, coring is done above or next to the plant of interest. The roots attached to the shoots are then separated from the roots of the neighbouring species. For row crops or experiments where the density of plants is fixed, sampling is stratified within and between rows or plants, according to row spacing (van Noorwijk *et al*., 1985). If the aim is to obtain a community‐level trait measurement, as in grasslands or other systems where plants are heterogeneously distributed due to environmental conditions or microtopography, a complete randomised design may be followed. Another option is to follow the protocol used for botanical *relevés* or soil sampling; that is collect soil cores along a line transect or at different places in a quadrat.

Furthermore, it needs to be considered that fine roots can be deeply rooted, and that it is generally not appropriate to solely sample the top few centimetres of the soil. The total depth and depth increments used should be considered based on the question of interest, but also the horizonation of the soil (e.g. roots growing in litter or organic soil layers may have very different traits from those growing in mineral soils or deeper soil horizons, Onipchenko *et al*., 2009; Fort *et al*., 2017). Also, the position of the core regarding the base of the plant targeted can strongly influence the fraction of roots sampled (Bouillet *et al*., 2002; Leuschner *et al*., 2004; Danjon *et al*., 2013a; Lee, 2018).

*Number of replications*. The soil core method requires many samples as the auger volume is generally small. The number of replicate cores per experimental unit or per species typically varies from three to 10. While this generally falls short of an ideal estimation of root biomass (for example, to estimate fine‐root biomass in a *Eucalyptus* plantation, Levillain *et al*. (2011) showed that it is necessary to sample 312 auger cores to achieve 10% precision or 35 cores for a 30% precision), it is often not feasible to take a larger number of core samples. To contend with high variability in natural communities, a composite sampling scheme can be followed by combining different (5–10) cores along a transect or within a quadrat and homogenising them to get a representative sample (Schroth & Kolbe, 1994). With this method, the replicates are the lines or the quadrats.

*Advantages and drawbacks.* The coring method is the cheapest and simplest way to quantify fine‐root biomass (Levillain *et al*., 2011; Addo‐Danso *et al*., 2016). It can also be used appropriately to capture spatial and temporal heterogeneity simply by increasing the number of replicates or coring dates. The small diameter of most cores can, however, be a disadvantage in heterogeneous plant communities and in depths where rooting density is low. Increasing replicate cores can compensate for such issues. Another drawback to coring is soil compaction resulting from the sampling process. Park *et al*. (2007) showed that soil compaction may generally result in a 10% overestimation of root mass density. Core‐sampling methods are not well adapted when the density of stones or very thick roots is high, and the volume of stones within a soil core should be accounted for in estimating soil volume and bulk density. When soil is very dry, soil coring can be challenging, it is therefore recommended to rewet the soil 1 d before coring, whenever possible.

##### In‐growth cores

**Fig. 8:**
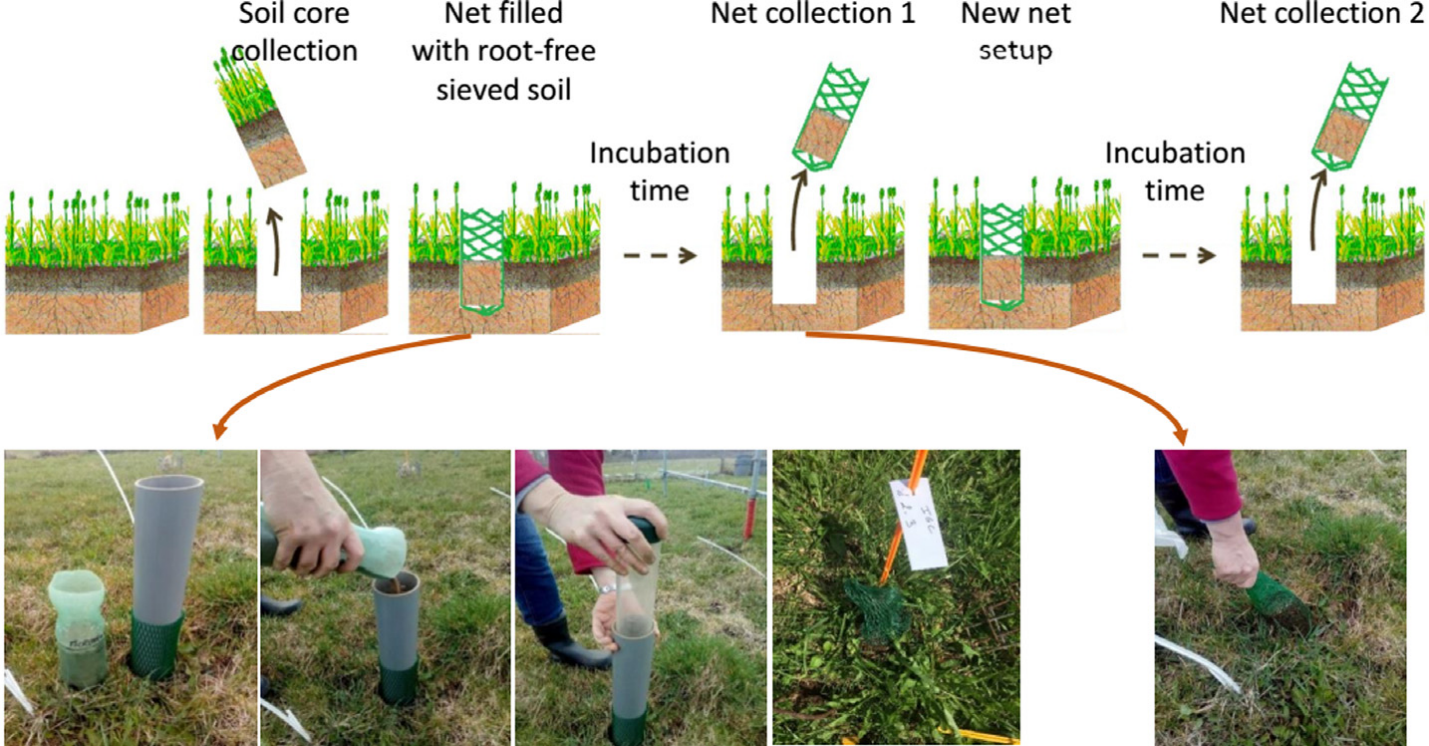
Soil in‐growth core method. A soil core is taken and used for initial root biomass estimation. The core is then filled with similar sieved soil, free of roots, placed into a mesh cylinder. After few weeks of incubation, the mesh bag is removed to estimate the fine‐root biomass production rate.

The in‐growth core method (Fig. 8) is based on providing a volume of root‐free soil to capture the growth (i.e. ‘in‐growth’) of fine roots from the surrounding soil (e.g. Lund *et al*., 1970; Flower‐Ellis & Persson, 1980; Vogt *et al*., 1998). Roots sampled using in‐growth methodology provide an estimate of fine‐root biomass production rate; ingrown roots can be used for analyses of root chemical composition and root morphology. The in‐growth core methodology can be approached in some different ways, depending on the system of interest, the dominant plant species and the surrounding edaphic and environmental characteristics. An initial soil core is taken and can be used for estimates of root biomass as described above. At this time, the moisture content and bulk density of the soil should also be quantified so that the in‐growth core can be targeted to the same amount of dry soil (i.e. the same bulk density) as surrounding soil. The soil removed from the core can be sieved to remove any living roots from the soil (additional close inspection of the soil using ×20 magnification jewellers’ glasses or a dissecting microscope for lingering living roots is often needed, especially in soils dominated by plants with a narrow root diameter or a large amount of root biomass). Alternatively, a large volume of soil from a similar soil depth (as many soil parameters, including carbon and nutrient content, soil texture, and microbial community composition change with soil depth) may be taken from elsewhere at the site, allowed to air dry and then sieved. When working in organic soils that cannot be sieved, it can be nearly impossible to remove (‘untangle’) all of the living roots from a large quantity of organic soil matrix in a timely fashion, especially in nutrient‐poor systems dominated by very fine‐rooted ericaceous shrubs. In such cases, we recommend purchasing commercially harvested peat ideally from a nearby peatland ecosystem (e.g. Bhuiyan *et al*., 2017), where the living roots have been allowed to decay and the peat has been milled to a homogenous texture (note that dry peat can require autoclaving with distilled water to rewet). The soil should be preferably prepared several months before use because of the risk of mineralisation inducing the presence of high concentration of minerals (Hassink, 1994; Chen *et al*., 2016).

Most commonly, the processed soil is filled into a mesh cylinder placed inside the hole. The sieved or processed soil should be packed into the cylinder in an amount that targets the native bulk density of the surrounding soil; this can sometimes require additional soil, as rocks and roots are lost during the sieving process. The mesh cylinder can be sown or stapled by hand or extruded plastic mesh can be used (https://www.industrialnetting.com/cores.html). A mesh bottom can be attached to the cylinder using a zip tie. The desired size of the openings in the mesh cylinder is dependent on the soil type, but should allow water and microfaunal access without allowing loss of soil and generally ranges between 1 and 6 mm (Lund *et al*., 1970; Vogt *et al*., 1998; Sullivan *et al*., 2007). The mesh cylinder should extend *c*. 10 cm above the soil surface to aid in retrieval. However, in case of grazed vegetation, the net should be protected from cattle using a grid that maintains the net at ground level.

To remove and replace the in‐growth core with minimal disturbance, use a very sharp knife to cut around the outside of the mesh cylinder to remove the in‐growth core from the soil; a dull knife will pull roots from the surrounding soil and inflate estimates of root production. Extra‐long bread knives are now commercially available. Pull the in‐growth core from the soil using the extra 10 cm of protruding mesh and replace with a freshly made core. Once the in‐growth core has been removed from the soil, roots protruding from the mesh should be clipped to the mesh surface so that the final, dry biomass of roots within the core can be reported per unit soil volume (or ground area). Fine roots may be sorted to species (see section **VII. Root washing, sorting and storage**), and may further be scanned to determine root length, diameter and other morphological traits. If depth increments are desired, in‐growth cores (mesh and all) may be frozen at −20°C and cut into 10‐cm depth increments (or finer) using a bandsaw.

In some systems, especially those that are particularly dry, the in‐growth mesh does not work well, as it can become ‘locked’ in the dry soil and tear upon retrieval. In this case, we suggest that a smaller diameter core is taken from within the volume of root‐free soil, avoiding the mesh altogether. Sieved or processed soil is placed directly into the original hole, taking care to pack the soil to the same bulk density as the original core. To ensure that the in‐growth soil is able to be precisely sampled upon retrieval, we recommend marking the circumference of the initial core with an aluminium or plastic collar before the addition of processed soil (e.g. Iversen & Norby, 2008). This helps to ensure that the original core location can be identified and that the smaller diameter core can be taken inside the anchored collar.

*Where to sample.* See the above discussion on where to sample soil cores for root biomass estimates; similar considerations apply with root in‐growth cores. Also consider that some species tend to have roots that grow laterally (e.g. trees, shrubs), while others grow straight down (e.g. most graminoids; Iversen *et al*., 2012); in‐growth cores may therefore need to be installed at an angle in soils dominated by graminoids, directly adjacent to the plant to better capture species with limited lateral root production (e.g. Sullivan *et al*., 2007).

*Number of replications*. See the above discussion on how many soil cores must be taken to assess root biomass; similar considerations apply with root in‐growth cores. Also consider that the timing of in‐growth core removal should vary across ecosystems depending on the rate of root growth (faster removal times for roots in the tropics – for example, every 3 months – and slower for roots in boreal systems, for example, incubation times of a year or more; Makkonen & Helmisaari, 1999; Metcalfe *et al*., 2007a). Ultimately, a balance between root growth and development and root mortality must be struck. In‐growth cores are essentially a ‘net’ estimate of root production, as some roots may be produced, die and decay during the period of incubation.

*Advantages and drawbacks.* In‐growth cores are relatively inexpensive to construct and may provide some of the only estimates of root production in ecosystems in which minirhizotrons are logistically or financially impossible to use (Girardin *et al*., 2013). However, there are some scientists who recommend never using root in‐growth cores for several reasons. First, the soil disturbance and root pruning induced by the method can stimulate root growth, leading to unnaturally high estimates of root production in the in‐growth cores. Second, different species have different responses to wounding, which may partially relate to their diameter (i.e. smaller diameter species tend to respond strongly to wounding; Eissenstat *et al*., 2015). Therefore, in a mixed‐species ecosystem, differences in root in‐growth into cores along environmental gradients may be due to the presence of species with varying responses to wounding. This is particularly troubling in heterogeneous ecosystems, but in‐growth cores can provide a reasonable estimate of fine‐root growth responses within experimental manipulations of similar species composition. Furthermore, we caution that some species produce pioneer roots in response to disturbance; these roots should be treated differently than the fibrous roots in the cores, as pioneer roots generally become transport roots with a differing anatomy, morphology, and chemistry (Zadworny & Eissenstat, 2011). An advantage of in‐growth cores is that newly produced fine‐root biomass is extracted directly from the cores, and therefore this destructive approach necessitates less scaling than the observational approaches described below. Furthermore, in‐growth cores provide access to newly grown fine roots for chemical and morphological analyses, although ingrown roots tend to have tissue that is less dense than roots in surrounding soils, perhaps because they are younger and have had less time for secondary growth, or perhaps because the soil matrix has been sieved and reconstituted, providing less resistance to growth.

#### b. Overview of root observation methods

##### Soil trench profile

**Fig. 9:**
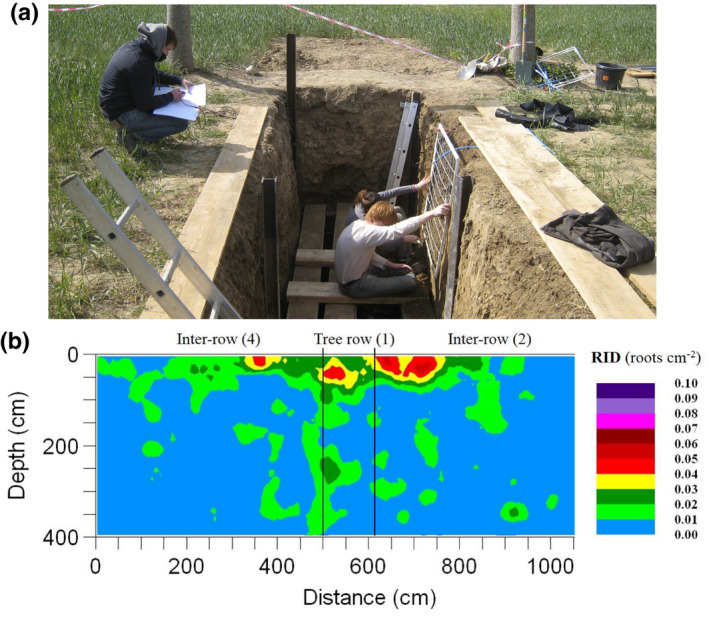
Trench profile method: (a) root mapping using a grid fixed on the wall of a 4‐m agroforestry trench to determine (b) the root intersection densities at different soil depths (RID, cm^−2^). Image courtesy of R. Cardinael and C. Jourdan.

The trench profile method consists of mapping or counting roots on a vertical or horizontal intersecting plane (van Noordwijk *et al*., 2000; Fig. 9). A trench is dug and visible roots are mapped on the walls of the trench, using a grid or an acetate sheet, to determine the root intersection density (RID, defined as the number of root intersections with the plane of observation counted on a given soil surface, cm^−2^). This method provides information on the root horizontal and vertical distribution and on the maximum rooting depth. A variant of this method is to use available soil profile walls created by road cuts or landslides, instead of digging trenches (Canadell *et al*., 1999; Maeght *et al*., 2013).

The size of the trench should capture most of the extent of the root system under investigation; it therefore depends on the plants studied and the aim of the research (see below ‘where to sample’). A minimum of 1 m width is, however, recommended to facilitate access by the observer. The face of the trench is sheared with a flat shovel after the trench has been dug down to the desired depth. A square grid system, consisting of 5 × 5 cm squares for herbaceous species or 10 × 10 cm squares for woody species, that can be constructed with nails and string is fixed against the profile wall and serves as a guide to record the number of exposed roots. The position of the roots can also be mapped on a plastic sheet. To facilitate observations, it may be helpful to add water 1 or 2 d before sampling or schedule sampling a few days after rain because moist, but not wet, soil is easier to work in. Having a second person to mist the grid wall or shearing the wall in sections may be necessary, especially in arid or semiarid environments where the trench wall may dry quickly. Direct sunlight, that dries roots quickly, can be avoided by working under a tent. A tally counter is helpful for counting the number of root intersections with the vertical plane in each grid square. The mean RID is calculated for each square in the grid by dividing the number of roots counted in each square by the surface area. Root distribution can then be graphed against grid coordinates (Fig. 9b). Conversion of an observed RID distribution to the root length density (RLD) distribution requires a calibration; this is feasible by sampling soil blocks from the trench wall and by taking into account the anisotropy of the root system (see van Noordwijk *et al*., 2000; Vansteenkiste *et al*., 2014; Cardinael *et al*., 2015 for more details).

*Where to sample.* The position of the trench depends on the plants under study. In uneven plant communities, as grasslands, trenches are dug at a position representative of the plant community. In cropping systems, where plants are grown in rows, trenches are dug perpendicular to rows and near the base of a plant, a length equal to the crop row and a 1 m depth is recommended to capture the majority of the root system. For trees, trenches are dug 1–5 m or more from the trunk, in tangential or radial position (Böhm, 1979), or between two trees for tree plantations (Cardinael *et al*., 2015). Typical trench depth range from 1 m to 10 m (Laclau *et al*., 2013; Maeght *et al*., 2013; Cardinael *et al*., 2015). Deep trenches are crucial to better understand the role of deep roots in plant and ecosystem functioning, however the instability of walls, which depends on soil type and moisture, is a major technical obstacle limiting the use of deep trench.

*Number of replications*. The trench profile method is rather destructive and time consuming, so that it is impossible to dig more than three trenches per plot (Laclau *et al*., 2013; Maeght *et al*., 2013). However, roots can be mapped on replicate profiles within the same trench. This is not possible in radial trenches where each root map has its own distance from the tree.

*Advantages and drawbacks.* Soil trench profile provides crucial information on root distribution in relation to soil spatial heterogeneity. They can also be used to take soil and root samples at different depths or to install probes or minirhizotrons (Maeght *et al*., 2013). The major drawback is that this method is destructive, laborious and time consuming, limiting the number of replications. It is often difficult to distinguish roots especially fine roots of herbaceous species. It provides only a two‐dimensional root distribution; predictions of RLD from RID requires laborious calibrations based on the sampling of soil monoliths from the trench wall.

##### Rhizoboxes

**Fig. 10:**
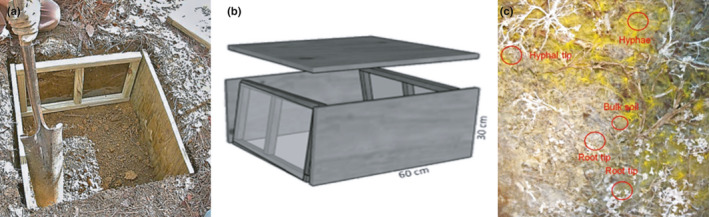
Rhizobox installation and observation. (a) Installation in the field, note that when installed, the windows may be tilted inward by *c*. 5–10° to facilitate better contact with added sieved soil; (b) diagram of rhizobox construction, an acetate sheet is fixed to the window frame; (c) observations of roots together with fungal hyphae. Images courtesy of J. Pippen (a), M. L. McCormack (b) and I. C. Meier (c).

Rhizoboxes, sometimes referred to as root windows or root boxes (Shaver & Billings, 1975; Böhm, 1979), allow the targeting of specific regions within the soil with high precision (Bagniewska‐Zadworna *et al*., 2014), facilitating the study of root phenology (Bai *et al*., 2010) and the effects of localised fertilisation on root growth (Adams & Eissenstat, 2015; Ceccon *et al*., 2016). They also enable access to roots of known age, rhizosphere soil, extensive formations of rhizomorphic fungi, and patches of diffuse hyphal growth and adhering soil (Grierson & Comerford, 2000; Dong *et al*., 2007; Meier *et al*., 2015). In the field, rhizoboxes may be built into the side of a hill or trench (one‐sided), or built within a community of interest (two‐sided or more) (Fig. 10). Windows may be shallow (i.e. a glass pane at the soil surface covered with a bag of soil) to target surficial roots; this may be especially useful for study of herbaceous species (K. Sun *et al*., 2016) and of surface roots common in many wet tropical forests. Deeper boxes, to *c*. 30 cm, tend to capture the majority of fine‐root biomass in most systems but root boxes can also be constructed to 1 m depth (or deeper) as required (e.g. Maeght *et al*., 2013; Mao *et al*., 2013). The design of a rhizobox is highly customisable, but the box must establish good soil contact (slightly angling the window from the soil can facilitate better contact even after minor soil settling) and minimise light penetration and thermal fluctuations, for example by insulating the windows with rigid foam board; a sturdy lid should be secured over rhizoboxes to avoid injury and to minimise animal access. We recommend establishing a flush and even soil face with the observation window to maximise root appearance at the window and minimise soil artefacts. If needed, sieved soil can be added between the window and soil face to maximise window contact, where added soil is tamped with a rod to achieve the appropriate bulk density and minimise air pockets.

Rhizobox observation windows can be constructed of glass panes, plastic sheeting, or plastic film (e.g. acetate). Plastic film offers the advantage of direct access to observed roots and/or fungi by cutting through the film, however care must be taken not to tear the film during installation, especially if back filling is necessary. Typical thickness used for plastic film is 0.005–0.01 mm, with thicker films providing more support and being less susceptible to accidental tearing while thinner films may be more easily cut when harvesting roots. Following window installation, foam or other insulation should be cut to fit over the window to minimise temperature fluctuations at the soil–window interface and therefore minimise potential chamber effects.

Roots observed on windows may be traced to track growth over time. More recently, cameras and scanners have been used (Bai *et al*., 2010; K. Sun *et al*., 2016). Each has its particular advantage, which may depend at least partly on the type and size of rhizotron or root box installation (see discussions in Mohamed *et al*., 2017). If researchers cut the acetate windows to harvest roots (i.e. roots of known age), clear tape can be used to repair access cuts in the plastic film. Ideally, rhizoboxes should be designed with easily removable observation windows so that the plastic film can be replaced after extensive root/fungal sampling or tracing. When tracing on observation windows, permanent paint pens should be used (e.g. Marvy DecoColor Paint Pen), as regular permanent markers often will not withstand repeated wiping of window condensation before each tracing event.

*Where to sample.* Areas prone to flooding should be avoided, as rhizoboxes in poorly drained soil may occasionally fill with standing water. Avoid termites and other wood‐eating insects and use pressure‐treated lumber if needed. Care should be taken when working in rhizoboxes as insects, rodents and reptiles may be encountered. When working in woody ecosystems, boxes should not be installed immediately adjacent to woody stems. Instead, installing rhizoboxes at least one, or a few metres from the trunk reduces the likelihood of damaging the tree and major coarse roots during installation and increases the likelihood for good fine‐root growth to occur on the window face as there is more space for incremental lateral branching from coarse roots at the base to more lateral and fibrous roots further out.

*Number of replications.* Rhizoboxes are laborious to install and can result in substantial disturbance to study areas. However, the materials required are somewhat inexpensive and, once installed, rhizoboxes can be used for several years. Although multiple roots and fungi can be observed and sampled from a single rhizobox, significant variation among boxes within a study system is common. As such, care should be taken when determining how many boxes are necessary to address a particular research question while balancing cost and disturbance.

*Advantages and drawbacks.* Rhizoboxes allow for direct observation and access to roots of known age and soil location, As discussed above in the section on in‐growth cores, there may be species‐specific artefacts resulting from root wounding, and pioneer roots are particularly prevalent in root windows, especially in the initial period after installation. Also, plant species often exhibit contrasting root growth angles so that roots reaching the rhizobox windows may represent a variable fraction of the root system, depending on the species. Rhizoboxes should ideally be installed a full growing season before initial use to allow for the soil environment to normalise at the window face and limit sampling of abnormal root growth elicited from wounding at installation.

##### Minirhizotrons

Minirhizotrons have been used since the early 1900s to estimate root length production (Bates, 1937) and their use and utility have been reviewed in Hendrick & Pregitzer (1996), M. G. Johnson *et al*. (2001), Iversen *et al*. (2012; focusing on wetlands) and Rewald & Eprath (2013). In addition to quantifying patterns in the birth, growth and death of individual roots, minirhizotron images allow for quantification of the diameter and length of distal roots. In general, a minirhizotron is a clear tube inserted into a premade hole in the soil and a minirhizotron camera system is used to capture images of roots at periodic intervals throughout the year from inside the tube. The general consensus is that tubes manufactured from acrylic plastic are the least likely to affect root characteristics (with glass as the control; Withington *et al*., 2003). However, care needs to be taken during shipping and installation, as acrylic tubes are breakable and easily scratched (longitudinal scratches can look very similar to roots).

For initial installation of minirhizotron tubes, an auger or steel corer of roughly the same diameter of the minirhizotron is used to make a hole in the soil, usually targeting a 45° angle to avoid water running down the surface of the tube and affecting root growth, and also to ensure the capture of roots that have either a lateral or vertical growth orientation. However, 30–60° or even vertical may be used in some cases. Depending on local soil conditions, an auger that is slightly smaller may be used to ensure good soil contact with the tube. However, if rocks are common in the soil, this may also result in scratching the tube surface during installation. Additionally, in clayey soils this may result in smearing the tube surface which obscures later viewing and imaging. Augers that are slightly larger than the tube diameter may make inserting the tube into the ground easier and minimise scratching. While in some cases adjacent soils may slightly swell to achieve good contact with the tube, this generally does not occur, and the use of larger holes often results in poor soil contact and limited visibility of roots on the tube surface. If a larger installation hole is needed to prevent scratching or smearing of the tube, M. G. Johnson *et al*. (2001) recommend a method for improving tube contact with the upper soil for minirhizotrons that image only the upper surface of the tube. Regardless of the installation tools and anticipated protocol, it is recommended that test holes are drilled or cored before the main installation date and the approximate fit is tested with a minirhizotron tube. This will allow adjustments to be made as needed (e.g. obtaining a smaller corer or grinding narrower auger bits as needed). There are several companies that provide minirhizotron cameras (Iversen *et al*., 2012), and each has a different tube size recommendation, so the installation will need to be tailored to the required tube size.

**Fig. 11:**
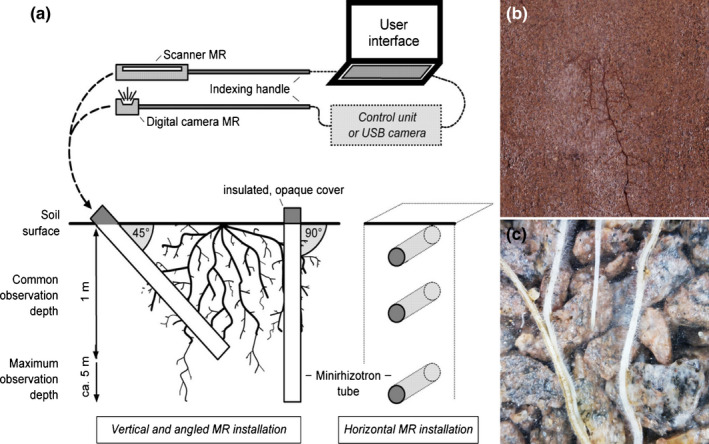
(a) Minirhizotron (MR) techniques with image acquisition devices (i.e. digital camera MR or scanner MR) and different options to install the MR tubes, that is angled or vertical from the soil surface or horizontally from trenches; (b) roots of *Fagus sylvatica* captured with a scanner MR system, CID 600; (c) roots of *Pisum sativum* taken using the Vienna Scientific MR camera MS‐190. Images courtesy of B. Rewald.

There are several important considerations for preparing the minirhizotron tubes for installation. Some commercially available tubes may be more than 1‐m long. This may be appropriate for some research questions, although if needed they can be cut to smaller lengths provided that each tube bottom is sealed with a waterproof cap. The tube, with water‐tight endcap in place, should be installed so that the amount of tube protruding from the soil is enough to accept an indexing handle associated with a minirhizotron camera system. In organic soils where the moss at the soil surface can grow up the surface of the tube each year, we recommend leaving enough tube protruding to accept this growth over the years without affecting the ability to attach a camera system. Before tube insertion, the upper portion of the tube that will remain outside of the ground is taped with two layers of electrical tape or pipe wrap down to 1 cm into the soil to exclude light. Following installation, the top of the tube is also capped (e.g. with a rubber stopper and aluminium can). The protruding portion of the tube should be shielded from penetrating radiation (which can affect root growth) and insulated from extreme temperatures. In addition to tape, or pipe insulation and caps to immediately enclose the top of the tube, some groups also cover the tube with a white PVC cap for added protection from light, temperature fluctuations and animals (Fig. 11). Once the tube has been installed, it often needs to be anchored into the soil to minimise any movement of the tube (Fig. 11). Movement may occur with frost heaving, high rain events (e.g. in saturated soils), or during the insertion of the camera as users accidently twist the tube. It is very important that each root image captures the same soil area so that the birth, growth and death of individual roots can be tracked. In mineral soils, a steel rebar driven into the soil at the protruding part of the tube at 45° angle can be used to anchor the tube. In organic soils, use a 3‐m angle iron driven into the peat and attached to the tube with a hose clamp and zip tie (Iversen *et al*., 2018).

Minirhizotron imaging ideally does not start immediately after installation of the tubes. It has been shown that a period of equilibration is needed after tube installation to allow for roots that have been cut or damaged to die and decompose, and also for new roots to grow and meet the tube surface (Iversen *et al*., 2008). Following installation, nutrient mineralisation in adjacent soil and severing and subsequent dieback of roots may increase fine‐root growth near the installation zone (M. G. Johnson *et al*., 2001). A waiting period of 1 yr or more may be necessary for the roots and soil to stabilise to some extent after such disturbance. A rule of thumb is that equilibration has occurred once fine‐root productivity and mortality are roughly equal.

Minirhizotron cameras can be purchased commercially, and are used to acquire images, either from the upper surface of the tube or from a nearly 360° view of the tube. These images are then used to trace individual root length and diameter over time, to estimate rates of root birth, growth and death. Several software options are available to assist in processing minirhizotron images including rootfly 2.0.2 (Clemson University, Clemson, SC; https://sourceforge.net/projects/rootfly/), RootSnap! (CID Bioscience), and rhizoTrak (Halle University, https://github.com/prbio‐hub/rhizoTrak; https://www.biorxiv.org/content/10.1101/547604v1). These may also be used to keep a record of changes in various qualities of individual roots such are root colour or root order. When possible, it may be useful to relate visual changes in roots to physiological changes to be able to interpret observations as root distribution of active roots (Comas *et al*., 2000). For example, some species have pronounced visual changes in pigmentation that can be related with vital staining (see section **XIII. 2. Percentage of viable root cells**) to declines in metabolic activity and death (Comas *et al*., 2000).

*When to sample.* Depending on the question of interest, minirhizotron images are often collected anywhere from weekly (Iversen *et al*., 2018) to monthly or longer (Pritchard *et al*., 2008; McCormack *et al*., 2012). Shorter time periods are needed to determine the phenology of root growth, while longer time periods are sufficient if root lifespan is the focus (see section **XVI. Root dynamics**). Some investigators may increase sampling frequency during highly active below‐ground growth periods (McCormack *et al*., 2015b). While acquiring minirhizotron images year‐round is ideal, freezing soil will often exert pressure on the outside of the minirhizotron tubes, making it difficult to insert the camera, and snow often makes it difficult to access the tube. Therefore, we know relatively little about root activity during the winter months (Blume‐Werry *et al*., 2015).

*Number of replications*. Minirhizotron tubes are the least expensive part of the method, and as many tubes as possible should be installed, following the recommendations in the section on root coring. This is particularly important given that minirhizotrons sample a relatively small volume of soil (Taylor *et al*., 2013). However, significant time is required to collect images in the field and then subsequently process images in the laboratory, which should also be considered when designing and installing long‐term plans for minirhizotron studies.

*Advantages and drawbacks.* Minirhizotrons are the best method for observing the timing (phenology) of root birth, growth and death, and provide a window into the rhizosphere environment that most other methodologies do not (Fig. 11). However, analysis of minirhizotron images is quite time consuming and often subjective. Furthermore, scaling the growth of individual roots from minirhizotron images to soil volume requires several assumptions (i.e. the image depth of field; Taylor *et al*., 2014), as does a conversion from length production to biomass production (i.e. the relationship between root mass per length and diameter must be determined empirically within a given site; Iversen *et al*., 2008). Scaling and conversion of minirhizotron data to ecosystem‐relevant metrics should only be done with extreme caution. Furthermore, root mortality has to be assumed as the ‘disappearance’ of a root and is therefore a conservative estimate of mortality that may be confounded by slow decomposition rates, especially in waterlogged ecosystems (Iversen *et al*., 2018). We also note that minirhizotrons do not allow the collection of information about chemical composition of fine roots or rhizosphere soil, so that data from soil cores are needed to estimate root nitrogen and carbon input into the soil. Also, in ecosystems with very dynamic uppermost organic layers, measuring fine‐root growth in the O‐horizon using minirhizotrons is complicated because of the shallow depth of this layer (Majdi *et al*., 1992; Majdi, 1996).

## 7 Root washing, sorting and storage

After roots have been removed from their surrounding soil environment, in the laboratory or the field, some processing steps remain before root traits can be assessed, including washing, sorting and storing.

### 1. Before washing

When rhizosphere soil needs to be collected for further soil analyses (e.g. for changes in soil chemistry or microbial community composition at the root interface) or for the measurement of rhizosheath size (see section **XXII. 3. Rhizosheath size**), roots are removed from soil manually using fine forceps allowing for the initial collection of rhizosphere soil. Rhizosphere soil is then often operationally defined and collected based on the research question. This may include all soil within a certain distance of the root surface (e.g. ≤ 2 mm), may be focused on soil that is loosely adhering to the root surface after initial cleaning of bulk soil (sometimes obtained by vigorous shaking of roots and collection of falling soil), or may be limited to tightly adhering soil collected manually from the roots using forceps or paintbrush.

### 2. Root washing

When the aim is to determine root biomass or root traits, the soil can be washed off the roots directly. However, the method used to wash the roots depends on the soil substrate and on the research objective. Ideally, roots should be washed free of soil immediately after harvest to minimise losses by respiration and microbial degradation. Because root washing is three‐fold to six‐fold more time consuming than root harvesting, a large root‐washing team may be needed, and we recommended organising the fieldwork in two teams, one harvesting the roots and one washing the roots. When it is not possible to harvest and wash the roots at the same time, roots may be kept in soil cores or monoliths stored in a refrigerator at 4°C for up to 48 h or kept frozen for longer periods at −20°C (Schuurman & Goedewaagen, 1965). Freezing minimises root respiration and microbial activity but also makes the roots more fragile and prone to breaking, which has consequences for a range of trait measurements, most particularly with respect to the analysis of root‐branch architecture.

We suggest the following well proven root washing method for mineral soils, but we list special cases below for which other methods will be needed. In the first step (soaking process), the frozen or unfrozen soil cores are placed individually in a bucket filled with water. The soaking allows the soil to slough off gently. The duration of soaking ranges from 30 min to one night depending on the soil clay content and compaction. During and at the end of the soaking period, the water is gently stirred, allowing the soil to fall from the living roots which generally stay suspended; organic matter, which floats, can be discarded. In some cases, particularly in organic soils, it may be important to closely inspect the floating organic matter to make sure no roots remain. This is particularly important if measurements to assess dead roots, which will often float, are important.

In the second step (sieving process), the water with suspended roots is poured over a fine‐mesh sieve or over a set of sieves of decreasing mesh size. The bucket with the remaining soil is refilled with water and the process of suspension and sieving is repeated. Roots retained in the sieves are transferred to a new bucket full of water. This soaking process is repeated as many times as necessary until soaking water is clear. All roots retained in the sieves are washed again using a gentle flow of water (preferably with the use of a garden hose shower head) while very gently rubbing the roots by hand over the fine‐meshed sieve. Care should be taken so that fine roots or old roots are not broken into small pieces and forced through the sieve. The hand‐rubbing step may generally be avoided in soil with low clay content. The mesh size used is critical to minimise root loss during washing and depends on the type of roots and objective of the research. It typically ranges from 0.2 to 2 mm. The use of a coarse sieve (2 mm) is more applicable for root systems that are relatively unfragmented (such as from pot experiments) and also more applicable for root weight determination than for root length determination. As an example, Caldwell and Fernandez (1975) found that 25% of the mass and 66% of the length of a root sample was lost when using a 1‐mm sieve compared with a 0.2‐mm sieve.

In the third step, when roots are free of mineral soil, they are transferred and spread in a flat tray filled with water to remove any remaining impurities (mainly adhering organic matter) with tweezers and to sort the roots (see section **VII. 3. Root sorting**). It can be helpful to use a tray that provides good contrast with the roots making them easier to identify. In particular, white roots, common among many herbaceous species, may be most visible against a black tray while dark brown roots, more common in mature roots of woody species, may be more visible in a white tray. Once roots have been fully cleaned, a final rinse with distilled or deionised water may be appropriate, especially if subsequent trait analyses will involve determining elemental content of micronutrients. Additionally, when the main objective is the determination of the chemical composition of the roots, it is important to reduce the soaking and washing time as much as possible to limit loss of water‐soluble compounds.

As a final note, it is important to recognise that not everyone works in a uniform way and washing may be faster or slower and more or less complete depending on the individuals, equipment and circumstances. Some investigators have chosen to standardise the amount of sample or the time spent on each sample to make the process more consistent (Metcalfe *et al*., 2007b). However, washing roots for accurate trait measurements and assessments of length does generally require thorough washing; by contrast, questions focused solely on the quantification of root biomass may not need exhaustive washing to obtain reasonably accurate assessments.

*Special cases.* When plants are grown in sand or artificial substrate (perlite, vermiculite, etc.), the first soaking step can be skipped. For clay soils, the addition of chemical dispersing agents in the soaking solution may facilitate the washing procedure. A list of possible chemicals and their concentration is given in Oliveira *et al*. (2000). For organic soils, soaking will not help to ‘untangle’ roots from surrounding peat, and can often result in a sloppy, opaque mess, hindering sorting and adding time to the process. In this case, the separation of live roots from organic debris is extremely time consuming and it may be necessary to limit processing to a representative subsample of soil or peat. However in such cases, it may also be helpful to employ additional tools such as jeweller’s glasses, fine forceps/tweezers, clear trays on top of a light source, and good ambient lighting.

Different root‐washing machines are available (Oliveira *et al*., 2000). The most commonly used are the hydropneumatic elutriation system (Smucker *et al*., 1982) and the Delta‐T root washer (Pallant *et al*., 1993). The main advantage of root‐washing machines is the standardisation of the process when different operators are involved, and the potential time savings when large numbers of samples are being processed. However, the elutriation process, in which lighter material is separated from surrounding soil by directed water spray and bubbled upward into a waiting sieve, delivers roots but also litter and other organic detritus. Therefore, the elutriated roots must still be tediously cleaned and sorted by hand. In addition, fine roots are often damaged during elutriation which may prevent further analysis of root‐branch architecture and morphology, and furthermore this technique does not work for roots growing in organic soils. Therefore, root‐washing machines are particularly useful for biomass estimation of roots growing in mineral soils.

### 3. Root sorting

Depending on the study objectives, cleaned roots are then sorted by categories, for example dead roots vs alive roots, fine roots vs coarse roots, absorptive fine roots vs transport fine roots, or by individual root orders (see section **IV. Below‐ground plant entities and root classifications**). Within each category, root subsampling is often done for further chemical, morphological or architectural trait measurements or for mycorrhizal colonisation or anatomical trait determination. Sharp tools are typically needed to dissect root systems into distinct categories. The most efficient (but expensive) tools are fine tip spring scissors, although scalpels and razor blades work fine (but damage plastic trays). Root sorting is most conveniently done in regular or photo trays. The use of jewellers’ glasses and fine forceps/tweezers is also sometimes advisable.

Sorting live from dead roots is challenging. Dead roots are far less elastic, their colour is darker, they are less dense and often float in water, and the lateral roots have often been broken off. The combined evaluation of these criteria determines whether a root is to be considered as alive or dead. Vital stains such as 2,3,5‐triphenyltetrazolium chloride and 2,3,4‐triphenyltetrazolium bromide or Congo red (Böhm, 1979) can also be used for quantitative estimation of living roots, but are not suitable for routine sorting or for roots to be used for chemical analyses (see also section **XIII. 2. Percentage of viable root cells**).

### 4. Separating roots by species

Traditionally, the relative species proportion in mixed root samples is determined by hand sorting or by tracing roots from the stems of known individuals. However, when roots are highly intermingled it is extremely challenging to recover whole root systems, as roots break during hand sorting. The identity of the broken roots can sometimes be assessed using anatomical (Brundrett & Kendrick, 1988) and morphological criteria such as colour, diameter, branching order and root tips (Hölscher *et al*., 2002; Schmid, 2002; Yanai *et al*., 2008). However, this method is very time consuming, user dependent and often subject to error. New techniques are now available to identify roots of different species without manual sorting, which may be appropriate in some cases. They are detailed in Rewald *et al*. (2012). For example, carbon isotope signatures allow discrimination of the roots from C_3_ and C_4_ grasses (Hobbie & Werner, 2004). The plant wax alkane and fatty alcohol compositions, which are species and plant part specific, have been used successfully to quantify the root mass fraction of each species in grass mixtures (Dawson *et al*., 2000; Soussana *et al*., 2005; Roumet *et al*., 2006). Near‐infrared spectroscopy (NIRS) also provides estimates of the percentage contribution of fine roots of herbaceous and tree species in mixed samples as well as the percentage of dead vs living grass roots (Roumet *et al*., 2006; Picon‐Cochard *et al*., 2009; Lei & Bauhus, 2010). Alternatively, molecular techniques have been used to determine the species identity of fine roots in species‐rich plant communities and to quantify the relative proportion of each species in mixed fine‐root samples (Jackson *et al*., 1999; Linder *et al*., 2000; Mommer *et al*., 2008, 2011; Jones *et al*., 2011; Frank *et al*., 2015; Zeng *et al*., 2015; Oram *et al*., 2018). DNA‐based approaches are some of the most promising techniques for root species identification and quantification and have the advantage of being less dependent on environmental conditions.

### 5. Storage after washing

If root traits cannot be assessed immediately after washing (and this is often the case given the time‐consuming nature of the hand‐sorting process), the root samples must be stored. Appropriate storage methods depend on the traits to be measured. After washing, subsamples of roots are generally taken and stored separately for determination of biomass, chemical composition, morphology, mycorrhizal colonisation and anatomy. The method of storage will depend on the intended use for the sample and are discussed below (see Table 3).

**Table 3:** Recommended storage methods of washed roots for further root trait measurements.

Storage method	Acceptable storage period	Biomass	Chemistry	Morphology	Mycorrhizal colonisation	Anatomy
Oven drying *c*. 60°C	> 1 yr	**√**	√ (total C, N, P, etc.)		**√**	
Freeze drying	> 1 yr		√ (labile/volatile; carbohydrates)			
+4°C	< 2 d			√		
−20°C	< 1 yr			**√**		
−80°C	> 1 yr			**√**		
Ethanol : water 60–95% : 40–5% v/v	< 2–4 wk at 20°C; < 1 yr at 4°C			√	**√**	
Glutaraldehyde : formaldehyde : cacodylate, 2% : 2% : 96% v/v						**√**

#### Determination of root dry mass and chemical traits

For measures of dry mass and chemical traits, root samples need to be dried as quickly as possible to remove water and inhibit mass losses through respiration, volatilisation and microbial and enzymatic degradation. For root dry mass determination, roots are usually oven dried at 60–70°C for 72 h (or until dry mass is stable) whereas coarse woody root dry mass determination requires oven drying at 101–105°C because wood contains bound water, in addition to free water (Williamson & Wiemann, 2010). For chemical analyses, samples should not be stored in water or a freezer because some soluble nutrients can be lost by diffusion in water or during thawing. Freeze‐drying is considered the best method; however lyophilisation systems are expensive and unavailable in many laboratories and so oven drying roots is a reasonable alternative, despite no single drying method could be relied on for all plant constituents. For standard elemental analysis (e.g. total carbon and nitrogen), samples are generally oven dried at 60–70°C for 48–72 h (until no additional water is lost); organic matter may be lost from the sample at lower temperatures (<60°C) through respiration of soluble sugars and enzymatic degradation and at higher temperatures (>80°C) through volatilisation and thermo‐chemical degradation (Smith, 1973).

#### Morphological analyses

For morphological analyses, root samples can be stored in water (assuming later chemical analyses are not required) or in moist conditions (e.g. wrapped in a damp paper towel) in a refrigerator at 4°C for preferably less than 48 h and up to 1 wk. If the storage period is longer than 48 h, roots should preferably be chemically preserved or frozen at −20°C. While the precise effect of storage method on root length, diameter and tissue density is not known, freezing is generally considered the best and cheapest method. Root samples are usually frozen in rigid reusable plastic containers without water. Rigid containers are preferable since they protect the roots from breakage during storage and transport. It is generally preferred to not re‐freeze roots to avoid further degradation of root tissues. If soil cores were frozen after harvest, the morphological traits of the washed roots should be measured immediately after thawing and washing. If freezing is not possible, roots can be chemically preserved (see Oliveira *et al*., 2000). While chemical preservation may be effective and appropriate for many analyses, future chemical analysis will be affected. Ethanol is the most commonly used preserving agent; however, a concentration less than 50 : 50, ethanol : water (v/v) introduces the possibility of pathogen growth in root tissue. The concentration of ethanol used should be proportional to the storage temperature; the higher the storage temperature the greater the ethanol concentration required (Böhm, 1979).

#### Mycorrhizal colonisation measurements

For estimates of mycorrhizal colonisation, roots should be immersed into 60–95% ethanol or methanol and stored at 4°C. The plant fixative formaldehyde : alcohol : acetic acid (FAA; 10% : 50% : 5% + 35% water, v/v) has also been used, but is not needed for simple assessments of colonisation and is therefore not recommended for this application because of its toxicity (see section **XIX. Mycorrhizal associations**).

#### Anatomical measurements

A specific procedure using a fixative composed of 2% (v/v) glutaraldehyde (pH 6.8) and 2% (v/v) formaldehyde (pH 6.8) in cacodylate buffer (0.1 M) is recommended for anatomical measurements, as described section **XIII. 1. b. Recommended microtechnique method**.

## 8 Horizontal plant mobility

Plants are sessile organisms but not all are bound to the place where they have germinated. Plants can explore horizontal space by several means: in addition to lateral growth below ground, they can also produce new roots along prostrate stems or new shoots along horizontal roots. This way they may abandon primary roots and move horizontally (Groff & Kaplan, 1988). This plant ‘movement’ differs from the movement of animals as it is realised by growth: a young part of a plant is positioned into a new place while older parts die and decompose sooner or later. As the horizontal movement of plants is connected with vegetative multiplication (several independent parts of a plant emerging from one seed may be formed), we call this clonal growth or clonality. The whole plant emerging from a seed is then called a genet, its physically independent part established by clonal growth is called a clonal fragment, and the smallest potentially independently viable unit is called a ramet (or offspring rooting unit). When horizontal movement is enabled by a stem and the stem connects ramets in a clone, we call those connections ‘stem spacers’; when horizontal movement is achieved by roots that connect ramets, we call such connections ‘root spacers’.

There are several traits that may help us to describe functionally important aspects of clonal growth: which organ is used for clonal growth, the multiplication rate, how fast a clone spreads and how long it remains connected. Although the combination of all those traits can give a more complete picture about clonal‐growth strategy, two traits from this list are not described here: multiplication rate, as it is very variable and depends on the biotic and abiotic conditions where the clone is growing; and morphology of a clonal‐growth organ, as its determination needs some morphological training. For both those traits, however, we recommend some specialised literature (Klimešová, 2018; Pausas *et al*., 2018; Klimešová *et al*., 2019). In spite of the central importance of clonality for plant fitness, both under resource stress or heterogeneity and in environments with strong interspecific competition (Jónsdóttir & Watson, 1997; Eilts *et al*., 2011), clonal trait data are only available for very restricted parts of the world, especially Central Europe (Klimešová *et al*., 2017).

### 1. Ability to grow clonally

*Ability to grow clonally* is the potential of a plant to produce physically independent rooting units (ramets) from one genetic individual (genet) (Aarssen, 2008) (Categories: clonal, nonclonal).

As this trait is not strongly conserved at the plant species level, data from databases (e.g. CLO‐PLA database; Klimešová *et al*., 2017) should be treated with caution and direct measurements on plants are recommended.

The clonal multiplication may be attained by different organs located above or below ground. Here, we refer only to clonal growth organs that not only lead to clonal growth but also enable sharing resources among rooting units and therefore affect resource acquisition. Consequently, we do not consider here the production of dispersible above‐ground clonal propagules (e.g. bulbils) that split from the maternal individual soon after formation, even though this clonal‐growth mode leads to the production of physically independent rooting units.

Clonal‐growth organs in the above‐mentioned sense are either horizontally growing stems (stem spacers, e.g. stolons or rhizomes) that are able to produce adventitious roots or horizontally growing roots (root spacers) that are able to produce adventitious shoots (Groff & Kaplan, 1988). The production of new spacers and rooting units via clonal growth is usually accompanied by the abandonment of old spacers and rooting units, lateral spread of the whole clone and the ability to share resources among rooting units. Lateral spread and mortality of old parts (the longevity of spacers and rooting units may or may not be synchronised) of a clone determine root lifespan and the volume of soil available to the clone for rooting via the physical connection by stem or root spacers.

Clonal and nonclonal plants differ in their root growth already from as early as the juvenile phase: clonal plants invest less of their resources into rooting depth and branching in nutrient‐rich patches compared with nonclonal plants (Šmilauerová & Šmilauer, 2007; Weiser *et al*., 2016). Interconnected above‐ground parts of a clone are synchronised in development (Hara & Šrůtek, 1995) and we can expect that also growth of fine roots in a clone is synchronised.

Clonal growth enables exploration of soil unparalleled in nonclonal plants. By selective placement of rooting units a plant may forage for resources in heterogeneous soil environments not only by roots as in nonclonal plants but also by clonal spacers bearing the roots. It can be achieved by morphological plasticity in spacer length, intensity and angle of branching (Slade & Hutchings, 1987; Oborny, 1994). Moreover, when one part of a clone is growing in a rich soil environment and another in a poor soil environment, the parts may share nutrients via spacers. Under experimental conditions, the growth by contrasting patches results in specialisation of a rooting unit for harvesting limiting resource and exporting it to the rest of a clone (Stuefer, 1998; F. Liu *et al*., 2016).

Clonal growth also has consequences for ecosystem functions as it leads to a horizontal redistribution of nutrients across the community and homogenises litter quality across poorer and richer soil patches, with consequences for spatial patterns of nutrient and water availability and soil organic carbon dynamics (Cornelissen *et al*., 2014).

Clonality is more prevalent in herbaceous species than in woody species and represents a still largely unexplored horizontal dimension of plants (Aarssen, 2008; Klimešová *et al*., 2018a). For the comparative ecology of clonality in functional terms, it is crucial to go beyond the determination of the morphological type of clonal‐growth organ and measure quantitative traits. Although there are some functional differences among individual morphological types (Klimešová *et al*., 2011a, 2017), their standardised description across biomes is rather difficult and their determination needs morphological training. Moreover, quantitative functional traits such as lateral spread are more directly connected with specific plant functioning than clonal‐growth organ morphology.

#### a. Sampling recommendations

When sampling a herb or small shrub, dig out the target plant from a soil monolith equal to the diameter of the projected above‐ground parts to 10 cm depth in low productive, mostly dry soil and to *c*. 30 cm in productive, mostly mesic soil. Then carefully wash the soil off the below‐ground plant parts by gently massaging the soil manually in or under (running) water. It is important to examine whether any part (except fine roots) was severed by excavating the sampled monolith, either laterally (horizontal stem of root) or below the monolith (stem or root); try to unearth such severed parts step by step, very carefully, starting from the point where it was cut until the end; then wash such parts as described above and record any other rooting unit (ramet) growing from them. Sample at least five plants, broadly representing the occurrence of different sizes and developmental stages per locality.

In some species, clonality may be restricted to certain parts of the season (especially the end of the growing season) or to certain developmental stages (old plants). Repeated observations during the growing season are useful when plants produce adventitious roots at the base but there is no spacer connected with the maternal rooting unit; this may be the result of clonality based on above‐ground dispersible propagules or a short‐lived connection. If the plant is not upright but creeping on the soil surface, follow the creeping stem cautiously and examine the presence of adventitious roots. Note that plants may combine different clonal modes and, in addition to an above‐ground creeping stem, may produce below‐ground spacers, therefore proceed cautiously during sampling. Clonality may be obligatory or facultative, occurring only in some plant individuals in a population or in some populations and not in others; therefore, replicate observations are needed.

Trees or large shrubs can usually not be dug out completely, although major disturbances (e.g. human, windthrow) can provide sudden opportunities for sampling. If one does not have equipment or permission to dig out a whole tree or shrub, an alternative is to dig a trench under the tree canopy close to the trunk or dig up young shoots emerging from the soil; assess whether such shoots are connected to the neighbouring tree (e.g. as root suckers via a root‐derived or via a stem‐derived clonal‐growth organ) or whether it has only a main root and therefore are likely to have originated from seed.

#### b. Storage and processing

Cleaned samples can be stored for *c*. 1 wk in a refrigerator for detailed evaluation. Alternatively, samples can be planted in a flower bed for later inspection.

#### c. Measurement procedure

**Fig. 12:**
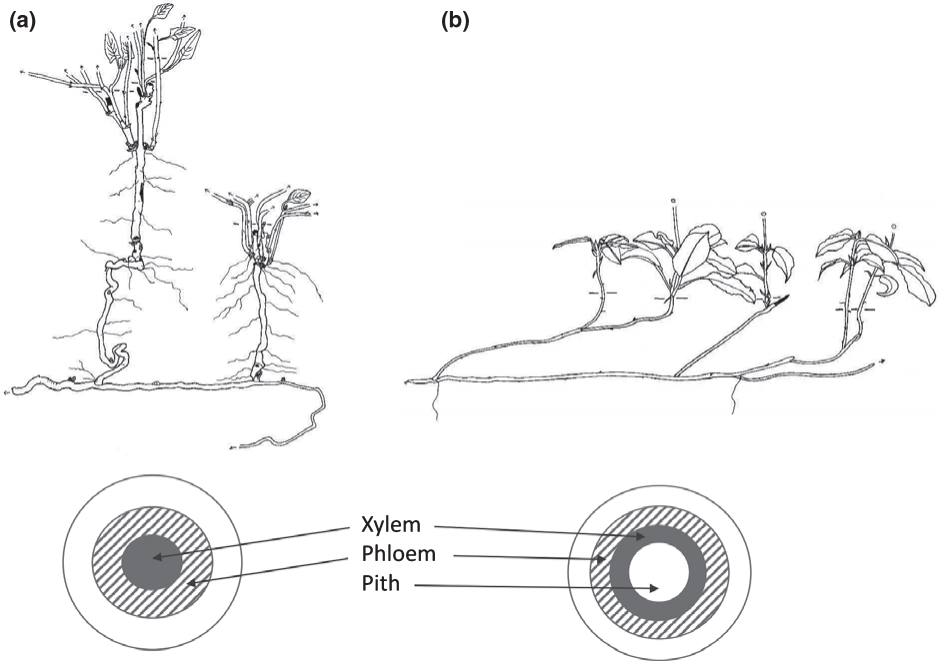
Anatomical and morphological differences between clonality derived from roots (a) and stems (b).

After excavation as described above, we define a plant as clonal if it forms stem or root spacers and associated offspring rooting units. However, this may in some cases be difficult to observe, as the connection between parental and offspring rooting unit may already have decomposed or because formation of more than one offspring is rare. In such cases, we recommend the following surrogate observations: (1) for dicotyledonous species, if the plant is forming a primary root and has no adventitious roots, even in older age, the plant is nonclonal. A plant is clonal if (2) the primary root is missing or overgrown by adventitious roots growing from below‐ground or above‐ground stems (stem‐derived clonal‐growth organ) or if (3) a horizontal organ from which a shoot is growing is anatomically a root structure and it bears adventitious buds (i.e. a shoot‐bearing root; anatomy and morphology should be used for the distinction; see Fig. 12).

#### d. Future research directions

For understanding clonal growth at large geographic scales it is necessary to study the diversity of clonal‐growth organs, their ecology and evolution. The clonal‐growth classifications are usually biome specific (for temperate Europe: Klimešová & Klimeš, 2008; for tropical grasslands: Pausas *et al*., 2018) and one system has only rarely been applied in other areas (Klimešová *et al*., 2011b; Filartiga *et al*., 2017). To develop a system with worldwide applicability is a difficult task that requires collaboration between specialists on different biomes, but the first steps towards this goal have been taken (Klimešová *et al*., 2019).

A more detailed categorisation of plants concerning clonality can be achieved at a finer scale of clonal‐growth intensities. Johansson *et al*. (2011) proposed a clonal index based on a combination of categorical values for lateral spread and multiplication rate from the CLO‐PLA database. Another important refinement is to assess whether clonal growth is always present in a species (obligatory), only under certain conditions (facultative) or only if induced by injury (regenerative). All those more detailed classifications of clonal growth need large data collection over multiple plant populations.

### 2. Lateral spread

*Lateral spread* is the distance a clonal plant grows laterally in 1 yr (typical units: m yr^−1^ or categories: < 0.01 m yr^−1^, 0.01–0.05 m yr^−1^, 0.05–0.25 m yr^−1^, > 0.25 m yr^−1^).

Lateral spread is realised through horizontal extension of stem or root spacers and enables root production in soil newly invaded by a clone. It can help a clone to exploit resource‐rich patches away from the patch in which the mother ramet started and thereby enhance its competitive strength. Support from the maternal part of a clone to young offspring ramets enable its establishment under strong competition, for example in grassland.

Lateral spread may range from a few millimetres to a few metres per year. Species with large lateral spread tend to prefer nutrient‐rich and mesic soil, whereas species with low lateral spread inhabit nutrient‐poor and dry places (Klimešová & Herben, 2015; Weiser & Smyčka, 2015). When offspring rooting units are produced once per year, lateral spread is equal to the distance between parental and offspring rooting unit. Lateral spread of herbaceous species is on average more extensive in large plants, but small plants may have considerable lateral spread as well, so that this relationship is not tight (Klimešová *et al*., 2011a).

It is probable that lateral spread interacts with other below‐ground traits but this has, to our knowledge, never been studied. As an example, extensive lateral spread necessitates for a plant to produce roots in habitats which may not yet have an established rooting zone, with probable consequences for their relationship with the soil microbiome (including mycorrhizal fungi and pathogens). Also, regarding root biomass allocation of clonal plants, the entry of a new ramet into a nutrient‐poor patch is not compensated by higher investment into root growth, compared to plants with an independent rooting unit (Stuefer, 1998), but rather by sharing resources from other rooting units that might grow in better conditions (F. Liu *et al*., 2016). A special case is represented by plants sprouting from roots where root foraging may be connected more directly with lateral spread. So far, however, the effect of soil heterogeneity on ramet placement in root‐sprouting plants has not been confirmed (Martínková *et al*., 2018).

#### a. Sampling, storage and processing recommendations

This trait is assessed only for clonal plants. See section **VIII. 1. Ability to grow clonally**. For adequately measuring this trait, it is particularly important to obtain an entire, connected clonal system or at least the youngest part with parental and offspring rooting unit.

#### b. Measurement procedure

Lateral spread measurements may be based on a retrospective method where the increment of the current year’s below‐ground organ may be recognised from older parts using morphological evidence such as remnants or scars of flowering stalks. This method requires regular growth and flowering only once a year. Another possibility is to mark the exact position of ramets in a community and by excavation of the clone after 1 yr; then measuring how far the clone spread from the marked position. This method is particularly recommended for nonseasonal climates. Both methods can only be used in plants where the connection between ramets lasts at least 1 yr. In cases in which the persistence of the connection among ramets is shorter, for example only for a fraction of the growing season (e.g. in above‐ground stems, stolons), inspection several times during (and perhaps just after) the growing season is necessary for the measurement.

In trees and large shrubs, trait assessment is complicated and has seldom been done. The reason is that they have long‐lived ramets that clonally reproduce only once per several years or even decades so that witnessing production of daughter ramets may be difficult. When the research aim is to assess this trait for woody plants, cautious consideration of species‐specific growing patterns is needed.

#### c. Future research directions

To better understand how clonal growth affects plant functions, future research should focus on the interplay of the yearly lateral spread with root traits, soil biota including mycorrhiza and soil properties. It would also be valuable to better describe intraspecific variability in lateral spread and its ecological correlates. Measuring lateral spread is also important for understanding the (metabolic) cost of clonal growth, but this has seldom been considered so far (Batzer *et al*., 2017).

### 3. Persistence of connection between ramets

*Persistence of connection between ramets* is the time an offspring rooting unit remains connected to the clonal network (typical units: yr or categories: < 1 yr, 1–2 yr, > 2 yr).

The persistence of the connection between ramets ranges from one season to decades in herbaceous species. Highly persistent spacers may result in large clones with numerous connected rooting units. The spacer longevity may be equal to the longevity of the rooting unit itself, shorter or longer. When spacers live longer than the above‐ground parts of offspring rooting units they still bear roots and determine the maximum potential longevity of roots (Klimešová *et al*., 2018b). Persistence of spacers increases with environmental stress regimes and is longer at dry, nutrient‐poor and cold ends of environmental gradients compared with in mesic, fertile and warm ones (Jónsdóttir & Watson, 1997; Klimeš, 2008). This may hold true also within species: along an altitudinal gradient *Rumex alpinus* had more persistent rhizomes at high than at low altitude (Šťastná *et al*., 2012).

We do not yet know how the longevity of spacers co‐varies with root traits, except for root longevity. We can expect that depth of rooting will be lower in short‐lived connections than in long‐lived ones while mycorrhizal symbiosis, biomass investments into roots and further traits may also be affected. Such relationships have remained unexplored to date.

#### a. Sampling, storage and processing recommendations

See section **VIII. 1. Ability to grow clonally**. Again take care to select a whole connected clonal system.

#### b. Measurement procedure

**Fig. 13:**
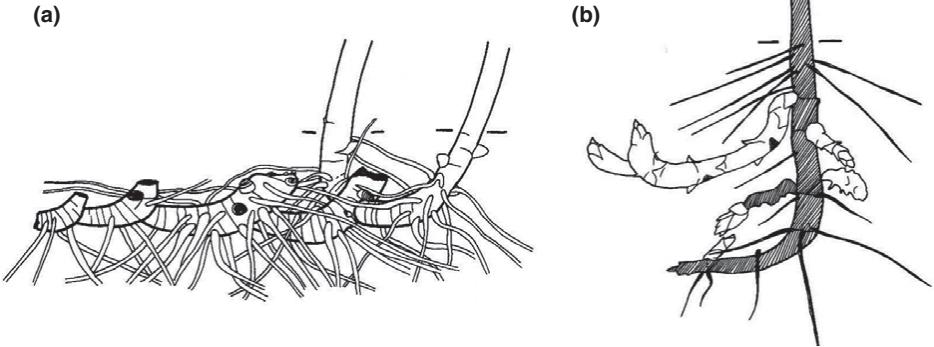
Examples of plants with long (a) and short (b) persistence of their below‐ground clonal organ. (b) Below‐ground plant parts during winter rest: black parts of the rhizome system are decaying while white parts are living and will sprout at the beginning of a growing season.

After concluding that a plant is clonal, check for remnants or scars of old rooting units along the targeted clonal‐growth organ. If found, and if they look progressively decayed towards the distal end of the organ, or if there is another regular structure that may be attributed to annual increments, counting them may help to estimate the longevity of clonal fragment (Fig. 13). Such an approach is not always possible as the connection between rooting units may decay sooner than the rooting units or because branching is irregular and rooting units are produced more or less often than once a year. In such cases it is better to classify the longevity of connection into categories. In some dicotyledonous herbs of seasonal climates, a chronological method may be used and annual rings on the oldest part of the spacer counted (Schweingruber & Poschlod, 2005).

In trees and large shrubs, assessment of the persistence of connections is complicated, again because of species‐specific growing patterns, and has seldom been done. Be aware that, in large clones of woody plants, connecting roots and rhizomes may survive a longer period than any ramet due to being grafted onto other clones.

#### c. Future research directions

The ecological context and consequences of this trait for both plant and ecosystem are currently poorly characterised. Particularly, we need to better understand how the persistence of connection between ramets keep influencing plant functioning even after the death of above‐ground plant parts; to what extent this trait varies at the intraspecific level and in response to what drivers; whether long‐lived spacers bear functioning roots and bud banks; and to what degree long‐lived spacers buffer plant growth over time and space in comparison with short‐lived spacers.

## 9 Below‐ground allocation

The relative amount of leaves and fine roots (and wood, for shrubs and trees) varies strongly among species and with edaphic and environmental conditions (Poorter *et al*., 2012b, 2015; Reich *et al*., 2014). The relative distribution of plant biomass above ground and below ground can be used to understand plant strategies (Evans, 1972; Nadelhoffer *et al*., 1985; Malhi *et al*., 2011; Cornelissen *et al*., 2014), as well as the consequences of these strategies for ecosystem carbon, water, and nutrient cycling (Freschet *et al*., 2013; Jackson *et al*., 2017). Furthermore, the amount of biomass in different plant compartments is important for model parameterisation and validation (Malhi *et al*., 2011; De Kauwe *et al*., 2014; Song *et al*., 2017).

The distribution of biomass across different plant compartments has generally been referred to as ‘allocation’, but this term has been inconsistently used in the literature (e.g. Litton *et al*., 2007; Poorter *et al*., 2012b; Reich *et al*., 2014). Here, we conceptualise ‘allocation’ at three spatio‐temporal scales: (1) the short‐term and small‐scale flux of photosynthate to respiration, biomass construction, carbohydrate storage, and exudation (‘sugar allocation’; Poorter *et al*., 1990), which is covered later in this handbook under section **XVII. Root respiration and exudation**, (2) the annual flux of organic matter to newly produced leaf, wood, or root biomass (‘growth allocation’; De Kauwe *et al*., 2014), and (3) the resulting distribution of biomass in short‐lived and long‐lived plant compartments (‘biomass allocation’; Litton *et al*., 2007; Poorter & Sack, 2012; Poorter *et al*., 2012b; note that in a young or annual plant, growth allocation and biomass allocation may be the same). All three types of allocation can be studied at scales ranging from organs to entire plants, and from individual plants to plant communities and entire ecosystems.

Conceptually, ‘allocation’ integrates processes ranging from gross primary production to respiration, exudation, loss of volatile organic compounds, the export of organic compounds to mycorrhizas, biomass production and mortality, and biomass losses to herbivory (Poorter *et al*., 2012b). However, in practice, allocation is quantified as the end result of these processes; namely, the net accumulation of carbohydrates or biomass in a given plant compartment over a given time period or the net amount of carbohydrates or biomass in a given plant compartment at a specific time. Allocation patterns vary with plant size and across species (Poorter & Sack, 2012; Poorter *et al*., 2015), and the controls over short‐term and longer term allocation among different plant compartments remains a vibrant field of research. A general consensus is that allocation varies in response to both endogenous and exogenous factors (e.g. source–sink balancing, photosynthetically active radiation, air temperature, soil temperature and resource availability; Farrar & Jones, 2000; Litton *et al*., 2007; Reich *et al*., 2014).

Below‐ground allocation is a key source of uncertainty in ecological and modelling analyses (Malhi *et al*., 2011; De Kauwe *et al*., 2014), and the amount of below‐ground plant production and biomass is likely to be much greater than what is currently predicted across some ecosystems and plant types (Robinson, 2007; Iversen *et al*., 2015). Below‐ground allocation can encompass many different organs, each of which has a range of purposes and longevities. Short‐lived fine roots are responsible for plant acquisition of water and nutrients, while in woody systems, longer lived coarse roots, as well as buried, below‐ground stems, serve to anchor the plant and connect the more distal, fine roots with the rest of the plant (de Kroon & Visser, 2003). These longer lived below‐ground organs also provide a location for the storage and accumulation of nonstructural carbohydrates needed for growth, maintenance, and storage (Patrick *et al*., 2013; see section **XIV. 6. Root nonstructural carbohydrates**).

To understand below‐ground allocation, we discuss how to quantify plant production and biomass, both above and below ground (e.g. Malhi *et al*., 2011). We conclude by considering below‐ground bud‐bank size and depth, which is related to plant capacity for storage and regrowth.

### 1. Biomass allocation

*Root mass fraction* is the ratio of root dry mass per total plant standing dry biomass (typical units: g g^−1^) (frequent abbreviation: root mass fraction (RMF)). This trait can be measured for entire below‐ground systems, that is the below‐ground mass fraction, or specific organs, for example fine‐root or coarse‐root mass fraction, or rhizome mass fraction.

*Root‐to‐shoot ratio* is total below‐ground plant biomass divided by above‐ground plant biomass (typical units: g g^−1^).

Biomass pools are generally estimated at one point in time and expressed in units of g biomass or C per plant, per g soil or per m^2^ ground area before conversion to fractions or ratios. While the ‘root‐to‐shoot ratio’, or its inverse (Mokany *et al*., 2006; Iversen *et al*., 2015), is a frequently used alternative expression for RMF, we advocate using a fraction rather than a ratio. Indeed, a fraction can be easily divided into more than two compartments, allows for a more straightforward understanding of the independent variation in multiple compartments and has a simpler statistical distribution (Poorter & Sack, 2012).

The distribution of standing biomass among plant organs is a result of both the amount of production and the turnover time of each biomass compartment, and gives an integrated perspective on the developmental trade‐offs encountered by plants (Litton *et al*., 2007). The distribution of standing biomass can be directly related to plant physiology and relative growth rate and used to understand plant responses to changing environmental conditions, including the ‘functional partitioning’ of plant biomass in response to changes in light and temperature, as well as water and nutrient availability (Poorter *et al*., 2012b). For instance, fine‐RMF is one of the below‐ground plant traits that responds most substantially and consistently to variation in soil nutrient availability (Freschet *et al*., 2018), although its role in nutrient acquisition remains unclear (Kulmatiski *et al*., 2017; Dybzinski *et al*., 2019).

While a large range of root traits is important for understanding root construction, turnover and physiology, RMF is an estimation of the relative amount of plant biomass dedicated to performing this range of functions. However, in some woody species, total plant biomass is dominated by long‐lived woody tissues (e.g. coarse roots and stems below ground, and the woody stem and branches above ground), confounding comparisons of RMF among diverse plant growth forms. Furthermore, in some herbaceous species the rhizome mass fraction can be as high as 50% of below‐ground biomass (Fiala *et al*., 2012). In such cases, other ratios that target specific functional plant biomass compartments, including the ratio of fine‐root to leaf biomass (Freschet *et al*., 2015a), the ratio of absorptive fine‐root biomass to transport fine‐root biomass (McCormack *et al*., 2015a), or the ratio of rhizome to root biomass, may allow for a better understanding of plant functioning (Mokany *et al*., 2006).

Root mass fractions are most easily measured on short‐statured plants or plants growing in pots, although they have been assessed at the stand level in the field, in forests, and across multiple biomes (reviewed in Mokany *et al*., 2006; Poorter *et al*., 2012b; Iversen *et al*., 2015). Root mass fraction is influenced by the seasonality of plant growth and turnover, and varies with plant size and age as well as the volume of soil available for root growth (Böhm, 1979; Litton *et al*., 2007; Poorter *et al*., 2015).

#### a. Sampling recommendations

##### Plants in containers

The sampling of young plants growing individually in pots is relatively straightforward. At harvest, depending on the question of interest, above‐ground biomass is separated into leaves, stems and – if desired – other fractions such as reproductive structures. The soil column is removed from the pot and roots are gently washed clean with water (see section **VII. Root washing, sorting and storage**). Care has to be taken to remove remaining soil particles attaching to the roots, especially grains of sand that look small but represent considerable mass due to their high density. Fine roots can be separated from coarse roots, as well as below‐ground stems or rhizomes, and all fractions are dried to constant mass and weighed. The same methodology can be used for plant communities growing in larger containers; if these communities are monocultures, subsampling a smaller area of the container can help to reduce the processing work associated with higher root densities associated with multiple plants.

##### Individual plants in the field

Individual herbaceous plants growing in a dense vegetation are very difficult to separate from each other, and this has been done only rarely (Freschet *et al*., 2015b). However, individual trees of small to large size are somewhat easier to harvest (Montero *et al*., 2005; Kuyah *et al*., 2012; see section **VI. Experimentation and sampling in laboratory and field**). For woody plants, leaves and branches can then be removed from the main stem and dried and weighed separately. The circumference of large stems can be measured at different heights to calculate volume, and wood slices sampled throughout can be used for fresh/dry mass conversion and mass calculation. Below ground, coarse roots can be mechanically removed to the point at which diameter becomes too small, weighed fresh, and subsampled to determine fresh/dry mass ratios. Separate studies of terminal parts of roots can be used to establish allometric relationships between circumference of roots at a given point and the mass of their distal parts that may have been missed in the sampling effort (van Noordwijk *et al*., 1994). It has been estimated that for *Pinus pinaster*, those parts that are not excavated anymore represent 17% of total root mass for small trees, and 4% for larger trees (Danjon *et al*., 2013b).

##### Plant communities in the field

For stands of plants it is generally not feasible to dig up the entirety of the below‐ground system, so above‐ground and below‐ground biomass are quantified using a range of techniques:

*Fine‐root biomass.* The biomass of fine roots may be quantified from root tracing, soil cores, or soil monoliths, or a combination of these three methods (see section **VI. Experimentation and sampling in laboratory and field**). When designing a sampling scheme, it is important to consider whether the data must characterise an individual plant species or an entire plant community, whether the depth distribution of fine roots is required, and the soil characteristics of the site. These factors are best determined by performing preliminary harvests to characterise the lateral spread of fine roots, soil texture and ease of root excavation. Preliminary harvests also help ascertain whether the fine roots of interest can be visually differentiated from fine roots of other plants in the area, a crucial piece of information that can guide development of a robust sampling protocol. Further discussion of below‐ground sampling stratification and sample sizes can be found under section **VI. Experimentation and sampling in laboratory and field**. Recommendations for the timing of sample collection can be found in Sala *et al*. (1988), while Klironomos *et al*. (1999) and Anderson‐Teixeira *et al*. (2015) also have useful advice on sampling stratification and sample sizes for below‐ground and above‐ground sampling, respectively.

Root tracing is accomplished by tracing coarse roots that emerge from above‐ground plant structures down progressively smaller roots until root branches containing intact, distal, fine roots are located. The tracing process must be performed carefully to prevent breaking off fine roots and root branches may be sampled with their surrounding soil and separated in the laboratory. For nonwoody or small plants, collection of all root biomass for an individual may be possible provided the lateral spread of roots is known. Root tracing ensures a high confidence in the species‐level identification of fine roots and is often used in conjunction with methods for studying root morphology and architecture, as well as physiological processes. The primarily limitation is the lack of spatial context: fine‐root biomass collected by tracing cannot be separated into depth intervals or directly scaled to ground area (but see Pecháčková *et al*., 1999).

By contrast, collection of soil cores (see section **VI. 3. Field observation, experiments and sampling**) results in a vertically explicit measure of fine‐root biomass, and depth increments can be used to calculate root biomass extinction coefficients (Jackson *et al*., 1996; see section **XI. Root spatial distribution**). However, since the above‐ground plant organs are no longer connected to fine roots, it can be difficult to identify the fine roots in soil cores to species and severed root branches within soil cores can limit concurrent observations of root branching and orders (see section **VII. 4. Separating roots by species**). Depending on the size of the soil core and the heterogeneity of fine roots at the site, scaling fine‐root biomass from soil core volume to ground area may require spatially explicit sampling to take into account the spatial structure of the plant community (Taylor *et al*., 2013).

Collection of soil monoliths that span the rooting zone can increase the area and volume of soil sampled as well as reduce the risk of soil compression associated with coring organic horizons (see section **VI. 3. Field observation, experiments and sampling**). Monoliths encompass both lateral and vertical spatial heterogeneity and provide a robust measurement of fine‐root biomass that can be scaled to ground area. Furthermore, in low‐stature vegetation, monolith sampling with intact above‐ground plant parts ensures paired observations of above‐ground biomass and below‐ground biomass, including fine roots (e.g. Shaver & Chapin, 1991). However, the time required to sort fine roots from monoliths can make their use impractical, and a common compromise is to collect soil monoliths from surface soils and soil cores from deeper in the soil profile (e.g. Mack *et al*., 2004). Fine‐root biomass can also be estimated from minirhizotrons using the allometry but has associated caveats related to scaling (see section **VI. 3. Field observation, experiments and sampling**).

*Coarse‐root*, *below‐ground stem and rhizome biomass*. An accurate assessment of the standing biomass of coarse roots and below‐ground stems which are distributed over large spatial scales generally requires a larger sampling area than that of fine roots. In forested ecosystems, this can require the use of a high‐pressure air system or water system to remove soil from an area of the ground that is on average larger than the horizontal projection of the branch spread of a tree (Böhm, 1979 and references therein), or alternatively, a backhoe or power winch could be used to pull out the entire root bole and coarse‐root system of individual trees (Norby *et al*., 2001; see section **VI. 3. Field observation, experiments and sampling** for more information). In addition, ground‐penetrating radar (GPR) has been used to nondestructively assess coarse‐root biomass and distribution in woody ecosystems (Stover *et al*., 2007). In herbaceous ecosystems, coarse roots, below‐ground stems and rhizomes can be excavated using the soil monolith technique described above.

*Shoot biomass.* In short‐statured ecosystems such as grasslands and shrublands, total above‐ground living biomass (i.e. ‘shoots’) is generally sampled from destructive harvests (Sala & Austin, 2000), where leaves, branches and stem wood are separated into living and dead compartments and by species. As above, carefully consider the size and number of sampling quadrats. Alternatively, biomass can be inferred nondestructively. The pin drop method, also referred to as point‐intercept method, involves dropping a thin pin or rod through the canopy and counting the number of times the pin makes contact with the various plant functional types present. Allometric relationships that describe the linear slope between pin hits and the above‐ground biomass can then be applied (Frank & McNaughton, 1990; Shaver *et al*., 2001). Provided allometric relationships between woody basal area (of short‐statured shrubs) and above‐ground biomass have been established, surveys of stem basal area can also be used for nondestructive quantification of above‐ground biomass (Berner *et al*., 2015).

In tall‐statured ecosystems, such as forests, the quantification of above‐ground biomass requires different approaches that differentiate ‘shoots’ into leaves and wood:

*Leaf biomass.* In deciduous forest stands, leaf‐litter baskets constructed from mesh with narrow‐diameter openings and suspended above the ground can be used to assess the total amount of leaf biomass (e.g. Nadelhoffer *et al*., 1985; Norby *et al*., 2001). In evergreen forests, leaf biomass must be measured by felling trees and then separating leaves or needles from woody plant parts, or doing this in a few locations to develop allometric relationships (Gower *et al*., 1997). Alternatively, it can be estimated from annual rates of litter production and measurements of leaf lifespan. Nondestructive measures, such as light sensors positioned above the canopy and below the canopy (Sala & Austin, 2000; Norby *et al*., 2003; McCormack *et al*., 2015b), or terrestrial laser scanning (Calders *et al*., 2015; Greaves *et al*., 2015), can also be used to assess total above‐ground biomass, but require ground‐truthing and allometric scaling (Bréda, 2003).

*Woody biomass (stems and branches)*. Woody biomass of trees can be quantified by measuring the circumference at breast height and then applying allometric relationships that relate tree basal area to height and taper observed for a given type of tree stem. Wood volume can be calculated by assuming the tree stem is in the shape of a truncated cone, and volume is then converted to wood biomass using data from increment cores (i.e. small‐diameter bores that sample a pencil‐sized piece of wood horizontally from the stem) that assess the distribution of wood densities throughout the stem (Norby *et al*., 2001; Malhi *et al*., 2011). Wood biomass extrapolated using this method encompasses living stems and branches as well as the ‘dead’ heartwood.

#### b. Storage and processing

See section **VII. Root washing, sorting and storage**.

#### c. Calculations

Biomass pools of plant organs above‐ground and below‐ground must be converted to similar units and then summed to obtain total plant biomass. Below‐ground mass fraction (or any component of it, e.g. fine‐root mass fraction, coarse‐root mass fraction) is then calculated by dividing the below‐ground biomass pool by the total plant biomass. See Poorter & Sack (2012) for further recommendations for the analysis of biomass allocation.

Generally, these calculations include the majority of plant biomass but should not be considered complete because they often do not include biomass of inflorescences or fruit. This oversight is most common in hard‐to‐sample forested ecosystems, but the reproductive fraction is often ignored even in more easily sampled in pots or short‐statured grassland ecosystems. It should be noted that, depending on the methodology used, these biomass estimates may also be influenced by past herbivory, leaf mass loss during senescence, and error associated with the application of allometries.

#### d. Future research directions

Understanding the distribution of plant biomass above and below ground has long been an area of active research, and there are well known drawings and pictures of below‐ground compared with above‐ground allocation in both herbaceous and woody plant communities (Weaver & Kramer, 1932; Böhm, 1979; Kutschera *et al*., 1992; Kutschera & Lichtenegger, 2002). While we generally understand large‐scale biogeographic patterns in biomass allocation (Reich *et al*., 2014), and the influence of environmental factors as well as plant size and phylogeny (Poorter *et al*., 2012b, 2015), we can advance our understanding of plant below‐ground biomass allocation in some ways. These include the development of techniques that quantify below‐ground biomass nondestructively (e.g. Franz *et al*., 2013) as well as the use of remote sensing or allometric relationships to predict below‐ground plant traits from above‐ground plant traits (e.g. Reich *et al*., 2014; Serbin *et al*., 2014). For plants grown in pots, nondestructive imaging by means of nuclear magnetic resonance (NMR) or computed tomography is now feasible (van Dusschoten *et al*., 2016), while molecular barcoding techniques are promising for understanding the root distribution for individual species (Partel *et al*., 2012; Herben *et al*., 2018a).

### 2. Growth allocation

*Below‐ground growth allocation* is the fraction of net primary production (NPP) that is accounted for by newly produced below‐ground biomass (typical units: g g^−1^, but in practice often reported as a %). This trait can be further separated for specific below‐ground plant entities (e.g. rhizome growth allocation, fine‐root growth allocation).

The flux of newly produced biomass into different plant compartments is generally estimated over a year or a growing season and requires multiple biomass estimates depending on the timing of the production and mortality of a given plant compartment. Care should be taken to define the time period of interest, as plant allocation of NPP varies over the course of the year in response to endogenous and exogenous conditions, with the production of leaves, wood, and fine roots generally offset in time (e.g. McCormack *et al*., 2015b; Iversen *et al*., 2018). Furthermore, while the allocation of NPP to leaves tends to be rather stable across changing environmental conditions, trade‐offs are often observed between NPP allocated to fine roots compared with stem wood (Litton *et al*., 2007; Malhi *et al*., 2011), or among above‐ground and below‐ground compartments (Poorter *et al*., 2012b). While this trait can be measured for a specific below‐ground plant organ (e.g. rhizomes, coarse roots, fine roots) or for the entire below‐ground system, the main interest in below‐ground growth allocation is generally in fine‐root growth allocation, which comprises between 20 and 30% of all new production in trees (Jackson *et al*., 1997; McCormack *et al*., 2015a), and can be much higher in forbs and grasses (more than 50%, Mokany *et al*., 2006).

#### a. Sampling recommendations

##### Plants in containers

See sampling recommendations above for determining the biomass of plants grown in containers (**IX. 1. a. Sampling recommendations** **)**. For young plants, such as most pot‐grown plants, biomass and growth allocation are essentially the same.

##### Plant communities in the field

Quantification of below‐ground production should include the biomass production of fine roots, coarse roots, and rhizomes:

*Fine‐root production.* Minirhizotrons, which are clear tubes inserted into the ground and repeatedly imaged to capture the birth, growth and death of fine roots, can be used to assess the rate and timing of annual fine‐root production (see section **VI. 3. Field observation, experiments and sampling** and **XVI. Root dynamics**). Estimates of root length production from minirhizotron windows can be converted to root biomass production through allometric scaling using continuous relationships between SRL and diameter of fine roots sampled from the soil and by assuming a depth of field for the minirhizotron images (e.g. Metcalfe *et al*., 2007a; Iversen *et al*., 2018; but see section **VI. 3. Field observation, experiments and sampling** for caveats associated with these calculations).

Alternative recommendations are to determine the turnover rate of the fine‐root population from minirhizotrons (i.e. the ratio of fine‐root production to standing crop at peak production; Gill & Jackson, 2000), and to apply this turnover rate to the biomass of fine roots in soil cores taken in the same location (Majdi & Andersson, 2005). However, there are caveats associated with this, as minirhizotrons and soil cores sample different portions of the heterogeneous fine‐root population (Guo *et al*., 2008b). Furthermore, stable isotope labelling (Matamala *et al*., 2003) or natural abundance ^14^C dating have been used to determine the turnover rate of fine roots, but ^14^C dating loses resolution at the annual scale of interest here (Gaudinski *et al*., 2001; Iversen *et al*., 2018). In‐growth cores or root windows may be used to assess fine‐root production in cases for which minirhizotrons are not feasible (see **VI. 3. Field observation, experiments and sampling**). Given the large amount of spatial variability in fine‐root production in most ecosystems, we do not recommend sequential coring to assess fine‐root production, although this was common in the past (Vogt *et al*., 1998).

*Coarse‐root and rhizome production.* Coarse‐root production is a component of below‐ground production that is rarely measured (Malhi *et al*., 2011). Often, coarse‐root production is assumed to be proportional to production or basal area of above‐ground stems in woody ecosystems (Norby *et al*., 2001; Giardina & Ryan, 2002; Litton *et al*., 2007), where the proportion is derived from destructive harvests. In some instances, allometric relationships between coarse‐root production and the length and diameter of coarse roots have been established (see Van Do *et al*., 2019). In some cases, dendrobands or growth ring measurements (as described for wood, below) have been used to assess the annual diameter growth of coarse roots (Buchwal *et al*., 2013; Addo‐Danso *et al*., 2016). For herbaceous species, rhizome productivity can be measured as the biomass of one spacer multiplied by the number of spacers produced per year (see also methods under section **VIII. Horizontal plant mobility**).

Different approaches of above‐ground production quantification are necessary for herbaceous and woody vegetation, as detailed below:

*Shoot production.* In short‐statured annual grasslands or herbaceous systems in which all above‐ground biomass is newly produced within 1 yr (i.e. shoots), clip plots harvested at ‘peak’ biomass may be used to assess green shoot production (i.e. peak standing crop is equal to above‐ground production), while repeated clip plots at slightly different locations each time may be used in a perennial grassland system (Singh *et al*., 1975). After harvest, herbaceous components such as leaves, blades, stems or sheaths are separated into living and dead compartments and by species if desired. The size and number of sampling quadrats should consider vegetation patch size, microtopography, and whether nonvascular species are of interest (Shaver & Chapin, 1991).

*Leaf production.* Repeated collection of leaf litter or needle litter from elevated litter baskets can be used to assess leaf production of woody vegetation in both deciduous and evergreen forests (e.g. Sala & Austin, 2000; Norby *et al*., 2001; Malhi *et al*., 2011). The use of litter baskets for measuring leaf production relies on the assumption that leaf‐litter production is equal the production of new leaves, although a species‐specific correction needs to be made for mass lost during senescence (Norby *et al*., 2000). When deploying litter baskets, it is important to consider the duration and season over which the basket will be deployed so that the entire annual flux of litter is captured. The ground area and location that litter baskets will occupy are also important considerations. Larger baskets will increase the sampled area but the footprint of the basket must reside underneath a relatively homogenous canopy. If the canopy is heterogeneous, litter baskets may be placed beneath the canopy of individual trees and litter fluxes may be scaled based on the canopy footprint or basal area.

In woody evergreen plants, current‐year leaves can often be distinguished based on branching patterns or tissue morphology of the stem from which they protrude (Shaver, 1986; Bernier *et al*., 2007). Annual leaf production can therefore be assessed during destructive harvests if current‐year leaves are separately sorted and quantified:

*Wood production.* Wood production can be divided into primary production (the elongation of stems, branches and twigs) and secondary production (thickening of stems, branches and twigs). For trees, quantification of total wood production is often estimated from measurements of secondary wood production from increment cores in trees with distinct annual growth rings, or through repeated surveys of stem circumference at breast height (1.3 m), where circumference is assessed manually or through the use of dendrobands (Nadelhoffer *et al*., 1985; Malhi *et al*., 2011). For smaller‐statured woody plants, callipers can be used to assess diameter at the base of the stem (Smith & Brand, 1983; Berner *et al*., 2015). In some instances, annual growth rings have been used to quantify secondary wood production in shrubs but application of these methods requires considering the particular morphology and growth pattern of the species in question (see Myers‐Smith *et al*., 2015).

Measurements of stem circumference or diameter are scaled to basal area and then woody biomass using allometric relationships (Sala & Austin, 2000; Norby *et al*., 2001), and biomass changes through time can be attributed to the production of new wood. Ideally, allometric relationships would account for stem taper and wood density as well as encompass the range of plant heights over which the allometry it to be applied. It is important to keep in mind that when secondary wood growth increments are used to infer total wood production (including both secondary and primary growth), there is an underlying assumption that tree morphology and growth strategy does not change over time; this may not be true for ecosystems that are in the process of a community composition shift or undergoing dramatic environmental change (Shaver, 1986; Bernier *et al*., 2007). A separate estimate of branch and twig production can be inferred from woody debris captured in litter baskets or in marked plots on the forest floor (e.g. Sala & Austin, 2000; Norby *et al*., 2001). Transects can also be established and repeatedly surveyed for freshly fallen branches (Malhi *et al*., 2017).

#### b. Storage and processing

See section **VII. Root washing, sorting and storage**.

#### c. Calculations

Production fluxes for plant organs above and below ground must be converted to similar units and then summed to obtain total production. The proportional contribution of below‐ground production (or any component of it, e.g. fine‐root production, coarse‐root production) can then be calculated relative to total NPP and expressed as a fraction (g g^−1^) or a percentage (%). Discussion of the relative uncertainty in the measurements of production for each plant compartment, and how these errors may be propagated to estimates of total NPP and fractional growth allocation, can be found in Walker *et al*. (2019).

While leaves, wood and fine roots encompass a large portion of plant production, they do not comprise the entire flux of NPP (Malhi *et al*., 2011). A complete inventory of NPP would also quantify production of flowers, fruits, exudates, volatiles as well as distribution of production to mycorrhizal symbionts, root nodules, parasites and herbivores. These fluxes, while rarely measured, maybe an important component of some ecosystems (e.g. fruit can comprise up to 15% of production in tropical ecosystems; Malhi *et al*., 2011). Furthermore, it should also be noted that the traditional methods of measuring leaf, wood and root production presented here rely heavily on biomass accrual between two observations. Any biomass produced and recycled between the observations is therefore not included in these estimates.

#### d. Future research directions

Because of the time‐consuming nature of measuring fine‐root production, fine‐root growth allocation remains poorly understood (McCormack *et al*., 2015a). These questions are now being assessed across research networks where multiple researchers quantify below‐ground and above‐ground production across large environmental gradients using the same techniques at each location (e.g. Anderson‐Teixeira *et al*., 2015). In addition, we know very little about the relative timing of the allocation of NPP to the production of fine roots, leaves and wood, which has implications for our understanding of changes in plant phenology and associated ecosystem processes in response to changing environmental conditions (McCormack *et al*., 2015b; Iversen *et al*., 2018; see section **XVI. Root dynamics**). The development of techniques that more easily and quantitatively sample fine‐root production, potentially by developing relationships with above‐ground processes, are needed (e.g. Laiho *et al*., 2014), although these techniques are likely to need calibration across multiple ecosystem types and environmental conditions.

### 3. Below‐ground bud‐bank size

*Below‐ground bud‐bank size* is the number of buds the plant has at disposal on below‐ground organs (typical units: cardinal number).

It reflects the capacity of plants to regenerate after damage that removes all or part of above‐ground biomass or to regrow after seasonal rest (dormancy due to dry or cold periods in seasonal climate). Below‐ground bud‐bank size can also be expressed per area of ground. However, this community‐level measurement (see section **III. Species‐level vs ecosystem‐level measurements**) depends not only on species composition but also on the size of plants; in cases for which communities differ substantially in plant size, such metrics may not be meaningful.

Perennial plants invest resources for current needs and future growth. Future investment is in the form of storage carbohydrates (see section **XIV. 6. Root nonstructural carbohydrates**) as well as structures that serve as a warehouse for carbohydrate storage and/or bear meristems for production of new shoots after damage or seasonal rest (Pausas *et al*., 2018). While the carbon investment in, and resulting biomass of, buds themselves is negligible in comparison with bud‐bearing organs (Vesk & Westoby, 2004), the production of below‐ground buds affects plant architecture and size. Woody plants that store buds in below‐ground bud banks (resprouters) are typically shorter than woody plants without this storage that regenerate from seeds (seeders) (Midgley, 1996), because of investment in a bud‐bearing organ that does not acquire resources. Moreover, the bud‐bearing organ is usually stem derived, and a plant must modify its growth to deposit stems (and therefore buds) in the soil. While buds may be produced on roots, this ability is restricted to *c*. 10% of plants (according to data from the temperate zone; Bartušková *et al*., 2017).

Seeders, that is plants regenerating from seed after severe disturbance, dominate in ecosystems where their lifespan spans two consecutive disturbances that are very rare (trees) or very frequent (annuals). Resprouters, which may be woody plants as well as herbs, are the most successful under intermediate disturbance frequencies (Bellingham & Sparrow, 2000; Herben *et al*., 2018b). Resprouters often grow clonally by producing adventitious roots on stems or adventitious buds on shoots (see section **VIII. 1. Ability to grow clonally**). Bud‐bank size is positively correlated with rhizome lifespan and negatively with their ability to spread laterally (Klimešová & Herben, 2015). Interestingly, perennial herbs (resprouters) that are able to coexist with annual seeders in habitats subjected to severe and frequent disturbance (e.g. arable land) are characterised by extensive lateral spread, short‐lived connection among ramets, and a bud bank on roots or deeply growing rhizomes (Herben *et al*., 2018b).

Snapshot data about bud banks may be misleading in cases in which bud‐bank size fluctuates during the growing season, which is typical for herbaceous species, especially those having short‐lived below‐ground organs (Klimešová & Klimeš, 2007; Ott & Hartnett, 2012). So far only few studies have quantified the size of the bud bank for individual species (Ott & Hartnett, 2012, 2015). The number of buds in bud banks may also be approximated by the number of leaves on below‐ground stems (Klimešová *et al*., 2017) or by the biomass of bud‐bearing organs (VanderWeide & Hartnett, 2015). However, additional roots buds can be formed after root injury.

#### a. Sampling recommendations

Excavate all below‐ground storage organs according to the procedure described under section **VIII. 1. Ability to grow clonally**.

In herbs with short‐lived below‐ground organs, bud number may fluctuate during the growing season with a minimum after sprouting (seasonal or regenerative) and a maximum number before dormant state. Therefore repeated assessments throughout the season are recommended in seasonal climates (particularly at the time of flowering and at the end of the growing season).

#### b. Storage and processing

Samples should be kept cool and wet while in the field; they can be stored for *c*. 1 wk in a refrigerator.

#### c. Measurement procedure

Count living buds under stereomicroscope, remembering that buds may be hidden under protective tissue. For countable buds on stem‐derived organs, express number of buds per one plant; in the case the plant is clonal (see section **VIII. 1. Ability to grow clonally**) and the excavated bud‐bearing organ has more shoots (i.e. connected ramets), divide the number of buds by the number of ramets. The direct count is not suitable for assessment of bud banks on roots as those buds may be formed only after plant injury. To check the ability of a plant to sprout from roots, plant roots should be collected, fragmented, the size of fragments assessed and allowed to resprout. Three root fragments from each of at least three plant individuals should be cut and placed shallowly into wet sand and allowed to resprout for at least 1 month. Coarse roots are more suitable than fine roots. For each plant, the number of visible buds per root fresh weight (FW) of coarse roots can be assessed, and this number should be divided by the fraction of observed coarse‐root FW per total coarse‐root FW of the plant.

#### d. Future research directions

Despite the critical importance this trait may have for plant functioning, our understanding of bud‐bank size is limited to only a few species, making it difficult to fully assess its ecological relevance across growth forms and biomes. Counting of buds has been mostly performed in the context of grazing on grassy vegetation (Ott *et al*., 2019), while much less has been done in fire‐prone areas or areas subjected to other kinds of disturbance. More generally, it remains largely unknown how disturbance regimes affect plant architecture and bud‐bank building. Furthermore, future research may need to better account for bud longevity, as this may not necessarily be the same as the longevity of the bud‐bearing organs and loss of function with age.

## 10 Root system architecture

The root system architecture (RSA) defines both the shape and the structure of the root system of individual plants. The shape (or spatial configuration, geometry) determines where the roots are in their growing environment, usually the soil. The descriptive traits dedicated to this very important aspect are considered under section **XI. Root spatial distribution**. The structure refers to the diversity of individual roots and root segments within the root system (see section **IV. Below‐ground plant entities and root classifications**), and root system topology, that is, the connections between individual roots. As the root system grows its shape and structure are continuously altered, so that the development of RSA is an important aspect of plant ontology (see sections **XVI. Root dynamics** and **XXI. Root tip morphology and elongation**).

Many works have shown the central role of the RSA to fulfil the various plant functions and ecosystem services performed by the root systems. It is particularly obvious for the soil–plant exchanges (water, nutrients, organic compounds; e.g. Larigauderie & Richards, 1994), but also for the within‐root transport of several resources and signalling molecules (e.g. Doussan *et al*., 1998 for water transport; Jung & McCouch, 2013 for hormones), as well as for the mechanical roles of roots (plant anchorage, soil protection against erosion and landslide; for example Stokes *et al*., 1996, 2009). Plant requirements change over time and consequently the root system must adapt dynamically. In addition, roots develop in a medium (usually a soil) in which the physical, chemical and biological conditions vary in space and time. Root functioning is thereby an interplay between a dynamic growing environment and a dynamic root system. For example, the uptake of nonmobile and scarce nutrients, such as phosphorus in unfertile soils, is highly dependent on root branching and elongation that allows the continuous colonisation of new, nondepleted sites (Robinson, 1996a). Another example is given by root thickening through secondary (radial) root growth that increases root mechanical strength and transport capacities while incorporating substantial amounts of assimilated carbon (Stokes *et al*., 2009). As the RSA with its dynamics is particularly complex, it is helpful to take a particular viewpoint, such as a limited set of target root functions or ecosystem properties, and to find out the suitable traits to improve our understanding of these functions. In addition, monitoring environmental conditions may be important as roots are highly plastic.

Much of the scientific understanding of the functional and ecological relevance of various traits is founded on theoretical models of root systems rather than direct experimental evidence. This is largely because many traits are directly or indirectly interconnected and cannot be considered individually, and also because trait values are highly dynamic. During the last 3 decades, various approaches aiming at the study of the dynamic RSA led to the development of detailed RSA models which address these issues (Dunbabin *et al*., 2013). The general principle of these models is to mimic and combine several *developmental processes*, applied to individual roots that are usually grouped into categories corresponding either to developmental branching orders or to other predefined types (see section **IV. Below‐ground plant entities and root classifications**). In most of these simulation models, a set of input parameters is defined for each root type to quantify their development. This includes descriptors of: (1) root emergence from the embryo and the shoot system (e.g. number of seminal roots and shoot‐borne roots), (2) root elongation and direction (e.g. root elongation rate, root growth angles (RGA)), (3) root acropetal branching (e.g. root branching density (RBD)), and (4) secondary growth that modifies the initial (primary) root diameter. Such traits are used both to calibrate RSA models and to further analyse their effect on root system shape, structure and functioning. RSA models are thereby tools for bridging data from the root developmental traits to the traits measured on whole root system in populations (Postma & Lynch, 2012; Pagès & Picon‐Cochard, 2014). For example, spatial traits such as the distribution of RLD or rooting depth can be estimated using RSA models (Pagès *et al*., 2012; Hecht *et al*., 2018). In addition to connecting scales, the RSA models have been used to estimate how RSA influences root functioning, including nutrient and water uptake and anchorage (Dunbabin *et al*., 2013). Plant phenotyping is currently being used to validate these model predictions (i.e. Saengwilai *et al*., 2014; Zhan & Lynch, 2015; Jia *et al*., 2018).

Root system architecture is typically described by several traits as there are no conclusive sets of traits that will capture every aspect of RSA. These traits can be divided into two categories: (1) traits that describe shape and structure of the whole‐plant RSA, or even multiple plant stands; and (2) traits that describe the developmental processes of RSA. Descriptors of the whole‐plant RSA include traits such as RLD (see section **XI. 1. Vertical root distribution**), specific root length (SRL; see section **XII. 2. Specific root length**), median rooting depth, maximum rooting depth (see section **XI. 2. Maximum rooting depth**), convex hull and fractal geometries (Fitter & Stickland, 1992; Bingham & Stevenson, 1993; Galkovskyi *et al*., 2012). The list of published descriptors is long and many descriptors have been used in very specific contexts only. As several of these whole‐plant descriptors are described elsewhere in this work, we focus here on the type of root systems only. Regarding traits that describe the developmental aspects of the RSA, many root developmental traits have also been proposed. These range from the cellular level to more epiphenomenal traits such as RBD and RGA. Many of the developmental traits require detailed observation of growth dynamics of individual plants, something often difficult to achieve in an ecological context. We focus here on RBD and RGA as they are much studied traits. RGA is considered an important trait for the higher‐order roots (major axes), which typically are the longer roots that influence the spread of the root system in horizontal and/or vertical directions. Most of the root length, however, is formed by smaller lateral roots and the later RBD is an important trait influencing root length locally. Like RGA, RBD can be determined based on partial observations of the root system, and has been much studied.

### 1. Types of root systems

In addition to classifications schemes that are used to accurately describe the multiple parts that compose root systems (already described under section **IV. Below‐ground plant entities and root classifications**), classifications of the entire root system also exist. These attempt to synthetise some architectural aspects of entire root systems using simple descriptors. Although we note that the links between such classifications and root ecology and functioning are not always clearly assessed, we give here an overview of the most common of such classifications systems.

#### Common morphological classification

Root systems are commonly classified into fasciculate (synonymous: diffuse, fibrous) and tap rooted (synonymous: central, conical) root systems. Fasciculated root systems are distinguished from tap rooted root systems by the number of major axis directly coming from the stem (including hypocotyl and mesocotyl and stolons). Fasciculated root systems have many equally dominant roots, whereas tap rooted root systems have only one dominant primary (or tap) root. This qualitative classification is attractive from an operational viewpoint because it is simple and based on the morphology of the proximal part of the root system (root crown), which is usually the easiest to extract from the soil. This classification, however, is too coarse and additional classification criteria are necessary to represent the large diversity in morphologies that can be observed. For example, the root systems of monocots all belong to the first category (fasciculate). Fasciculated root systems can be further distinguished according to the level of aggregation of the main roots inserted on the shoot system. Therefore, the root system can be clearly caespitose (dense) when aggregation is high, or diffuse when the main roots are inserted at larger distances from each other (e.g. along a rhizome) (Weaver, 1958). Among gymnosperms and dicotyledonous species, there are a large number of cases with intermediate root types, which do have a dominant taproot with several less dominant major roots (e.g. Dunbabin *et al*., 2003). Many plant species have a prominent taproot when young, which loses its dominance at later stages. Several environmental factors, among them soil depth or water tables, can also modify the hierarchy among roots. We are not aware of any quantitative approach that defined measurable traits to describe this diversity and can thereby only caution against careless use of this morphological classification.

#### Classification based on the ‘set‐up strategy’

Cannon (1949) suggested an alternative classification, based on the developmental sequence (or ‘set‐up strategy’). This classification defines two fundamental categories: (1) the primary (or seminal) root system originating from the seed (comprising the radicle that develops into a primary root and basal roots originating from the scutellar node), and (2) the secondary root system (also called adventitious or shoot‐borne root system) originating from the shoot system. Both seminal and shoot‐borne root systems produce lateral branches to eventually give rise to the entire root system. Many plant species can have both seminal and shoot‐borne roots, but are typically dominated by one or the other. Juvenile plants growing from seed are typically dominated by primary root systems, whereas the secondary root system develops later. By contrast, plants growing from cuttings or stolons have only a secondary root system.

Cannon (1949) defined six subtypes among the primary root systems, according to the length of the taproot in comparison to the laterals, and to the distribution of these laterals along the taproot (more or less superficial or evenly distributed). Among the secondary root systems, he distinguished four subtypes according to the level of similarity of shoot‐borne roots (‘uniformal’ vs ‘multiformal’) and to the closeness of their origin (caespitose vs diffuse).

**Fig. 14:**
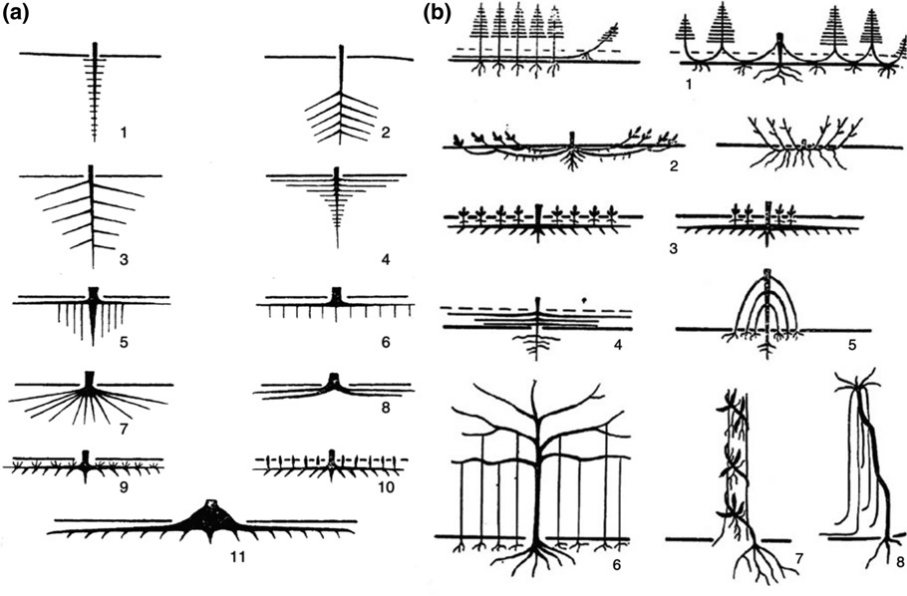
Classification of root systems for trees and shrubs, according to Krasilnikov (1968). (a) Primary root systems, with 11 different types; (b) secondary and mixed root systems, with eight different types.

A similar classification dedicated to tree species has been developed later by Krasilnikov (1968) and Kahn (1977). Krasilnikov defined 11 different morphological types among the primary root systems, and eight among the secondary and mixed root systems (Fig. 14). Mixed root systems were defined as root systems in which the primary and secondary root systems coexist and remain both important.

As for morphological classification, we are not aware of attempts to determine the set‐up classification based on measurable quantitative traits. However, the set‐up classification is based on root biology and is less dependent on environmental conditions and thereby possibly more useful in ecological studies. For example Cannon discussed the significance of the various types regarding the water ecology of plants. He suggested that the long taproot may favour the access to deep resources, while the superficial laterals allow the capture of shallow penetrating rainfalls.

#### Classification based on multiple morphological and functional criteria

Bodner *et al*. (2013) used a large array of traits measured at several scales to classify root systems. They used traits associated with whole: (1) plant growth, (2) biomass allocation, (3) root system shape, and (4) root axes morphology, branching, anatomy and physiology. These traits were chosen for their developmental and/or functional significance. Using statistical procedures, Bodner *et al*. (2013) could classify root systems into functional groups which can be objectively calculated. As this method extends the classification criteria, it also requires many traits to be measured. But it is potentially an interesting way of synthesising data and sorting out the global root system diversity.

#### Integrating particular adaptations of roots

Special adaptations of individual roots can strongly influence the whole RSA. For example buttress and stilt roots are special adaptations that improve anchorage or support; cluster roots are dense clusters of laterals that improve phosphorus uptake on soils with very low phosphorus availability; aerating roots (or pneumatophores) rise above ground and provide oxygen; tuberous roots are storage organs; clonal roots transfer resources across plant ramets; and haustorial roots absorb water and nutrients from another plant. These roots that are characterised by their functional adaptations could be included in future root system classifications. They could complement the data‐defined approach proposed by Bodner *et al*. (2013) or the set‐up classification.

### 2. Root growth angles

*Root growth angle* is the smallest angle between the direction the root is growing and the horizontal plane (typical units: degrees) (frequent abbreviation: RGA). Roots growing upward are given negative angle values.

While most researchers measure absolute angles (relative to horizontal), the literature needs to be read with care as many alternative terms and definitions exist (e.g. Fitter, 1987), including angles from vertical and tangents (e.g. root branching angle, insertion angle, gravitropic set‐point angle). Furthermore, due to tropisms, the initial branching angle close to the base of the root is not the same as the gravitropic set‐point angle (Digby & Firn, 1995), further away from the root.

RGA is typically measured on main root axes and has been associated with shallow and deep foraging strategies, but also with degree of inter‐root and intra‐root competition (Ge *et al*., 2000). RGA is under genetic control (e.g. Liao *et al*., 2004; Omori & Mano, 2007; Wang *et al*., 2018), as illustrated by the rice genotype Deeper Rooting 1 (DRO1) which displayed higher RGA together with greater drought tolerance (Uga *et al*., 2013; Kitomi *et al*., 2015). RGA are independent of size, and can be determined on relatively young plants, as they do not change over time, assuming roots do not move significantly in soil. Nonetheless, RGAs of shoot‐borne roots can differ with increasing stem node number in maize and rice, which often are formed sequentially (Araki *et al*., 2000). RGA is not only influenced by species and genotype, but also by the environment. Low phosphorus conditions have been reported to decrease RGA, whereas RGA increased in low N soils (Bonser *et al*., 1996; Trachsel *et al*., 2013).

RGA has been repeatedly found to correlate to root vertical distribution. For shallower layers this correlation is negative, whereas for deeper layers the correlation is positive, that is plants that have low RGA tend to have relatively more roots (e.g. higher RLD) in the topsoil (Oyanagi *et al*., 1993; Oyanagi, 1994; Trachsel *et al*., 2013). This relative distribution has also been quantified using mean and median rooting depth, which both correlate positively with RGAs (Trachsel *et al*., 2013). Shallow RGAs have been associated with increased uptake of phosphorus whereas deeper RGAs have been associated with increased uptake of nitrate and water, although the associations depend on environmental conditions, that is the stratification and replenishment of the resource (Lynch & Brown, 2001; McCulley *et al*., 2004; Lynch, 2013). Given the genetic variation in RGAs, its consistent plastic responses and their positive impact on nutrient uptake, RGA is an important functional trait. Nonetheless, most evidence comes so far from agronomic crops grown on tilled field soils, often with relatively deep profiles. The relevance of RGA in an ecological context, across species, remains to be linked with the extensive ecological literature on rooting depth.

#### a. Sampling recommendations

Angles are mostly determined for main root axes such as basal, and shoot‐borne roots and since these axes often are often relatively thick and sturdy, angles may be determined based on root crown excavation (see section **VI. 3. a. Overview of root sampling methods**; Trachsel *et al*., 2011). Hecht *et al*. (2018) estimated branching angles of the shoot‐borne roots of barley in the field by comparing the root length distribution of thick roots of cores in and in between the rows (see section **XI. Root spatial distribution**). Angles can also be determined by nondestructive imaging of roots (see section **VI. 3. b. Overview of root observation methods**; Bonser *et al*., 1996). Hereby special care should be taken in 2D systems, such as paper pouches and root boxes and windows, where the growth direction of the roots is deflected by the hard surface. This deflection often causes roots to grow down. Using maximum, instead of average angles, or only measuring angles of roots that do not grow against the window or pouch surface but parallel to it, might be a better sampling strategy.

#### b. Storage and processing

Angles are lost during storage and processing, and thereby should be measured or imaged directly after sampling.

#### c. Measurement procedure

Root growth angle is measured for individual roots, and for the whole plant the average angle may be reported, with possibly distinguishing root classes. Recent modelling studies have suggested that not only the average but especially the within‐root system variation in RGA might be of importance for nitrate uptake (Dathe *et al*., 2016). RGA is easily determined by placing the root system or root crown on a board with drawn angles (Trachsel *et al*., 2011). Alternatively, they can be determined using image analysis software, after taking a picture using a tripod or any type of fixed structure setting the camera lens parallel to the root plane (York *et al*., 2018). Software developed for root crown evaluation are DIRT and REST (Colombi *et al*., 2015; Das *et al*., 2015) whereas SmartRoot is an example of a software that can be used on 2D paper pouch images (Lobet *et al*., 2011). More recent options include the RhizoVision Crown hardware platform, which combines a back light, a monochrome camera, and a clip‐and‐replace system for high‐throughput and reproducible imaging of root crowns (Seethepalli *et al*., 2020), while the RhizoVision Explorer software computes angles for all skeleton pixels in the ‘Whole root’ mode with average orientation and the frequencies of shallow, medium and steep angles provided. The generic image analysis ImageJ program (http://rsbweb.nih.gov/ij/) also contains a tool for measuring angles. Angles in 3D carved images can be determined using RootReader3D (Clark *et al*., 2011).

#### d. Future research directions

As RGA is an important component of root foraging strategies and influences the degree of root growth towards neighbouring plants, RGA measurements may be a promising tool for studying root interactions within and across species. Within pots, the use of magnetic resonance imaging and X‐ray microtomography technologies have shown great potential for studying the coarser part of RSA (Metzner *et al*., 2015; van Dusschoten *et al*., 2016), and may be readily applied to the study of RGA, providing sufficiently large containers are used. In the field, GPR can detect coarse roots (> 1 cm) noninvasively but the RGA influences the signal, which poses a measurement challenge for interpreting the data (Tanikawa *et al*., 2013). The technique, however, is improving and, recently, attempts to determine root weight of wheat have shown promising results (X. W. Liu *et al*., 2018), suggesting future potential application for RGA measurements.

### 3. Root branching density

*Root branching density* is the number of laterals on a given length unit of parent root (typical units: mm^−1^ or cm^−1^) (frequent abbreviation: root branching density (RBD)). Also referred to as root branching intensity (RBI).

*Root interbranch distance* is the length along the parent root that separates neighbouring laterals (typical units: mm) (frequent abbreviation: root interbranch distance (IBD)). It corresponds to the length of internal links *sensu* Fitter (1987).

Typically, IBD represents the inverse of RBD. However, some authors approximate RBI by counting the number of root tips of multiple roots simultaneously (e.g. per length of the first two order roots, following the morphometric classification; Comas & Eissenstat, 2009), thereby aggregating information on root branching from several root orders. Measuring RBI in such a way does not yield comparable values with IBD values or RBD values measured on one single root.

Root branching density has a strong impact on the overall number of roots and their distribution over the soil profile and shows substantial interspecific variation (Pagès & Kervella, 2018). Structured variation among roots within the whole root system has also been reported (Pagès, 2014; Postma *et al*., 2014; Wu *et al*., 2016). However, in heterogeneous soils, their regulation under the influence of exogenous factors has often been recognised as a major way for the plant to locally adapt the root density to resource availability (Forde & Lorenzo, 2001; Hodge, 2004, 2009). At both the interspecies and intraspecies levels, lower RBD represents a tendency of root systems to explore larger volumes of soil rather than intensifying their search in localised soil compartments (Wiersum, 1958; Eissenstat *et al*., 2015). Root branching is highly plastic and sensitive to the heterogeneity of nutrient availability in the soil, especially to nitrogen in poor soils (Fitter & Stickland, 1991; Larigauderie & Richards, 1994; Freschet *et al*., 2018). Some of these studies indicate nonetheless that such trends may be strongly species‐specific with differences among plant functional types, for example between forb and graminoid species.

#### a. Sampling recommendations

See section **VI. Experimentation and sampling in laboratory and field**.

#### b. Storage and processing

See section **VII. Root washing, sorting and storage**.

#### c. Measurement procedure

Since RBD is sensitive to both the local soil conditions and to the parent root type, its measurements should take into account these two factors and clearly specify the sampling strategy to increase their relevance and allow valuable comparisons (Dubrovsky & Forde, 2012). The precise sampling design will depend on the particular objective that can be the study of the effect of soil heterogeneity, or the comparison of species (or genotypes) in similar conditions. Typically, RBD is measured on the rather young, but fully branched, part of the considered roots, keeping in proximal position from the distal bare zone, that is by measuring the number of first‐order root branching on second‐order roots following the morphometric classification. When RBD is measured in older, more proximal parts (e.g. higher root orders following the morphometric classification), its value may be altered by root decay and self‐pruning.

Root branching measurements most often capture ‘acropetal branching’ because branching usually occurs in an organised schema, with young lateral roots sprouting preferentially following the parent root apex. However, in some situations, branching can occur in a more diffuse pattern along the parent root, especially after perturbations inducing the cessation of growth of the parent root. Such situations remain much less frequent than the very general process of acropetal branching and may need to be treated separately, as they do not yield comparable values of root branching to measurements made on nonsevered or younger roots.

Root branching density is conveniently measured on images of roots made on rhizotrons or from scanned excavated roots. When images come from rhizotron windows (see section **VI. 3. b. Overview of root observation methods**), it is necessary to check that all lateral roots are actually visible, as lateral roots usually do not have strong gravitropism and may grow at the back side of the parent root, away from the window into the soil. Branching density (or the reciprocal IBD) is usually obtained by counting the lateral roots along the length of parent root from the basis of the axis to the last lateral branch (i.e. excluding the root segment after the last branching). Including the distal bare zone is a frequent error (as discussed by Dubrovsky & Forde, 2012). If sampling prohibits collecting the whole root, for example when using root cores, a shorter section (2–5 cm) can also be used. This can be done directly on excavated roots or on images (for obtaining scanned images see section **XII. 2. Specific root length**). Care needs be taken not to forget the broken roots that normally leave traces of their existence on the parent root, at least in the rather young parts. The root length is typically measured using a ruler or an image analysis software such as ImageJ software, using the ‘segmented line’ tool. The variations in the branching density trait, when estimated by counting laterals on root parts, are potentially sensitive to the length of the root parts on which they are performed. Therefore, we advise to repeat these measurements on a range of parent roots within one sample (typically 5–10 depending on the size of the root sample) because this variable usually exhibits very large random variations (Pagès, 2014).

## 11 Root spatial distribution

Root distribution refers to the horizontal and vertical extent of soil from which plants can interact with the soil matrix (Jackson *et al*., 1996; Robinson, 1996b) and bedrock (Dawson *et al*., 2020). Root distribution determines the below‐ground ‘zone of influence’ of plants within the ‘critical zone’, whether with respect to the plant influence on soil functioning (Freschet *et al*., 2021), or acquisition of soil resources (Casper *et al*., 2003; Bartelheimer *et al*., 2006; Klimešová *et al*., 2018a).

In plant competition studies, little or no overlap of root distribution among different species can be considered as an indication of below‐ground resource partitioning and niche segregation (Mueller *et al*., 2013; Ravenek *et al*., 2014) or root territoriality in response to neighbours (Novoplansky, 2009; Cahill & McNickle, 2011). The distribution of roots, whether homogeneous, heterogeneous, sparse or dense, plays a part in determining the extent of water and nutrient acquisition by roots and their associated symbiotic communities (Jumpponen *et al*., 2010; Mommer *et al*., 2018). Similarly the distribution of roots in the soil profile influences carbon inputs and the priming of soil microbial communities through root exudation and decay (Kuzyakov, 2010; Cheng *et al*., 2014; Lange *et al*., 2015).

Root distribution is a function of species, plant development, symbiotic associations and soil characteristics. For example, some plant species, even within plant functional groups, inherently root deeper than others (Kutschera *et al*., 1992; Kutschera & Lichtenegger, 2002; Comas *et al*., 2013; Schroeder‐Georgi *et al*., 2016). However, as plants grow and develop, their root system generally increases in absolute size, which can affect root distribution. Therefore, measurements need to carefully consider the developmental stage of individuals in interspecific studies to reduce confounding influences of plant size and ontogeny on root allocation (Poorter *et al*., 2012b). Moreover, plants are plastic and will adjust their root systems to soil conditions, such as nutrient and water availability (Fransen *et al*., 1998; Hutchings *et al*., 2003). For example, nutrient limitations may encourage foraging for nutrients, which may lead to altered root distributions. In addition, if nutrients are distributed patchily, roots will distribute unevenly (Hutchings & De Kroon, 1994; Hodge, 2004). Similar plastic responses have been described for water shortage. In arid and semiarid climates where the soil dries at the surface and water is relatively more available deeper in the profile as the season progresses, one strategy of plants to deal with drought is to allocate a greater proportion of their roots to deeper layers (Wasson *et al*., 2012; Comas *et al*., 2013). Alternative strategies of extreme tolerance to water deficiency may include xylem resistance to hydraulic failure or drought deciduousness (Gleason *et al*., 2016), but fall beyond the scope of the aim of this chapter. Dense soil layers or superficial groundwater levels may also limit the vertical extent of root distributions (Fan *et al*., 2017).

The oldest and potentially simplest way to investigate root distribution is likely to be the spade. Digging large trenches to quantify root encounters and plot them on vertical and horizontal axes is possible, but destructive, and typically restricted to a low number of replications (Parrish & Bazzaz, 1976; Dannowski & Block, 2005). By comparison, coring the soil and collecting roots from these soil samples is less destructive but needs a fair number of samples to account for vertical and horizontal spatial variability. It is a basic, but robust technique to get reliable measures of root distributions.

Measuring root distributions is a matter of handling large numbers of samples to cover inherent spatial variation in a plot. Taking many root cores to determine vertical or horizontal root distributions requires decent planning for root washing, as this is always a very time‐consuming step. The procedures for measuring root distributions will therefore be a compromise between precision and available time. There is a trade‐off between time spent per sample and the number of samples that can be processed. Alternatively, minirhizotron tubes can be used to examine root distribution. When taking minirhizotron images to estimate root distribution, the time‐consuming step of root washing will be avoided, but taking and analysing the root images will easily cover that ‘free’ time. There are, however, similar trade‐offs as with soil cores in that there is a greater advantage in installing more minirhizotron tubes than in processing more images in a single tube.

In principle, sequential soil coring can also be used to assess broad temporal patterns in root distribution (Fargione & Tilman, 2005), but is not recommended for studies in which a fine temporal resolution is needed. To study seasonality of root distributions a minirhizotron approach is preferred (Eissenstat & Yanai, 1997; Chen & Brassard, 2013). Minirhizotrons mitigate the problem of spatial variability between consecutive soil cores by repeatedly sampling the same patch of soil over time. In the near future, ground penetrating radar (GPR) might provide a useful alternative for investigations of root distribution (Smithwick *et al*., 2014). GPR has been used in the past to detect and map plant roots nondestructively where different radar frequencies are used to penetrate the soil to various depths (Wu *et al*., 2014). To date, this technology has been verified to detect coarse roots down to 1 cm in diameter (Hruska *et al*., 1999) but advances in the technology may allow for fine root detection in the future (X. W. Liu *et al*., 2018).

### 1. Vertical root distribution

Vertical root distribution is the distribution of roots in units of biomass or length over sequential soil layers ranging from the soil surface to deeper horizons (e.g. Persson & Stadenberg, 2009; Mueller *et al*., 2013; Ravenek *et al*., 2014; Weemstra *et al*., 2017). The patterns of root distribution can be analysed directly by assessing either root length or mass density measurements:

*Root length density* is the length of roots per unit soil volume (typical units: m cm^−3^) (frequent abbreviation: root length density (RLD)).

*Root mass density* is the mass of roots per unit soil volume (typical units: g cm^−3^) (frequent abbreviation: root mass density (RMD)).

Alternatively, it can be expressed as the percentage of total root mass or length per layer, also referred to as ‘Cumulative root biomass‐or‐length fraction or curves’ (Comas *et al*., 2005). Several traits that are more integrative of the pattern of root distribution can also be derived, including:

*Vertical root mass distribution index* is the extinction coefficient (β) of an asymptotic equation (*Y* = 1 − β^d^) fitting the cumulative proportional root biomass over depth (no units). It represents the steepness of the exponential decline of root mass over soil depth (Gale & Grigal, 1987; Jackson *et al*., 1996). Values of β approaching 1 imply a greater proportion of roots with depth, while lower values represent a greater proportion of roots near the soil surface. This index can also be expressed per unit root length.

*Mean rooting depth* is the rooting depth at which half of the root mass occurs above (typical units: cm) (frequent abbreviation: mean rooting depth (MRD)).

Often, more than 70% of root biomass is found in the upper 30 cm of the soil (Jackson *et al*., 1996). The density of root biomass, root length and/or surface area in soil facilitates plant influence on soil biotic and abiotic properties (Bardgett *et al*., 2014), leading to major changes of soil chemistry (Mueller *et al*., 2012; Lange *et al*., 2015). Trade‐offs between root morphology and RLD seem to indicate that species with greater SRL, root length per unit biomass) or thinner roots are able to produce greater RLD for increased soil exploration, interactions with soil biota and higher enzymatic production (Comas *et al*., 2012; Weemstra *et al*., 2017; Lugli *et al*., 2020).

In soil functions, RLD and RMD are important components of soil carbon stocks with organic matter contributions to the soil food web coming from root death as well as exudation (De Deyn *et al*., 2008; Mommer *et al*., 2015). In grassland biodiversity experiments, increased root biomass in mixtures compared with monocultures drives increased microbial biomass and carbon stocks in diverse grasslands (Fornara & Tilman, 2008; Cong *et al*., 2014; Lange *et al*., 2015). As carbon fuels the mineralisation process in the soil, greater carbon stocks in soil are often correlated to faster mineralisation rates (Fornara & Tilman, 2008; Cong *et al*., 2014). In addition, plant communities with a greater RLD and RMD have reduced leaching of nitrogen and other nutrients (Thorup‐Kristensen *et al*., 2003). While RLD and RMD have clear effects on soil carbon and soil nutrients, the size of the effects will depend on species (Guo *et al*., 2005; Richter *et al*., 2015; Chen *et al*., 2017), and climatic conditions (Maeght *et al*., 2015).

RLD also increases the structural stability of soil in different ways (Gould *et al*., 2016). Greater RLD and RMD increase soil porosity, infiltration and water movement through soil (Fischer *et al*., 2015, 2019; Wright *et al*., 2017). Additionally, increased root exudates associated with greater RLD and RMD can increase soil aggregate adhesion (Morel *et al*., 1991; Czarnes *et al*., 2000; Hallett *et al*., 2009). According to Hallett *et al*. (2009), plant roots have bigger effects on soil stability than the fungal communities they support. Finally, increased RLD and MRD decreases soil erosion by physically holding soil in place (Gyssels *et al*., 2005; Stokes *et al*., 2009; Loades *et al*., 2010; Berendse *et al*., 2015).

When research questions pertain to soil exploration for certain activities involving the entire root system, such as plant anchorage, the vertical distribution of the entire root system (coarse and fine roots) may be most informative. If research questions centre on areas of soil where root physiological activity is concentrated, then root length and biomass measurements of the fine‐root system alone may be preferred.

#### a. Sampling recommendations

If spatial aspects of root distribution are the key interest of research, root coring may be the simplest method to use. However, if there are also temporal aspects to the research questions posed, minirhizotrons are the preferred method.

##### Root coring

The best way to take a root core depends on the research question and type of experimental set‐up, being pot or field study; see section **VI. 3. a. Overview of root sampling methods**.

In the case of a pot study in which plants are growing for a relatively short time, the easiest method of harvesting the soil section of interest is using a relatively sharp knife. For example, remove the pot and cut the entire soil volume including roots into different depth slices or different quadrants. If the research question requires deeper pots, customised PVC pipes can be halved and re‐fixed with tape to ease soil extraction from pots before cutting (see e.g. Schroeder‐Georgi *et al*., 2016). Because root biomass/length often tends to increase towards the sides of the pots, we prefer the method of cutting entire sections rather than taking a core through the pot.

Taking root samples in mesocosms, common‐garden experiments or from the field typically requires the use of root augers. See section **VI. 3. a. Overview of root sampling methods** for further details. If the soil core is removed in ‘one go’, use a measuring stick (or the markings on the soil corer) to mark the soil layers and cut the soil profile with a knife, starting from the top layer to the bottom layer.

A typical soil profile in grassland could be: 0–5; 5–10; 10–20; 20–30; 30–40; or 40–50 cm; but other studies have used every 15 cm down to 1.5 m for crops. If a distinct organic topsoil layer is clearly distinguishable from deeper mineral layers it is advised to separate the profile accordingly. If the samples have a high RLD, depth increments are reduced to reduce the amount of roots in the sample and make it more manageable for washing. Woody plants often have less RLD in soil so depth increments in forests may be a bit longer to save on the number of samples that needs processing.

#### b. Storage and processing

##### Root coring

See section **VII. Root washing, sorting and storage**.

#### c. Measurement procedure

##### Root coring

For mass‐based measurements, the clean root biomass is oven dried at 60°C for 48 h and weighed. For length‐based measurements, the clean root length is assessed as described under section **XIII. 2. Specific root length**. Regarding the measurement of root length or mass density, soil volume is obtained from calculations of the internal volume of the corer rather than the soil core itself, which may have been compacted during sampling.

##### Minirhizotrons

Minirhizotrons allow continuous, nondestructive measurements of vertical and temporal root distribution once they are installed in a plot or mesocosm (for installation and use procedures, see section **VI. 3. b. Overview of root observation methods** **)**. When assessing the presence and distribution of roots the frequency of image collection is an important factor to consider, especially in relation to the length of root lifespan anticipated. Intervals of image collection greater than the median root lifespan can result in underestimates of root populations, as roots can appear, die and disappear between imaging times and would be missed in assessments of distribution. Conversely, very frequent intervals increase the workload for image collection and analysis. Collecting images every 2 wk is suggested for many species, at least during the growing season when roots may be actively growing and dying (Comas *et al*., 2000; D. Johnson *et al*., 2001). Monthly image collection can be adequate during the dormant season when changes occur more slowly.

##### Calculations

From these measurements, MRD is calculated as the sum of the root mass per soil layer multiplied by the mean depth of each layer divided by the total mass of roots in all layers (e.g. see Dimitrakopoulos & Schmid, 2004; Mommer *et al*., 2010; Ravenek *et al*., 2014). The vertical root distribution index (β) can be determined by fitting an asymptotic equation to the cumulative proportional root biomass over depth (Gale & Grigal, 1987):

$$Y=1‐\beta^{d}$$

where *Y* is the sum of all root mass samples from the surface to depth *d* (cm); β is the fitted ‘extinction coefficient’ of the root biomass. High values of β indicate that the community has a greater proportion of the roots at deeper depth. Low values indicate that the roots are positioned near the soil surface. Values typically vary between 0.91 and 0.98 (Jackson *et al*., 1996).

##### Species‐specific root distribution

Root biomass in field studies is most often measured at the level of the whole plant community, as roots of different species are difficult to distinguish morphologically. This lack of morphological differences has hampered identification of root biomass at the species level. However, several studies with a few distinct species have taken effort to separate different root systems by hand (Janeček *et al*., 2004; Mommer *et al*., 2011; Hendriks *et al*., 2013). However, new techniques are now available to identify roots of different species in species‐rich plant communities without manual sorting, as described under **VII. 4. Separating roots by species**.

#### d. Future research directions

Vertical root distribution is an important indicator of plant resource uptake strategy and below‐ground biotic interactions, yet we know little about its plasticity in time and space and how this vertical distribution depends on both biotic and abiotic factors. Studies on root competition or facilitation are mostly limited to pair‐wise experiments in artificial settings, while quantitative data on changes in vertical root distribution with changes in plant community composition or increasing soil temperature are scarce, as is data due to changes in seasonality or diversity loss. Vertical root distribution is challenging to assess in mixed communities, unless species have distinct root characteristics. Nonetheless, species percentages can be determined from genetic analyses of root samples collected from cores (see section **VII. 4. Separating roots by species**).

### 2. Maximum rooting depth

*Maximum rooting depth* is the maximum soil depth at which roots occur (typical units: m).

Maximum rooting depth is a specific but critical aspect of vertical root distribution. Greater maximum rooting depth is generally thought to reflect the importance of water acquisition for the plant. Deep maximum rooting depths have been found to be advantageous to plant species growing under limited soil water availability in both natural and agricultural systems (Schenk & Jackson, 2002; Ho *et al*., 2005; Schenk & Jackson, 2005; Hund *et al*., 2009; Lopes & Reynolds, 2010; but see Sun & Mao, 2011). The maximum rooting depth can be similar or greater than the maximum shoot height and is strongly dependent on ecosystem type and plant growth form (Canadell *et al*., 1996; Jackson *et al*., 1996; Schenk & Jackson, 2002). Knowledge of a plant’s maximum rooting depth can be important for understanding plant ecosystem functions with a relatively small number of roots deep in the soil profile indicating critical water uptake and/or complex hydraulic lift of water from deeper soil layers (Caldwell *et al*., 1998; Jackson *et al*., 1999). A few roots at depth may be disproportionately relevant for water uptake under drought conditions. Species variation in maximum rooting depth is enormous, ranging from 0.3 to > 70 m (Fan *et al*., 2017). In ecosystems where water is available deep in the soil profile or in riparian zones, many successfully adapted species avoid drought by producing deep roots that can access this water (Abrams, 1990; Comas *et al*., 2013). However, while maximum rooting depth has explained important functional differences among woody plants (Jackson *et al*., 1999) and some herbaceous plants (Ho *et al*., 2005), it seems to be less predictive for other herbaceous plants (Awad *et al*., 2018). Jackson *et al*. (1999) showed that tree roots of *Quercus fusiformis* between 10 and 25 m depths were able to access groundwater and use hydraulic lift to support themselves and other plants in their vicinity. Recently, it has been demonstrated, using observations and modelling that hydrology is a predictor of global patterns of plant maximum rooting depth (Fan *et al*., 2017). Nevertheless, in water‐limited grasslands and savanna ecosystems shifts in root distribution to surface soils without changes in maximum depth appear equally effective to increase plant performance (Nippert & Holdo, 2015).

#### a. Sampling and measurement procedure

Measurements to quantify maximum rooting depth are among the hardest to execute in root ecology if rooting depth is exceptionally deep. To confirm maximum rooting depth, soil layers below the root zone need to be sampled. The maximum root depth is determined on the entire root system, but is often recorded as the depth of a trench, road cut, mine pit, cave, or other excavation (Canadell *et al*., 1996). The methodology to measure maximum rooting depth is determined by the available options to take cores or make trenches and pits, manually or with machines such as hydraulic corers mentioned above or soil excavators (see also section **VI. 3. a. Overview of root sampling methods** **)**.

An alternative method can be to infer maximum rooting depth from the results of isotopic tracer studies using natural abundance of δ ^18^O and a gradient in isotopic composition of water in the soil. As no isotopic fractionation occurs during soil water uptake by root systems, xylem water is an indicator of mean depths of water uptake. However, this technique is more readily used to differentiate different water sources (precipitation at different times, groundwater) and broad distinctions of water use rather than maximum rooting depth (Ehleringer & Dawson, 1992; Leroux *et al*., 1995; Ludwig *et al*., 2004; Kulmatiski *et al*., 2006; Hoekstra *et al*., 2014). Alternative techniques inject deuterated water (D_2_O) or other tracers (Currie & Hammer, 1979) to different depths to quantify uptake, yet mostly not deeper than 1.2 m (Kulmatiski *et al*., 2010 and references therein). More recently, global synthesis data on maximum rooting depth was linked to topography and soil hydrology to estimate water‐uptake depths using inverse modelling (Fan *et al*., 2017). However, even though the resulting patterns of rooting depths revealed a strong topographic and hydrological signature at the landscape scale, the use of inverse modelling to estimate local and species‐specific maximum rooting depths has yet to be demonstrated.

#### b. Future research directions

Direct measurements of maximum rooting depth are time consuming and destructive, whereas indirect measures are limited in precision, especially at deeper soil depths. While general linkages may exist between maximum rooting depth and plant above‐ground characteristics at the community level (e.g. Schenk & Jackson, 2002), such relationships are unlikely to hold at the species level. Nonetheless, measurements of maximum rooting depth remain tractable and relevant in many plant communities with relatively shallow rooting.

### 3. Root horizontal distribution

Horizontal root distribution is the distribution of roots in units of biomass, length or root counts over sequential sections from the base of the plant outwards. Similar to root vertical distribution, this can be analysed directly as the amount or length of roots per section or the percentage of total roots per layer, also referred to as ‘Cumulative root biomass‐or‐length fraction or curves’ (Comas *et al*., 2005). From the pattern of root distribution, the ‘evenness’ of root lateral distribution can be assessed, and several traits can be derived, including:

*Lateral root mass distribution index* is the extinction coefficient (β) of an asymptotic equation (*Y* = 1 − β^d^) fitting the cumulative proportional root biomass over the distance to the plant base (no units). Values of β approaching 1 correspond to a greater proportion of roots away from the plant base, while lower values illustrate a greater proportion of roots near the plant base. This index can also be expressed per unit root length.

*Lateral rooting extent* is the maximum distance between superficial roots and the base of the plant (typical units: m).

It is generally assumed that lateral roots spread further than their above‐ground counterparts, although evidence on lateral root distribution is scarce due to the hidden nature of roots. Lateral distribution is an important factor for consideration when establishing the nature of competitive interactions among plants (Schmid *et al*., 2015; Klimešová *et al*., 2018a), as horizontal distribution of roots has the potential to detect ‘root territories’, such as the size of the plant ‘neighbourhood’ and root diversity within it (Brisson & Reynolds, 1994; Brisson & Reynolds, 1997; Casper & Jackson, 1997). Horizontal root distributions were also frequently measured in the 1990s in studies exploring the causes and consequences of root plasticity for nutrient uptake (Hutchings & De Kroon, 1994; Hodge, 2004). For example, comparisons have been made between root lateral distribution in several soil compartments in response to nutrients (Hodge *et al*., 2000; Hutchings *et al*., 2003; Mommer *et al*., 2012), neighbours or both (Bartelheimer *et al*., 2006; Cahill *et al*., 2010; Schmid *et al*., 2015).

While root coring and the digging of trenches are the most common methods to determine the lateral spread of roots, as described here, alternative methods include the application of radioactive tracers or stable isotopes (Currie & Hammer, 1979; Mamolos *et al*., 1995; Johnsen *et al*., 2005; Peek & Forseth, 2005). These methods measure the distance from the enriched patch (in metres) to the plant that has taken up the signal. Also, species‐specific DNA markers have been used to determine the horizontal distribution of species, based on their presence in a below‐ground soil grid sample (Jones *et al*., 2011; Hiiesalu *et al*., 2012).

#### a. Sampling and measurement procedures

##### Root coring

See section **XI. 1. Vertical root distribution**. Taking (many) root samples to measure root biomass or length is an ideal option to measure the variation in root placement per surface of soil or pot. When interested in quadrants (e.g. in cases of root foraging responses, see Jackson *et al*., 1996; Visser *et al*., 2008; Rewald *et al*., 2011), a sharp custom‐made metal cross with blades can be hammered into the pots to cut sections.

##### Digging a trench and surveying intercepts on a grid wall

A slightly older protocol to sample horizontal root distribution is to dig a trench of a size that captures most of the extent of the root system(s) under investigation. See section **VI. 3. a. Overview of root sampling methods**. Regarding the lateral root mass distribution index, root mass is approximated by root count.

#### b. Future research directions

Root horizontal distribution of plant species is challenging to assess in mixed communities, unless species have distinct root characteristics. If roots are traced back to the base of plants, distinct root characteristics can sometimes be determined. Alternatively, species percentages can be determined from genetic analyses of root samples collected from cores (see section **VII. 4. Separating roots by species**).

## 12 Root morphology

Root morphology refers here to the external shape or form of roots. Individual traits provide metrics on various aspects of root shape and the biomass invested in these forms. Root morphology reflects, to an extent, the anatomy (i.e. internal root tissue organisation) and architecture (i.e. general root system organisation). As such, morphological traits provide useful information about below‐ground allocation supporting soil exploration (e.g. SRL, Ryser, 2006; Freschet & Roumet, 2017), influencing growth and resource conservation (e.g. Bauhus & Messier, 1999a; Ryser & Eek, 2000). Morphological traits have been used to broadly investigate plant foraging strategies, adaptation to climatic conditions, and ecological adaptations to local environments such as light (Comas *et al*., 2002; Comas & Eissenstat, 2004), soil age, altitude (Alvarez‐Uria & Körner, 2011) and nutrient distribution in soils (Ostonen *et al*., 2007; Holdaway *et al*., 2011; Valverde‐Barrantes *et al*., 2015; Freschet *et al*., 2017). Finally, morphological traits may also reveal effects on ecosystems with traits mirroring ranges of root chemistry and recalcitrance that have potential consequences for root degradation and soil carbon accrual (Freschet *et al*., 2012a; Birouste *et al*., 2012).

Morphological descriptors are among the easiest root traits to measure and have therefore gained much attention in root research, particularly root diameter (average and distributions across orders), SRL, and root tissue density (RTD; Iversen *et al*., 2017). However, these traits represent only a few aspects of root functioning and are often considered without the inclusion of traits that may be more directly linked to functioning but are cumbersome to measure. In this context, recent studies and syntheses have emphasised the need to consider selecting an inclusive set of root traits associated with a function of interest (e.g. McCormack *et al*., 2017; Freschet *et al*., 2018). As relationships between root functions and morphology vary to some extent depending on taxa, growth form, or interactions with other organisms such as mycorrhizal fungi (Eissenstat *et al*., 2015; McCormack *et al*., 2015a), it is important to also consider root morphological traits in the broader context of root function and evolution.

As discussed under **IV. Below‐ground plant entities and root classifications**, root systems are not homogeneous, but consist of different orders, ages and diameters, all with potentially different functions. As root morphological traits vary strongly with root orders (Pregitzer *et al*., 2002; Guo *et al*., 2008a), they are highly sensitive to the type of root sampling and sorting. For instance, when considering several root orders together, the proportion of roots of different diameters in a sample must represent all root orders adequately, alternatively over‐representation or underrepresentation of coarser, heavier roots may lead to errors in trait calculation and spurious comparisons across species. Bias in root diameter distribution may also result from a proportionally higher loss of fragile fine roots compared with coarser roots during sampling and cleaning. Finally, lack of care during root cleaning and the weighing of mineral soil material together with roots, can strongly bias root weight estimations and morphological trait calculations.

Finally, examination of root morphological traits among species and ecosystems can lead to a greater understanding of how plants function and respond to the environment, although, as previously mentioned, it is important to also include other traits in these broad studies. At the global scale, changes in climatic conditions show a strong effect on average species trait values of SRL, root diameter and RTD (Freschet *et al*., 2017; Valverde‐Barrantes *et al*., 2017). Cold and dry climates seem to favour thinner and less dense root systems, which possibly reflects the demands for fast‐growing and efficient root systems in areas characterised by short pulses of nutrient availability and limited growing seasons (Körner & Renhardt, 1987; Chen *et al*., 2013). Soil texture has a significant influence on soil aggregation, water retention and fertility, thereby also affecting root morphological traits (Oades, 1988; Bronick & Lal, 2005). Furthermore, higher cation‐exchange capacity, higher soil water content and richer soil organic matter content tend to occur in fine‐grained soils compared with texturally coarser soils (Schaetzl & Thompson, 2015). Species with thinner roots, high SRL and low RTD may be more competitive in coarse‐grained soils, where they can benefit from increasing the total absorbing length per unit carbon invested (Eissenstat, 1992; Holdaway *et al*., 2011) or dry upland sites, where they may benefit from less radial resistance to water movement (Comas *et al* 2012). Conversely, soils with high clay content require an increased force for the physical penetration of the soil, favouring denser roots and thicker diameters (Materechera *et al*., 1992).

### 1. Mean root diameter and mode of diameter distribution

*Mean root diameter* is the average of all root diameter observations of a root diameter distribution (typical units: mm).

*Root modal diameter* is the diameter of a root distribution that is most frequently represented (typical units: mm). It is typically estimated from classifying the range of root diameter into root diameter classes of 0.1 mm, or even 0.05 mm for very fine roots.

*Root median diameter* is the value of a root diameter distribution for which half of all root diameter observations fall either above or below (typical units: mm).

**Fig. 15:**
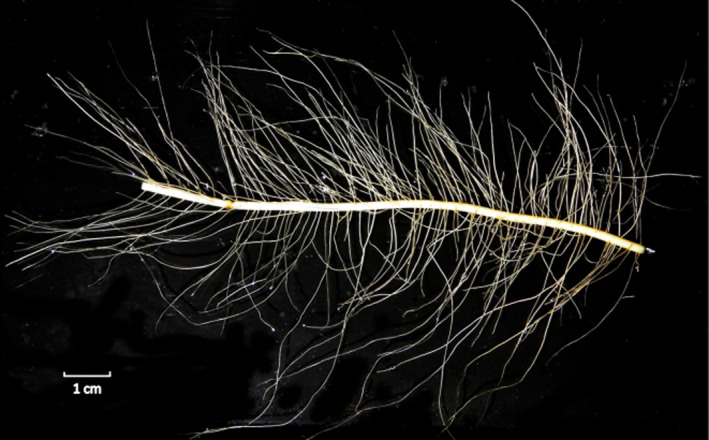
Picture of a piece of a root of *Typha latifolia* L. (Typhaceae), an herbaceous wetland monocot. This root illustrates the potential root order dimorphism and its consequences for measurements of morphological traits. The pictured root, grown in water, has a total length of 6.33 m, out of which 0.11 m (1.7%) is taken by the thick basal root. Average diameter of the fine lateral roots is 0.21 mm, that of the basal root is 1.66 mm. Average diameter of the whole sample is 0.23 mm. Specific root length of the basal root, lateral roots and total sample are 6 m g^−1^, 230 m g^−1^ and 138 m g^−1^, respectively. The porous basal root with a root tissue density (dry mass per fresh root volume; RTD) of 0.08 g cm^−3^ takes up 53% of the sample volume, while the less porous lateral roots with a RTD of 0.125 g cm^−3^ take up 47% of the volume, resulting in an average RTD of 0.10 g cm^−3^.

Of morphological root characteristics, diameter is possibly the most tangible one, as roots are visibly thick or thin. However, quantifying root diameter of a plant is not straightforward (see also under **IV. 4. Root diameter‐based classifications**). Root diameter within a root system typically has a skewed distribution. Even for herbaceous plants, the largest portion of the total length of a root system is usually made of fine roots, while a few coarse roots may provide most of the biomass (Boot & Mensink, 1990; Fig. 15). For woody species this problem is even larger, due to secondary growth of some roots. The mean value may be a useful trait for species ranking, but a plant may actually only have a few roots, if any, that fall in range of its mean diameter. This should be taken into consideration if data are used in modelling or scaled to the community level.

There can be strikingly large variation in root diameter among co‐existing species that follows phylogenetic patterns (Comas & Eissenstat, 2009), but root diameter differences among species is also associated with specific adaptations to environmental stresses and to life history (Ryser, 1998). Annual species generally have thin roots that are specialised for quick resource acquisition, while thick roots may be an adaptation to harsh conditions and improve stress tolerance (Genard *et al*., 2001) or an adaptation to support greater mycorrhizal colonisation (Comas *et al*., 2014). Woody species adapted to highly organic soils also can have especially thin root tips (Valenzuela‐Estrada *et al*., 2008) as can leguminous species (Freschet *et al*., 2017). Aerenchymatous roots tend to be thick as thin roots may constrain oxygen supply under hypoxic or anoxic conditions (Visser *et al*., 2000).

At the intraspecific level, root diameter may vary according to branching order, plant’s ontogenetic stage, nutrient availability and mycorrhizal infection (Berta *et al*., 1995; Bouma *et al*., 2001a; King *et al*., 2002; Pregitzer *et al*., 2002). Plants grown under high nutrient supply tend to have thicker roots but increased nutrient supply may also lead to increased proliferation of fine roots (Ryser & Lambers, 1995; King *et al*., 2002; Hodge, 2004). These potentially simultaneous, but contrasting, effects of increased nutrient supply on diameter of different root orders further emphasise the importance of quantifying diameter distribution of the entire root system, rather than relying on the mean diameter of the root system. Measuring mean root diameters for explicit root orders is another way to overcome the problem of ontogeny and root functional types to make data comparable across studies and species (McCormack *et al*., 2015a). A bimodal distribution of root diameter can also indicate two functionally distinct portions of the root systems, which should be treated separately. Despite the classical focus on mean root diameter, measuring root diameter distribution or root modal diameter may therefore bring particularly valuable insights into root morphology and functioning (e.g. Boot & Mensink, 1990; Pregitzer *et al*., 2002; Poorter & Ryser, 2015).

Together with RTD, root diameter distribution is a trait that underlies variation in SRL, therefore affecting a plant’s capacity to explore the soil. Plant’s responses to environmental conditions may result in changes both in root dry tissue density and in root diameter, but changes in these two traits may simultaneously have contrasting effects on SRL: thinner roots might increase SRL while higher RTD decreases SRL (Ryser, 1998). Consequently, the assessment of root diameter distribution may be important for a proper understanding of the response in SRL.

#### a. Sampling recommendations

See section **VI. Experimentation and sampling in laboratory and field**.

#### b. Storage and processing

See section **VII. Root washing, sorting and storage**. Root morphological traits should be measured without delay, as root fresh mass and root air space can change during storage, especially in aerenchymatous roots. Very importantly, if there is any doubt about saturation of the roots, they should be rehydrated, for example by keeping them between moist paper towels at 4°C overnight (as described for leaves in Ryser *et al*., 2008). If measurements must be delayed, samples can be measured after storing in a refrigerator within a few days. Root preservability depends on storage conditions and varies among species, but a delay of 2 d is often considered the upper limit for reliable measurements. If stains are used for imaging and samples are stained after they are processed (e.g. with neutral red at 0.16 g l^–1^), stains act as a preservative and can allow for sample storage for up to 2–3 wk. Roots can also be stored in ethanol (concentration > 50%) or frozen, but one should consider that some of the contents may be alcohol‐soluble, and build‐up of ice‐crystals may damage cells and may lead to leaching of some of the contents when thawed.

#### c. Measurement of root diameter

Root diameter can be measured manually using a microscope, or digitally based on image analysis.

##### Microscopic measurement

A representative sample of the root system should be selected, and diameters of 100–150 random root pieces measured using a microscope with a measuring ocular. The root system should be cut in pieces of *c*. 1 cm, thoroughly mixed and a representative part of the root system sampled, possibly after sequential subsampling. The sampled 100–150 pieces of roots should be arranged on microscopic slides with some water, covered with a cover slide. A ×40 magnification enables measurement of roots up to *c*. 1 mm diameter in steps of 0.025 mm. Just as when measurements are taken using image analysis, root diameter distribution should be analysed.

##### Measurement using image analysis

**Fig. 16:**
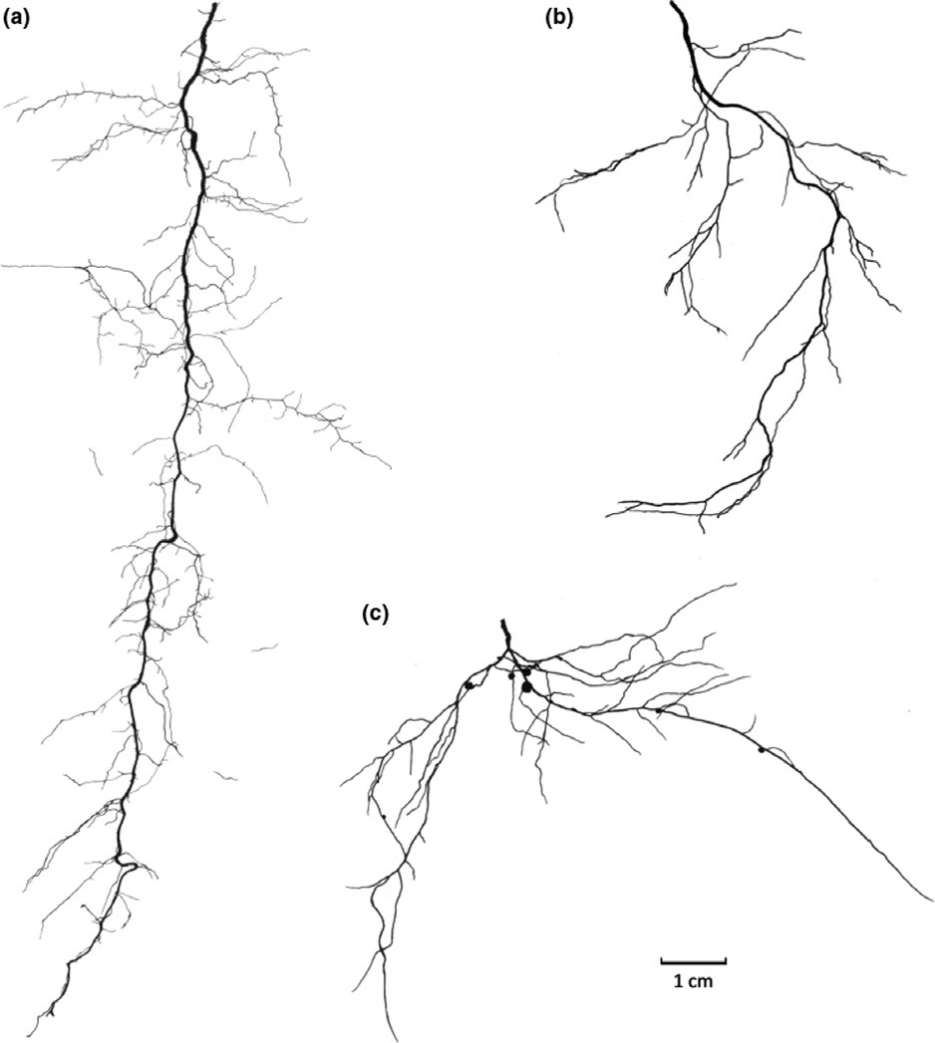
Typical greyscale images used for root morphological trait analysis. Roots from three herbaceous species with contrasting root types are displayed, a grass (a) *Bromus erectus* Huds. (Poaceae); a forb (b) *Sanguisorba minor* Scop. (Rosaceae); and a legume (c) *Lotus corniculatus* L. (Fabaceae). Note that nodules are visible on the legume roots but are typically removed for morphological analyses of roots (and nodule biomass should be separately assessed, see section **XX. 2. Nodule investment**). Also, note that roots should not be allowed to overlap too much for accurate length, diameter and volume estimations: this can be achieved by clipping the roots into subunits.

For root diameter measurements using image analysis, a high‐resolution and high contrast image of the root system is needed (e.g. Fig. 16). In general, it is advisable to scan images on a flat‐bed scanner with a back‐light system (also referred to as transparency unit) and resolution of at least 600 dpi (Delory *et al*., 2017; Rose & Lobet, 2019) and up to 1200 dpi or more for samples of very fine roots. At higher resolutions, it is important to check whether root hairs are mistakenly identified as roots. To obtain good contrast, scanners must include a back‐light system. When acquiring a scanner, consider that dpi is not the only important criterion for acquiring good images; the quality of the optics varies also dramatically between scanners. Roots should be spread in a transparent glass or plastic tray containing a layer of water *c*. 4–5 mm deep. Deeper water is sometimes needed to avoid having roots distorting the water surface and creating shadows but should be generally avoided as spreading roots vertically is likely to lower image resolution and lead to underestimation of total root length. Glass trays outlast plastic ones, which are more apt to accumulate both large and microscopic scratches on the tray, eventually dramatically lowering the quality of scans. It is also good to replace trays regularly, to avoid any bias by scratches. Glass trays can be easily built from regular glass glued with plastic rims. Alternatively, for small samples, Petri dishes can be used for scanning, which can be replaced frequently, and can also be useful for subsequent drying of large samples. Distilled water is preferably used. To avoid the formation of air bubbles in the tray, the water should preferably be de‐gassed either by a vacuum treatment or by leaving it to stand overnight prior the use, or at least, it should not come from an aerated faucet. Roots should be positioned without longitudinal overlap using plastic‐coated or rounded‐tip tweezers to avoid scratching the tray. Large samples may need to be separated into smaller subsets and homogeneously spread across the entire tray. Care should be taken not to position roots very close to the edges of the tray, because edge lines might interfere with image analysis.

Once the image is acquired, a range of image analysis software can be used, including the recently released free and open‐source RhizoVision Explorer (Seethepalli & York, 2020; fast and reliable even with large size images, can be downloaded at https://doi.org/10.5281/zenodo.3747697), the free and open‐source IJ_Rhizo plugin for ImageJ (Kimura & Yamasaki, 2003; Pierret *et al*., 2013, can be downloaded at https://www.quantitative‐plant.org/software/IJ_Rhizo), or the commonly used commercial WinRhizo (Regent Instruments, 2000). rhizovision explorer and winrhizo are standalone software with full user interfaces built for the Windows operating system, while IJ_Rhizo is a macro for the cross‐platform imagej software. The accuracy of any digital image analysis strongly depends on the quality of the initial image (Ortiz‐Ribbing *et al*., 2003). Digital analyses are sensitive to resolution of the image and to thresholding setting, and care has to be taken with proper settings (Bouma *et al*., 2000; Zobel, 2008; Delory *et al*., 2017; Rose & Lobet, 2019). Photographic images are not recommended because shadows are difficult to avoid and distort the analysis (Ortiz‐Ribbing *et al*., 2003). The fist step of analysis with all these software is to determine the root from its background, which can be done by simple greyscale thresholding if root images are acquired using the back light described above, assuming the roots are dark and the background is white. All these software include filtering options that can delete small particles such as sand before trait measurements. Measuring length is generally accomplished using skeletonisation that determines the central pixels of the roots, and then determining the length of the skeleton. Image analysis programs acquire localised root diameters by measuring it from the image at each skeletal pixel, multiplying pixel size by the smallest number of pixels on a line perpendicular to the root axis (Bouma *et al*., 2000). Diagonal pixels are accounted for by using the square root of 2 (√2) during length measurements. All these software packages further provide an average diameter per image. For WinRhizo, average diameter is based on the total projected area divided by the total root length in the image, which may be inaccurate when heterogeneous diameter classes are present. RhizoVision Explorer and IJ_Rhizo both report an average diameter using the diameters of all skeleton pixels, which is more accurate. However, both IJ_Rhizo and WinRhizo use the average diameter and total length measured to calculate total volume, which has been shown to lead to severe underestimation of root volume when heterogeneous root diameters are present (Delory *et al*., 2017; Rose & Lobet, 2019). If these software are used to calculate total root volume, it has to be done separately for each diameter class and summed to achieve greater accuracy (see section **XII. 3. Root tissue density and root dry matter content**). RhizoVision Explorer does not suffer from this inaccuracy, since total volume is measured by assuming each pixel of the skeleton is a cylinder of variable diameter and summing the volumes of all skeleton pixels.

Programs use greyscale images in TIFF, PNG or JPEG file format, which are further converted into thresholded (binary) and skeletonised images. If image size is not an issue, TIFF or PNG formats are usually preferred as it contains the highest level of detail compared to JPEG lossy compression. TIFF and PNG support lossless compression that does not decrease quality but can reduce file size substantially for high contrast images. The threshold for identifying roots can either be set manually, or an automatic threshold detection can be applied (Bouma *et al*., 2000). Extreme threshold values can produce overestimations or underestimations of root diameter. If high threshold values are needed to detect most or all of the finest roots in a sample (see section **XII. 2. Specific root length**) and these threshold values are high enough to overestimate root diameter, samples may benefit from staining (see below). For images with poorer contrast or heterogenous lighting, methods such as adaptive or local thresholding may be used in software such as imagej, before using the thresholded image with whichever root image analysis software. Newer machine learning methods for segmenting roots from complex backgrounds such as soil are becoming available. Alternatively, operators need to find a balance between detecting a reasonably accurate proportion of total root length while not substantially overestimating root diameters. In such cases, the automatic thresholding mode might be preferable, particularly for new operators.

Two frequently encountered problems when acquiring root images are the lack of contrast between the background and the roots, and the overlapping between roots in pictures with high RLD. For woody plants, poor contrast may not be a problem since many roots show some level of pigmentation, but the roots of nonwoody plants are often pale. Before scanning, pale roots can be stained to improve contrast with the background, alternatively threshold setting can be used (within limits) (Bouma *et al*., 2000; Delory *et al*., 2017). Stains that have been used include neutral red (Bouma *et al*., 2000) and toluidine blue (Lupini *et al*., 2018) but some other stains can also be used, depending on root properties (Richner *et al*., 2000). As staining slightly changes the chemical composition of the root, stained root samples should not be further used for chemical analyses, for example C or N concentrations. The density of roots on the tray can be a problem if large root systems must be scanned at once. Delory *et al*. (2017) recommended that images should have an RLD lower that 1 cm cm^−2^.

##### Comparisons of root diameter distribution

Root diameter distribution can be expressed as a frequency distribution across specified diameter classes, for example 0.1 mm intervals or 0.05 mm intervals for very fine roots, depending on the species studied (Boot & Mensink, 1990; Pregitzer *et al*., 2002). It can also be described by several complementary metrics, namely mean, median and modal root diameter to facilitate its comparison across plants and species. Calculating these metrics allows also for the identification of right‐skewed and potentially bimodal distributions, as long as diameter classes are chosen fine enough. To positively identify bimodal distributions, visual inspection of the distribution is unavoidable. The presence of a bimodal distribution suggests that the root system may need to be separated into two functionally distinct parts, which will need to be treated separately. The proportion of the root system represented by the mode could be an additional informative parameter, however, it is highly dependent on the choice of diameter classes and should therefore be treated carefully in interspecific comparisons. Furthermore, while it is possible to calculate the average mode across a cohort (sample) of analysed root systems and compare different cohorts statistically, comparing proportions of the root system represented by the mode is only valid if all compared samples have the same mode. Therefore, if proportions or frequencies are compared between samples, they should be focused on root orders and not on artificial diameter classes.

#### d. Future research directions

Fine roots primarily comprise stele tissue and the tissues outside the stele (including the epidermis, exodermis, cortex and endodermis, *sensu* Kong *et al*., 2017). To better understand the ecological constraints behind variations in root diameter, future studies should focus on the role of these tissues in contributing to root diameter, and address the physiological and ecological implications for roots constructed differently. Additionally, measuring root median and modal diameter is an interesting avenue to explore variations in root morphology and minimising confounding effects resulting from the pooling of different classes of roots (with potentially different functions).

### 2. Specific root length

*Specific root length* (formal name: root specific length) is the length of a root per unit dry mass (typical units: m g^−1^) (frequent abbreviation: SRL).

Specific root length is often regarded as a core trait for below‐ground economics, as it reflects the potential extent of soil exploration (for nutrients and water) per unit cost (in terms of plant biomass allocation). Root length is considered to be key for nutrient acquisition, especially in situations when plants are competing for below‐ground resources (Andrews & Newman, 1970; Caldwell *et al*., 1985). Similarly, root dry mass reflects the investment of photosynthates in the production of this root length. Therefore, SRL has been considered as the below‐ground analogue of specific leaf area, which is a central trait in the leaf economics spectrum (Reich *et al*., 2014). However, a consistent response of SRL to resource limitation is often missing (e.g. Freschet *et al*., 2015a). A possible explanation for this is that responses of SRL are confounded by different functional roles of different root orders, allometric effects, and contrasting effects of the environmental factors and mycorrhizal associations on the different components of SRL (i.e. root diameter and tissue density; Poorter & Ryser, 2015).

Variation in SRL is a result of variation in two component traits: root diameter, which determines the length of the root produced per unit root volume, and RTD, which determines the root volume produced per unit dry mass. SRL can be mathematically represented by these two traits as stated by Ostonen *et al*. (2007), where:

$$\;\text{SRL}=4/(\text{root \textbackslash{} }\;\text{tissue \textbackslash{} }\;\text{density}\times d^{2}\times \pi )$$

with *d* the root diameter. However, it is important to note that this relationship is valid only for a given root diameter class and should not be used for average values of root diameter in heterogeneous root systems (see section **XII. 3. Root tissue density and root dry matter content**).

In addition to being an important variable in the context of resource uptake, variation in SRL and its components is also associated with root lifespan, that is, resource loss rate (Ryser, 1996; McCormack *et al*., 2012). SRL can further affect ecosystem level processes, such as root decomposition rates (Hobbie *et al*., 2010).

#### a. Sampling recommendations

See section **VI. Experimentation and sampling in laboratory and field**.

#### b. Storage and processing

See section **XII. 1. Mean root diameter and mode of diameter distribution**.

#### c. Measurement procedure

Root length must be measured on fresh samples, which are then dried in a drying oven at 60–80°C for 24–48 h and weighed. For relatively small samples of fine roots, drying can be achieved within 24 h but it may take longer for woody or desiccation‐tolerant root systems, especially if they are large and/or wet. Fine roots tend to stick on surfaces when drying, and therefore, to avoid losses of material and additional work to scrap samples, it is helpful to blot the root surface water with paper towel, and gently roll the roots to a ball to reduce their surface area and confine samples before putting them in the bag or envelope to be dried. Often, samples for SRL are small, especially when sampling from specific root orders, and require weighing on a microbalance, which typically have small weighing plates to accept samples. Alternatively, if samples are large, roots can be dried in Petri dishes, which can be stacked if each layer is covered with a sheet of paper.

The measurement of the root length can be done either with the grid‐intersection method, developed by Newman (1966) and modified by Marsh (1971) and Tennant (1975), or with an image analysis software. The grid‐intersection method has the advantages of an easier sample preparation for fine pale roots and not requiring expensive machinery, while image analysis saves the tedious hand count. Comparisons of the two methods have shown that both deliver accurate data (Delory *et al*., 2017). When using the grid‐intersection method care has to be taken to avoid observer fatigue that may lead to inaccurate results, while when using image analysis care has to be taken to achieve proper contrast of the image by staining pale fine roots, and to spread the roots on the dish extremely carefully to avoid overlap (Goubran & Richards, 1979; Bouma *et al*., 2000; Ortiz‐Ribbing *et al*., 2003; Delory *et al*., 2017).

##### Grid‐intersection method

The grid‐intersection method is based on a linear relationship between the number of intersections between roots randomly scattered on a grid and the grid lines. Tennant (1975) describes the relationship as: Root length = 11/14 ⋅ number of intercepts (sum of horizontal and vertical) × grid unit length.

The root sample is spread on a transparent tray, with an underlying grid. The tray should contain a few millimetres of water, just enough to be able to spread the roots easily around with two pairs of tweezers, but not so much that the roots are floating around. The roots should be spread evenly across the tray, but their position and direction with respect to grid lines should be random. The tray should be large enough for the given sample size to avoid crowding.

To avoid observer fatigue, it is important to minimise the effort by choosing an optimal sample size. Unnecessarily large samples take time to prepare and measure, without improving data quality. Furthermore, operator fatigue may decrease the accuracy of the measurements (Richards *et al*., 1979). It is often better to measure multiple small subsamples than spending a long time on one large sample. Experience has shown that *c*. 2–4 m of root on a Petri dish of 20 cm diameter using a 1‐cm grid works very well. If the sample is smaller, a higher number of counts per sample necessary for an accurate result can be achieved by choosing a smaller grid size. A good method for an immediate test about the quality of the measurement is to compare the vertical and horizontal counts. Given the randomness of intersections, the counts should be close to each other. Less than 10% difference between the counts can be considered sufficient for most practical purposes.

The roots should be easily visible. A magnifier of *c*. ×3 helps observing the roots. The roots should have a high contrast with the background. Proper illumination is essential. For pale roots a dark background with illumination from the side is efficient. A tally counter is used to count the root–gridline intersections along the horizontal and vertical gridlines.

##### Root length assessment using image analysis

Details for image collection (e.g. Fig. 16) and thresholding for root length analyses are similar to those above for root diameter (see section **XII. 1. Mean root diameter and mode of diameter distribution**). The choice of threshold values for root length analyses should aim at finding a reasonable balance between capturing total length (or reasonably high proportion) of very fine roots and avoiding the detection of nonroot objects. Thresholder images used by the system evaluate root length as measured on the skeleton image (Bauhaus & Messier, 1999b; Regent Instruments, 2000). Skeletonisation is a transformation that results in a pixel thick line overlaid along the centre of objects within the image, where IJ_Rhizo and WinRhizo use thinning, while RhizoVision Explorer uses the distance map (Bauhus & Messier, 1999b; Kimura *et al*., 1999; Richner *et al*., 2000; Seethepalli & York, 2020). Additionally, WinRhizo detects overlapping parts depending on the grey gradation between pixels in the image. One common method to improve root detection is to employ the Lagarde’s mode (Bouma *et al*., 2000) where the user can personalise a particular threshold separating background from root areas, and extend the *a priori* threshold to the rest of the image analysis. Recent versions of RhizoVision Explorer, WinRhizo and IJ_Rhizo also provide length estimates based on the Kimura method that essentially sums the pixels in the skeleton while making corrections for diagonal segments and crossings. As mentioned above, lower resolution images can have a biased effect on the analysis, since in low‐resolution images individual pixels cover a larger area, leading to larger overlap between threshold areas, which can lead to underrepresentation of the total length (Bauhus & Messier, 1999b) (see also section **XII. 1. Mean root diameter and mode of diameter distribution**).

#### d. Future research directions

Specific root length is one of the most studied root trait due to its high potential relevance for plant and ecosystem functioning (e.g. in plant economics, soil exploration, soil exploitation) and its easy of measurement. However, the morphological traits underlying variation in SRL, root diameter distribution and RTD, can vary independently from each other (Poorter & Ryser, 2015), making the response of this trait difficult to predict (Freschet *et al*., 2015a). Future studies would benefit from exploring the response of SRL in light of changes in both RTD and root diameter (and/or root order) distribution.

### 3. Root tissue density and root dry matter content

*Root tissue density* (formal name: root dry matter concentration) is the dry mass of root per unit volume of fresh root (typical units: g cm^−3^) (frequent abbreviation: root tissue density (RTD)).

*Root dry matter content* is the dry mass of root per unit fresh root mass (typical units: mg g^−1^) (frequent abbreviation: root dry matter content (RDMC)).

Terminology for describing the quality of roots in terms of invested dry matter per unit functional root has been inconsistent (Birouste *et al*., 2014). Root tissue density, as a physical variable, is defined as the ratio of mass to volume. For plant material this should technically mean fresh mass per volume (Roderick *et al*., 1999), but this variable may not be the best in describing the economics of investment, that is amount of photosynthates required to build a certain quantity of plant material. Therefore, two other ratios are commonly used to describe the costs of root construction in terms of dry matter: dry mass per fresh root volume, commonly referred to as RTD (Ryser, 1996), and root dry mass per fresh mass, referred to as root dry matter content (RDMC; e.g. Birouste *et al*., 2014).

High RTD or RDMC are generally associated with a conservative plant growth strategy (Niklas, 1995; Vernescu & Ryser, 2009; Kitajima & Poorter, 2010). These traits are also interpreted as investment in hardy and robust tissue, which is expected for organs with long lifespans to resist physical stress, mechanical damage and herbivory (as demonstrated for leaves, e.g. Bumb *et al*., 2018). However, for roots, further evidence is needed to support these theoretical expectations (but see Ryser, 1996; Genet *et al*., 2005). Conversely, a low RTD or RDMC is associated with fast growth, fast expansion, and short‐lived organs and is a characteristic of plants with an acquisitive strategy. Consequently, root litter with high RDMC has been shown to decompose slower than litter with low RDMC (e.g. Freschet *et al*., 2012a).

Both RTD and RDMC reflect root anatomy: cell size, cell wall thickness and proportion of vascular tissue in contrast with parenchyma. The difference between these two traits lies in the inclusion of air space. Among plant species of dry environments, a strong correlation has been found between the two traits (Birouste *et al*., 2014), but if the set of investigated species includes species both of well aerated and anoxic soils with large variation in the amount of air space, the relationships may vary, depending on whether root volume or root fresh mass is used as the denominator for the ratio. Aerenchyma contributes to volume but have no mass, and the proportion of air space in wetland plant roots can be up to 50% (Visser *et al*., 2000).

#### a. Sampling recommendations

See section **VI. Experimentation and sampling in laboratory and field**.

#### b. Storage and processing

See section **XII. 1. Mean root diameter and mode of diameter distribution**.

#### c. Measurement procedure

Root dry mass and volume need to be measured to calculate RTD. Root volume (V) can be calculated based on root diameter (see section **XII. 1. Mean root diameter and mode of diameter distribution**). Assuming a cylindrical cross‐section of the roots, the volume of each diameter class can be calculated as: *V* = Π *r* ^2^ l.

where r is the radius of the diameter class, and l is the total length of that diameter class. The volume of the root system is the sum of the volumes of all diameter classes. While this information is provided by most image analysis software, at the date of writing this manuscript, current and previous versions of WinRhizo and IJRhizo still provide an erroneous value of root volume. Using the mean diameter of the whole root system to calculate its volume leads to an error, which can be considerable (Ryser, 2006; Rose, 2017). The calculation must be done separately for each diameter class, because the relationship between root volume and root diameter is not linear. Fortunately, most programs also provide this information, which then can be used to calculate a correct root volume. The recently released free and open‐source RhizoVision Explorer does not have this bias, as described under section **XII. 1. Mean root diameter and mode of diameter distribution** (Seethepalli & York, 2020; https://zenodo.org/record/3747698#.Xz0wqn7gpM8). Alternatively, root volume can be measured directly using displacement of liquid (Curran *et al*., 1996), root buoyancy (Curran *et al*., 1996) or a pycnometer (Visser & Bögeman, 2003; Ryser *et al*., 2011).

Root dry matter content is the ratio between root dry mass and root fresh mass. Measurement of root fresh mass is not trivial. On the one hand, evaporation quickly leads to loss of water, and to an underestimation of the water‐saturated fresh mass. If there is any doubt about saturation of the roots, they should be rehydrated, for example by keeping them between moist paper towels in a fridge (as described for leaves in Ryser *et al*., 2008). Conversely, harvested roots grown in hydroponics or washed with water will have water clinging to their surface, which should be removed by gently blotting between absorbent papers such as filter paper or paper towel. This can be done only if all root surfaces are exposed to blotting, which means that fresh mass can be measured reliably only for relatively small root samples. Lack of visible tracks of water on the paper towel paper used for blotting is a sign of a complete removal of the surface water from the root. After blotting, weighing of the root should be done without any delay, as evaporation quickly dehydrates the root. Observer bias within a study can be minimised by randomly assigning samples to observers.

#### d. Future research directions

As the rate of resource loss is a key aspect of plant economics, future studies are critically need that further demonstrate the ecological role of RTD and RDMC in protecting roots from multiple biotic and abiotic influences.

## 13 Root anatomy

Root anatomy is the general term that refers to the internal structure of roots and may focus on the internal makeup of root tissues in relation to overall root shape and size. Anatomical examination of root tissues allows for a unique perspective on root functioning, including water and nutrient movement through root tissues, physical interactions of roots with soil microbiota, and plant adaptation to environmental conditions (e.g. soil structure and profile, nutrient availability, oxygen limitation, drought, low temperature) (e.g. Peterson, 1992; Peterson *et al*., 1993). Furthermore, anatomical examination allows for assessment of root ontogenetic development and viability, which also impact root functioning, for example, in resource acquisition or root lifespan. As a result, root anatomical studies are useful for differentiating different types of roots (e.g. absorptive vs transport, or specific root orders) according to their function. Selecting roots within designated root functional types has been shown to be critical for appropriate interpretation and contextualisation of root trait data (Bagniewska‐Zadworna *et al*., 2012; Freschet & Roumet, 2017). However, to date, studies of root anatomy and its relation to root functioning among different species (*sensu* Hodge *et al*., 2009; Freschet & Roumet, 2017) are rare.

Numerous procedures have been developed and published for plant organ sampling, preservation, storage, cross‐sectioning, and staining for anatomical analyses (e.g. Jensen, 1962; Huang & Yeung, 2015; Yeung *et al*., 2015). However, aspects of these procedures may still appear complicated for inexperienced researchers. Similar to procedures for studying other root traits, proper sampling of roots to control for position in the root system is essential for comparative studies of root anatomy (*sensu* Pregitzer *et al*., 2002; McCormack *et al*., 2017). Moreover, sample processing of different root functional types requires different methods. For instance, transport roots, characterised by profuse secondary xylem, require longer fixative time compared with absorptive roots characterised by primary growth.

### 1. Microtechnique method for the analysis of root anatomy

#### a. Generalities of microtechniques used for the analysis of root anatomy

Most root anatomical properties are measured on cross‐sections or longitudinal sections of the roots of interest. Microtechnique utilises the use of a light microscope to visualise these sectioned roots. The simplest approach utilises free‐hand sections, which entails using a razor blade to cut root sections as thin as possible by hand. This approach, however, presents many difficulties. Suitable transverse free‐hand sections are particularly difficult to cut, especially on fine roots, and sections are often too thick and difficult to study in a serial manner (e.g. analysing a series of cross‐sections through a root). As they generally do not provide clear images of internal structures, they do not offer reliable information on cellular content.

By contrast, the same measurements on tissues that have been fixed, embedded, sectioned using rotary microtome and stained with a specific histochemical stain result in more detailed and sharper images. Classical methods of tissue embedding for anatomical studies utilise several steps during root processing, including fixation, dehydration, embedding, sectioning and light microscopy observation of roots. A critical aim of anatomical studies is the fixation and preservation of cells in tissues as close as possible to their exact state at the time of sampling. It should be noted that chemical fixatives can cause several artefacts in the root tissues, so that carefully selecting fixing solutions and their timing of use is critical to reduce or avoid tissue shrinkage, swelling, organelle degradation and other changes of cellular components. While use of chemical fixatives must be treated and applied carefully, they still are applied in most situations. Physical fixation, however, is not a practical approach for the majority of anatomical studies. It involves freezing roots in liquid nitrogen which may be difficult in the field and can only be applied on extremely small pieces of roots to maintain comparable freezing rates and avoid artefacts.

Fixing agents are traditionally described by two main types of characteristics: (1) their reaction with proteins to either alter protein chemistry and protein aggregation or form cross‐links that creates a nonaggregated structure (coagulants and noncoagulants); and (2) their potential for incorporation into the tissue (additive or nonadditive, see below). Coagulant fixatives, based on ethanol, methanol, acetone or chromium, remove water from tissues leading to coagulation and cause irreversible protein denaturation. When cells of roots are exposed to these fixatives, they change the conformation and solubility of protein molecules, and cause changes in the structure of other cellular components, such that these are not recommended for root anatomical analyses. Noncoagulant fixing agents, such as formaldehydes, glutaraldehyde, acrolein, and osmium tetroxide (OsO_4_) react with proteins and other components, which results in better preservation of root cellular components compared with coagulant fixatives. Additive fixatives are those that can be retained in the tissue after rinsing (many of additive fixatives are noncoagulants). They combine with molecules in the cell to quickly become an actual component of the cell structure, and can continue to impact subsequent steps in processing. This contrasts with nonadditive fixatives, such as alcohol and chromium fixatives, that are not retained in the tissue after the fixation period and are unsuitable for conducting detailed histological research (but are suitable for structural analyses).

Many of the commonly used fixative solutions include multiple ingredients. Fixatives such as FAA (formalin–acetic–alcohol) or AA (acetic–alcohol) are acidic fixatives that dissolve cellular structure, and break bonds between nucleic acids and proteins. Their use generally results in the loss of most cellular components and of the generation of several other undesirable artefacts, including cell shrinkage, membrane invagination and plasmolysis. Aldehydes, such as formaldehyde or glutaraldehyde, are the most widely used and tested fixatives for preserving root structure and retaining tissue components *in situ*. Caution should be used, however, when selecting an appropriate fixative, given that ‘not all roots are the same’. Preliminary tests should be conducted to determine the balance of ingredients that best preserves the ‘natural’ state of the root that is going to be the subject of a critical analysis. The ideal fixation should immobilise complex macromolecular assemblies in their native state *in situ* and retain the spatial relationship of all organelles. In addition to the concentration and blend of fixative components, critical aspects of preserving root structure include: temperature, fixative pH, length of fixation time, osmolarity and the penetration rate of the fixatives used, as discussed in more detail below.

The most universal technique for root chemical fixation is presented in the procedure below. It is well adapted to the routine anatomical analysis of all types of roots and rhizomes and to observe root mycorrhizal colonisation. It can be used to sample roots under both laboratory or field conditions and only requires access to a refrigerator.

#### b. Recommended microtechnique method

Harvested samples of roots should be immediately placed in glass vials or Eppendorf tubes filled with a fixative composed of 2% glutaraldehyde (pH 6.8, v/v) and 2% formaldehyde (pH 6.8, v/v) in cacodylate buffer (0.1 M). It is crucial to mix these solutions immediately before use and that the level of the fixative in the vial or tube is at least 5 mm above the root samples. Samples must sink to the bottom of the solution, alternatively the open‐topped vials or tubes need to be placed in a vacuum desiccator and vacuum applied to remove excess air from the tissues and facilitate penetration of the fixative. After 2 h of fixation at room temperature, the sample vials should be transferred to 4°C. After overnight incubation in the fixative, the samples should be rinsed three times with cacodylate buffer (0.05 M; pH 6.8) and then dehydrated in a graded ethanol (10, 30, 50, 70, 80, 90, 95, 3 × 100%, 1 h in each solution). During dehydration, water is progressively removed from the tissue and replaced with ethanol to prepare the material for infiltration of the embedding medium. All changes of liquids should be done in the same vials, using a transfer pipette with thin end to avoid losing root samples. Fixed root samples can be stored in cacodylate buffer for 48–72 h when working in the field if more time is needed before proceeding to the steps of dehydration. Dehydration of the tissues can also be paused at 70% ethanol if longer storage times are required. Samples can be stored at 4°C in 70% ethanol for indefinite periods of time.

After dehydration, root samples can be further processed using a variety of techniques suited for histological analyses using light microscopy. Embedding samples in paraffin or paraplast waxes is typically suitable for small, thin roots, however, hard, lignified root samples or very fine roots need to be embedded in a plastic embedding medium or resin, such as Technovit resin, as described later. An ethanol/butanol (TBA) series in concentrations of 3 : 1; 1 : 1; 1 : 3 (v/v) for 20 min each, followed by three changes of pure TBA, is needed before embedding in paraffin or paraplast. Alternatives to TBA are the product Histo‐Clear, isopropanol or xylene. The xylene is rarely used, however, due to its toxicity to humans. Paraffin is readily soluble in the purest concentration of any of these solvents. Samples can be stored overnight at room temperature in the final solution of the solvent that was used. The steps following dehydration are dependent on the melting point of the selected paraffin, which is usually 53–60°C for most of the available products. The paraffin can be melted in an oven with a reliable thermostat. Liquefied paraffin should be prepared at least 1 d before to ensure complete melting of the wax in a container that facilitates pouring. TBA should be replaced by paraffin and samples should be put into the oven for infiltration for a duration of 2 d (at 53–60°C). In the oven, the majority of TBA solvent will decant and be replaced with liquid paraffin. The paraffin should be changed several times while the samples are in the heated oven.

After infiltration, solidified paraffin blocks containing properly oriented root samples need to be created. Several methods can be used to facilitate this procedure, however, the simplest approach is to pour the melted paraffin and samples into specialised casting moulds (commercially available or self‐made from photography paper or aluminium foil can be used to construct casting boats). The moulds or boats are placed on a hot plate and the samples are arranged in the appropriate orientation, which is dependent on whether a transverse, longitudinal or tangential section is required. Avoiding the introduction of air bubbles into the melted paraffin is crucial. Re‐melting of the paraffin is required if bubbles are introduced. Once the samples are properly arranged, the moulds or boats can be moved to the cool end of the hot plate, which will allow the paraffin to solidify. After initial hardening the samples can be placed in a cold‐water bath for final solidification.

The resulting blocks of paraffin with the embedded samples should then be trimmed in preparation for sectioning, remembering to leave some paraffin between the root sample and the face of the block. When roots are very thin and small, several root samples (embedded with an appropriate amount of space between them) can be sectioned simultaneously in the same block. In general, the blocks can be stored in a refrigerator or at room temperature for several months. Young, white roots are often difficult to see when they are embedded in white wax. In this case, eosine, safranine O, or toluidine blue stains in 70% ethanol can be inserted in the dehydration series to provide contrast with the white wax in which they will be embedded. Cross‐sections or longitudinal sections of roots embedded in paraffin or paraplast are obtained with a rotary microtome at a thickness of 8–12 µm with excellent ribbon continuity.

Another approach that can be used to prepare root samples for sectioning is to embed them in Technovit resin. This approach is required when cross‐sections thinner than 8 µm are needed because paraffin is not rigid enough to support the roots to produce satisfactory sections. Good infiltration of resin will also provide more clear images of sectioned material, so it is ideal for root samples, especially those with a considerable amount of lignified tissues. Thinner sections are usually essential to show distribution of the connections between cells, pit characteristics, cell differentiation details during short root formation, cellular configuration of the apical meristems of roots, *etc*. Dehydrated samples, prepared in the same manner as for wax embedding, are incubated in a mixture of ethanol/Technovit 7100 resin in a ratio 3 : 1, 1 : 1, 1 : 3 (v/v) for 24 h at 4°C for each step. The samples are then incubated in Technovit 7100 resin, and finally hardened and embedded in specialised moulds (available commercially) according to the manufacturer’s instructions. As with the paraffin‐embedded sections, an attempt should be made to properly orient the sample before the hardening of the resin.

The choice of the embedding method depends on root type, root thickness and the purpose of later analyses needed, for example measurements of traits. In each case a test run is recommended.

##### Sectioning samples with the use of a rotary microtome (or vibratome)

Both, older, sliding microtomes or fully automated rotary microtomes can be used to obtain sections of a consistent thickness repeatedly in relatively short time. The use of a rotary microtome can be readily learned by observing someone with experience and expertise, also using one of the video tutorials available, for example http://www.histologytutorials.com/. Once one is accomplished in its use, rotary microtomes can be used to obtain serial sections (i.e. sections that represent progressive advances into the tissue). Depending on the size of the sampled sections, several to many sections of roots can be placed on a single microscope slide. After adhering the sections to the slides with an adhesive solution, the wax is dissolved using for example xylene and the tissues become available for staining (see below). Although samples embedded in Technovit cannot be sectioned with ribbon continuity, serial sections can still be obtained by placing individual sections in a row on a slide. Although consistent sections of resin‐embedded samples are more difficult to obtain, they are cut much thinner (*c*. 2 µm thick), so even if only one in five sections is obtained, the obtained sections will still represent the same serial thickness (10 µm thick) obtained from wax‐embedded samples.

The use of a vibratome provides an alternative for obtaining sectioned samples of root tissue embedded and sectioned on a rotary microtome. In this case, roots can be directly sectioned as fresh or after fixation, but without embedding in paraffin. Nevertheless, very thin roots, such as fine fibrous roots, should be simply embedded in an agarose gel that solidifies at room temperature. The gel provides support for the tissue during sectioning. Vibratomes are equipped with a vibrating razor blade to cut through root. The vibration amplitude, speed and angle of the blade, as well as section thickness, are all parameters that can be manipulated. Disadvantages of using a vibratome are that sections are thicker than with the rotary microtome and serial sections are not possible.

##### Staining protocols

The selection of a stain and a staining procedure are dependent on the purpose of the anatomical study. Paraffin sections of root tissue, regardless of orientation, can be double‐stained with 1% Safranin O and 0.1% Fast Green and Technovit, resin‐embedded sections can be stained with 0.1% toluidine blue (pH 4.4) for general analysis of root structure (Jensen, 1962).

##### Slide mounting

For samples embedded in paraffin or paraplast: cover the sections on slides with drops of rapid hydrophobic embedding agent, for example Canada balsam or Entellan®. The space between the cover glass and the slide should be completely filled, avoiding air bubbles. The cover glass must be sealed right up to each edge. Prepared slides can be stored for years. The sections embedded in Technovit do not need mounting, and mounting of vibratome obtained sections depends on the purpose, for example for immunocytochemistry mount in Prolong Gold or for simple observation mount in phosphate‐buffered saline (PBS) buffer or PBS : glycerol, 1 : 1 v/v.

### 2. Percentage of viable root cells

*Fraction of viable root cells* is the fraction of vital root cells per total number of cells (unitless). It can be measured in the entire cross‐section or in particular tissues.

Root lifespan varies considerably, depending on the species and environment (Eissenstat & Volder, 2005) but different tissues within a root can die at different times. As a result, the functioning of a root changes throughout its lifespan. For example, a root cannot take up soil resources that require energy for uptake once the root cortex dies.

#### a. Sampling recommendations

See section **VI. Experimentation and sampling in laboratory and field**.

#### b. Storage and processing

It is suggested to process samples immediately after collecting. Cleaned root samples can either be hand‐sectioned, or, better, cut with a vibratome into 30–35 µm thick cross‐sections (see above for the benefits and disadvantages of both approaches). An assay utilising fluorescein diacetate (FDA) can then be used to assess cell viability in different portions of the root, which can be related to root age (if known) or stress conditions. To this end, the sections are then incubated in 100 µl of FDA solution (5 mg of FDA dissolved in 1 ml of acetone to form a stock solution which is then diluted by at least 1 : 1000 in PBS buffer, depending on root material). It is very important to avoid using a high concentration of stain as it may provide erroneous results. Therefore, preliminary staining assays should be performed using different concentrations (0.001, 0.002, 0.005, 0.01 and 0.02 of the stock solution) of FDA to determine the optimum concentration that provides the lowest level of background fluorescence. After 15 min of incubation at room temperature, sections should be rinsed three times in PBS, placed on a slide in PBS and covered with a coverslip. If performed properly, a light green fluorescent signal will only be emanating from living cells (Rotman & Papermaster, 1966), due to the conversion of the nonfluorescent fluorescein diacetate into a fluorescent compound called fluorescein. Background fluorescence can be assessed with heat‐killed roots. This assay provides fine‐scale information on the physiological status within a root cross‐section or for the overall cross‐section.

#### c. Measurement procedure

The fluorescein fluorescence is excited by a wavelength of 470 nm (blue excitation and green fluorescence) and can be registered and quantified (in percentage of living cells of the whole root section or particular tissue) for entire cross‐sections using a fluorescence microscope, or individual cells using an epi‐fluorescence or confocal microscope. A transect or grid to count numbers of fluorescent or nonfluorescent cells is recommended to avoid duplicate measurements of the same cells. Since measurements of viability can vary depending on the concentration of FDA used and any other adjustments to the described protocol, caution should be used in interpreting the results unless consistent practices are uniformly applied. Alternatively, other viability assays can be used, such as fluorescent dyes based on the reaction of esterases in living cells that release fluorescent calcein from a conjugated dye in a similar manner that fluorescein is released from fluorescein diacetate. The other fluorescent dyes used to assay cell viability include for example calcein AM or carboxyfluorescein (carboxyfluorescein diacetate, CFDA). Slides should be viewed as soon as possible after staining.

#### d. Future research directions

Generally, despite strong theoretical expectations, the relationships between the fraction of viable root cells and the ability of roots to perform a range of functions remain largely untested, making the concept of root lifespan ambiguous. Also, little information is known of the relationships between plant cells and fungal hyphae vitality.

### 3. Ratio of absorptive to transport roots

*Ratio of absorptive to transport roots* is the ratio of the number of roots that lack phellem and secondary xylem to these presenting phellem and profuse secondary xylem. This represents the ratio of roots presenting only primary growth and those presenting secondary growth.

Roots exhibit different functions, as some roots are responsible for nutrient and water uptake, while other roots provide transport and structural support functions. Directly determining the function of individual, intact roots can be problematic due to the complexity of root systems, but determining root functioning indirectly can be accomplished via anatomical analysis (discussed by Guo *et al*., 2008a). Determining primary or secondary growth within‐root type and order is an indicator of their function and can be used to approximate differences in root lifespan within a branched root system (Peterson *et al*., 1999; Bagniewska‐Zadworna *et al*., 2012; McCormack *et al*., 2017; Zadworny *et al*., 2017).

**Fig. 17:**
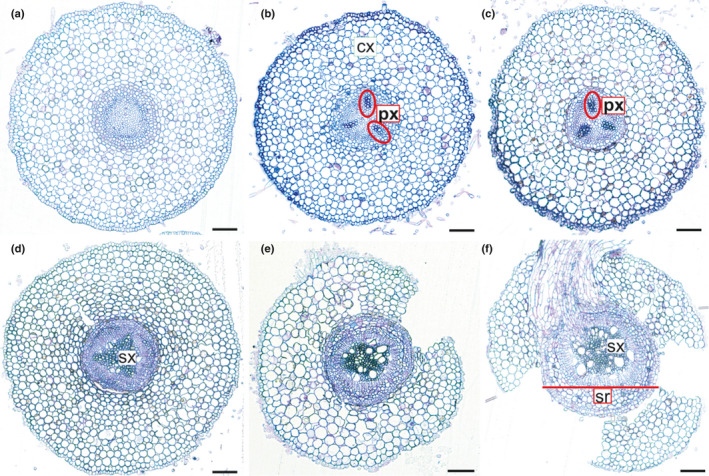
Root cross‐section through different growth zones showing primary (a–c) and secondary (d–f) development of *Populus trichocarpa* roots. Roots were fixed and embedded in Technovit, sectioned using rotary microtome and stained with toluidine blue using the protocol described under section **XIII. 1. a. Generalities of microtechniques used for the analysis of root anatomy**. Traits that can be assessed through such serial images include: root diameter, cortex thickness, cortex and stele area fractions, cortex‐to‐stele area ratio, primary/secondary xylem differentiation distance from root tip, archic structure (here: triarch), the number of xylem poles in primary xylem, number of conductive elements per root section or per xylem pole, conduit diameter, conduit wall thickness, cell wall thickness ratio to conduit diameter, root specific hydraulic conductance (*Lx*), critical tension for conduit collapse (*t/d*)^2^. cx, cortical parenchyma cells; px, primary xylem (within ellipses); sx, secondary xylem; sr, secondary growth of root. Bars, 100 µm.

During primary growth, roots in the region of maturation display a clear separation between their cortex and stele (Fig. 17a–c). Cortex is composed mostly of cortical parenchyma cells. The innermost layer of cortex forms an endodermis and one or more of the outermost layers can differentiate as exodermis. The stele is usually composed of one layer of pericycle, and vascular tissues consisting of phloem and xylem poles, and centrally located parenchyma or sclerenchyma cells. The presence of parenchymatic cortical cells colonised by mycorrhizal fungi indicate their role in nutrient uptake (e.g. Peterson *et al*., 1999). Secondary growth occurs in roots that grow in thickness and possess secondary xylem and phloem vascular tissues arising from vascular cambium that expand in a radial direction (Fig. 17d–f). During secondary growth, the cortex is shed. Therefore, roots with secondary growth possess a periderm composed of phellem (cork tissue), phellogen (cork cambium) and phelloderm (a parenchymatic tissue). Both phellem and phelloderm are derived from cell divisions in the phellogen (cork cambium) outward and inward, respectively (Evert, 2006). Phellem (the outer layer of periderm) tissue restricts water and nutrient absorption due the hydrophobic nature of suberised phellem cells and the loss of cortical parenchyma cells. The development of roots with greater protection from the surrounding environment and a higher investment in secondary growth may be a means to extend root lifespan and decrease root turnover (Wells & Eissenstat, 2001). The production of secondary xylem tissues also changes root primary function from absorption to transport.

#### a. Sampling recommendations

See section **VI. Experimentation and sampling in laboratory and field**.

#### b. Storage and processing

See section **XIII. 1. Microtechnique method for the analysis of root anatomy**. Once anatomical cross‐sections are produced, they can be stored indefinitely and measured at any time.

#### c. Measurement procedure

It is important to obtain measurements from cross‐sections obtained from similar distances from the root tip or half way through the root segment in higher root orders. Root segments should be observed under a light microscope at ×10, ×20 and ×40 objective magnification and then classified as having either primary or secondary growth based on the presence or absence of phellem and profuse secondary xylem (Fig. 17). In addition roots might be observed under ultraviolet (UV) light to ensure that no cork layers were suberised (verified by lack of autofluorescence). The sections of the same root order, collected from comparable distance from its branching from higher root order, have to be compared. Based on the anatomical observations, the fraction of roots with primary or secondary growth (i.e. having absorptive and transport potential) can be calculated within‐root branches, or across the branching hierarchy.

#### d. Future research directions

While previous studies have found substantial intraspecific variation in the ratio of absorptive to transport roots, as dependent on root age and root environment (Guo *et al*., 2008a; Bagniewska‐Zadworna *et al*., 2012; Zadworny *et al*., 2016) the drivers of these changes are still largely unknown. Development of new methods that allow quick identification of primary and secondary root growth would considerably facilitate future research because root system dissection of individual orders and anatomical observation is extremely time consuming.

### 4. Root cortex thickness and density

*Root cortex thickness* is the thickness of the ring of cortical cells beginning outside the stele and extending to the root epidermis (typical units: µm).

*Root cortex cell density* is the number of cortical cells in a radial line (unitless).

Although multiple processes determine the water and nutrient capacity of absorptive roots, the presence of parenchymatic cortical tissues in roots is essential for these activities, as nutrient uptake declines rapidly with the loss of cortical cells (Steudle & Peterson, 1998). The parenchyma cells within the cortex of roots exhibit structural diversification. Therefore, the thickness or number of cell layers present in the cortex of roots are of special physiological importance to root uptake function. The ability to acquire nutrients from the soil for plant growth and development may be highly modulated by adjustments in the characteristics of root cortical tissues, especially in response to diverse environmental conditions (Zadworny *et al*., 2016). For example, Scots pines growing at higher latitudes partially compensate for the reduced availability of nutrients by increasing the thickness of the root cortex, thereby increasing uptake capacity potential (Zadworny *et al*., 2016). In sharp contrast, the radial conductivity of water flow into the roots is negatively correlated with the width of the cortex (Eissenstat & Achor, 1999; Rieger & Litvin, 1999). Yet, parenchymatic cells within the cortex also form a tissue base for colonisation of roots by mycorrhizal fungi, which results in increasing the active surface area available for nutrient absorption (Comas *et al*., 2012). Indeed, plants growing under harsh conditions may greatly benefit from a larger number of parenchyma cells in the cortex of roots that would provide favourable conditions for the establishment of symbiotic associations with fungi, thereby increasing their ability to acquire nutrients from the surrounding soil (Read, 1991; Brundrett, 2002; Ostonen *et al*., 2011; 2013).

#### a. Sampling recommendations

See section **VI. Experimentation and sampling in laboratory and field**.

#### b. Storage and processing

See section **XIII. 1. Microtechnique method for the analysis of root anatomy**. Once anatomical cross‐sections are produced, they can be stored indefinitely and measured at any time.

#### c. Measurement procedure

Root cross‐sections should be observed under a light microscope at ×10, ×20 or ×40 objective magnification. Measurements can be made with assistance from an open‐source image processing software such as imagej (https://imagej.nih.gov/ij/), as well as several commercial software packages (included with the software to make digital images, for example AxioVision or ZEN (Carl Zeiss Microscopy, Jena, Germany), ROXAS (WSL, Birmensdorf, Switzerland)). Calibration is necessary before measurements. Using microscopy software, calibration is made automatically based on defined image scale. Using other software, for example imagej, it is necessary to calibrate the image scale and to specify the unit of measurement. After the analysis of cross‐sections by the software, they should be screened to ensure that the software was able to capture measurements correctly. Importantly, cortical thicknesses are highly dependent on the longitudinal distance from the root tip. Therefore, it is important to obtain measurements from cross‐sections obtained from similar distances from the root tip or half way through the root segment in higher root orders when comparing roots between different species or even within a species, especially when examining root response to adverse environments. Measurements should be obtained from root sections at least in two crossing directions to the highest level of precision (1 µm). Multiple individuals should be measured and then combined to obtain an average value. It is also recommended that several cross‐sections from individual roots are measured. The cortex thickness is typically approximated as all tissue outside the stele (ToS, *sensu* Kong *et al*., 2019), which facilitates its standardised measurement (among experienced and nonexperienced experimenters) and only very slightly overestimates the true cortex area. Indeed, the root epidermis represents a rather negligible fraction of the root cross‐sectional area and is in many cases impossible to differentiate with confidence from the cortex.

#### d. Future research directions

The role of cortex thickness in mycorrhizal colonisation and nutrient acquisition remains largely speculative and quantitative studies linking this trait to mycorrhizal fungi colonisation are necessary to better evaluate the value of this trait across root orders.

### 5. Cortex and stele area fractions

*Root cortex area fraction* is the root cortical area per total area of a root cross‐section (typical units: mm^2^ mm^−2^).

*Root stele area fraction* is the root stele area per total area of a root cross‐section (typical units: mm^2^ mm^−2^).

*Root cortex‐to‐stele area ratio* is the quotient of the cortical area and the stele area of a root cross‐section (typical units: mm^2^ mm^−2^).

The size of cortex and stele are typically compared within a given root order, because they vary strongly across orders (e.g. McCormack *et al*., 2015a). Due to their major role in resource uptake, and the loss of cortex in transport roots, these traits are typically determined for absorptive roots (e.g. first‐order and second‐order roots following a morphometric classification). The area ratio between the cortex, a tissue responsible for water and nutrient absorption, and the stele, which is responsible for resources transport to, and exchanges with, the rest of the plant, represents a crucial factor for understanding the balance between water and nutrient absorption and transportation (Guo *et al*., 2008a). A large cortex area fraction theoretically implies a higher possibility for connection to symbionts by providing larger space for mycorrhizal fungal hyphae and arbuscules. This is likely to increase the plant ability to acquire limiting resources via greater arbuscular mycorrhizal colonisation intensity (Comas *et al*., 2012; Kong *et al*., 2017) and therefore higher fungal biomass per unit of root mass (Valverde‐Barrantes *et al*., 2016). However, while the construction of more cortex tissue could enhance nutrient absorption via mycorrhizal acquisition, it may require longer root lifespan to amortise the cost of producing cortical parenchyma cells (Weemstra *et al*., 2016; Kong *et al*., 2017). Despite this, a large cortex area fraction has sometimes been related to faster root turnover (Guo *et al*., 2008a,c). A low cortex area fraction implies a shorter path for water and less impedance to water movement, favouring water stress tolerance under dry conditions (Eissenstat & Achor, 1999; Comas *et al*., 2012). Therefore, the anatomical composition of the roots can be used to explore mechanisms underlying root responses to different environment stresses and the evolutionary relationships developed over time with fungal partners (Kong *et al*., 2017).

Whereas the root cortex‐to‐stele area ratio has been the trait most studied so far, future studies should put a greater focus on the separate use of the two component traits of this ratio, that is the cortex area fraction and the stele area fraction (e.g. Kong *et al*., 2019). First, these two fractions focus on one specific tissue each and can therefore be more directly linked to the specific function(s) of these tissues. Second, variations in fractions more faithfully and directly describe variations in root ecophysiology as they do not have the same mathematical property as ratios, which vary disproportionately above and below a ratio of 1.0.

We note that all of the cortex‐to‐stele area ratio and cortex and stele fractions can reflect changes in either the size of the cortical or the stelar tissue, compared to, for example, direct measurement of cortex thickness (see section **XIII. 4. Root cortex thickness and density**). Kong *et al*. (2019) showed that in woody plants cortical area was the most variable part influencing total root diameter, whereas in herbaceous plants the stele varied more than the cortical tissue. Therefore, researchers must take caution of the interpretation of changes in any of these traits depending on the specific group of plants studied.

#### a. Sampling recommendations

See section **VI. Experimentation and sampling in laboratory and field**.

#### b. Storage and processing

The cortex and stele fractions of roots can be assessed using two methods. The first method follows the section **XIII. 1. Microtechnique method for the analysis of root anatomy** and is specifically recommended for researchers interested in the assessment of several anatomical traits.

The second method is less labour intensive and can be combined with assessment of mycorrhizal traits. It is based on root staining and transparency rather than on root sectioning. With this method, washed roots should be processed immediately. Roots or root fragments are cleared, acidified and stained as described under section **XIX. 2. Root mycorrhizal colonisation intensity** until a sharp contrast between the (dark‐blue) stele and the (unstained) cortex along the lateral axis of the root is clearly visible under a stereomicroscope. Roots are then mounted lengthwise on microscope slides with polyvinyl alcohol (PVA). A cover slip is gently laid down on the root from one side to another to avoid air bubbles without pressing the root which would lead to deformations. The slides with the coverslips on top are left to rest overnight to solidify the mounting medium.

#### c. Measurement procedure

For the first method, see section **XIII. 4. Root cortex thickness and density** measurement procedure. For the second method, lateral views of the stained roots allow measuring the thickness of the stele and the cortex without having to incise the root. These measurements can be done with a stereomicroscope and micrometre, or from microscope images with image analysis software (e.g. imagej). Similar to root cortex thickness measurements, the cortex area is typically approximated as all ToS (*sensu* Kong *et al*., 2019), which facilitates its standardised measurement (among experienced and nonexperienced experimenters) and only very slightly overestimates the true cortex area, as mentioned above.

Similar to the measurement of previous anatomical properties, a comparison of roots requires a standardised position (e.g. a similar distance from the branching of higher‐order roots) of cortex and stele measurements and multiple root replicates are typically needed.

##### Calculations

Regarding the first method, direct measurements of root cortex and stele area can be done by analysing microscope images with image analysis software. These will give the most accurate results as roots are not always circular. Alternatively, when calculating area based on root and stele diameter, at least two perpendicular measurements should be taken. This is not possible in the second method, where area calculations can only be based on one measurement of root and one for stele diameter.

#### d. Future research directions

The links between root cortex and/or stele area fractions and root economics (lifespan, respiration) and the nutrient‐acquisition strategy (relative reliance on mycorrhiza) is to date based on very few studies and further research is critically needed to more clearly establish the potential key role of this trait in multiple aspects of root functioning. As the second method (on intact root fragments rather than sectioned roots) allows quantification of mycorrhizal colonisation (see section **XIX. 2. Root mycorrhizal colonisation intensity**) on the same roots, these two below‐ground properties can be measured simultaneously to improve our insights concerning the relationships between root anatomical, morphological and mycorrhizal traits.

### 6. Fraction of passage cells in exodermis

*Fraction of passage cells in exodermis* is the number of passage cells relative to the total number of exodermis cells in the root perimeter (unitless).

In herbaceous plant roots, the dermal tissue, rhizodermis, usually remains intact for the entire life of the plant and becomes encrusted with suberin, which serves a protective function. By contrast, the rhizodermis in the fine (nonwoody) roots of woody plants is usually short‐lived and replaced by an underlying hydrophobic tissue called the exodermis. The exodermis is composed of very compact cells, and possesses a Casparian strip and suberin lamellae. These latter two features restrict the flow of water through the symplast rather than through the apoplast, so that water must pass through cell membranes rather than solely through cell walls (Endstone *et al*., 2003; Ma & Peterson, 2003). The exodermis prevents water loss from root tissues to the surrounding soil, serves as a defence against microorganisms, provides mechanical support for root tissues, and isolates living, cortical parenchyma cells from exterior soil particles (Enstone & Peterson, 1992; Kamula *et al*., 1994; Huang & Eissenstat, 2000; Taylor & Peterson, 2000; Sharda & Koide, 2008). The outer layer of the exodermis, however, serves not only as a barrier to water and nutrient transport within absorptive roots, but also to facilitate the passage of water and nutrients into the root. This dual function occurs through cell type diversity. One type of exodermis cell has secondary wall thickenings, that is suberin lamellae, which are fluorescent under UV, while another type includes passage cells that lack suberin lamellae. These passage cells may be characterised by the presence of Casparian bands, or can be short cells that possess Casparian bands only in their anticlinal walls (Enstone *et al*., 2003). Variability in the distribution of the different cell types in roots is dependent on root type, root morphology, and edaphic factors (Eissenstat & Achor, 1999; Hishi *et al*., 2006; Kirfel *et al*., 2017). Passage cells in absorptive roots are sites where nutrients are exchanged between roots and their surrounding environment. Therefore, identifying the location and density of passage cells and root hair cells (see section **XXII. Root hair morphology and development**) gives insights into the passage of resources and resource acquisition capacity of a root. However, it should be noted that not all species produce an exodermis (e.g. Lambers *et al*., 2018).

#### a. Sampling recommendations

See section **VI. Experimentation and sampling in laboratory and field**.

#### b. Storage and processing

Two different staining protocols can be used to distinguish passage cells with light microscopy. The type of stains and staining methods utilised may provide different results depending on the root type and plant species. The first protocol to distinguish passage cells is based on the autofluorescence of lignified cell walls, Casparian bands and suberin lamellae; all of which absorb UV light, as well as the visible light spectrum. To identify and count the number of passage cells, cross‐sections of roots are obtained as described under section **XIII. 1. Microtechnique method for the analysis of root anatomy**. Then, 5–10‐μm thick cross‐sections of roots are stained with 0.5% toluidine blue O in 1% sodium tetraborate (pH 4.4) buffer as described by Feder and O'Brien (1968).

As an alternative to the traditional identification of passage cells within the exodermis, Casparian bands within exodermal cells can be specifically stained (Brundrett *et al*., 1988). Treatment of cross‐sections with a berberine stain that is more resistant to photobleaching can lead to more consistent results. This approach requires quenching the autofluorescence of roots by aniline blue and enhances the specific localisation of suberin and lignin berberine. Due to the negative effect of fixation and embedding procedures on cell wall permeability, fresh cross‐sections of living, unfixed roots are preferred for this. Sections obtained with a vibratome can be stained with 0.1% berberine hemi‐sulphate in water and after several rinses with distilled water, counterstained with 0.5% aniline blue in water for 30 min. After several rinses with distilled water, the sections should be mounted on a microscope slide with 0.1% FeC1_3_ in 50% glycerin. Sections should then be observed under UV light within a few hours after staining (Brundrett *et al*., 1988).

#### c. Measurement procedure

**Fig. 18:**
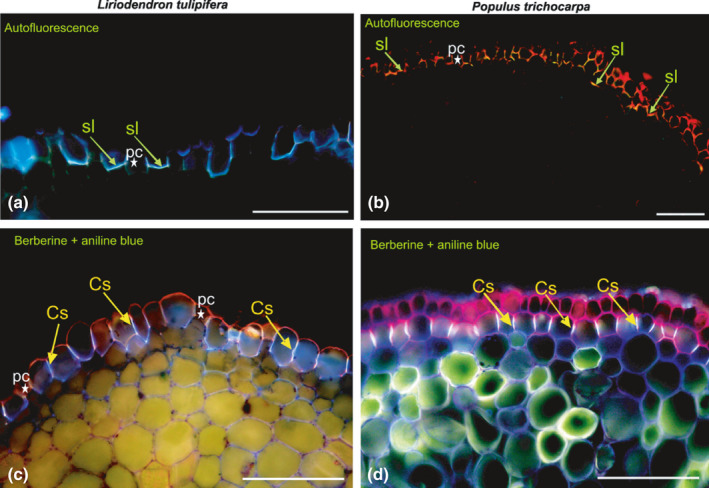
Visualisation of exodermis in *Liriodendron tulipifera* and *Populus trichocarpa* roots; exodermis autofluorescence (a, b) and root sections stained with berberine and aniline blue (c, d). Cs, Casparian strips; sl, suberin lamellae; pc, passage cells (asterisks). Bars, 100 µm.

Cross‐sections of root tissues should be observed under UV light (excitation wavelength 365 nm). The intensity of excitation utilised should be the same for each sample, especially when comparative studies are being conducted. The autofluorescence of exodermis cell components will rapidly lose their fluorescence due to photobleaching if too strong of a UV light is utilised. Autofluorescence of the cell walls and Casparian bands deposited in the anticlinal walls distinguishes passage cells from exodermal cells that are impermeable to water and nutrients (Fig. 18).

Using the aniline blue and berberine staining protocol, Casparian bands in the exodermis stain an intense yellow‐white, and suberin lamellae in the exodermis stain a modest blue‐white or blue. The form of suberin in the Casparian bands of passage cells can be distinguished from suberin lamellae (Brundrett *et al*., 1988). The fraction of passage cells in each root cross‐section should be calculated as the number of exodermal passage cells relative to the total number of exodermis cells in the root perimeter. Depending on the aim of the study, the fraction of passage cells might be normalised by the length of the root perimeter.

#### d. Future research directions

The relative abundance of passage cells theoretically shapes root foraging strategies through the regulation of water and nutrient movement, as well as mycorrhizal colonisation. However, demonstration of the relevance of this traits and its variation across root ontological stages and across species requires further testing at the intraspecific and interspecific level.

### 7. Xylem developmental stage and anatomical features

Xylem developmental stage is the degree of differentiation and development of the water and nutrient conducting tissues. Its development and anatomical features can be assessed through several metrics:

*Distance of primary xylem differentiation from root tip (typical units: mm)*.

*Distance of secondary xylem differentiation from root tip* (typical units: mm).

*Number of xylem poles* (unitless).

*Number of conduits* (vessels and tracheids) (unitless).

*Conduit lumen diameter* (typical units: µm).

*Conduit wall thickness* (typical units: µm).

*Cell wall thickness to conduit lumen diameter ratio* (typical units: %).

Xylem is the vascular tissue responsible for the conduction of water and nutrients in terrestrial plants from the roots to the stems and leaves. Root xylem is a complex, heterogeneous tissue, whose structure and composition varies with species, root type, root functional divergence, distance from the root apical meristem, and environmental conditions. The differentiation and maturation of root tissues can be divided into several stages leading to the formation of mature functional tissue (Fig. 17). Xylem tissue is classified as primary or secondary depending on the origin of the developing xylem cells.

#### Primary xylem

Primary xylem is the xylem that arises from the procambium during primary growth (Evert, 2006). It includes two types of conductive cells, protoxylem and metaxylem, which vary in their diameter and rate of differentiation: metaxylem develops first but matures after protoxylem; metaxylem cells are wider conducting elements (vessels or tracheids) than protoxylem; and a cross‐section of a typical primary root (e.g. first‐order roots) reveals primary xylem together with phloem arranged alternately in a central vascular cylinder (Bagniewska‐Zadworna *et al*., 2012, 2014a,b) whereas metaxylem is arranged centripetally in relation to the outer protoxylem. Both conducting elements – that is protoxylem and metaxylem – are characterised by distinct lignified secondary cell wall thickenings (Fig. 17b,c) that provide biomechanical support: protoxylem typically forms annular and helical thickenings, while metaxylem forms strongly lignified cell walls with scalariform, reticulate, and pitted thickenings (Evert, 2006). Formation of fully functional xylem conducting elements ensures the undisturbed flow of water after its absorption by root hairs and fine roots and its immediate transfer to higher‐order roots for transport to the above‐ground parts of a plant. It is assumed that the axial hydraulic resistance within xylem is not rate‐limiting once metaxylem cells are formed. The number of xylem conducting elements (vessel elements and tracheids) in xylem tissue impacts the transport of water and nutrients. The number of xylem ridges in primary roots varies between species and within roots of the same species, forming diarch, triarch, tetrarch, etc. roots that manifest their wider transport ability, and is also an important index used to classify the life cycle of individual roots (Hishi & Takeda, 2005).

#### Secondary vascular tissue

Secondary vascular tissue refers to the formation of vascular tissues (secondary xylem and secondary phloem) from the vascular cambium, which is a meristematic layer of cells in roots to produce secondary growth. It is referred to as secondary growth because these cells were not present during the formation of primary plant structure. The vascular cambium arises between the primary xylem and phloem. Additionally, pericycle cells divide simultaneously with procambium initials. As a result, a cylinder of cambium is formed (Fig. 17d–f). Once formed, the vascular cambium undergoes periclinal cell divisions; producing xylem cells inward and phloem cells toward the outside of the root. Secondary xylem is composed of tracheids, vessel elements, lignified fibres and interspersed, nonlignified xylem parenchyma cells. Mature conducting elements with strongly lignified cell walls, but absent transverse end walls, are perfect conduits for water and nutrient transport in roots. Fibres are dead cells that do not possess a protoplast at maturity and therefore mainly function in mechanical support.

Vascular tissue differentiation in roots governs water and nutrient movement through individual roots. The lack of secondary xylem decreases the efficiency of water uptake and transport of the root (Eissenstat & Achor, 1999; Huang *et al*., 2010). However, there can be a great degree of variability in the amount of xylem cells produced in different roots of the same tree (Zadworny & Eissenstat, 2011; Bagniewska‐Zadworna *et al*., 2012). Variation in xylem differentiation and maturation in roots of individual plants, as well as differences in the ages of individual roots in different trees, play an essential role in determining axial hydraulic conductivity. The age of individual roots can be determined by assessing tissue development, mainly in terms of the amount of primary vs secondary xylem formation (Hishi, 2007). There is a magnitude of difference in conductivity of primary xylem tissues among species, suggesting that a transport bottleneck in developing roots is avoided by a greater investment in both the number and diameter of conduits in primary xylem tissues and the rapid development of secondary vascular tissue (Bagniewska‐Zadworna *et al*., 2012).

#### a. Sampling recommendations

See section **VI. Experimentation and sampling in laboratory and field**.

#### b. Storage and processing

Anatomical observations of serial sections of roots embedded in paraplast/paraffin are useful for examining the sequence of events that occurs during xylem differentiation and maturation. Double staining sections with Safranin O and Fast green allows for observation of lignified tissue that are stained red and cellulose cell walls that are green. Vibratome sections can be stained with phloroglucinol which will stain lignified cell walls dark red/purple in the presence of alcohol and 20% HCl. Paraplast/Technovit embedded sections or vibratome sections can be examined under a fluorescence microscope (simple UV excitation is sufficient) to evaluate cell wall lignification/suberisation. Lignified cell walls appear violet when they autofluoresce at UV, 330–450 nm; yellow at 470–535 nm, and green above 530 nm.

#### c. Measurement procedure

See section **XIII. 4. Root cortex thickness and density** for details on measuring these anatomical properties. Microtome sections, examined with light microscopy, should be measured to quantify the number of xylem poles, conductive elements and to measure conduit diameter or cell wall thickness of xylem cells in roots. Alternatively, observations made with transmission electron microscope can be used.

#### d. Future research directions

Xylem development and structure underlies water acquisition capacity, hydraulic failure, water transport limitation and root lifespan, but few direct tests of their linkages to plant response to water stress have been made across a range of species to quantify their respective contribution to this process.

### 8. Critical tension for conduit collapse *(t/d)^2^*

*Root critical tension for conduit collapse* is the squared value of the ratio of the cell wall thickness to the ‘hydraulic weighted mean lumen diameter’ of vessel elements or tracheids (unitless) (frequent abbreviation: (*t*/*d*)^2^).

Euler buckling theory suggests that the critical tension resulting in conduit collapse is constant when cell wall thickness (*t*) scales proportionately with hydraulic weighted mean lumen diameter (*d*) (Hacke *et al*., 2001; Blackman *et al*., 2010). As such, the ratio of *t/d* (assuming proportional scaling) represents the premium a species places on avoiding conduit collapse. Species can be compared with evaluate potential differences in cavitation resistance of their root xylem as indicated by (*t*/*d*)^2^ (Hacke *et al*., 2001). Additionally, (*t*/*d*)^2^ and root specific hydraulic conductance (see below) can also be examined among species to identify potential trade‐offs in risk aversion and capacity for water movement.

#### a. Sampling recommendations

See section **VI. Experimentation and sampling in laboratory and field**.

#### b. Storage and processing

See section **XIII. 1. Microtechnique method for the analysis of root anatomy**.

#### c. Measurement procedure

See section **XIII. 4. Root cortex thickness and density**. Microscopy software should be used to measure conduit diameter or cell wall thickness of root xylem cells. Hydraulic weighted mean conduit diameter (*d*) is measured as the lumen diameter of vessel elements or tracheids, hydraulically weighted to present the measurement proportionally to its effect on hydraulic conductance (Sperry *et al*., 1994). It can be determined by measuring the lumen radius of all conduits in a cross‐section or growth ring, and applying the following equation: *d = 2(Σr^5^/Σr^4^)* (Sperry *et al*., 1994), where *r* is the lumen radius. The cell wall is not included in the diameter. For coniferous species, the fact that tracheid lumens are square is taken into account by using a different equation for measurement of the hydraulic weighted mean conduit diameter: *d* = (*Σa)^0.5^*, where *a* is lumen area.

Cell wall thickness (*t*) is measured as the thickness of the cell wall of an individual conduit. It should be measured on at least five conduits per root cross‐section or growth ring. To measure wall thicknesses, the distance between two opposite edges of the cell wall is measured.

#### d. Future research directions

Despite the immediate relevance of this trait, the legacy of drought events on this trait, including the plasticity of both *t* and *d* in response to interannual environmental changes, remains largely unknown.

### 9. Root specific hydraulic conductance (*Lx*)

*Root specific hydraulic conductance* is the calculated amount of water that can move axially through tissue per the cross‐sectional area of the tissue (typical units: m^3^ s^−1^ MPa^–1^ cm^−2^) (frequent abbreviation: *Lx*).

The theoretical/calculated root specific hydraulic conductance for individual roots within each root branching order is estimated using the Hagen–Poiseuille equation. The trade‐offs between this axial hydraulic conductance and cavitation resistance may be of special importance for understanding plant adaptation to different environments. Within large xylem conduits, a greater hydraulic conductance can come at a cost of increased risk of cavitation according to the Hagen–Poiseuille law (Hacke *et al*., 2001), but species‐specific strategies are also important for assessing these trade‐offs (Gleason *et al*., 2016). Additionally, plasticity in root *Lx* may be associated with different strategies among plants and important for success in different habitats (Zadworny *et al*., 2018).

#### a. Sampling recommendations

See section **VI. Experimentation and sampling in laboratory and field**.

#### b. Storage and processing

See section **XIII. 1. Microtechnique method for the analysis of root anatomy**.

#### c. Measurement procedure

See section **XIII. 4. Root cortex thickness and density**. Calculations are made from anatomical cross‐sections. *Lx* for individual roots can be estimated using the Hagen–Poiseuille equation (Valenzuela‐Estrada *et al*., 2009): *Lx* = π × *n*_v_×(*R*_a_^4^)/8 × η × SA.

where *n*_v_ is the mean number of xylem conduits per root, *R*_a_ is the average weighted conduit radius in m, η is the viscosity of water (1 × 10^−9^ MPa s at 20°C), and SA is a root cross‐sectional area in cm^2^.

## 14 Root chemistry

The term 'root chemistry' encompasses both the elemental chemistry and molecular chemistry of roots. In general, roots are primarily composed of 17 nutrient elements, with carbon (C), oxygen (O) and hydrogen (H) derived from the atmosphere and soil water, and the other 14 elements taken up by the roots from soils (Barker & Pilbeam, 2007). Six elements are considered as macronutrients for plants (i.e. required at concentrations > 0.1 mg g^−1^): calcium (Ca), potassium (K), magnesium (Mg), nitrogen (N), phosphorus (P), sulfur (S); and eight elements as micronutrients (i.e. essential elements required only in small quantities): boron (B), chlorine (Cl), copper (Cu), iron (Fe), manganese (Mn), molybdenum (Mo), nickel (Ni), zinc (Zn) (Barker & Pilbeam, 2007). A few additional elements are considered beneficial elements to plant functioning: cobalt (Co), iodine (I), sodium (Na), selenium (Se), silicon (Si), vanadium (V) (Barker & Pilbeam, 2007; Epstein, 2009). These elements are involved in various biological reactions and form part of the molecules that fulfil multiple functions within plants. In turn, these molecules can be a part of different cell components and compartments such as cytosol, cell organelles or as cell walls; can form metabolites such as sugars, organic acids, amino acids, nucleic acids, phenolics, lipids, or aliphatics; or can be linked together in macromolecules such as starch, cellulose, hemicellulose, pectin, lignin, suberin, proteins, DNA and RNA. Below, we highlight a small selection of root chemical traits that we consider having most relevance for a range of plant and ecosystem functions (see also Freschet *et al*., 2021).

Root chemistry may vary spatially and temporally. For example, fine‐root N concentrations can vary across seasons in temperate environments (Zadworny *et al*., 2017), and vary with root age and soil depth (Freschet & Roumet, 2017). This clearly represents a problem when scaling measurements of root nutrients from a single location and time to represent an entire fine‐root system throughout the year, as is often done in empirical and modelling studies (McCormack *et al*., 2017).

Root stoichiometry refers to the relative quantities or ratios of different elements in root biomass. In plants, such relative proportions among elements is important for their growth performance (e.g. Sprengel–Liebig’s law of the minimum; van der Ploeg *et al*., 1999; Ågren, 2008). The roles of macronutrients and micronutrients in plants are well understood. However, the relative requirements of the elements are poorly quantified, and dependencies among elements are not well investigated. The most investigated relationship is that of C : N : P, because N and P commonly limit growth, and C provides the structural basis of root biomass (Ågren, 2008). Despite the variability in biomes, plant species and soil types, there are global patterns in fine‐root N, P and N : P ratio along latitudinal gradients (Yuan *et al*., 2011). These biogeographic gradients are likely to result from the collective influences of complex interactions between climate, soil and biological factors (Yuan *et al*., 2011) and most particularly changes in the age of the soil, with P becoming increasingly limiting with increasing soil age (Walker & Syers, 1976).

### 1. Root nitrogen concentration

*Root N concentration* (formal name: *Root N content per dry mass*) is the mass of N per root dry mass (typical units: mg g^−1^) (frequent abbreviation: root N concentration (RNC)).

Nitrogen is a macronutrient and occurs in roots in concentrations of 5–35 mg g^−1^ (Iversen *et al*., 2017), with a mean value on a global scale of *c*. 10–11 mg g^−1^ (Gordon & Jackson, 2000; Yuan *et al*., 2011). N is the most abundant nutrient element in roots, in addition to C, O and H. The major portion of N in plants is in proteins, which contain *c*. 85% of the total N in plants (Barker & Pilbeam, 2007). Nucleic acids contain *c*. 5% of the total N, and the remaining 5–10% of the total N is in low‐molecular‐weight, water‐soluble, organic compounds of various kinds (Barker & Pilbeam, 2007). Proteins can either act as enzymes or, alternatively, as structural, receptor, translocator or storage proteins.

Roots take up N as nitrate or ammonium, but under normal aerated soil conditions, the main N form is nitrate (Barker & Pilbeam, 2007). However, roots can also take up amino acids, and even small peptides. For nitrate, to be used in the synthesis of proteins and other organic compounds, it must first be reduced to ammonium, metabolised into amino acids, and then assimilated into proteins or N‐containing macromolecules (Barker & Pilbeam, 2007). Most of the ammonium is metabolised in the roots to organic compounds such as amino acids, nucleic acid, nucleotides, coenzymes or proteins. However, some plant species such as grasses reduce most of the nitrate to ammonium in their leaves (Scheurwater *et al*., 2002). In contrast with ammonium, nitrate is mobile and can be stored in the vacuoles of the roots or transported to shoots. Similar to leaf N concentration, phenotypic variation in RNC is strongly related to plant‐available soil N concentration (e.g. Freschet *et al*., 2018) which suggests a potential role of RNC as a proxy for the N status of plants.

The highest RNCs can be expected in the first‐order roots (root tips) or the finest roots (i.e. < 0.5 mm diameter). Globally, mean values decrease from 19 mg g^−1^ N for first‐order roots, to 16 mg g^−1^ N for second‐order roots, 13 mg g^−1^ N for third‐order roots, and 10 mg g^−1^ N for third‐order roots (Iversen *et al*., 2017). The high N concentrations of the root tips are related to their fast metabolic rates, as they host the meristems, where cell division and elongation occur, and where root cap cells, border cells, mucilage, and exudates are lost or excreted (Jones *et al*., 2009). Moreover, root tips are often colonised by mycorrhizal fungi that may further add to their N concentrations. Indeed, cell walls of fungal hyphae (mycorrhizal mantle, Hartig net) contain 100–200 mg g^−1^ chitin, a long linear polymer of *N*‐acetylglucosamine (Bowman & Free, 2006), so that fungal hyphae contain 20–40 mg g^−1^ N.

Worldwide, first‐order roots have a mean N concentration of 19 mg g^−1^ (mostly ranging between 10 and 28 mg g^−1^ N) that does not differ among biomes. However, RNC differs between herbaceous and woody species (Ma *et al*., 2018). Root N concentration is weakly positively correlated with root C concentration (Ma *et al*., 2018) and strongly with root respiration rate (Reich *et al*., 2008; Roumet *et al*., 2016).

Root N concentration is an important driver of C and nutrient cycling. Plant productivity of natural ecosystems is often limited by N, because N is not derived from rock weathering, but from N_2_ fixation through symbiotic or free‐living soil microbes, or from wet or dry deposition. In this view, the recycling of N is important for the cycling of most elements, and soil microorganisms and ectomycorrhizal fungi are most relevant for the mobilisation and uptake of organic N (Philippot *et al*., 2013).

#### a. Sampling recommendations

See section **VI. Experimentation and sampling in laboratory and field**.

#### b. Storage and processing

See section **VII. Root washing, sorting and storage**. At the end of the processing, the fresh roots and the root fractions of interest are oven dried (60°C) for 3 d. The temperature of drying is based on the compatibility with the chemical analyses. Alternatively, roots can be freeze dried. Dried root material is then milled to a fine powder, and stored under dry conditions until further analyses.

#### c. Measurement procedure

Here, 2–4 mg of dry, milled root material is weighed into small tin containers (3.2 × 6 mm) using an analytical microbalance and forceps. The tin containers are then combusted by external oxygen flash combustion at 1050°C. The gaseous combustion products containing N (N_2_, NO_x_) are carried by helium (He) as a carrier gas through a Cu‐column, and are converted to N_2_ (Boudouard reaction); N is finally measured by gas chromatography with thermal conductivity detection. The gaseous conversion and the gas measurement occur in a CN elemental analyser (see also Herzog *et al*., 2014).

#### d. Future research directions

Compared with the role of N in leaf physiology, the variation among plant species in the role and distribution of N among compounds and tissues remains largely unknown. This generally limits the use of RNC as a proxy for other root functions depending on N.

### 2. Root carbon concentration

*Root C concentration* (formal name: *Root C content per dry mass*) is the mass of C per root dry mass (typical units: mg g^−1^) (frequent abbreviation: root C concentration (RCC)).

Carbon is, next to O and H, the main element in roots and occurs in most cases at concentrations between 340 and 550 mg g^−1^ in fine roots (Freschet *et al*., 2017). Plants take up C as CO_2_ via photosynthesis in the leaves, convert it into sugars and transport it via the phloem to the roots mainly as sucrose, although roots can also take up C from the rhizosphere as amino acids and small peptides, where C forms a backbone. Sucrose is either respired or metabolised to primary or secondary metabolites. From these metabolites, macromolecules such as polysaccharides and polyphenols are formed. Most of the root C is sequestered in cell walls as cellulose, hemicellulose, pectin, and lignin (Ågren, 2008). Carbohydrates are the dominant C compounds in roots. The carbohydrates are usually separated into structural carbohydrates (including cellulose and hemicellulose) and nonstructural carbohydrates (NSC, including soluble sugars, starch and fructans), for which specific measurement protocols are provided below.

Mean C concentration in roots usually do not vary strongly across species and environments (Roumet *et al*., 2016), but has been found to correlate with other root traits such as SRL and RTD at a global scale (Ma *et al*., 2018). A strong relationship occurs also with N concentration, with average C : N ratio of 43 : 1 to 62 : 1 for fine roots (Gordon & Jackson, 2000; Yuan *et al*., 2011) and 25 : 1 for first‐order roots (Ma *et al*., 2018).

Root C concentration, together with root lifespan (Brunner *et al*., 2013; Chen & Brassard, 2013) influences many ecosystem functions such as C cycling, C sequestration, climate regulation, provisioning of food, fibre and fuel, and habitat for organisms (Bardgett *et al*., 2014).

#### a. Sampling, storage and processing recommendations

See section **XIV. 1. Root nitrogen concentration**.

#### b. Measurement procedure

See section **XIV. 1. Root nitrogen concentration**.

### 3. Root phosphorus concentration

*Root P concentration* (formal name: *Root P content per dry mass*) is the mass of P per root dry mass (typical units: mg g^−1^) (frequent abbreviation: root P concentration (RPC)).

Phosphorus in fine roots occurs in concentrations of 0.6–1.4 mg g^−1^ (Brunner *et al*., 2002; Genenger *et al*., 2003), and with a mean value on a global scale of 0.8–0.9 mg g^−1^ (Gordon & Jackson, 2000; Yuan *et al*., 2011). Roots take up P as orthophosphate, either as H_2_PO_4_^–^ or as HPO_4_^2–^ ions depending on pH. In plants, most organic P is associated with ribosomal RNA, which is required for protein synthesis. P is also used for biochemical energy transfer processes, with the energy being released when a terminal phosphate is split from adenosine triphosphate (ATP). Beyond that, P occurs in phospholipids, nucleic acids, nucleotides, coenzymes, and phosphoproteins (Barker & Pilbeam, 2007). Phytate (i.e. phytic acid) is an inositol hexakisphosphate and a principal storage form of P in plants, especially in seeds.

For similar reasons as RNC, the lowest order roots generally have the highest P concentration. Cells of mycorrhizal fungi, and in particular their vacuoles, which colonise the root tips contain abundant quantities of poly P, which is an inorganic polyphosphate consisting of linear chains of phosphoanhydride‐linked phosphate residues (Werner *et al*., 2007), which add to the P concentration of colonised roots. The total poly P concentrations in fungal hyphae can vary strongly from 9 µg g^−1^ to 1.8 mg g^−1^ FW (Werner *et al*., 2007).

Root P concentration can have a strong influence on nutrient cycling. Plant productivity in natural ecosystems is often limited by P, because P input into soils mainly originates from rock weathering (e.g. apatite) if it is a component of the parent rock. Therefore, recycling of P from plant litter is important, and here ectomycorrhizal fungi contribute significantly with their phosphatases to the mobilisation of organic P by converting organic P to inorganic P (Philippot *et al*., 2013).

Phosphorus concentration in fine roots is tightly linked with N concentration via the need for ribosomal ribonucleic acids in protein formation (Gordon & Jackson, 2000; Yuan *et al*., 2011).

#### a. Sampling, storage and processing recommendations

See section **XIV. 1. Root nitrogen concentration**.

#### b. Measurement procedure

A minimum of 50 mg, but ideally 200 mg, of dry and ground root material has to be solubilised by high‐pressure digestion in a high‐pressure microwave oven in polytetrafluoroethylene (PTFE) tubes. The dried material is digested at 12 MPa and 240°C for 10 min in 2 ml of a HNO_3_ (40%)/HF (1.3%) (v/v) solution and then diluted with 10 ml H_2_O. To measure the root elements, the dissolved samples are determined using inductively coupled plasma–optical emission spectrometry (ICP–OES). This technique has multielement and high‐throughput options (Al, B, Ba, Ca, Cr, Cu, Fe, K, Mg, Mn, Na, Ni, P, Pb, S, Zn are in a good range; Cd, Co, Mo are usually below the detection limits) (A. K. Richter *et al*., 2011). As the measurement of P requires ample amount of dried root material, this may not be realistically measured on separate root orders.

### 4. Root potassium concentration

*Root K concentration* (formal name: *Root K content per dry mass*) is the mass of K per root dry mass (typical units: mg g^−1^).

Potassium occurs in roots in concentrations between 0.4–5.4 mg g^−1^ (Brunner *et al*., 2002; Genenger *et al*., 2003) with a mean of *c*. 2.8 mg g^−1^ (Gordon & Jackson, 2000). Roots take up K as K^+^. Potassium ions cycle via the xylem from roots to above‐ground plant parts, and via the phloem from leaves to roots. During growth, the primary root meristem needs K for stimulating plasmalemma ATPases that produce the necessary conditions for the transfer of metabolites from the phloem, e.g. sucrose and amino acids (Barker & Pilbeam, 2007). A high K concentration is required in the cytosol for protein synthesis and in the vacuoles for cell expansion in growing tissues, with K in the vacuoles not only serving as K storage but also functioning as an osmotic substance (Barker & Pilbeam, 2007).

Although K is one of the most abundant elements of the Earth’s crust, K can leach beyond the rooting zone, because it is highly mobile in soil (Alfaro *et al*., 2004). Therefore, the availability of K to plants is usually limited leading to reductions in plant growth and yield (Hafsi *et al*., 2014). A shortage of K in plants leads to browning and curling of leaf tips, as well as chlorosis between the veins. Much remains unknown about the physiological and molecular mechanisms by which plants detect and respond to changes in K concentrations in their environment (Wang & Wu, 2013).

#### a. Sampling, storage and processing recommendations

See section **XIV. 1. Root nitrogen concentration**.

#### b. Measurement procedure

See section **XIV. 3. Root phosphorus concentration**.

### 5. Root manganese concentration

*Root Mn concentration* (formal name: *Root Mn content per dry mass*) is the mass of Mn per root dry mass (typical units: mg g^−1^).

Manganese is a micronutrient occurring in fine roots in concentrations of 0.1–0.8 mg g^−1^ (Brunner *et al*., 2002; Genenger *et al*., 2003). Roots take up Mn as Mn^2+^; however, the availability of Mn for plant uptake is affected by soil pH with a good availability at pH 4.5–6.5. In plants, Mn is involved in many biochemical functions, primarily acting as an activator of enzymes involved in photosynthesis, respiration, amino acid, lignin, and hormone synthesis (Barker & Pilbeam, 2007). Within plant litter, Mn stimulates lignin degradation through the formation of Mn peroxidases involved in lignin oxidation (Keiluweit *et al*., 2015a).

#### a. Sampling, storage and processing recommendations

See section **XIV. 1. Root nitrogen concentration**.

#### b. Measurement procedure

See section **XIV. 3. Root phosphorus concentration**.

### 6. Root nonstructural carbohydrate concentration

*Root nonstructural carbohydrate concentration* (formal name: *Root nonstructural carbohydrate content per dry mass*) is the mass of carbohydrate molecules that do not participate in root structure per root dry mass (typical units: mg g^−1^).

*Total below‐ground nonstructural carbohydrate storage* is the mass of carbohydrate molecules that do not participate in the structure of plant tissues found in below‐ground plant organs (typical units: g).

Nonstructural carbohydrates (NSC) is the term used to describe different carbohydrate molecules (monosaccharides, sugar alcohols, disaccharides, oligosaccharides, polysaccharides) that are not forming structure of below‐ground organs (as opposed to e.g. cellulose and hemicellulose), but may be stored and reused according to plant needs.

Nonstructural carbohydrates are key regulators of the physiological adjustment of plants to environmental stress (Körner, 2003; Dietze *et al*., 2014). In addition, NSCs provide substrate for growth and metabolism. The NSC level of a plant is an important indicator of the C source and sink capacity, and can further inform on plant growth, buffering capacity, and adaptative strategies (W. Liu *et al*., 2018). Nonstructural carbohydrates are particularly useful for plants to support regrowth after seasonal dormancy and regeneration after disturbance (Janeček & Klimešová, 2014) or to sustain the demands of metabolically active organs, for instance absorptive roots (Aubrey & Teskey, 2018). Therefore, quantitative studies of the contribution of NSC to the C balance are important to understand the survival and growth of plants (W. Liu *et al*., 2018). The NSC concentration in roots tends to be high in actively growing roots that are involved in absorptive functions due to a higher metabolic demand. Nonetheless, storage organs (e.g. specialised organs such as taproots, rhizomes, tubers and bulbs) often represent the bulk of plant NSC storage (Pausas *et al*., 2018). The NSC deposited in below‐ground storage organs may represent in some plants more than 30% of the organ biomass (Patrick *et al*., 2013). Below‐ground storage is especially important in herbaceous plants and some shrubs.

Stored NSC compounds are never used wholly for the regeneration of above‐ground parts (but see Klimeš *et al*., 1993), which suggests that they also represent to some extent the accumulation of excess carbon that cannot be utilised for growth (Körner, 2015; Prescott *et al*., 2020). Active storage, when carbohydrates are stored at the expense of growth, and passive accumulation, when carbohydrates are stored as the growth is limited, can be partly recognised in experimental setting (Carvalho & Dietrich, 1993). The passive accumulation may affect biomass allocation of a plant to such extent that the well documented relative increase of root biomass in nutrient‐poor soils in comparison with above‐ground biomass may not only represent greater investment into acquisitive functions of roots but an effect of carbohydrate accumulation in below‐ground organs due to nutrient‐limited growth (Kobe *et al*., 2010).

Some NSCs also play additional roles in plants. Water‐soluble monosaccharides and oligo‐saccharides are important for osmotic protection against drought, salinity and cold (Bartels & Sunkar, 2005; Van den Ende, 2013; ElSayed *et al*., 2014) and some of them are important phloem transport sugars (Liu *et al*., 2012; Jensen *et al*., 2016). Fructans are oligosaccharides and polysaccharides comprising fructose molecules with osmotic functions. Raffinose family oligosaccharides are carbohydrates derived from trisaccharide raffinose. These small oligosaccharides may be transported in phloem, but also participate in long‐term storage, similarly to starch and larger fructans. Smaller carbohydrates, oligosaccharides and polysaccharides can be interconverted, depending on the balance between carbohydrate production and consumption leading to storage or to other functions, which are important for plant functioning (Dietze *et al*., 2014).

The chemical composition of carbohydrates is usually determined by taxonomic group, while the carbohydrate concentration is species specific (Janeček *et al*., 2011). Among individuals of the same species, below‐ground NSC storage appears to scale with above‐ground biomass, and therefore reflect plant size; this, however, is not true across species (Klimešová *et al*., 2018), where differences in NSC storage are not a function of plant size, but affected by plant anatomy and physiology (Plavcová *et al*., 2016).

The amount and proportion of NSC to structural carbohydrates could influence the initial decomposition of the roots as it progresses into senescence. Roots with a higher amount of NSC tend to decompose faster, as they provide more labile substrates for microbial metabolism (Hartmann & Trumbore, 2016).

#### a. Sampling recommendations

See section **XIV. 1. Root nitrogen concentration**. Carbohydrates can be found in all plant organs, but relatively long‐term storage of carbohydrates (at least one season long) is expected in specialised storage organs that are bulky and long‐lived. Therefore, NSC measurements to estimate the total below‐ground nonstructural carbohydrate storage typically target only the specialised storage organs.

#### b. Storage and processing

See section **XIV. 1. Root nitrogen concentration**. Regarding specialised storage organs it is important to consider that different regions of the storage organs may differ in carbohydrate concentration (as exampled in Portes & Carvalho, 2006; Aubrey & Teskey, 2018). Therefore, we recommend to cut a disc crossing the whole storage organ or sample deep into the organ by using a corer.

Samples for carbohydrate assessment are preferably fixed to prevent metabolic processes that would change carbohydrate composition and concentration. This can be done immediately or after a short‐term storage (up to 5 h at room temperature). Fixation may be done by freezing in a −80°C deep freezer, liquid nitrogen or in solid CO_2_. In remote places or when liquid nitrogen is unavailable, samples can be fixed in ethanol (e.g. Chlumská *et al*., 2014). It is also possible to extract carbohydrates directly from fresh material, however, an aliquot should be taken for conversion of concentration to a dry mass basis.

Frozen samples may be stored in deep freezer (−80°C), then lyophilised, weighed and kept in dry conditions until further analysis (for drawbacks of this method see Nelson & Smith, 1972). If samples were fixed in ethanol, the liquid phase must be preserved and added to the liquid phases of the next extraction steps.

#### c. Measurement procedure

##### Extraction steps

The choice of procedures for carbohydrate assessment depends on knowledge of which carbohydrates are stored in the studied species, because carbohydrates may have different solubility and react differently with substances used for quantification. This information can be obtained from the literature (e.g. Hendry, 1987; Janeček *et al*., 2011), or if it is not available, the identification of which carbohydrate types are stored in a particular plant species should be done. This is highly important because there is no universal method that enables quantification of all carbohydrate types by one method (Landhäusser *et al*., 2018). Moreover, comparisons among different laboratories showed that NSC estimates can only hardly be compared, whereas starch estimates were reasonably consistent (Quentin *et al*., 2015). If carbohydrate composition is not known, all steps described below should be done, whereas if composition is known only relevant steps should be selected.

In total, 50 mg of dried and finely ground root material is extracted three times with 5 ml of 80% ethanol by boiling the samples in glass tubes capped with glass marbles in a 95°C water bath for 10 min each (Chow & Landhäusser, 2004). During this period, samples should be thoroughly shaken three times on vortex. After each extraction, the tubes are centrifuged for 5 min, and the supernatants of the three extractions combined for sugar analysis. After the extraction all the aqueous alcoholic solutions must be pooled, the alcohol removed by evaporation, carbohydrates dissolved in deionised water and stored in a freezer for further steps. This procedure will extract the smaller soluble saccharides. The residues remaining in the tubes can be used for starch extraction, either stored wet at −20°C or freeze dried.

As starch is a water‐insoluble polysaccharide, it needs specific procedures for extraction. One of the most accurate methods is the use of starch‐degrading enzymes, α‐amylase and amyloglucosidase, in water solution. We recommend using the Association of Official Agricultural Chemists (AOAC) Method 996.11, American Association of Cereal Chemists (AACC) Method 76.13 and International Association for Cereal Science and Technology (ICC) Standard method 168 (www.megazyme.com; Chlumská *et al*., 2014). In the first step starch should be hydrolysed by thermostable α‐amylase into maltodextrins. This should be done by incubation of the above‐mentioned residue with thermostable α‐amylase (300 U) in MOPS buffer (pH 7) in boiling bath for 6 min. In the second step maltodextrins should be degraded to d‐glucose by amyloglucosidase. In this step the residue is incubated with 20 U of amyloglucosidase in sodium acetate buffer (pH 4.5) at 50°C for 30 min.

When a sample contains fructans (see Hendry, 1987), the use of distilled water for extraction is necessary, because aqueous alcoholic solutions will extract only small saccharides. We recommend Megazyme AOAC Method 999.03 and AACC Method 32.32.01 (www.megazyme.com; Chlumská *et al*., 2014). For fructan extraction, put 200 mg of dried and finely ground root material into a 200 ml beaker and add 40 ml of 80°C hot distilled water. Place the beaker on a magnetic stirrer with heater and stir for 30 min. Not only fructans, but also disaccharides and monosaccharides and shorter molecules of starch and maltodextrins are extracted. These carbohydrates needs to be removed from the extract. First, these compounds should be hydrolysed using a mixture of sucrase, β‐amylase, pullulanase and maltase and then glucose and fructose should be reduced by sodium borohydrate to the sugar alcohols. Fructans should thereafter be enzymatically hydrolysed to fructose by fructanases in sodium acetate buffer (pH 4.5).

##### Analysis of water‐soluble carbohydrates

Pooled supernatants of water‐soluble carbohydrates extraction can be analysed by high‐pressure liquid chromatography (HPLC). Soluble carbohydrates are separated on a Dionex CarboPac™ PA 20 3 × 150 mm column at 22°C, using a 2 mM NaOH solution as the mobile phase with a flow rate of 250 µl min^−1^ (Churakova *et al*., 2018). The quantification of individual sugars is made by calibration with pure standards (based on *a priori* knowledge of which carbohydrates are present in the species analysed) and samples must be chromatographed in the same analytical conditions as the standards. As the soluble carbohydrates are separated and identified based on standards, the individual sugars can be quantified.

##### Determination of starch amount

The glucose that came after starch hydrolysis can be detected using HPLC as described above, or spectrophotometrically after colour formation using enzymes (e.g. glucose oxidase and peroxidase; Galant *et al*., 2015). d‐Glucose is oxidised by glucose oxidase to d‐gluconate. During this reaction, hydrogen peroxide is formed and can be measured in a colorimetric reaction catalysed by peroxidase in which quinone imine dye arises. The absorbance of the dye is measured spectrophotometrically at 510 nm against the reagent blank. For the accurate calculation of starch, when quantifying the free glucose, it is necessary to multiply the amount obtained by a correction factor (162/180) that considers the mass of the molecule of glucose without a water molecule, as they are linked in starch.

##### Determination of fructan amount

Similarly as for glucose after starch hydrolysis, fructose and glucose formed after fructan hydrolysis can be determined by HPLC or using colorimetric 4‐hydroxybenzoic acid hydrazide (PAHBAH) reducing sugar method (Lever, 1973). Glucose and fructose react with PAHBAH reagent in a boiling bath and create PAHBAH coloured complex. The determination of fructose and glucose content is then determined by measuring absorbance spectrophotometrically at 410 nm against the reagent blank. The same correction factor as for starch (162/180) should be used for calculation of fructan amount.

##### Calculations

Total below‐ground NSC storage is determined by calculating separately the weight of each of the water‐soluble carbohydrates and starch. The volume of supernatant is multiplied by the concentration of the carbohydrate considered, divided per mass of analysed subsample and multiplied by the total weight of the organ(s) considered as potential storage organ(s). Total below‐ground NSC content is calculated by expressing total below‐ground NSC storage per unit total plant mass.

#### d. Future research directions

Carbohydrate storage is usually studied for the comparison of species subjected to experimental manipulations, and numerous questions remain as to: whether carbohydrates are reabsorbed before the senescence of storage organs; what proportion of carbohydrate storage is due to active storage; how carbohydrate concentration is constrained by storage organ anatomy; how carbohydrate storage affects root exudates; and how carbohydrate storage co‐varies with root nutrient‐acquisition traits? The application of isotopic tools can help to get a more quantitative understanding of NSC dynamics in plants (Endrulat *et al*., 2016). For ^13^C isotopic measurements of starch, the samples are filtered with centrifugal filter devices to take out the enzymes and then dried in tin capsules in a vacuum centrifuge (Endrulat *et al*., 2016). Using these new tools in combination with environmental manipulation is promising to make progress on longstanding and fundamental questions about the role of NSC storage (e.g. Hartmann & Trumbore, 2016).

### 7. Root phenolic concentration

*Root phenolic concentration* (formal name: *Root phenolic content per dry mass*) is the mass of phenolic compounds per root dry mass (typical units: mg g^−1^).

Phenolics are compounds that have a hydroxyl group directly attached to an aromatic ring; in plants, most of them are synthesised through the phenylpropanoid pathway. Plant phenolics comprise a vast array of compounds (>5000), which range from monophenolics (compounds with one phenolic ring) to polyphenolic compounds (compounds with more than one phenolic ring). Polyphenolic compounds can be further broadly classified into simpler compounds such as flavonoids, and polymeric compounds such as lignins and tannins (Suseela & Tharayil, 2018). After cellulose, phenolics are the most abundant class of compounds in plants, and depending on species and age and identity of the tissue, phenolic compounds can vary considerably (Kulbat, 2016).

Phenolic compounds play important roles in plant resistance to abiotic and biotic stresses, for example by providing structural integrity to cell walls, protecting against pests and pathogens, and facilitating plant–organism interactions (Dai & Mumper, 2010; Tharayil *et al*., 2011). They also affect seed germination and growth of plants, contribute to plant defence, and protect against excessive sun exposure, injuries and metal stress. Phenolics in plant continue to influence the ecosystem functions even after the senescence. For example, tannins are a form of polyphenolics that precipitate proteins and other organic compounds, which may slow down soil mineralisation and enhance soil C storage (Top *et al*., 2017).

#### a. Sampling, storage and processing recommendations

See section **XIV. 1. Root nitrogen concentration**.

#### b. Measurement procedure

Depending on cellular compartmentalisation, the same phenolic compounds could perform different biological functions; therefore it is critical to differentiate the phenolic classes according to their localisation and integration within the plants. To maintain the identity of spatial localisation and extent of integration, the phenolics of the roots are extracted sequentially. The sequential extraction separates the phenolic compounds into three fractions: ‘free phenols’, ‘bound phenols’, and ‘lignin phenols’ (Tamura & Tharayil, 2014; Wang *et al*., 2015).

*Free phenols through solvent extraction.* This procedure extracts the phenolics that are sequestered in vacuoles. In total, 50 mg of a ball‐milled sample is extracted by shaking for 3 h with 5 ml of 80% acetone in glass tubes on a rotary shaker at room temperature (20°C). For dried roots, it is recommended to sonicate the samples for 15 min in the extraction solvent. For senesced roots, and for higher‐order roots, sonication and re‐extraction is required to completely extract the free phenols, because of their woody nature. After centrifugation (2000 ***g*** for 15 min), 1.5 ml of supernatant is transferred to amber vials and stored at −20°C until analysis with chromatography‐mass spectrometry for quantifying free phenols (see below). The remaining supernatant is discarded, and the tissue sediment is washed with 2.5 ml of methanol and centrifuged twice. The methanol is discarded and the tissue sediment (solvent‐extracted fibre) is dried at 50°C overnight and is further used to estimate the bound phenols.

*Bound phenols through mild base hydrolysis.* This procedure extracts the phenolics that are ester‐bound to the cell wall. The above solvent‐extracted fibre is hydrolysed with 6 ml of freshly prepared 1 M NaOH (sparge with argon (Ar) for 30 min) in an 8‐ml glass tube. Potential oxidation of compounds is further minimised by maintaining an Ar atmosphere in headspace during hydrolysis. The bound phenols are extracted from residual fibre by shaking at 20°C for 24 h, after which the tubes are centrifuged and the supernatant is transferred to another tube and acidified with 50% (v/v) HCl. The phenolic compounds in this acidified supernatant are partitioned against 2 ml of ethyl acetate by gentle shaking in tubes, which then are cooled on ice, centrifuged, and the top layer of the ethyl acetate is transferred to vials and stored at −20°C until analysis with chromatography‐mass spectrometry for quantifying ester‐bound phenols (see below). The residual pellet obtained after base hydrolysis is washed twice with 5 ml of deionised water, centrifuged, dried (50℃) and stored for lignin analysis using copper oxide oxidation.

*Gas chromatography‐mass spectrometry (GC‐MS) analysis of phenolic compounds*. The free phenolics and bound phenolic fractions obtained through the previous steps are derivatised using *N*‐methyl‐*N*‐methyl‐*N*‐(trimethylsilyl)‐trifluoroacetamide (MSTFA) with 1% (v/v) trimethylchlorosilane (TMCS) before analysis using a gas chromatograph coupled to a mass spectrometer (GC‐MS). For the derivatisation, 200 µl of the respective sample extract is transferred to 300‐µl low‐volume vials and dried under an N_2_ stream. Then, 100 µl of derivatisation reagent is added, vortexed and incubated at 60°C for 25 min. Samples are analysed on GC‐MS within 24 h of derivatisation. Here, 1 μl of each sample is injected using a 10 : 1 split into a gas chromatograph coupled to a single quadrupole mass spectrometer. Helium is used as a carrier gas with a constant flow rate of 1.0 ml min^−1^. The temperature of the injection port is maintained at 270°C and the source and quad temperatures are set at 150°C and 240°C, respectively. The separation of phenolic compounds is attained using a DB5‐MS column (30 m × 250 µm × 0.25 µm). The initial oven temperature is maintained at 80°C for 2 min, ramped at 10°C min^−1^ to 300°C and held for 5 min. Both scan (range m/z 100–400) and SIM mode are used for identification and quantification. The monophenols are quantified using an external calibration curve obtained using authentic standards. The blank and standard recovery tests are conducted for quality assurance and control (QA/QC). The limit of detection for each of the phenols ranges from 0.01 to 0.1 mg l^−1^, the recovery ranges from 91% to 117%, and the relative SDs for blanks and standards are both within ± 5%.

*Analysis of condensed tannins.* The total content of condensed tannins in the acetone extracts (used for free phenol; extractable tannins) and in the solvent‐extracted fibre (used for bound phenol; nonextractable tannins) is quantified using the acid–butanol assay (Top *et al*., 2017). For the extractable tannins, subsamples (100 µl) are dried under N_2_ gas at 40°C before adding 6 ml of the butanol : HCl (95 : 5) containing iron reagent. For the analysis of the nonextractable tannins, *c*. 20 mg of the solvent‐extracted fibre is weighed into glass tubes and combined with 6 ml of the butanol : HCl reagent. Samples are then placed in a water bath at 90–95°C for 1 h and then cooled on ice. The amount of depolymerised anthocyanidin in the samples is quantified spectrophotometrically by measuring the absorbance at 550 nm. Purified condensed tannins from the same species or Quebracho tannins are used as standards for this analysis. The absorbance of the sample is compared with the standard curve and the concentration of tannins is expressed as the equivalent of the tannins used as the standard.

*Analysis of hydrolysable tannins.* The total content of hydrolysable tannins (HT; sum of gallotannins and ellagitannins) in the acetone extracts (used for free phenol) and in the solvent‐extracted fibre (used for bound phenol) is quantified after methanolysis under acidic conditions (Top *et al*., 2017). Methanolysis is carried out with 100 µl of acetone extract or 20 mg of solvent‐extracted fibre in 2.2 ml of 1.8 M methanoic HCl at 85°C (Hartzfeld *et al*., 2002). The amounts of methyl gallate and ellagic acid formed are then quantified using HPLC coupled to a UV–visible diode array detector. Separations is performed on a C_18_ column using an optimised mobile phase composition that consists of gradient elution of acetonitrile : water containing 0.1% (v/v) formic acid as solvent modifier. The limit of quantification is defined as having a signal‐to‐noise ratio of 10, and all values are reported based on the peak area at 272 nm. The concentration of HT is calculated by comparing sample peak area with that of an external calibration curve generated using authentic standards of methyl gallate for gallotannins and ellagic acid for ellagitannins.

### 8. Root lignin concentration

*Root lignin concentration* (formal name: *Root lignin content per dry mass*) is the mass of lignin per root dry mass (typical units: mg g^−1^).

Lignin is an important organic constituent of vascular plants that is predominantly formed from three basic building blocks: guaiacyl, *p*‐hydroxyphenyl and syringyl monolignols. These building blocks are combined into a polymer with high molecular mass and complex structure, which is characterised by many cross‐linkages. Lignin has a high presence of C atoms, which constitutes *c*. 64% of its mass, and is relatively reduced, which implies that it is costly to produce (Poorter & Villar, 1997). It is hydrophobic, and present in the xylem of many species, facilitating water transport (Boerjan *et al*., 2003). Lignin links with cellulose, hemicellulose and pectin, providing mechanical strength, especially in woody species. Moreover, lignin impedes digestion by herbivores, protects plants against pathogens and reduces lodging as well as the speed of decomposition (Faust *et al*., 2018). It also plays a role in the stress response of plants to low temperature, drought, and salinity (Q. Liu *et al*., 2018). Lignin, therefore, plays a variety of important roles in plants.

Lignin exists in many forms, and different phylogenetic groups of species have different proportions of the three monolignols (Boerjan *et al*., 2003). Moreover, composition may differ among organs and change over time (Abiven *et al*., 2011). This diversity of lignin forms may represent a source of variability for estimations of lignin concentration (Dence, 1992), and can affect plant functioning and litter decomposition (Talbot *et al*., 2012).

A variety of methods is available to estimate the lignin concentration in plants. Traditionally, proximate analyses have been used, in which plant material is treated first with different solvents to remove apolar compounds such as lipids and chlorophyll and water‐soluble compounds such as sugars and minerals. Subsequently, milder and stronger acids are used to hydrolyse starch, cellulose and hemicellulose. The residue that is left after this sequential extraction is determined gravimetrically (Ritter *et al*., 1932; Brauns, 1952) and called Klason lignin, which is a proximate estimate of lignin. A rather similar method has been developed to relate to the digestibility of plants for herbivores (ADL, Acid Detergent Lignin; Van Soest, 1967). This differs from the Klason lignin procedure in the sequence in which acid concentration and temperature are utilised to hydrolyse the polysaccharides (Hatfield *et al*., 1994). A third alternative makes use of the large difference in C concentration between lignin and the (hemi‐)celluloses, and estimates the lignin concentration by determining the C and N concentration of the residue left after mild hydrolysis (CHN method; Poorter & Villar, 1997). Despite being relatively simple and reproducible, these methods generally lead to overestimation of the true lignin fraction in plant samples (Preston & Trofymow, 2015). The residue referred to as Klason lignin, ADL‐lignin and CHN‐lignin may contain proteins precipitated during earlier extraction steps (this is corrected for in the CHN‐lignin method) and other polymeric compounds such as waxes, suberin or condensed tannins, all of which resist acid hydrolysis, as well as nonsoluble compounds such as silicates (Faust *et al*., 2018). In all of these gravimetric approaches, it is good to estimate and correct for the mineral fraction (including silicates) in the residue by determining the ash content, especially for roots as contamination by soil minerals is likely.

Alternative methods aim to break down lignin into monomers, whose concentrations are subsequently determined. The acetyl bromide soluble lignin (ABSL) method uses acetyl bromide to break the bonds and determine the absorption of the phenolic ring of the monomers at 280 nm in solution (Morrison, 1972; Barnes & Anderson, 2017). The copper oxide oxidation (CuO) procedure can also be used to break these bonds and determine the relative proportion of the various monomers by mass spectrometry (Harman‐Ware *et al*., 2016) or NMR (Cheng *et al*., 2013). While these methods suffer far less from possible contaminations in the residue and provide the molecular identity of lignin, they have the drawback that the digestion method, depending on the species, might not break all cross‐linkages, and therefore the total yield of lignins might be underestimated.

Several other approaches can also be used to determine the content and composition of lignins, including pyrolysis followed by GC‐MS (Dignac *et al*., 2009), thiolysis, or near‐infrared spectrometry (Elle *et al*., 2019). Near‐infrared spectrometry is a particularly efficient method for large numbers of samples, once a precise calibration curve has been established with other methods.

Here, we present two proximate methods (ADL, CHN), as well as a method to determine more directly the monolignols (CuO). Since these methods yield contrasting results (Faust *et al*., 2018), we advise to explicitly refer to either of ADL‐lignin, CHN‐lignin and CuO lignin.

#### a. Sampling, storage and processing recommendations

See section **XIV. 1. Root nitrogen concentration**.

#### b. Measurement procedure

##### ADL method

The ADL method is common in ecology. A range of protocols can be found, mostly in conjunction with the determination of ‘neutral detergent fibre’ (e.g. Sluiter *et al*., 2012). Here we follow a simplified protocol, as published by Chaves *et al*. (2002), which focuses on lignin. More details can be found in their source publication.

Dry glass tubes of *c*. 20 ml are weighed and *c*. 250 mg of a milled sample of roots is added. Then, 10 ml of a solution of 2% cetyl trimethylammonium bromide in 1 liter 0.5 M H_2_SO_4_ is added, and the tubes vortexed and refluxed for 1 h at 100°C. The tubes are vortexed regularly during the reflux. Subsequently, the residue is centrifuged for 10 min at 2500 ***g*** and the supernatant is removed. The residue is then washed four times with hot water (15 ml) and twice with acetone (15 ml). The residual acetone is evaporated by keeping the glass tubes overnight at 60°C in a water bath. The tubes are then dried at 90°C and weighed. Working in a fume hood, 1.5 ml of 12 M H_2_SO_4_ is added, and the solution is left to digest the residue for one h at 30°C in a water bath, vortexing every 10 min. The residue is then filtered with glass microfibre filters of known weight, and washed first with hot water (15 ml) and then acetone (15 ml). The residue plus filter are dried and weighed together, then ashed at 450°C and weighed again (filter plus ash). The acid detergent lignin is the difference between weight plus filter before and after ashing, with a correction for the weight loss of the filters themselves, as determined separately.

##### CHN method

This method avoids the treatment with strong acids and can be integrated into a full proximate analysis of the chemical composition of a plant (Poorter & Villar, 1997). It makes use of two extraction steps, one for apolar compounds such as lipids, and one for starch, followed by CHN analysis of the residue.

Dry glass tubes of 10 ml that can be closed with a screw cap are weighed and 250 mg of a milled and dried sample of roots is added (or less so if still a reasonable assessment of the ash in the final residue can be made at the end of the analysis). Then, add 0.8 ml of demineralised water and 2 ml of methanol, close the tube with a screw cap, vortex and wait 5 min. Add 1 ml of chloroform, close the tube again, vortex, and wait 5 min. After centrifugation for 10 min at 2500 ***g***, the supernatant can be carefully removed with a Pasteur pipette. All this can be done at room temperature. Redo the extraction with the remaining pellet. If desired, the total supernatant could subsequently be used to determine lipids, soluble sugars and soluble phenolics following Bligh and Dyer (1959). In the next extraction step, 6 ml 3% (v/v) HCl is added to the pellet. After vortexing, the tubes are placed in a water bath for one h at 100°C. Then let the tubes cool down, and centrifuge them for 10 min at 2500 ***g***. The supernatant can be removed with a Pasteur pipette. Redo this HCl extraction with the remaining pellet. In this way starch, the remainder of the fructans as well as pectin and probably some hemicellulose will be broken down. The supernatant could be analysed for insoluble sugars. The final residue is dried overnight at 40°C, then two nights at 70°C and weighed.

The final residue, with a weight denoted as R_F_, consists of minerals, protein, (hemi)cellulose, lignin and probably some other high‐C‐containing secondary organic compounds such as cutin and tannins. A small sample of this residue (*c*. 3 mg) is taken for analysis of CHN with an elemental analyser (see section **XIV. 1. Root nitrogen concentration**). The remainder of the residue is weighed and ashed at 550°C in a muffle furnace, followed by a determination of ash weight. Assuming that all weights are in mg and concentrations are expressed in g g^−1^, the concentration of lignin in the residue can now be calculated as follows:

• First, assuming that the weight of the residue R_F_ is the sum of lignin (Lig), some of the total structural carbohydrates in the form of cellulose and hemicellulose that remained after the extraction (TSC), some cell wall and precipitated protein (Pro) and some ash (Ash). In formulae:

(Eqn 1) $$R_{F}=\mathrm{Lig}+\mathrm{TSC}+\mathrm{Pro}+\mathrm{Ash}$$

The ash‐free weight of the residue is then calculated as:

(Eqn 2) $$R_{F}^{1}=R_{F}‐\mathrm{Ash}=\;\mathrm{Lig}+\mathrm{TSC}+\mathrm{Pro}$$

• Second, starting with the N concentration [N]_F_ as determined by CHN analysis, the protein content in the ash‐free residue can be calculated, assuming N represents 16% of the protein, as in RuBisCo:

(Eqn 3) $$\mathrm{Pro}={\left[N\right]}_{F}\times \frac{1}{1‐\frac{\mathrm{Ash}}{R_{F}}\;}\times 6.25\times R_{F}^{1}$$

Then, ash and protein‐free weight of the residue becomes:

(Eqn 4) $$R_{F}^{2}=R_{F}^{1}‐\mathrm{Pro}=\mathrm{Lig}+\mathrm{TSC}$$

• Third, assuming an average C concentration in protein as in RuBisCo, (540 mg g^−1^), the C concentration of the ash‐free and protein‐free part of the residue is then calculated as:

(Eqn 5) $${\left[C\right]}_{F}^{2}=\frac{{\left[C\right]}_{F}\;\times \frac{1}{1‐\frac{\mathrm{Ash}}{R_{F}}}\times R_{F}^{1}‐\text{Pro}\times 0.54}{R_{F}^{2}}$$

where [C]_F_ is the C concentration in the residue R_F_, as measured by CHN analysis.

Assuming that the ash‐free and protein‐free residue $R_{F}^{2}$ only contains structural carbohydrates and lignin, and further assuming the C concentration of these compounds to be 440 and 640 mg g^−1^, respectively, the lignin content can then be calculated as:

(Eqn 6) $$\text{Lig}=\frac{{\left[C\right]}_{F\;}^{2}\;‐\;440}{\;640\;‐\;440\;}\times R_{F}^{2}$$

If S was the amount of sample originally weighed and processed in that tube, the lignin concentration in the original sample and expressed in mg g^−1^ becomes:

(Eqn 7) $$\left[\text{Lig}\right]=\frac{\text{Lig}}{S}\times 1000$$

##### CuO procedure

The heteropolymeric lignin is depolymerised under high temperature and pressure in the presence of CuO as catalyst to its monomeric phenols (SVC: S = syringyl lignol, V = vanillyl lignol, C = cinnamyl lignol), which are then quantitatively determined using GC‐MS (Wang *et al*., 2015). Although the CuO oxidation can be directly done on nonextracted plant material, it is advisable to sequentially extract it with solvent (extractable phenolics) and mild base (ester/ether bound phenolics) before proceeding to CuO oxidation as this scheme would provide a more accurate reading of SVC lignin. Briefly, the lignin fraction is oxidatively depolymerised in 23 ml acid digestion vessels (Model 4745, Parr Instruments). The base hydrolysed pellet (50–100 mg) is combined with 500 mg CuO, 75 mg Fe(NH_4_)_2_(SO_4_)_2_·6H_2_O, and 5 ml freshly prepared 2 M NaOH (presparged by Ar for 30 min to remove dissolved gases) in a Teflon cup (Tamura & Tharayil, 2014). The tube is rinsed with 5 ml of 2 M NaOH twice and transferred to the Teflon cup, which is then sparged again with Ar for *c*. 3 min and immediately capped. The vessel is carefully sealed and incubated at 155°C for 160 min. After CuO oxidation, the vessel is cooled in an ice bath for 20–30 min to room temperature. All digested liquid is then transferred to 50 ml centrifuge tubes and 50 µl of 200 mg l^−1^ *trans*‐cinnamic acid and ethyl vanillin is added as internal standards. About 1.5 ml 24 M H_2_SO_4_ is then added to adjust the pH to <2, and the tube is gently shaken (*c*. 5–10 min) until any coagulation is observed. The tubes are centrifuged, and 12 ml supernatant is transferred to glass tubes wrapped with aluminium foil. The depolymerised lignin‐derived phenols are then liquid‐liquid extracted by 2 ml ethyl acetate at low temperature and 1 ml ethyl acetate layer is stored in GC vials at 4°C. During the whole sequential extraction, parallel blanks are also extracted, following the same procedures for QA/QC. The monophenols are then derivatised using MSTFA, and analysed using GC‐MS as described under section **XIV. 7. Root phenolic concentration**. The sum of monophenols (S, V, C) reflects the total lignin gained by the CuO method and is referred to as CuO lignin.

## 15 Root mechanics

Root mechanical properties are the physical properties that the root material exhibits upon the application of forces such as swaying, uprooting and substrate movement. Roots must anchor a plant in the ground (Fitter, 1987; Freschet & Roumet, 2017) and whereas herbaceous species need to avoid vertical uprooting by animal grazers, tall trees must resist lateral loading during windstorms (Coutts, 1986). Flooding and substrate mass movement (e.g. landslides, rockfall, avalanches), can also cause lateral uprooting failure for all types of vegetation (Stokes *et al*., 2009). Resistance to uprooting of a plant or tree depends on the shape of the root system and its material properties. Strength is defined as the maximum stress that a material (or, for example, a root), can withstand while being deformed (through tension, compression or bending), divided by some measure of the dimension of the root or root system. The strength of individual roots can be tensile, compressive or bending but the most commonly studied mechanical trait of individual roots is tensile strength.

Over the last 30 yr, many experimental studies have been performed, mostly using a winch and load cell, to measure the force required to overturn adult trees living in regions exposed to wind storms (e.g. Cucchi *et al*., 2004; Nicoll *et al*., 2006). As a tree sways and overturns during a windstorm, roots fail initially in shear (i.e. deformation of a material in which parallel internal surfaces slide past one another) and compression around the root system. During overturning, lateral roots on the windward side of the tree are held in tension, and usually provide 25–50% of the resistance to overturning, depending on species and RSA (Coutts, 1986; Yang *et al*., 2017). Wood is up to three times stronger in tension than in compression (Kretschmann, 2010), therefore, the tensile strength of roots is a major component of tree anchorage. In lateral uprooting tests that better mimic the action of substrate movement on herbaceous and shrubby plants, mechanical failure occurs earlier in those plants that have a smaller root volume (Stokes *et al*., 2007), less lateral root spread (Mickovski *et al*., 2005) and with less root per unit volume of soil (Burylo *et al*., 2009). Although vertical uprooting studies on herbaceous species performed in field conditions are extremely scarce, data have shown that the tensile force required to uproot whole plants is positively correlated to root system depth and width, extent of branching and number of roots (O’Toole & Soemartono, 1981; Devkota *et al*., 2006). While field experiments are highly useful for understanding the mechanics of plant anchorage, results can be complex to analyse, especially as soil type and moisture content affects significantly the anchorage of a plant. Also, some part of the root system always remains embedded in the soil, therefore it is difficult to determine the strength due to the roots that fail and are pulled out of the soil, and the strength due to the remaining part of the root system hidden in the soil (Giadrossich *et al*., 2017). Therefore, it is not appropriate to consider uprooting and overturning of whole root systems as mechanical traits. Instead, we will focus on the measurement of mechanical properties of individual roots, that contribute to the overall resistance to vertical and lateral uprooting of a plant. An estimation of total anchorage strength can be obtained by summing the basal tensile strengths of all the roots.

Disentangling the influence of individual root traits on anchorage is challenging, but numerical models have been developed that enable us to understand the role of single roots in resisting plant uprooting (Fourcaud *et al*., 2007; Wang *et al*., 2018). At an experimental level, most of the studies that have focused on the mechanics of single roots can be found in the geotechnical engineering literature. During a shallow landslide on a vegetated slope, roots held in tension usually break after the soil has failed, and so can actually prevent or retard slope failure (Stokes *et al*., 2009). The number, cross‐sectional area, orientation and to a lesser extent, the material properties of roots crossing the potential failure plane on a slope, largely determine the contribution of vegetation to stability of the slope (Mao *et al*., 2012). Root area ratio (RAR; the fraction of a plane of soil occupied by roots), tensile strength and the resistance of individual roots to being pulled out of soil (pull‐out resistance), are important input parameters in geotechnical engineering models that calculate the contribution of vegetation to slope stability (Stokes *et al*., 2009). When individual roots are pulled out of soil, as for the anchorage of whole root systems, pull‐out resistance depends largely on soil physical properties as well as the unknown part of the root left in the soil after the test, and so cannot be considered a trait (Giadrossich *et al*., 2017). Only tests on individual roots, that are not contained within a substrate, can be used for quantifying mechanical traits.

Tensile strength depends on both the geometry of a root and its material properties. The thicker a root is, the more force will be required to cause failure. If a root has high elastic material properties, it will be able to resist loading further without failing, therefore improving both plant anchorage and slope stability.

### 1. Root tensile strength

*Root tensile strength* is the force required per cross‐sectional area of the root to cause failure of the root, either through breakage or nonreversible deformation (typical units: megapascals (MPa), or Newtons (N) mm^−2^) (frequent abbreviation: *T_r_*).

To measure the strength of individual roots, it is necessary to perform mechanical testing on whole roots or root samples in the laboratory. Generally, roots that are thinner than 10.0 mm, are tested in tension whereas those that are > 10.0 mm in diameter are tested in bending or compression (Giadrossich *et al*., 2017). These tests are not limited by technical constraints in the laboratory, but reflect the way that roots behave mechanically during uprooting. Herbaceous species with thin roots are held in tension as a grazing animal pulls upwards. Tall trees however, that sway and are pushed sideways during a windstorm, have thicker roots held in tension, bending and compression around the tree. As soil shears underneath the soil–root plate, thinner roots are usually broken in tension or slip out of the soil. Nevertheless, crop species, such as sunflower (*Helianthus annuus*) and maize (*Zea mays*) can fail through lodging, that is, the lateral displacement of stems or roots near the soil surface. During lodging, roots that are < 10.0 mm can be subjected to failure mechanisms similar to those found in trees (Goodman & Ennos, 1999). Similarly, when substrate mass movement occurs, lateral forces displace plants sideways and roots on the upper side of the plant are held in tension whereas those downslope usually bend and buckle. Roots with a high tensile strength will break after the soil has failed, and up to the point of failure, these roots are capable of anchoring the plant to the ground and retard or prevent mass slope failure. Thicker roots can act as soil nails, held in compression or bending, and pinning the root systems into the substrate (Stokes *et al*., 2009).

Material properties of roots are significantly affected by their water content, with drier roots being significantly stronger. After 5 h of air drying fully hydrated roots that were < 2 mm in diameter, Boldrin *et al*. (2018) demonstrated that tensile strength increased by up to 162%. Therefore, for roots growing in drier soils (e.g. on the upper parts of a slope), tensile strength is greater than in wetter areas such as hollows (Hales *et al*., 2009), and so in drier soils, plants are better anchored and substrate is more mechanically reinforced. As soil moisture changes throughout the year, tensile strength will also fluctuate (Ghestem *et al*., 2014; Hales & Miniat, 2017). Generally, roots in compacted soils have a higher tensile strength than those growing in looser soils (Ghestem *et al*., 2014), but few comparative studies exist detailing the direct effects of the local environment on root mechanical properties.

Until recently, it was thought that tensile strength of a root was almost entirely dependent on the proportion of cellulose and lignin present: thinner (supposedly younger) roots are relatively stronger than thicker (supposedly older) roots because of the higher amounts of cellulose (Genet *et al*., 2005) and lignin (Zhang *et al*., 2014) present. The structure of cellulose renders it particularly efficient for resisting failure in tension (Sjöström, 1993). However, in these studies of tensile strength, roots were usually grouped into diameter classes, with no consideration of developmental age. It has since been shown that the strength of individual, primary roots of oats (*Avena fatua* L.) and barley (*Hordeum vulgare* L.) increased by more than an order of magnitude with increasing tissue age along an axis (Dumlao *et al*., 2015; Loades *et al*., 2015).

The tensile strength of a single root or root section will also depend strongly on its cortex‐to‐stele area ratio (Chimungu *et al*., 2015; Dumlao *et al*., 2015; Mao *et al*., 2018). As root cross‐sectional area is a function of root diameter squared, cortex thickness will have a large effect on the estimation of strength. The cortex is usually much weaker mechanically than the central lignified stele due to its high content of large, thin‐walled parenchyma cells (Mao *et al*., 2018). In very young roots (i.e. the lowest topological orders), tensile strength is low and a higher proportion of cortex is present compared with older, stronger roots that have thinner cortex (i.e. higher topological orders). However, as roots age, tensile strength decreases and Mao *et al*. (2018) suggest that increasingly large roots have a higher probability of material defects occurring in either cellulose structuration or cell wall layer bonding, resulting in the lower strength. As the majority of tests presented in the literature have been made on roots with cortex and bark left intact, and for easier comparison of results, it is standard that mechanical tests be performed on roots with an intact cortex and bark structure (Giadrossich *et al*., 2017). Because the importance of topological order has only recently become clear, few comparative studies exist, detailing how tensile strength varies depending on species and local environment. However, Loades *et al*. (2013) tested the effects of waterlogging and compaction on nodal (originating from the stem), seminal (origination from the grain) and lateral (borne from seminal roots) roots of *H. vulgare*, and demonstrated that mechanical responses of roots to treatment depended on root type. Seminal roots were always stronger than lateral roots for the same treatment and although compaction had no effect on tensile strength, waterlogging resulted in weaker roots, possibly due to changes in root anatomical structure. To our knowledge, the only study comparing the influence of anatomical features on root tensile strength in different species, showed that an increase in the percentage of fibres significantly improved strength in broadleaf tree species (Jiang *et al*., 2013).

Relationships between tensile strength and other root traits are scanty, and have shown only weak positive relationships with RTD and root nitrogen concentration, and a negative relationship with soluble sugar (Ghestem, 2012) and nonstructural carbohydrate concentration (Genet *et al*., 2011). To the authors’ knowledge, no comparative studies exist that investigate the relationship in tensile strength between above‐ground and below‐ground organs.

#### a. Sampling and experimental recommendations

See section **VI. Experimentation and sampling in laboratory and field**. The length of root tested can influence the estimation of tensile strength for two main reasons: (1) long roots are more likely to have flaws along their length, for example, due to wounds, the misalignment of cells or the presence of a branching point, and may break at a smaller force than a shorter root of the same diameter; (2) Choosing only straight, potentially nonflawed sections of roots to test can bias measurements, and do not represent the wide variability observed in nature (Giadrossich *et al*., 2017). Roots are often tortuous, branched and have many weak points. Therefore, by testing a long section of root, data will provide information about the overall strength of the whole root, whereas tests on near perfect specimens will yield more precise data about the behaviour of the root material itself. Specimen length should ideally be between 20 and 30 times the central diameter of the sample (Ghestem *et al*., 2014; Giadrossich *et al*., 2017).

#### b. Storage and processing

Mechanical tests on individual fine roots are best carried out on fresh samples (i.e. as soon as possible or at least within 1 wk of sampling, Bischetti *et al*., 2005). Root moisture content is a dominant factor affecting mechanical traits (Boldrin *et al*., 2018), as dried roots (with 50% less moisture content), can be more than twice as strong as fresh roots when tested in tension (Hales *et al*., 2013). Several methods have been used to conserve roots for several weeks or months (Giadrossich *et al*., 2017), but a preferred method would be to keep root samples in a 15% alcohol solution (Bischetti *et al*., 2005) to prevent microbial degradation and/or keep refrigerated at 4°C. It is not recommended to freeze roots as this may alter mechanical properties at the cellular or tissue level.

#### c. Measurement procedure

When measuring mechanical traits of individual roots that are < 10 mm in diameter in the laboratory, force (*F*, measured in Newtons, N) is applied to the sample, using a Universal Testing Machine. The force applied is tensile, that is the longitudinal pulling apart (breakage) of an entire root or root section clamped at its extremities (Giadrossich *et al*., 2017). As force is applied to a root, the neighbouring particles of root material exert internal forces on each other. Stress (σ) is the physical quantity that expresses these internal forces and is quantified as: the force per unit area induced in the material in response to the externally applied force. Root tensile strength (*T_r_*) is calculated as force divided by the original cross‐sectional area at the point where it breaks:

(Eqn 8) $$T_{r}=\frac{F}{\left(\frac{\pi }{4}\right)d^{2}}$$

where *F* is the maximum force when a root is loaded until failure, and *d* is root diameter.

A testing machine has a stationary and a moving part that pulls the root. Force is recorded with a force transducer (or load cell), usually coupled to a displacement sensor. Force transducers create an electrical signal whose magnitude is directly proportional to the force being measured. Different maximum capacities (measured in Newtons, N) are available and are chosen depending on the thickness of the root. Tests should be performed at a speed of 1–10 mm min^−1^ (Giadrossich *et al*., 2017), as faster test speeds lead to an overestimation of tensile stress (Cofie & Koolen, 2001).

When performing a mechanical test, it is important to quantify sample dimensions accurately. The diameter of the specimen is usually measured at the failure point, but if the root is noncircular or tapers quickly, the measurement can be taken as the average between the height and diameter at several points along the root. The diameter is usually measured with a calliper or a binocular microscope equipped with a micrometre scale (e.g. Loades *et al*., 2010; Hales *et al*., 2013; Mao *et al*., 2018), although calliper measurements may induce errors due to pinching of soft root tissue.

**Fig. 19:**
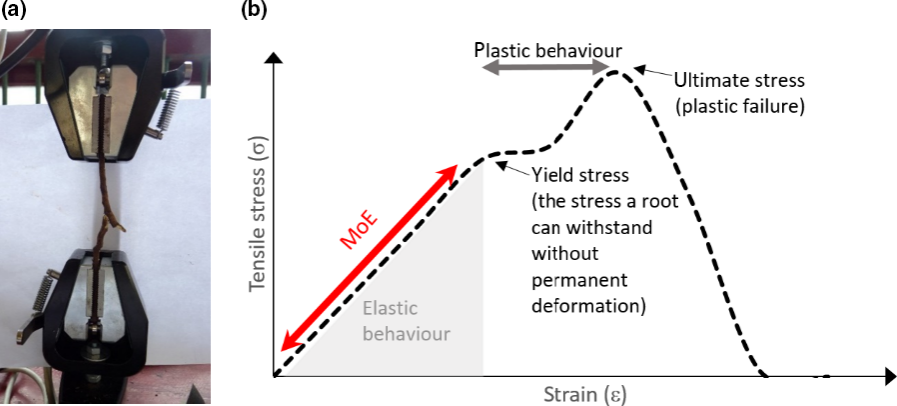
(a) A section of root is clamped between the jaws of a Universal Testing Machine and stretched until failure occurs in tension; (b) during a tensile test, stress (σ) and strain (ε) increase quasilinearly and the root has an elastic behaviour. Modulus of elasticity (MoE) can be calculated from the slope of the linear relationship between stress and strain. Once permanent deformation starts (yield stress), the root undergoes nonlinear plastic behaviour. The point at which ultimate failure occurs is used to calculate tensile strength. Note that yield stress can be difficult or even impossible to observe during a test and usually only a small inflection point occurs in the slope of stress and strain.

Depending on the diameter of the root, different clamping systems can be used to attach the root to the testing machine. The most common method is to clamp the root at each extremity using standard clamps (Fig. 19a). However, roots often slip or break near or within the clamps, although Hales *et al*. (2013) argue that even when failure occurs near the clamps the test should be considered as valid. Over the last 20 yr, several types of clamping systems have been developed. Simply adding a thin layer of cork or sandpaper to the inside of screw clamps significantly increases grip (de Baets *et al*., 2008). However, one of the best methods is to avoid clamping the root directly and so avoid damage to the sample through crushing by clamps, although sample preparation is more time consuming. For example, Operstein and Frydman (2000) successfully attached 30 mm pipes filled with a matrix of stone and epoxy at the ends of roots. Tosi (2007) epoxied the ends of roots to steel forks, which were then clamped to the testing machine and Nilaweera & Nutalaya (1999) strengthened the ends of the samples by epoxy resin moulding. It is recommended that different clamping methods be tested on the root samples and that the final choice be made depending on the difficulty in testing the root sections (e.g. if cortex slips off the stele easily, or if samples are crushed in the clamps) and the time that can be devoted to sample preparation.

During a mechanical test, data obtained are the maximum force at which the sample fails and how far the sample is stretched before failure occurs. Strain (ε), is the relative extended deformation of a sample during a test and is dimensionless (Fig. 19b):

(Eqn 9) $$\varepsilon =\frac{\Delta L}{L}$$

where ΔL is the extension of the sample during the test and *L* is the original length between the clamps. Strain in a root held in tension is composed of three phases: (1) an initial phase of stretching followed by (2) a phase of reversible deformation called the elastic phase (Fig. 19b), which is sometimes, but not always, followed by (3) a phase of nonlinear and irreversible deformation called the plastic phase leading to ultimate failure. Phase (2) terminates when the yield stress is reached (Fig. 19b). The yield stress is the stress a root can withstand without permanent deformation.

#### d. Future research directions

In studies of plant anchorage and soil reinforcement, the mechanical behaviour of the above‐ground and below‐ground parts is usually dissociated, except in studies of tree overturning. It is not known whether the tensile strength of roots is correlated with that of shoots or leaves. Similarly, if correlations between tensile strength and other plant traits such as root or shoot tissue density were strong, the possibility of using an easier‐to‐measure trait as a proxy for tensile strength would be highly useful. Therefore, in future analytical studies, examining how tensile strength co‐varies with other plant traits, such as RTD, would be valuable and could then be used as a proxy in models of slope stability.

How tensile strength is modified by the chemical and physical structure at a cellular level is a major next step in unravelling the mechanisms that determine root mechanical properties. Not only are anatomical studies required that correlate tensile strength with root structural traits (Mao *et al*., 2018), but a micro‐scale analysis of cellulose behaviour during cell wall deformation (Cosgrove, 2016) would explain how root mechanical properties are altered throughout the growth of a root and in particular, with regard to changes in moisture content (Kolb *et al*., 2018).

Uprooting experiments in both field and laboratory conditions are scanty. Due to the technical difficulties linked to scaling effects in laboratory tests, whereby plants are for example grown in pots and uprooted vertically using a Universal Testing Machine, it is advised to perform uprooting tests in the field in nonconfined conditions, unless plant specimens are very small in relation to container size (Ennos, 1989; Giadrossich *et al*., 2017). However, disentangling the effects of soil physical properties, root age and architecture on plant anchorage is challenging. The development of uprooting models that use mechanical data on individual roots help explain the role of each root in the uprooting process (Yang *et al*., 2017), thereby significantly increasing our understanding of the anchorage process. For example, models that take into account progressive breaking of roots during plant or soil failure, as well as root slippage rather than breakage, require information on the mechanical behaviour of single roots (Pollen & Simon, 2005). To improve these models, an understanding of how tensile strength changes along roots, as well as spatially within a root system, depending on age, RSA and local soil properties, is required (Schwarz *et al*., 2013). Improving anchorage models would also help explain how some grass species resist grazing better than others, or how certain herbaceous species can remain anchored on unstable substrate on slopes (Ghestem *et al*., 2014) and riverbanks exposed to periodic flooding (Docker & Hubble, 2008).

Future analytical studies, examining how tensile strength co‐varies with other plant traits, such as RTD, would also be valuable and could then be used as a proxy in models of slope stability or plant uprooting.

### 2. Root modulus of elasticity

*Root modulus of elasticity in the longitudinal direction* is the slope of the quasilinear part of the relationship between root stress (σ) and root strain (ε) (typical units: megapascals (MPa)) (frequent abbreviation: modulus of elasticity (MoE_L_)). As described under section **XV**. **1. Root tensile strength**, root stress is the force per unit root cross‐section area induced in a root in response to the externally applied force and strain is the relative extended deformation of the root per total root length, during the reversible elastic stretching of a root in the longitudinal direction. This trait represents a measure of the elasticity of the root, with higher MoE_L_ meaning the root has a better capacity to withstand tension without undergoing permanent deformation.

During the estimation of root tensile strength (see section **XV. 1. Root tensile strength**), data are obtained simultaneously that characterise a second material property: Young’s modulus (*E*) or elastic modulus or modulus of elasticity (MoE). Young’s modulus refers to the elastic behaviour of an isotropic material, such as metal and so for roots, should be a material property independent of organ size. However, as roots are orthotropic, that is, they have unique and independent mechanical properties in the directions of three mutually perpendicular axes, most authors use the term MoE followed by the direction in which the force was applied during loading, for example MoE_L_ for the elastic modulus measured in the longitudinal direction.

Roots that are highly resistant to being deformed elastically have a high MoE_L_, and can remain anchored in soil, even after soil failure has occurred (Cohen *et al*., 2009), therefore holding vegetation in place and retarding or preventing mass substrate failure. Root MoE_L_ is related strongly and positively to tensile strength, therefore factors that influence tensile strength usually also affect MoE_L_. As MoE_L_ is a material property, whose value is independent of root cross‐sectional area and root length, the utility of this trait lies in comparative studies of root elasticity among roots of for example different ages, species and growing in a range of environments. As both plant anchorage and soil reinforcement on slopes depends largely on the number and dimensions of roots in soil, variations in MoE_L_ have been much less studied than for tensile strength and few data exist detailing how MoE_L_ is modified by either internal or external factors.

As with tensile strength, when roots lose moisture, their MoE_L_ increases significantly (Boldrin *et al*., 2018). Kolb *et al*. (2018) suggested that upon drying, it is probable that the hygroscopic matrix polymers between the cellulose fibrils shrink, leading to an increase of the volume fraction of microfibrils of cellulose in the cell wall, so modifying MoE_L_.

Although MoE_L_ is a material property independent of organ size, as with tensile strength, the overall MoE_L_ of a woody root is significantly affected by the cortex : stele ratio (Mao *et al*., 2018). The younger the root (and therefore the lower the topological order), the higher the proportion of cortex (comprising mostly large, thin‐walled parenchyma cells) and the lower the root MoE_L_ (i.e. with a reduced capacity to withstand tension without undergoing permanent deformation, Mao *et al*., 2018). Because the importance of topological order has only recently become clear, few comparative studies exist, detailing how MoE_L_ varies among species or with the local environment.

As with tensile strength, relationships between MoE_L_ and other root traits are rare, and have shown only weak positive relationships with RTD and root nitrogen concentration and a negative relationship with soluble sugar content (Ghestem, 2012). To the authors’ knowledge, no comparative studies exist that investigate the relationship in MoE_L_ between above‐ground and below‐ground organs.

#### a. Sampling, storage and processing recommendations

See section **XV. 1. Root tensile strength**.

#### b. Measurement procedure

See section **XV. 1. Root tensile strength**. During a tensile test, data obtained include stress (σ) and strain (ε). The MoE_L_ (i.e. resistance to being deformed elastically, and not to failure) is defined as the slope of the quasilinear part (elastic zone) of the relationship when stress and strain are plotted together (Fig. 19b) and calculated as:

(Eqn 10) $${\mathrm{MoE}}_{L}=\left(\frac{\sigma }{\varepsilon }\right)\left(\frac{L}{\left(\frac{\pi }{4}\right)d^{2}}\right)$$

where $\frac{\sigma }{\varepsilon }$ is the linear slope at the beginning of a tensile test, *L* is the initial length of the sample and measured as the distance between the clamps, *d* is root diameter.

#### c. Future research directions

As MoE_L_ is strongly related to tensile strength and is a value obtained during a tensile test, the future research directions are similar to those for root tensile strength. Data for MoE_L_ are rarely presented in studies of root tensile strength, yet these values are important input in models that describe the stepwise failure of bundles of roots during substrate failure (Cohen *et al*., 2011).

As MoE_L_ is standardised per cross‐sectional area of root and root length, data can be used for a fundamental understanding of root material properties, and how they change in certain conditions, or with plant ontogeny. It is not yet fully understood which anatomical and chemical characteristics of root structure govern MoE_L_ (Mao *et al*., 2018), and if correlations with other traits occur. Nor is it known if resource supply and carbon allocation, for example in the form of nonstructural carbohydrates, influence material properties, although Genet *et al*. (2011) show that root wood is weaker when nonstructural carbohydrates are less abundant. In particular, experiments examining how MoE_L_ is affected during for example root drying, combined with a chemical and physical analysis of cell wall ultrastructure, would explain how root mechanical behaviour is affected by soil moisture content. Such data would be especially useful in dynamic models of plant anchorage and slope stability over different seasons and soil types.

## 16 Root dynamics

The term root dynamics refers to the timing of root birth and death (i.e. phenology) and the resulting lifespan of individual roots and turnover of root populations. These processes together are also sometimes referred to as root demography. Patterns of birth, death, and replacement are recognised as important aspects of plant ecology partly mediating the absorptive capacity of the fine‐root system and accounting for between 10% to over 50% of annual net primary productivity in terrestrial plant systems (Aerts *et al*., 1992; Ruess *et al*., 2003; McCormack *et al*., 2015a). However, the requirement for repeated, nondestructive observations to directly assess root dynamics has remained a strong impediment to our broad understanding of first, the typical patterns of variation across species, and second, how abiotic and biotic factors affect root dynamics both across and within species.

This chapter discusses why and how to measure key aspects of fine‐root dynamics in field conditions. These measures are related to root activity and may be used to understand plant growth responses to abiotic and biotic factors; for example, long‐term or short‐term responses to climate change, management, and so on. While root dynamics are inherently temporal in nature, spatial aspects, including rooting depth, should also be considered when interpreting variation in root dynamics. Root dynamics also represent an important part of shifting resource allocation within a plant over the course of the growing season (e.g. from leaves to roots and to stems). As such, aspects of root dynamics, particularly root phenology, are linked with aspects of whole‐plant phenology. For example, the production of roots may be either synchronous or asynchronous with leaves with variation occurring both among growth forms and within individuals but across years (Steinaker & Wilson, 2008; Steinaker *et al*., 2010; Abramoff & Finzi, 2015; McCormack *et al*., 2015b). Shifts in root dynamics in response to abiotic (radiation, temperature, soil moisture, soil strength and porosity, C allocation) and biotic conditions (competition, symbionts, pests), and the amount of temporal offset between the peak in shoot and root production can also be informative of fine‐root to whole‐plant strategies for resource investment and allocation.

### 1. Lifespan

*Root lifespan* is the time between the birth and the death of a root (typical units: d or yr).

It is generally assumed that during this period the root is metabolically active and provides some function for the plant (e.g. resource uptake), although it is expected that the specific metabolic rate and functional activity of the root will vary with root age (Bouma *et al*., 2001b; Volder *et al*., 2009). It is also important to note that age may vary within a root as the construction and development of a single root may occur over the course of days to weeks; similar, root senescence may occur gradually (e.g. root cortex may senesce, leaving a viable stele; Schneider & Lynch, 2018). However, this differentiation is not considered within this section and age is considered at the individual root level from first appearance until all functions have ceased. Previous studies have highlighted significant variation in average fine‐root lifespans both within and among species and important linkages between root traits, local environmental conditions, and fine‐root lifespan are beginning to emerge (Withington *et al*., 2006; McCormack *et al*., 2012; Adams *et al*., 2013; Pilon *et al*., 2013; K. Sun *et al*., 2016; Gu *et al*., 2017; Mueller *et al*., 2018). For example, species that construct thicker, or denser roots tend to have roots that live longer than thinner roots with low tissue density (B. Liu *et al*., 2016; Liese *et al*., 2019). Outside of the plant, additional factors such as climate (Eissenstat *et al*., 2013), and the local availability of soil resources (Adams *et al*., 2013) are also likely to be important factors mediating fine‐root lifespan. However, datasets reporting lifespan across species and environments and studies that include experimental manipulations to directly test environmental effects on fine‐root lifespan are still relatively few in number which limits our ability to make meaningful inferences at broad scales (McCormack *et al*., 2013; McCormack & Guo, 2014). The importance of fine‐root lifespan to plant resource allocation and acquisition coupled with our limited understanding of variation in lifespan among species and across environments make this a particularly important process to target for future study.

In this respect, it is important that fine‐root lifespan not be viewed and studied separately from other aspects of plant ecology, but as an integral suite of plants traits whose expressions and responses are mediated by their environment. This means that we explicitly seek to understand and quantify variation in lifespan in relation to other key plant and environmental factors. Explicit considerations of root and plant traits (McCormack *et al*., 2012; K. Sun *et al*., 2016), age of the plant (Coleman & Aubrey, 2018), plant evolutionary history, the time when roots are constructed (Anderson *et al*., 2003; Gu *et al*., 2017), or the depth at which roots are produced (Coleman *et al*., 2000; Maeght *et al*., 2015; Mueller *et al*., 2018) can help develop a clearer picture of plant strategies for resource acquisition and use.

While fine‐root lifespan is inherently a process that occurs at the level of a root segment or individual root (i.e. the lifespan of a root), we are most often interested in how lifespan manifests across the root population. For example, average lifespan is frequently reported and represents a central tendency, either mean or median, of a root population. While measures of central tendency are useful and provide a tractable means for making comparisons across species or environmental contexts, there is also increasing evidence that rather than a single central tendency (uni‐modal) many fine‐root populations display a bimodal tendency with many roots that are relatively short lived as well as another portion of fine roots that are longer lived (Gu *et al*., 2017). As such, future studies are likely to be able to tease apart important aspects of plant investments in root lifespan by investigating similarities and differences based on central tendencies (i.e. mean, median) as well as by interpreting differences in the multimodal distributions in lifespan within the fine‐root population.

#### a. Measurement procedures

Approaches to study or estimate fine‐root lifespan can be divided into two main groups; direct observations and indirect approaches. Direct observations are techniques in which the researcher directly views or images individual roots in the soil environment. The most common approach today is the minirhizotron technique which uses clear tubes installed into the soil through which the soil environment and roots may be repeatedly imaged overtime using a scanner or camera. Full rhizotrons have also been used to measure root lifespan (Pregitzer *et al*., 1993), but because of their expense and disturbance required for installation they are now much less common. In addition to rhizotrons and minirhizotrons, many researchers have utilised various sizes of root windows and rhizoboxes/root boxes (Bai *et al*., 2008; Xia *et al*., 2010). These may be as simple as a glass window placed on the soil and covered between viewings to block light (K. Sun *et al*., 2016), to small dugouts with glass or acetate framed along the sides with insulation and covering (Fernandez & Caldwell, 1975; Meier *et al*., 2015), as well as also rhizoboxes (of various sizes) refilled with substrate, planted with target species and maintained *ex situ* (Beyer *et al*., 2013) (see full description under section **VI. 3. b. Overview of root observation methods**). While they are more difficult to install, limiting replication, root windows and rhizoboxes do have a distinct advantage in that, unlike minirhizotrons, it is generally possible to manipulate and harvest (specific) roots from within a viewing window and to measure root and soil parameters more thoroughly. This may be advantageous for research questions related to how root processes and stress factors related to root lifespan may change as roots age. Moreover, imaging procedures for rhizotrons of various kinds are becoming increasingly inexpensive (Mohamed *et al*., 2017).

##### Direct root observations with (mini‐)rhizotrons

**Fig. 20:**
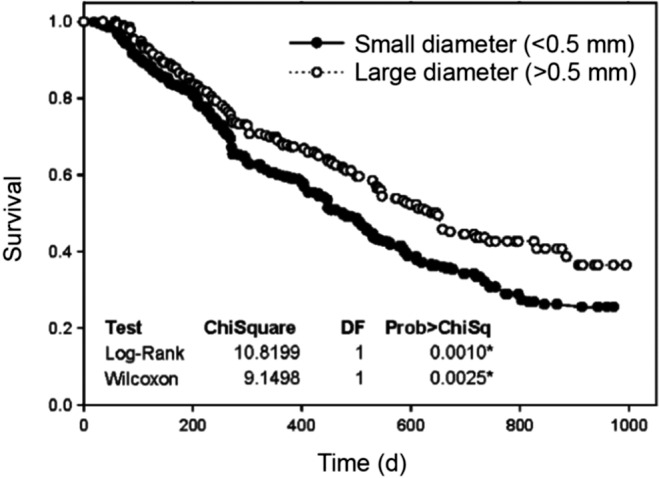
Survivorship curve and comparison between small and large diameter roots observed with the minirhizotron technique. Note that the point at which the survival probability reaches 0.5 (i.e. 50%) represents the estimated median lifespan for the population. Therefore, the median lifespan for small and large diameter roots would be interpreted as *c*. 460 and 630 d, respectively. Survivorship curves were generated using Kaplan–Meier regression. Each circle represents the timing of death of an individual root while roots that have unknown death dates treated as censored and estimated. Log‐Rank and Wilcoxon tests are used to identify significant differences in the survivorship behaviour between groups. Figure taken from McCormack *et al*. (2010).

See section **VI. 3. b. Overview of root observation methods** for a description of the minirhizotron technique. Additional discussion can be found in Rewald and Ephrath (2013) and in Iversen *et al*. (2012). From observations made in minirhizotrons, the timing of birth and death of individual roots is observed and the process is repeated for many individual roots. Birth is simply determined by the appearance of a new root from one session to another. Assessing when roots have died can be more difficult but common criteria often include changes in colour (e.g. blackening), shrivelling or disappearance. In all cases, the criteria used to determine root death in a given study should be clearly stated. Once the lifespans of many individual roots have been observed, the typical or average root lifespan of a population may be estimated. Analysis of direct observations is focused on comparing survivorship among species or treatment. The shape of the survivorship curve is determined by the individual lifespans of each root observed in the population. Kaplan–Meier regression and Cox Proportional Hazards are both common approaches used to assess patterns of lifespan and survivorship (Kaplan & Meier, 1958; Cox, 1972). Each has particular advantages and disadvantages. Kaplan–Meier regression is most commonly used to directly compare the survivorship patterns among two or a few groups (e.g. comparing between roots produced in shallow vs deep soil or comparing lifespan between species or sites). Fig. 20 presents survivorship curves for groups of small or relatively large diameter roots observed by McCormack *et al*. (2010) compared using Kaplan–Meier regression. Cox Proportional Hazards is most commonly used to determine the effect of different covariates on the lifespan of a particular group.

Under ideal circumstances, the time at which a root is constructed and later dies can be observed for every root in a study. However, this is often not the case as some roots are ‘lost’ due to accidental movement of the observation surface (e.g. shifting of the minirhizotron tube; Børja *et al*., 2009) and because there may be many roots that are still visible when a study ends and whose final death will not be directly observed. Those roots whose full lifespan from birth to death cannot be directly observed are considered as ‘censored’ and must be treated differently during analysis. If censored roots are removed from the analysis it would tend to shorten the estimated lifespan of the population as long‐lived roots would have a higher probability of still being alive at the end of a study. By contrast, including all censored roots but not treating them as censored in survivorship analysis may also bias outcomes (Børja *et al*., 2009). All major statistical platforms which facilitate standard survival analyses (i.e. Kaplan–Meier, Cox Proportional Hazard) allow for simple coding of censoring (i.e. yes or no).

Much like minirhizotrons, rhizotrons, root widows and rhizoboxes simply present a surface on which roots may grow and be visually observed. See section **VI. 3. b. Overview of root observation methods**. Unlike minirhizotrons, very little specialised equipment is required. So long as researchers can directly access the window surface (e.g. walking into a rhizotron building or opening a root box), the collection of root data from each can be the same. With repeated observations, researchers can identify when and where a root emerges on the transparent interface, and the root can then be repeatedly observed and recorded across later inspection dates. This may be done manually using pens and tracing, or by using scanners and digital images (Mohamed *et al*., 2017). Once collected they may be analysed in a manner analogous to that used for minirhizotron image analysis.

##### Indirect approaches

Indirect approaches, such as in‐growth cores and sequential coring (see section **VI. 3. a. Overview of root sampling methods**) or C isotope techniques, are used to infer or estimate lifespan, but do not directly observe lifespan. These methods often use estimates of fine‐root turnover rates (see section **XVI. 2. Root production, mortality and turnover rate**) and then infer average root lifespans by calculating the inverse of the root turnover rate. For example, if the estimated turnover rate is 0.5: yr^−1^, indicating that half of the root population is replaced in a given year, the resulting average lifespan would be reported as 2.0 yr, although it should be noted that lifespan is more commonly reported in days. However, calculating turnover rate and lifespan directly as the inverse of one another can be problematic, particularly in short‐lived root populations (see McCormack *et al*., 2014), and should be done with caution.

#### b. Future research directions

While root lifespan is a key aspect of plant strategies for below‐ground resource acquisition and conservation, and plant success, we are currently lacking understanding of how this trait trades‐off with other aspects of plant economics, how such trade‐offs determine optimal root lifespan (Eissenstat & Yanai, 1997) and how this is modulated by environmental conditions (Yanai & Eissenstat, 2002; McCormack & Guo, 2014).

### 2. Root production, mortality and turnover rate

*Root production* is the dry mass of roots produced per soil volume and per year (typical units: g m^−3^ yr^−1^). It can also be expressed per unit length of roots and/or per unit of ground area.

*Root mortality* is the dry mass of roots that died per soil volume and per year (typical units: g m^−3^ yr^−1^). It can also be expressed per unit length of roots and/or per unit of ground area.

*Root turnover rate* is the root dry mass production per dry mass of a given pool of roots over a period of time (typical units: yr^−1^). It can also be expressed based on numbers of roots or length of roots produced per total number or length of a given pool of roots, most often associated with minirhizotron observations.

While root turnover rate is also sometimes expressed as the root mortality per dry mass of a given pool of roots, root production and mortality are not necessarily synchronous processes and depending on the period considered, one process might be dominant over the other, which highlights the need to measure both root production and mortality throughout the year to accurately capture root turnover rates.

The distinction between dead and live roots, whether using minirhizotron images or the in‐growth core method, is a critical scientific problem, still unresolved, leading to large source of errors of root turnover rate (e.g. Lukac, 2012). In addition to the criteria often used with image‐based techniques such as minirhizotrons (e.g. colour, shrivelling), physical and textural clues such as friability and squishiness can also help determine root viability for roots collected from cores (see discussion under section **XVI. 2. b. Overview of root observation methods**). Although more time consuming, the vitality of sampled roots can also be assessed using tissue stains including fluorescein diacetate (FDA; e.g. Fernandez *et al*., 2013), tetrazolium chloride (TTC; e.g. Comas *et al*., 2000), and neutral red (Drennan & Nobel, 1996). Regardless, as methods such as minirhizotron or in‐growth core are more accurate to measure net root production, we recommend using root production instead of root mortality for the numerator estimation of root turnover rate.

The pool of roots (denominator) used for calculation is also critical. For example, maximum, mean and minimum values of standing root biomass are each independently used in the literature, which then leads to large differences in reported turnover rates (Brunner *et al*., 2013). These authors recommend using mean values of root mass because the resulting root turnover is more representative of an ecosystem being at steady state; using mean values instead of maximum biomass increased calculated root turnover by 30%. As previously discussed, turnover rate can also be calculated as the inverse of root lifespan (Lukac, 2012), although this should be done with caution (see discussion under section **XVI. 1. Root lifespan**).

Root turnover represents an important component of the global terrestrial C and nutrient cycles, such that roughly 11–28% of global annual NPP is transferred into the soil via turnover processes (McCormack *et al*., 2015a). However, root production and turnover are known to vary widely within and across plant species or biomes and are sensitive to local soil and climatic conditions (e.g. Fitter *et al*., 1998; Vogt *et al*., 1998; Eissenstat *et al*., 2000; Brassard *et al*., 2009; Lukac, 2012; Yuan & Chen, 2012a). Although empirical connections between rates of root and leaf turnover have been limited (Tjoelker *et al*., 2005; Withington *et al*., 2006), root turnover is sometimes expected to be slower than above‐ground turnover as roots may have more recalcitrant tissue than leaves (Jones & Donnelly, 2004). The analysis of 190 published studies showed that fine‐root turnover rates of shrublands (0.34 yr^−1^) is lower than that of trees, grasslands and wetlands (0.53, 0.56 and 0.55 yr^−1^, respectively), whatever the latitudinal geographic zones considered (Gill & Jackson, 2000). This means that all fine roots of forest, grassland and wetland ecosystems are renewed every 2 yr. More recently, Brunner *et al*. (2013) revaluated values of root turnover from 17 studies on European trees species by comparing different methods and calculations. Their estimates gave ranges of fine‐roots turnover from 0.86 ± 0.16 and 1.11 ± 0.21 yr^−1^ for beech, 0.88 ± 0.11 and 1.11 ± 0.14 yr^−1^ for Norway spruce, when maximum biomass and mean biomass data are used, respectively. Analysing 3 yr of root dynamics on 12 temperate tree species grown in common garden using minirhizotrons, McCormack *et al*. (2014) highlighted large interannual differences in root turnover within species, largely due to significant variation in root production between years. This suggests that fine‐root dynamics (production, mortality, and turnover) are likely to vary within a given species over time according to internal and external factors, necessitating long‐term studies. At a global scale, for example, Gill & Jackson (2000) highlighted the positive effect of mean annual temperature on root turnover across biomes, due to stimulation of annual root production. Nevertheless, this response may reflect the numerous indirect effects of temperature on abiotic and biotic factors that influence root longevity. In addition, no clear trend was observed for the effect of precipitation and potential evapotranspiration on root turnover in this study. However, Bai *et al*. (2010) found somewhat contrasting effects of warming and increased precipitation on root production and turnover with strong interactive effects. In addition, soil nutrient availability also has an effect on root turnover through changes of root production or root mortality (Eissenstat & Yanai, 2002), although results are inconsistent across studies. The inconsistencies may be caused by highly interrelated but nonadditive effects of key soil parameters, in combination with a plants’ C allocation pattern, on root growth and mortality. However, methodological issues may also play a role.

#### a. Measurement procedures

There are several methods for measuring fine‐root production and root mortality *in situ*, all of which have drawbacks which influence the reliability of the observation.

##### Minirhizotron

See section **VI. 3. b. Overview of root observation methods**. Root turnover rate (yr^−1^) is most often calculated from measurement of annual new root length production (cm cm^−2^ yr^−1^) divided by average living root length (cm cm^−2^). However, calculations may sometimes be based on standing root length that can include live and dead roots or may be based on maximum and minimum observations of standing length (see discussions in McCormack *et al*., 2014). The use of the minirhizotron technique to estimate turnover rates has historically been low, but is becoming increasingly common as minirhizotrons become more widely used. Minirhizotrons have the advantage of directly observing patterns of root growth while most other methods to calculate turnover do not. However, a negative aspect of the minirhizotron technique is that it is often biased to the most distal, ephemeral portion of the root system, especially in the years immediately after installation and therefore may overestimate root longevity and underestimate turnover rates.

##### Mass‐based root turnover rate

This is most often calculated as root production divided by average standing root mass (see section **XVI. 2. Root production, mortality and turnover rate**). Root production may be measured in one of several different methods including in‐growth cores, in‐growth‐donut or with sequential soil coring. As the in‐growth‐donut principle is very similar to in‐growth cores, it will not be described here, but full description is given by Milchunas (2009).

*In‐growth core.* See section **VI. 3. a. Overview of root sampling methods**. Once the in‐growth cores are retrieved and brought to the laboratory, roots and other below‐ground organs such as rhizomes are washed, oven dried at 60°C and weighed to measure root mass. Sum of root mass is used for calculation of mass‐based estimates of below‐ground net primary productivity (g m^−2^ yr^−1^), where m^−2^ is the surface area of the lateral surface area of the core (i.e. based on the core diameter). In some cases, calculations using root dry mass per unit of volume (i.e. based on the core diameter and height) may also be used but estimates based on surface area are also commonly used as a more direct analogue to measurements of above‐ground net primary productivity. The amount of roots grown into the in‐growth core is better controlled if the incubation duration is shorter than the estimated minimum root longevity plus decay rates. The period of incubation varies based on the specific research question and among ecosystems, from 2 wk for crops (Steingrobe *et al*., 2001) and steppe (Gao *et al*., 2008), up to 1–3 yr for forest ecosystems without the use of a net (Ostonen *et al*., 2005; Kubisch *et al*., 2016). For grassland sites, the period generally varies from 2 wk to 9 months (Allard *et al*., 2005; Gao *et al*., 2008; Bessler *et al*., 2009; Garcia‐Pausas *et al*., 2011; Li *et al*., 2011).

*Sequential soil coring.* This is one of the oldest methods to assess production of live and dead root mass *in situ* (McClaugherty *et al*., 1982). Soil cores are collected at different dates and different locations, generally over the course of a year or growing season. Live and dead roots are sorted after washing, oven dried and weighed separately (see section **VII. Root washing, sorting and storage**). From these data, fine‐root production, mortality and turnover can be estimated using several approaches. The ‘maximum–minimum’ method is based on differences between the maximum and minimum root mass measured during the sampling period. This approach is the least accurate as it substantially underestimates root dynamics and should be used only when significant changes are recorded between the maximum and minimum (Vogt *et al*., 1998). Although two to three sampling times are theoretically enough for this estimation, higher sampling frequencies (preferably covering hypothesised annual peaks/minimums in dynamic systems with, for example, distinct drought/wet seasons or warm/cold seasons) allow better estimation. Other methods require the sorting of fine‐root biomass and necromass. The ‘sum of changes’ method is typically based on positive differences in root mass between successive sampling times, over the entire sequential sampling period (Persson, 1978). Root production is calculated as the sum of all positive live root biomass increment, whereas root mortality is calculated as the sum of all positive dead root biomass increments. Root turnover is calculated as the ratio between live root production to the sum of live and dead root production. The ‘Decision matrix’ method by Fairley & Alexander (1985) and recently improved by Yuan & Chen (2013), assumes rapid fine‐root turnover and that even monthly sampling may miss some fine‐root production and mortality. With this method, all (significant) changes in both live and dead standing root mass between sampling dates are included in calculating production, mortality and decomposition. Finally, the ‘compartment–flow model’ method described by (Santantonio & Grace, 1987) is based on two compartments (alive and dead) and three flows (production, mortality and decomposition) and requires an additional estimation of root decomposition rate.

Overall, the root in‐growth method is less labour intensive than sequential root biomass coring because sampling is less frequent and sample preparation (washing, sorting) is less time consuming. Another advantage of the in‐growth core method is to control the age of the new roots at a given local patch. Repeated harvests at the same location also allow estimation of below‐ground net primary productivity that is not driven by spatial variability of the vegetation, which can be very high in patchy or grazed vegetation. However, there are several drawbacks of in‐growth cores including changes in the quality of soil substrate introduced into the core (see main drawbacks given by Dodd & Mackay, 2011), the unknown importance of disturbance due to repeating cutting of the core which sometimes elicits a wounding response by the plants, potentially inflating production numbers, and the conditions created by the in‐growth core that may favour new root to growth associated with competition‐free soil. Yet, according to Kubisch *et al*. (2016), compared with other techniques, the in‐growth core method has been found to produce rather conservative figures of fine‐root production in temperate forests (e.g. Hertel & Leuschner, 2002; Hendricks *et al*., 2006). For grasslands, there is no clear trend, however, in‐growth core estimates have been reported as either lower or higher than sequential soil coring estimates (Neill, 1992; Gao *et al*., 2008). These differences among sites indicate that the performance of a method may depend on ecosystem characteristics (Chen *et al*., 2016; K. Sun *et al*., 2016).

##### Additional methods of root turnover estimation: isotopes and budgeting

Some studies have successfully used various isotope‐based estimates of root turnover (e.g. labelling with ^13^C or ^14^C, or utilising signatures of atmospheric ‘bomb’ ^14^C; Gaudinski *et al*., 2001; Matamala *et al*., 2003; Milchunas, 2009). However, these methods can be difficult to interpret, are often biased to longer estimates of turnover, and have distinct limitations due to the frequent use of stored carbohydrates in the production of new roots (for more discussion, see Strand *et al*., 2008; Vargas *et al*., 2009; Adams & Eissenstat, 2014; Ahrens *et al*., 2014). In addition, budgeting approaches have been widely used, particularly in whole‐plant and ecosystem studies due to the requirement that all major pools and fluxes of a given element (i.e. carbon or nitrogen) are measured for a given system to estimate turnover (Aber *et al*., 1985; Nadelhoffer *et al*., 1985). These approaches can be useful but may have difficulty constraining estimates of nutrient fluxes accurately in natural ecosystems without thorough sampling and accounting of all relevant pools and fluxes. Consequently, they may lead to poor, frequently higher, estimates of fine‐root turnover rates. Despite this drawback, budgeting approaches have been used successfully in different ecosystems (e.g. forests, Nadelhoffer & Raich, 1992), and provided reasonable estimates of production in the shortgrass steppe ecosystem (Milchunas, 2009). While isotope approaches and budgeting approaches each have valuable uses, the other approaches including minirhizotron observation and coring techniques are preferred as more direct means to quantify root turnover.

#### b. Future research directions

As a consequence of limited, sometimes inaccurate knowledge on fine‐root turnover, our ability to predict C and nutrient cycling within plants and into the soils of terrestrial ecosystems in the context of climate and land use change is fundamentally limited. Direct and indirect methods assessing root production and root mortality are used to estimate fine‐root turnover, but their comparisons made at similar site or across sites revealed that there is an up to five‐fold discrepancy in estimates of fine‐root turnover, for example between data obtained on root longevity from minirhizotron and mean residence time using carbon isotopes (Guo *et al*., 2008b; Strand *et al*., 2008; Yuan & Chen, 2012b). The main problem comes from the absence of relatively easy, direct methods to measure root turnover (Milchunas, 2009; Lukac, 2012; Yuan & Chen, 2012b). Root mortality has been identified to be one major limitation to assess root turnover (Norby & Jackson, 2000). Therefore, as methods used to assess root mortality are often imprecise (e.g. root disappearance, corresponding to root decomposition for minirhizotrons; variable calculations for sequential cores; regression analysis for isotopes that rely on tenuous assumptions of root C origin), it is necessary to improve methodology to assess root mortality. Several spectroscopic methods have been used to determine root vitality (Nakaji *et al*., 2008; Picon‐Cochard *et al*., 2009; Rewald & Meinen, 2013) and are promising to improve understanding of root mortality. Another critical point concerns the definition of dead roots since parts of root tissue can be alive whereas other portions of tissue are not. The only methods able to distinguish live tissues and cells are vital staining, such as fluorescein diacetate (see section **XIII. 2. Percentage of viable root cells**), but significant challenges remain to make staining protocols work reliably and upscale from cells or tissue to root populations and entire root systems (Stūrīte *et al*., 2005; Richter *et al*., 2007).

### 3. Root phenology

Phenology is the timing of events and in the context of roots, the timing of individual root production and mortality. From this notion, four traits are typically of interest:

*Time of peak root production* is the time of the year at which root production is highest. Often expressed as in relation to a calendar date, but may more broadly refer to a month or season. Timing of peak root production may also be reported in relation to above‐ground phenophases such as leaf emergence, flowering, grain filling or fruit ripening.

*Time of peak root mortality* is the time of the year at which root mortality is highest. Often expressed as in relation to a calendar date, but may more broadly refer to a month or season. Timing of peak root mortality may also be reported in relation to above‐ground phenophases.

*Time of root growth initiation* is the calendar date at which root production starts increasing after a period of low or null growth. Time of root growth initiation may also be reported in relation to above‐ground phenophases.

*Time of root growth cessation* is the calendar date at which root production stops or becomes minimal after a period of high growth. Time of root growth cessation may also be reported in relation to above‐ground phenophases.

Under certain circumstances, for example in agrosystems where the timing of plant life cycles is manipulated, it might be recommended to use plant developmental stage or days after seeding as reference rather than calendar dates, which are mostly relevant in natural ecosystems.

By determining when roots are constructed and when they die, inferences can be made into how and when plants allocate resources throughout a growing season and across years. These traits provide direct clues into how plants may compete in natural environments (Harris, 1977), or how individual plants, plant species, and plant communities may respond to changing climate conditions (McCormack *et al*., 2015b; Radville *et al*., 2016; Schwieger *et al*., 2018). Among other environmental parameters, temperature has a role to play in seasonally cool to cold ecosystems (Burke & Raynal, 1994), while water is likely to be important in drought prone ecosystems (e.g. rain roots). However, the expected positive responses of root growth to increased temperature and water availability may be reversed in some ecosystems (Kou *et al*., 2018) indicating that the strength and direction of responses to basic environmental cues may shift among species and climate zones.

#### a. Measurement procedures

The same methods of direct observation used to determine root lifespan may be used to observe root phenology (i.e. minirhizotrons, rhizotrons, root boxes/windows and rhizoboxes), see section **XVI. 1. Root lifespan**. In addition, root phenology is also routinely measured at root phenotyping platforms, often using artificial media allowing for automatic root detection on images (2D) or tomographic techniques (3D) (Atkinson *et al*., 2019). In any system, researchers must record the date at which each individual root or amount of root length is first observed. Later, the date at which each individual root or amount of root length dies or disappears is also recorded. From this, key root phenology variables can be obtained: the timing at which roots begin to be observed (timing of initiation of root growth), the time when the most number or length of roots are first observed (timing of peak root production), and similarly the timing when the most roots or root length are observed to die and root growth ends for the growing season (timing of peak root mortality and growth cessation). When displaying patterns of root growth across a time series one of two approaches is usually used; either reporting absolute numbers of root production/mortality (i.e. root number or root length) or reporting proportional or relativised production/mortality. In the latter, root production or mortality numbers are scaled based on the relative proportion of root activity for a given time period. Using an example of production, the proportion may be calculated as the number of roots observed to be produced during a given time period (e.g. time between an observation date and the previous observation date) divided by the total number of roots observed to be produced across the entire year or observation period. In cases for which some experimental units (e.g. a species or site) have much higher total production and mortality than others, reporting proportional production and mortality makes it easier to compare differences across these experimental units. Similarly, reporting proportional results may make it easier to visualise differences in phenology within the same experimental unit, but across years if total root production varies widely. However, reporting proportional production also hides these important differences in total amounts of production and mortality that are often integral to interpreting root function and plant strategies. Therefore, when patterns of root phenology are reported as a proportion, researchers should also report data expressing total amounts of root production or mortality in text, tables or additional figures to enable full and accurate interpretations.

In many cases, direct observations of root phenology are preferred over indirect methods. However, there are some additional approaches that may be used to assess patterns of new root growth and activity that are also useful. First, repeated collection of root samples from bulk soil or soil cores may provide some inference into the timing of root growth, especially for monocultures of annual crops. It may also be possible to visually assess when there is an increase in the appearance of new roots, which may be lighter in colour than older roots (at least for a few days depending on rates of pigmentation), it may be similarly possible to miss pulses of new root growth even with relatively high sampling frequencies if root senescence occurs in parallel to root growth. However, this is often not a problem in crop plants, especially for sampling before flowering. As this approach requires repeated, destructive sampling it is most suited for larger, homogeneous sites. Patterns of root growth and activity may also be inferred by monitoring patterns of soil respiration (Cardon *et al*., 2002), which requires less disturbance than root harvesting. However, these efforts may be confounded with changes in soil conditions, specific root respiration rates, and microbial biomass that each can occur independently of new root construction, and great care must to taken when interpreting patterns of root phenology obtained this way.

#### b. Future research directions

While efforts to study root phenology are not new (Lyr & Hoffmann, 1967; Böhm, 1979), many aspects of below‐ground phenology remain unknown, including a mechanistic understanding of among‐species differences, or of the environmental cues most important for initiating and stopping root growth at a system level. Importantly, there is also a lack of knowledge on how root phenology is interconnected with the growth and phenology of other plant tissues in unmanaged herbaceous and woody plants (Steinaker *et al*., 2010), or how root phenology may be a mechanism for plant–plant competition (Harris, 1977). Provided that assessments of below‐ground phenology can be made in a robust manner with adequately high frequencies to identify important phenological transitions in relation to other plant components, there exist numerous opportunities to advance our understanding of plant growth, functioning and competition.

## 17 Root respiration and exudation

Root respiration and exudation are sources of plant‐derived carbon (C) that are continuously released by roots to soil and the atmosphere. Both root processes represent a major portion of woody and herbaceous plant C budgets, since a large fraction of the daily assimilated carbohydrates is expended in concurrent root respiration and exudation: root respiration can consume up to half (Lambers & Oliveira, 2019) and root exudation up to a third of the assimilated photosynthates (Liese *et al*., 2018). In interaction with other root characteristics, exudates can significantly influence the nutrient acquisition of plants, while root respiration influences a plant’s efficiency for soil resource uptake.

With the advancement of measurement techniques, the number of publications on root respiration and exudation has substantially increased since the 1990s, particularly on root exudation. Most studies investigate herbaceous plants, but a growing number focuses on trees, especially for root respiration. Geographical knowledge gaps remain for both South American, African, and Australian/Oceanian vegetation. Despite the importance of both root respiration and exudation for the carbon balance and nutrient acquisition of plants, the two processes are rarely measured and discussed together (but see Lambers, 1987), are likely to be due to separate expertise and comparably large measurement efforts.

### 1. Specific root respiration

Aerobic respiration is the complete oxidation of organic compounds with O_2_ serving as electron acceptor, with water and CO_2_ as final products; under hypoxic or anoxic conditions, anaerobic respiration (fermentation) results in ethanol (or lactate) and CO_2_ as final products. Root respiration (RR) typically includes aerobic as well as anaerobic (fermentation) respiration and can be described as:

‘all respiration derived from organic compounds originating in plants including the respiration of living root tissue, the respiration of symbiotic mycorrhizal fungi and associated microorganisms [and if methods based on isotopes are used] the decomposing organisms operating on root exudates and recent dead root tissues in the rhizosphere’ (Wiant, 1967; after Hanson *et al*., 2000).

While the ATP yield of fermentation is markedly less then that of aerobic respiration, ATP production efficiency under aerobic conditions can depend on the involvement on different metabolic pathways, such as alternative oxidase and uncoupling proteins, beside oxidative phosphorylation (e.g. Funayama‐Noguchi *et al*., 2020). See Plaxton & Podestá (2006) and Del‐Saz & Ribas‐Carbo (2018) for reviews on the (eco‐)physiology of plant respiration.

*Specific root respiration* is the amount of CO_2_ released or O_2_ absorbed by roots per unit root dry mass and time (typical units: µmol g^−1^ s^−1^) (frequent abbreviation: specific root respiration (RR_S_)). Instead of mass, RR_S_ can be expressed per volume (µmol cm^−3^ s^−1^), surface area (µmol cm^−2^ s^−1^) or length (µmol cm^−1^ s^−1^). This has the practical advantage of a more reliable size determination by image analyses compared with weighing very small (< 0.001 g) entities, and relates RR_S_ more closely to the number of cells, root length or surface (related to resource foraging and uptake, respectively).

Estimates of the contribution of RR to total soil respiration vary from 10 to 90% (Hanson *et al*., 2000), largely depending on environmental conditions, and RR consumes in extreme cases up to 75% of C allocated to roots (Majdi *et al*., 2007). These costs can be directly correlated with whole plant and above‐ground biomass accumulation (Rewald *et al*., 2016). The RR_S_ depends on three major energy‐requiring processes: (1) ion uptake (mostly N) and mobilisation (via exudation), (2) growth and defence, and (3) maintenance of living cells (Van der Werf *et al*., 1994). Root mycorrhizal symbionts and bacteria on the rhizoplane and in intercellular spaces can contribute to measured RR_S_ (Nielsen *et al*., 1998; Bulgarelli *et al*., 2012), while CO_2_ fixation in the roots by phosphoenolpyruvate carboxylase and exudation of carboxylates would lower respiratory CO_2_ release (Funayama‐Noguchi *et al*., 2020). The specific C and O_2_ costs per unit of ion uptake or root growth roughly indicate the C‐use and O_2_‐use efficiencies, therefore RR_S_ is one important variable determining the roots’ foraging/uptake efficiency (George *et al*., 2003; Lynch, 2015). Ion transport across membranes may account for 25–50% of RR_S_ (Lambers & Oliveira, 2019). RR_S_ allocated to root growth, maintenance and ion uptake processes has been estimated by modelling, requiring net nutrient uptake rate(s) and relative growth rates as additional input parameters (Poorter *et al*., 1991; Van der Werf *et al*., 1994; Nielsen *et al*., 1998; Nakamura & Nakamura, 2016).

RR_S_ is species specific, and depends on plant intrinsic factors such as root type and ontogenetic status as well the availability of photosynthetic assimilates. For example, respiration of young roots (lower order or finer roots) per unit of root mass is often much faster than that of old (coarse, lignified, lower RNC, greater RTD) roots, but can also differ among different types of root tips and root types (Palta & Nobel, 1989; DesRochers *et al*., 2002; Volder *et al*., 2005; Rewald *et al*., 2014). Measured RR_S_ of larger root entities such as branched root segments (i.e. entities encompassing several root orders) varies significantly among plant functional types, and RR_S_ is generally considered faster in fast‐growing species compared with in slow‐growing ones (Poorter *et al*., 1990; Rewald *et al*., 2014). However, some results suggest that, under nonlimiting conditions, the specific respiration rates of species‐specific root entities such as lateral root tips are rather similar within plant functional types (Asplund & Curtis, 2001; Jia *et al*., 2013; Rewald *et al*., 2014). For graminoids, for example, the effect of specific root entities on RR is illustrated by a significant positive correlation between RR and the proportion of ‘very fine’ roots (< 0.2 mm; Roumet *et al*., 2016). Therefore, if similar functional entities are identified, root systems may possess convergent functional units with similar RR_S_ in the same habitat (Burton *et al*., 2002). As RR_S_ strongly differs among root orders and the relative proportion of distinct root orders differs among species, RR of entire root systems cannot be inferred directly from RR_S_ of specific root orders; in species comparisons, researchers should consider on what root entity RR_S_ was measured (Rewald *et al*., 2014). However, Funayama‐Noguchi *et al*. (2020) recently reported that the overall expenditure of C for RR_S_ remained similar in two *Lupinus* spp. irrespective of the presence of cluster roots, pointing towards a tight regulation of whole root system C consumption across different entities via closely controlled metabolic pathways.

RR_S_ is subject to influences of environmental factors, including soil temperature, moisture, nutrients (especially N), excess ions (such as Cl^–^), and O_2_ availability. With an increase in soil temperature, RR_S_ increases and is generally modelled as increasing exponentially with temperature, with a Q_10_ of about 2. However, this is true only over a limited temperature range, and large differences between species and root types regarding Q_10_ and the rate/degree of temperature acclimation have been reported (Cox, 1975; Palta & Nobel, 1989; Atkin *et al*., 2005, and references within; Huang *et al*., 2005). At moderate to high soil temperatures, RR_S_ decreases as soil moisture deficits increase (Bryla *et al*., 2001; Huang *et al*., 2005; Jarvi & Burton, 2013). Root O_2_ deficiency, for example during waterlogging, usually restrict RR_S_ (Ben‐Noah & Friedman, 2018); the ‘critical O_2_ pressure’ is defined as the lowest O_2_ concentration to support maximum RR_S_ (Armstrong *et al*., 2009). In some cases, RR_S_ is reduced by high CO_2_ concentrations (Qi *et al*., 1994; Burton *et al*., 1997; but see, e.g. Burton & Pregitzer, 2002; Drake *et al*., 1999). RR generally increases when roots are suddenly exposed to increased ion concentrations (‘salt respiration’). Regarding N forms, acquisition and assimilation of NH_4_^+^ often requires less C than that of NO_3_^–^ (Bloom *et al*., 1992; Rewald *et al*., 2016). However, as preferences for N forms differ widely and NO_3_^–^ assimilation may take place in shoots (while organic acids produced during the reduction of NO_3_^–^ in leaves can be decarboxylated in roots), the relation between RR_S_ and N form is highly context dependent and often results in modified respiratory quotients (RQ; Lambers & Oliveira, 2019). RQ of herbaceous species were reported to range between *c*. 0.8 (NH_4_^+^ fed) to *c.* 1.0–1.6 (NO_3_^–^ and N_2_ fixing, with a trend to greater values in the latter). We know less on the influence of other soil nutrients on RR_S_, although a lack of certain nutrients as well as excessive concentrations of salts and metals alter RR_S_ rates (Trolldenier & von Rheinbaben, 1981; Llamas & Sanz, 2008; Otgonsuren *et al*., 2016).

RR_S_ feature a strong temporal variability based on plant phenological and environmental changes (Ruehr & Buchmann, 2010). This diurnal and seasonal variation is highly species‐specific (Cox, 1975; Trolldenier & von Rheinbaben, 1981; Widén & Majdi, 2001) and are likely to relate to the amounts of assimilates available for RR_S_ at given time points (Ekblad & Högberg, 2001; Noguchi, 2005; Xu *et al*., 2008; Kuzyakov & Gavrichkova, 2010).

RR_S_ of grass, herb and tree roots show a positive linear correlation with RNC (Pregitzer *et al*., 1998; Volder *et al*., 2005; Atkinson *et al*., 2007; Ceccon *et al*., 2016). As RNC is closely related to the amount of protein, it can serve as a predictor of root activity. Furthermore, Roumet *et al*. (2016) reported negative correlations of RR_S_ with traits related to tissue quality (C : N and Lignin : N ratios, decomposability). RR_S_ is also frequently related to root diameter classes, with lower‐diameter roots having faster RR_S_ (Cox, 1975; Pregitzer *et al*., 1998; Marsden *et al*., 2008b; Makita *et al*., 2009). However, other morphological traits such as SRL, RTD or RDMC may be better suited to predict the variability in RR_S_ across (fine) root systems, with RR_S_ declining markedly with increasing RTD and RDMC and increasing with increasing SRL (Rewald *et al*., 2014; Lai *et al*., 2015; Roumet *et al*., 2016; but see Makita *et al*., 2012a). Because RR_S_ is largely related to the amount of living cells (Veen, 1981; Scheurwater *et al*., 2000), any trait representing this amount more closely (e.g. living cortical area; Lynch, 2015) is a good predictor of RR_S_.

#### a. Sampling, storage and processing recommendations

##### *Ex situ* measurements

**Fig. 21:**
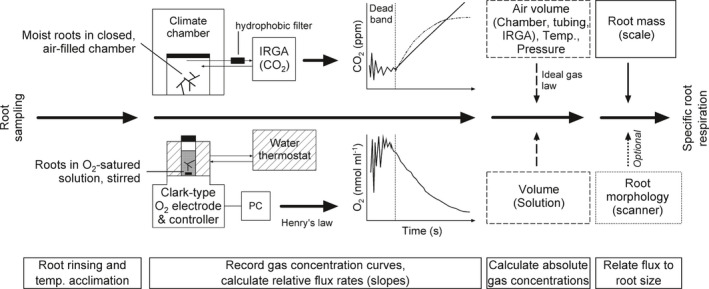
Typical workflow of specific root respiration (RR_S_) measurements with closed‐chamber, gas‐phase CO_2_ (upper) and aqueous‐phase O_2_ (lower) gas‐exchange systems. (1) Roots are retrieved, rinsed and allowed acclimate to temperature. (2) Roots (or root segments) are inserted into temperature‐controlled, closed chambers to record changes in relative CO_2_/O_2_ concentrations, using either an infrared gas analyser (IRGA) or an O_2_ electrode. A ‘dead band’ illustrates the importance of chamber equilibration (mixing and temperature); subsequently, the slope of the gas concentration curve (δCO_2_ or δO_2_/δt; ppm s^−1^) is calculated by either linear or exponential regressions methods. For gas‐phase measurements, linear regressions often underestimate the slope; for aqueous‐phase O_2_ measurements, a quasilinear area of the curve is frequently visually selected for slope calculations. (3) The absolute gas concentration (mol m^−3^) is calculated by using either the ideal gas law (gas‐phase measurements; in retrospect) or Henry’s law (for dissolved gas; often integrated in O_2_ electrode software) together with information on chamber/solute volume (m^3^), air pressure (Pa) and temperature (K). (4) Absolute flux rates (µmol s^−1^) are divided by root dry mass (g), or other size parameters, to calculate RR_S_ (e.g. µmol g^−1^ s^−1^). See text for details.

For *ex situ* approaches, that is direct measurement of CO_2_/O_2_ fluxes in chambers, excised root samples must be retrieved (Fig. 21). This has the advantage that RR_S_ can be measured with limited confounding influence due to the presence of soil, and that clearly defined entities (root types, root orders, etc.) of root systems can be sampled. To obtain enough intact root samples, soil monoliths or relatively large cores should be sampled. Roots are subsequently either rinsed (with tap water) or brushed free of loose soil and organic matter (Makita *et al*., 2012b). Root samples can be dissected further to fit within chambers or to measure specific root entities; for woody root systems, a separation into fine and coarse roots is recommended. Root excision is one possible reason for the overestimation of RR_S_, due to the faster respiration by ‘wounds’ (Rakonczay *et al*., 1997; Marsden *et al*., 2008a; Makita *et al*., 2012b), although other studies have reported that effects of excision on RR_S_ are small (Marshall & Perry, 1987; Lipp & Andersen, 2003). As carbohydrate levels in roots start to decline after excision, time between sample retrieval and measurements is usually, as a general precaution, highly standardised between samples and kept as short as possible (‘immediately’ or often < 1 h; Bloom & Caldwell, 1988). However, the results of Lak *et al*. (2020) illustrate that storage up to 5 h results in only minor deviations compared with measurements 30‐min postrinsing/‐excision if roots were kept hydrated. By contrast, storage temperature had a larger effect on measured RR_S_, with storage at room temperature or refrigerated being preferable over storage at temperatures above the target temperature during measurements.

##### Continuous measurements *in situ*

(Continuous) respiration measurements on undetached roots *in situ* or in specific experimental settings are scarce. For example, Bryla *et al*. (2001) and Dannoura *et al*. (2006) measured CO_2_ efflux of intact roots in sand or weathered granite (‘near‐zero CO_2_ efflux’) in a glasshouse and the field, respectively. Huang *et al*. (2005) used soil CO_2_ efflux in a chamber with no roots as blank instead of sand. While Bryla *et al*. (2001) and Huang *et al*. (2005) inserted uncovered root branches into substrate‐filled chambers, Dannoura *et al*. (2006) replaced the native soil. Both methodologies require careful handling of roots; desiccation and injuries need to be avoided. In any case, root branches need to be harvested at the end of experiments to determine RR_S_; however, a potential confounding effect for long‐term measurements is the root growth/turnover taking place during measurements.

#### b. Measurement procedures

Several methodologies can be used to determine RR_S_, direct measurements or indirect calculations. Criteria for deciding which system is most suitable for a particular application (in addition to costs) include the gas to be measured (CO_2_ or O_2_); the medium for measurement (aqueous or gaseous); the size and metabolic activity of the sample in relation to the range of gas concentrations (analyser resolution and range); whether or not fast transient responses are to be measured (analyser response time); the need for continuous or intermittent measurements (open or closed systems); and whether or not destructive sampling can take place. If financially feasible, several units of one system can be operated in parallel to avoid long time lags between sampling and measurements and to process the often relatively large numbers of replicates required (*n* ≈ 15–30). Key methodologies are described in the following; but see the comprehensive review by Hunt (2003) for further information on modes of operation and pitfalls outlined for respiration measurements (although based on leaves).

##### Direct measurements: CO_2_ efflux

Several methods have been used to measure CO_2_ efflux rates, including infrared gas analysers (IRGA), gas chromatography, and KOH absorption of CO_2_ (Van Cleve *et al*., 1979). These days, however, various brands of IRGAs are commonly used to determine root CO_2_ efflux rates in laboratory and field settings (Fig. 21). Both open and closed‐chamber settings are used: in an open system, respiration rates are determined by the difference in the amounts of CO_2_ entering and leaving the gas‐phase cuvette, while in a closed system δCO_2_ is monitored over a certain time (e.g. 3–10 min) or threshold (e.g. 50 ppm) (Ramos Tamayo *et al*., 2001; Hunt, 2003). The advantage of open systems is the relatively constant CO_2_ concentration and the ability to set the initial CO_2_ concentration (e.g. close to soil CO_2_ concentrations). However, closed‐chamber systems are most commonly used (Fig. 21, upper), partially because of lower costs (compared with differential systems), straightforward calibration and operation, and the more suitable design for measuring low CO_2_‐based RR_S_ (since the gases being exchanged accumulate or decline over time) once chamber equilibration has been reached (‘dead band’; Fig. 21).

##### Direct measurements: O_2_ uptake

Development of the Clark‐type O_2_ electrode in the 1960s has allowed the determination of oxygen‐depletion rates in closed cuvettes (González *et al*., 2001; Hunt, 2003). Measurements are either conducted on roots in aqueous (using oxygen‐enriched solutions, mostly (tap) water or (buffered) nutrient solutions; Fig. 21, lower) or gas‐phase in closed‐chamber systems (Jia *et al*., 2013; Rewald *et al*., 2014). Needle‐type O_2_‐microelectrodes that can be inserted directly into roots, and differential O_2_‐sensors for open chamber systems are not covered here. Clark‐type O_2_ electrodes must be prepared (electrolyte, spacer paper, polytetrafluoroethylene or fluorinated ethylene propylene membrane) and calibrated (with air‐saturated water and under zero oxygen conditions) daily, often the most time‐consuming aspect of O_2_‐electrode use. After equilibration of both electrode and samples (‘dead band’), oxygen‐concentration curves are recorded in mV over several minutes (e.g. 5–10 min; depending on O_2_‐consumption rates). As the electrochemical reaction at the electrode consumes O_2_ too, care must be taken to compensate for this consumption and to stay above the ‘critical O_2_ pressure’ during measurements. In addition, solutions must be stirred (magnetic stirring bar) thoroughly to reduce the depletion of oxygen boundary layers at both the root surface and the electrode (Asplund & Curtis, 2001; Armstrong *et al*., 2009).

##### Direct measurements: CO_2_ and O_2_

As the ratio between released CO_2_ and consumed O_2_ (RQ is not constant, but depends on the substrate of respiration and environmental conditions such as a N source), the results of either measurement cannot be translated instantly in all cases (Del‐Saz & Ribas‐Carbo, 2018). Measuring net CO_2_ and O_2_ fluxes in parallel is not standard procedure (but see Rachmilevitch *et al*., 2006; Meier *et al*., 2013a).

Faulty RR_S_ measurements determined by either of the ‘direct’ methods often relate to poor calibration, leaks and diffusion through chamber materials and tubing, and insufficient mixing. Furthermore, while *ex situ* measurements are generally conducted under temperature‐controlled conditions, using water baths or climate cabinets, an insufficient temperature equilibration of specimens will contribute to greater variation and systematic changes in measurements (Atkin *et al*., 2009; Lak *et al*., 2020). Under varying temperature conditions, individual samples’ temperature should be logged and RR_S_ should be temperature‐normalised using Q_10_ values (Makita *et al*., 2012a). Furthermore, the exact volume of the chamber (headspace) or amount of solution (Fig. 21), or flow rates (in open chamber systems), has to be known to calculate absolute flux rates (Hunt, 2003). Using either the ideal gas law (gas phase) or Henry’s law (dissolved gas in solutions), together with information on temperature (K) and air pressure (Pa), the chamber/solute volume (m^3^) is key to calculate the amount of CO_2_ or O_2_ molecules (mol; Fig. 21). For gas‐phase, closed/static chamber approaches, the choice of a linear or exponential regression function (Fig. 21) for calculating slopes of gas concentration curves can significantly influence the results, with underestimations by using linear regressions, as frequently shown for soil gas efflux measurements (Kutzbach *et al*., 2007; Kroon *et al*., 2008; Forbrich *et al*., 2010). For O_2_‐depletion measurements, a quasilinear section of the curve is often visually selected for slope calculation (in the instruments’ software). The methodology of (slope) calculation should be reported to increase the reproducibility of RR_S_ measurements.

##### Indirect determination

Indirect methodologies are generally based on separating root and soil microbial contributions to soil respiration.

*Basal respiration.* The basal method (Marshall & Perry, 1987) uses a two‐compartment soil respiration model; a volume of soil is isolated with a trenched plot and all above‐ground biomass is removed. Total soil respiration is measured periodically with a closed‐chamber IRGA system on trenched and nontrenched plots. As the trench excludes living roots, total soil respiration decreases, representing soil microbial, heterotrophic respiration (R_h_). RR_S_ is calculated by subtracting R_h_ from total soil respiration measured on nontrenched locations (Lalonde & Prescott, 2007). When applying this method, care has to be taken, as several factors might strongly influence R_h_ including soil heterogeneity, soil disturbance during trench installation (e.g. compacting or mixing), decomposing roots, and modified water and nutrient conditions (by the lack of uptake by roots).

*Isotopes.* In isotope‐based methods, either variation in the natural ^13^C : ^12^C ratio (Ekblad & Högberg, 2001; Cheng *et al*., 2005) or ^13^C labelling (Johansson, 1991; Swinnen *et al*., 1994) has been used for separating total soil respiration into RR and R_h_ respiration. These methods permit continuous measurements with the least amount of disturbance to the soil and roots; however, they generally yield lower rhizosphere contributions than other methods. Furthermore, there are uncertainties about how quantitative these methodologies are when used in the field (Hanson *et al*., 2000). See section **XVII. 2. Root exudation** for further details.

For both described indirect methods, root biomass (below the measurement area) must be sampled in conjunction with respiration measurements to calculate RR_S_. Measuring soil temperature and moisture is also necessary to interpret RR data.

#### c. Future research directions

In general, traits related to C costs for root maintenance respiration and nutrient uptake and assimilation should gain more attention compared with C assimilation in future studies. The quantitative description of RR_S_ of key species to changes in soil temperature and moisture (and atmospheric deposition of N and pollutants) is particularly important for plant growth modelling, especially under future climates. Furthermore, an enhanced understanding of how functional entities within‐root systems shape RR_S_ of root branches or the whole root system, and their temporal and cue‐triggered dynamics, is key to enhance our understanding of plant functioning in general and C budgets in particular.

Several emerging technologies are likely to play an important role in future RR measurements. The planar optode method depends on the quenching by O_2_ of luminescence from organometallic ruthenium compounds. Optodes may be used in gas phases or aqueous phases and advantages over O_2_ electrodes include no oxygen consumption, fast response time, μm‐level of resolution and spatial coverage (mm^2^ to cm^2^), and an extended measurement period (seconds to days). Although the methodology has not yet been used to determine RR_S_ (e.g. related to surface area), it has a large potential to resolve spatial and temporal complex respiration patterns at root–soil (rhizosphere) interfaces and to relate RR to root growth, nutrient uptake and exudation (Tschiersch *et al*., 2012; Blossfeld *et al*., 2013; Rodeghiero *et al*., 2018).

Cavity‐enhanced Raman multigas spectrometry (CERS) can be used to estimate the respiratory quotient, an indicator of the nature of the respiratory substrate (Rachmilevitch *et al*., 2006). Recently, Hanf *et al*. (2015) introduced a methodology to simultaneous and rapid *in situ* monitoring of O_2_ and CO_2_ fluxes of leaves. It is envisioned that this technology, although expensive, can play an important role in advancing research on RR_S_.

### 2. Root exudation

Root exudation is the release of soluble, low‐molecular‐weight organic C compounds from roots into the surrounding soil. It occurs either because of the leakiness of root cells and passive diffusion down a concentration or electrochemical potential gradient between root cells and the soil solution (basal root exudation) or by secretion of specific carboxylates via anion channels or vesicle transport (upregulated root exudation). Two traits can be measured:

*Net root exudation rate* is the amount of soluble low‐molecular‐weight organic C compounds released from roots into the surrounding soil, minus reabsorption and microbial consumption, per root mass and unit of time (typical units: µmol C g^−1^ h^−1^). It can also be expressed per root length (µmol C cm^−1^ h^−1^) or soil mass (mmol C g^−1^ soil h^−1^).

*Gross root C efflux rate* is the amount of all low‐molecular‐weight organic C compounds released from roots into the surrounding soil (which potentially includes root necromass and symbiont cells) per soil mass and unit of time (typical units: g C kg^−1^ soil yr^−1^). It can also be expressed per surface area (kg C ha^−1^ d^−1^).

Depending on project aim and research method, the whole (fine) root system, terminal intact fine‐root segments or individual root orders are considered. Root exudates are mainly released at the root apex and at lateral branching points; specific compounds can be released from the root hair region (Czarnota *et al*., 2003; Yan *et al*., 2004). The development of specialised cluster roots is accompanied by a short yet intense burst of root exudation (Watt & Evans, 1999; Shane *et al*., 2003).

The major components of root exudates are low‐molecular‐weight or polymeric carbohydrates, amino acids (among them phytosiderophores), organic acids, secondary metabolites (e.g. phenolics and terpenoids), enzymes and inorganic ions (Neumann & Römheld, 2007). Root exudates influence the size and sink strength of soil organic matter, as: (1) they are preferentially used by soil microbes as substrate, which stimulates these microbes to decompose also recalcitrant soil organic C via a priming effect (Kuzyakov *et al*., 2000; Phillips *et al*., 2011, 2012; Meier *et al*., 2017), (2) they are chemical signals for mycorrhizal fungi (Akiyama *et al*., 2005) and rhizobia (Currier & Strobel, 1976), which increase the accessibility of nutrients (Smith & Read, 2008; Courty *et al*., 2010; Fellbaum *et al*., 2012), (3) the exuded exoenzymes degrade fungal soil organic matter directly (Weisskopf *et al*., 2006), and (4) they increase soil aggregation (Traoré *et al*., 2000; Baumert *et al*., 2018). Under climate change, enhanced significance is attributed to root exudation, to enhance microbial priming effects by upregulated root C exudation, which is assumed to delay progressive N limitation (*sensu* progressive N limitation hypothesis; Luo *et al*., 2004) under elevated CO_2_. Root exudation can be regulated up in response to metal toxicity, nutrient deficiency (e.g. of P, N, Fe, Zn or Mn), and the presence of neighbouring plants and specific rhizosphere microbial taxa (Jones *et al*., 2004; Bais *et al*., 2006; Marschner, 2012).

In addition to their role in soil organic matter build‐up, root exudates also have allelopathic effects upon neighbouring plants and they may influence the growth and composition of the rhizosphere microbial community, either due to their antimicrobial and antifungal activities or due to the stimulation of pathogenic, saprotrophic and mycorrhizal fungi and rhizobia. The quantity and chemical composition of root exudates can be influenced by plant age and species, genotype, distance along the root, environmental conditions (e.g. light intensity, temperature, nutrient availability, mechanical impedance), and the presence of beneficial or pathogenic soil microbes (Farrar & Jones, 2003; Neumann & Römheld, 2007). Generally, plant–plant and plant–microbe interactions lead to a shift in the chemical composition of root exudates (Akiyama *et al*., 2005; Perry *et al*., 2005; Jousset *et al*., 2011), while light intensity (Nakayama & Tateno, 2018), temperature (Boone *et al*., 1998), elevated CO_2_ (Phillips *et al*., 2011; Phillips *et al*., 2012), nutrient deficiency of for example P or Fe (Ward *et al*., 2011; Yin *et al*., 2014), water stress (Preece *et al*., 2018), mechanical impedance (Boeuf‐Tremblay *et al*., 1995), and pathogenic fungi (Meier *et al*., 2013b) can increase the amount of exuded C. Among the abiotic influences, daily amount of photosynthetically active radiation (as the driver of C assimilation) has been suggested as a key factor for the quantity of root exudation (Phillips *et al*., 2008; Kaiser *et al*., 2010; Nakayama & Tateno, 2018). Root exudation also increases with increasing total root length and increasing RBI (Xu & Juma, 1994; Groleau‐Renaud *et al*., 1998; Darwent *et al*., 2003; Yin *et al*., 2013). Accordingly, fibrous roots in the topsoil had faster root exudation rates than pioneer roots in the subsoil (Tückmantel *et al*., 2017).

Methods for the collection of root exudates have developed along two main trajectories: a (semi‐)artificial experimental trajectory that aims at sterile controlled collection conditions for the understanding of mechanisms, and an *in situ* ecological trajectory that attempts to cover the natural complexity of root–rhizosphere interactions, for example in mature forests. Due to the labour‐intensive nature and methodological constraints associated with measuring root exudation directly, root exudation is sometimes inferred from the gap in the (root) C budget. However, since this measure is at best inaccurate and cannot be confidently associated with root exudation, we strongly discourage this practice. Since this handbook aims at collecting basic trait measurement methods related to root ecology, we will focus on comparatively simple methods for analysing root C exudation in natural environments only. An overview of laboratory applications and modelling approaches can be found elsewhere (e.g. Luster & Finlay, 2006; Neumann *et al*., 2009; Vranova *et al*., 2013; Downie *et al*., 2015; Oburger & Schmidt, 2016). Among the most commonly used methods used for measuring the root exudate C flux *in situ* are: (1) the collection of soluble root exudates in hydroponic systems or trap solutions and (2) the analysis of C partitioning throughout plant and soil compartments in labelling or girdling experiments. Root C efflux estimates from labelling or girdling studies often exceed root exudate estimates from hydroponic collections, which may reflect the different processes targeted with these methods: the soil pool recovered in whole‐plant C‐labelling studies probably includes root necromass and symbiont cells, and therefore is a result of rhizodeposition, while the collection of exudates in trap solutions includes reabsorption (influx) and microbial consumption of the exuded compounds and, therefore, represents net root exudation. As an additional approach for studying the root exudate C efflux, the leaf Mn concentration has been recently identified as a proxy trait for carboxylate exudation, since the exuded carboxylates mobilise soil Mn and increase its availability, which the roots poorly control for during their nutrient uptake (Lambers *et al*., 2015; Pang *et al*., 2018; Zhou *et al*., 2020).

#### a. Sampling, processing and measurement procedure

##### Net root exudation rate

Net root exudation rate can be measured using the culture‐based cuvette method, a modification of hydroponic sampling adapted by Phillips *et al*. (2008) for field use. Hydroponic sampling of root exudates avoids changes of the exudation profile due to microbial decomposition and sorption of exudates to the soil matrix. The cuvette method allows the collection and compositional analysis of root exudates from mature trees in their natural environment, and therefore is the method that is the closest to investigating the natural exudate composition and exudation patterns of mature trees. However, it must be kept in mind that they may still differ from their original pattern, since root exudates are sampled in a medium that differs from a natural soil environment (e.g. with respect to nutrient availability, aeration and the interaction with soil microbes).

In the culture‐based cuvette method, the sampled fine‐root system (e.g. the two to three most distal root orders) developed in soil remains attached to the plant during the whole sampling process. Extraction of the fine‐root system from soil must be conducted carefully to maintain the integrity of the (mycorrhizal) root system as much as possible: root systems are excavated with soft brushes and fine forceps and adhering soil particles are removed using deionised water, 0.5 mM CaCl_2_, or autoclaved dilute nutrient solution. Species identity of the root system must be confirmed with a morphological key or by tracking the root system back to the mother tree.

The root system can recover from the excavation stress in moist, sandy soil overnight. On the next day, the soil‐free and cleaned intact root system is placed into a sterile cuvette (preferentially a glass syringe or alternatively a plastic syringe from which the plunger is removed), back‐filled with sterile glass beads (≤2 mm in diameter) that provide the mechanical impedance and porosity of soils, and moistened with sterile, dilute nutrient solution. The cuvette is closed with a rubber septum and covered by parafilm, while ensuring the integrity of the emerging root. Sterile cuvettes with glass beads and culture medium (i.e. without roots) are treated similarly and included as control. The experimental set‐up is covered to dampen temperature differences to the surrounding soil and roots can equilibrate in the cuvette environment for 48 h. Subsequently, cuvettes are cleaned by being flushed three to five times with the culture medium using a low‐pressure vacuum. New culture medium is added, and the root systems are equilibrated. After the incubation period, the trap solutions containing exudates are collected from each cuvette, filtered immediately through a sterile 0.22 μm syringe filter, and kept cool for transport to the laboratory, where they are either analysed immediately or concentrated by freeze‐drying and stored at −20°C for later analyses.

There are two issues that must be considered when collecting root exudates with the culture‐based cuvette method:

1. Root exudation is influenced by the concentration or electrochemical potential gradient between roots and the soil solution. Consequently, the nature of the trap solution will influence the amount of root exudates and the concentration of specific compounds (Kuijken *et al*., 2015). Sampling of root exudates in water for short periods may lead to potential overestimation of exudation rates as a consequence of diffusion between root cells and the low ionic strength solution (Neumann & Römheld, 2007; Vranova *et al*., 2013), while sampling in water over a long time period (> 24 h) may lead to an underestimation of exudation rates due to low nutrient availability in the solution (Jones & Darrah, 1993). Exudation generally increases and influx decreases with the strength of the solution (Vranova *et al*., 2013). Ideally, the trap solution should resemble the soil solution as closely as possible, for example, by using a dilute nutrient solution (pH‐adjusted) that does not interfere with subsequent chemical analyses. Occasionally, ≥100 µM Ca^2+^ is added to the trap solution to ensure root membrane integrity (Vranova *et al*., 2013).
2. Time is a very critical factor when sampling root exudates in steady state *in situ* conditions. The quantity of exuded compounds will increase over time which facilitates chemical analyses, while concurrently increasing the number of invading microbes. Ideally, the exposure time is just long enough to yield enough signal for later analyses (which is species specific and site specific), but short enough to avoid significant microbial consumption. Sterile conditions in the trap solution are sometimes maintained by the addition of sterilising or protecting agents (e.g. antimicrobial compounds, K_2_SO_4_, Micropur® containing Ag^+^), yet rhizotoxic effects on the plant and the efficiency of their force in soil are disputed. Some authors note a negligible effect of microbial degradation for sampling periods of less than 24 h (Jones & Darrah, 1993), while others find significant effects (Kraffczyk *et al*., 1984; Kuijken *et al*., 2015). The ideal time should be estimated from prior time series of root exudate collection in combination with the intended chemical analysis. In addition, the diurnal cycle of photosynthate production has to be considered when planning the time for root exudate collections due to the overriding influence of solar radiation on root exudation (Nakayama & Tateno, 2018): ideally, a full diurnal cycle is covered by the exudate collection.

Subsequent to exudate collection, basic analyses include the measurement of the concentration of dissolved organic C in the trap solution with a total organic carbon (TOC) analyser and weighing of the dried root system. Net root exudation rates (gross root exudation minus reabsorption and microbial consumption) are then calculated as the total amount of C flushed from each root system over the incubation period divided by the total root mass and incubation time (mmol C g^−1^ h^−1^). For expression of net root exudation rate per unit root length, see description of root length measurements under section **XII. 2. Specific root length**. Advanced analyses include the identification of the exuded metabolites by enzymatic assays, capillary zone electrophoresis/mass spectrometry, or ecometabolomics.

##### Gross root C efflux

Gross root C efflux can be measured by several methods including isotope labelling and natural abundance methods.

*Isotope labelling with ^13^C*. Isotope labelling is used to trace the partitioning and fate of assimilated C in plants, soil and associated microorganisms. This method estimates total rhizodeposition, that is the release of root cap and border cells, insoluble mucilage, soluble root exudates, and volatile organic C (including root respiration), the C flux to mycorrhizal fungi, and the death and lysis of root cells (Jones *et al*., 2009). Labelling can be conducted as a pulse, which can be repeated several times during the growing season (Whipps, 1990; Bromand *et al*., 2001; Kuzyakov, 2001), or conducted continuously, which is more expensive, but results in constant isotopic ratios in all metabolites. The former can be used for studying the distribution and diffusion of root C efflux, while the latter is more appropriate for the estimation of the total amount of C transferred from plants to soil (Ge *et al*., 2015). The separation between root‐derived C and soil organic C is necessary and can be achieved by labelling of shoots in a ^13^CO_2_ atmosphere. Isolation of the headspace from the atmosphere has to be achieved, for example by an airtight Plexiglas chamber (Kuzyakov *et al*., 1999, 2001), and root and shoot zones have to be physically separated, for example by a monofilament screen or by low melting paraffin overlaid by silicon paste. A sufficient amount of ^13^C is injected as 99 atom‐% ^13^C‐CO_2_ into the Plexiglas chamber, while tracking the absolute CO_2_ concentration in the chamber. The amount of label needed can be estimated from the strength of the label, the daily assimilation rate of the investigated plant species, and the targeted bulk soil enrichment.

Labelling can take place for short (1–3 h, pulse labelling), repeated, or long periods (continuous labelling) according to the research question. During the labelling, that is the closure of the hoods, the climatic conditions inside the chamber must be controlled. Subsequently, the remaining unassimilated ^13^CO_2_ from the Plexiglas chamber is continuously pumped through a 1 M NaOH solution to trap CO_2_ and its concentration in the solution is analysed by isotope ratio mass spectrometry. Partitioning of the assimilated ^13^C can be identified from the ^13^C amounts in various pools (shoots, roots, microorganisms, dissolved organic carbon (DOC), and soil; see Pausch & Kuzyakov, 2018). To minimise the error of ^13^C discrimination, the difference in the atom% ^13^C value of the soil and root fractions from the labelled sample and an unlabelled control (atom% ^13^C excess) should be used for the calculation of rhizodeposition. The amount of C derived from gross rhizodeposition is estimated from the ratio of atom% ^13^C excess in soil to atom% ^13^C excess in roots and multiplied by the C concentration in the soil fraction. Rhizodeposition can be standardised by the amount of assimilated ^13^C to account for tracer losses during labelling, photosynthetic ^13^C discrimination and shoot respiration.

*Isotope natural abundance*. The natural abundance technique uses the isotopic signature difference between C_3_ and C_4_ plants to estimate the amount of C released by roots to soil: C_4_ plants are planted on C_3_ soil or *vice versa* (Cheng, 1996; Rochette & Flanagan, 1997). The method is based on the different δ^13^C signature of *c*. 27 ‰ in C_3_ plants and of *c*. 14 ‰ in C_4_ plants (Tieszen & Boutton, 1989), and consequently also in significant differences in the respective soils. It can easily be used under field conditions. The method is dependent on significant changes of the δ^13^C signature in soil and is therefore often applied over one or even several growing seasons for a strong signal and combined with fractionation procedures. Dried and ground root and soil samples are then analysed for their C isotope ratios by high‐resolution isotope ratio mass spectrometry. Net C inputs by roots are calculated as:

(Eqn 11) $$\text{Root}\;\text{derived}\;{\text{C = [C}}_{\text{soil}}*(\delta^{13}C_{\text{soil},t1}‐\delta^{13}C_{\text{soil},t0})]/(\delta^{13}C_{\text{root}}‐\delta^{13}C_{\text{soil},t0})$$

where *C_soil_* is the C concentration in soil, δ*^13^C_soil_*_,_ *_t1_* is the ^13^C signature of soil at the end of the experiment, δ*^13^C_soil_*_,_ *_t0_* is the ^13^C signature of soil at the beginning of the experiment, and δ*^13^C_root_* is the ^13^C signature of roots (Phillips *et al*., 2012). Finally, it is also possible to couple natural abundance and ^13^C pulse labelling for specific research questions (Werth & Kuzyakov, 2006).

#### b. Future research directions

While it is increasingly recognised that root exudation encompasses spatially and temporally complex fluxes that depend on the physiological and ontogenetic status of the plant as well as on its environment, the ecological context‐dependence and the importance of the chemical composition of root exudates is often oversimplified and remains understudied. For exudates of forest trees, it has recently been shown that their composition depends on the mycorrhizal association types and that it is decisive for rhizosphere soil functions (Liese *et al*., 2018). Such improvements of root exudate chemical analyses that move from targeted analyses of specific compound groups to root exudate fingerprinting (cf van Dam & Bouwmeester, 2016) open new perspectives towards mechanistic understanding of the role of root exudates in plant and soil functions. Future research should further aim at analysing the significance of the quantity and quality of root exudates in natural (complex) plant–soil systems.

Sensitive chemical and noninvasive imaging techniques allow evaluating the biological activity of specific exuded compounds and their mobility and persistence in the rhizosphere. Recently developed methods in the visualisation of rhizosphere processes via isotope imaging have great potential in identifying soil processes that occur at a biologically relevant scale. Examples are pulse labelling in combination with nanoscale secondary ion mass spectrometry (NanoSIMS), which allows tracing the partitioning and fate of stable isotopes, as well as the resource competition between plants and different microbial species (e.g. Keiluweit *et al*., 2015b). Rhizosphere autoradiography by ^14^C phosphor imaging can be used for visualising the allocation of photoassimilates to roots and rhizosphere (Pausch & Kuzyakov, 2011). However, isotope imaging often requires embedding intact soil samples in resin, which is technically demanding for field samples, and is currently limited by the number of imaging instruments available worldwide (cf Oburger & Schmidt, 2016).

## 18 Physiology of resource uptake

Nutrients and water are major soil resources that can limit plant growth (Craine *et al*., 2012; Fisher *et al*., 2012); therefore, a thorough understanding of the processes involved in nutrient and water uptake by plants is essential. The acquisition of inorganic or organic nutrients by roots from the soil (i.e. the process of nutrient uptake) on the one hand depends on the nutrient availability at the absorbing surface of the (mycorrhizal) root and, other the other hand, on the capability and physiological efficiency of the uptake systems of the roots (cf Gessler *et al*., 2005). Nutrient availability comprises the chemical properties of nutrients, their solubility and sorption to the soil matrix, but also the nutrient re‐supply by microorganisms. It also strongly depends on the root foraging capacity and on the roots’ capacity to mobilise sorbed nutrients, involving the release of carboxylates (e.g. Lambers *et al*., 2018). These aspects are strongly influenced by species interactions (plant–plant, plant–microbial, e.g. Simon *et al*., 2017) as well as by abiotic factors (e.g. drought, waterlogging, Kreuzwieser & Gessler, 2010). By contrast, the environmental control over the physiological properties of root uptake systems is less understood. Abiotic factors (temperature, drought) are assumed to modify most strongly the root uptake capacity (Bassirirad, 2000; Gessler *et al*., 2005), but the effects of biotic interactions also need to be considered (Simon *et al*., 2013). In addition, for specific nutrients, expression of genes encoding nutrient transporters, and therefore uptake capacity, is modified by nutrient availability, for instance for nitrate (Santi *et al*., 2003). In contrast with the uptake of mineral nutrients, water uptake is not an active process, because it is driven by the water potential difference between the soil and the atmosphere. The plant, however, controls the conductance of its hydraulic system in the short term (stomatal conductance, aquaporin expression) and in the longer term (xylem vessel hydraulic properties) (Jackson *et al*., 2000). The ability of the root system to explore the soil for water resources, the hydraulic properties of the plant, together with atmospheric water demand therefore determine the water use of a given plant.

Information on soil resource uptake is not only relevant from a plant‐nutrition or water‐use perspective, but also to understand plant resilience to changing environmental conditions, as plants might be able to adjust their nutrient or water demands through acclimation or phenotypic plasticity (Jump & Peñuelas, 2005; Nicotra *et al*., 2010; Hoffmann & Sgrò, 2011). In addition, knowing resource uptake also helps to understand fundamental principles of species interactions and species coexistence: classic resource‐based niche theory predicts some sort of niche differentiation to reduce competitive interactions and to allow coexistence. As all plants rely on a rather small number of basic resources (light, CO_2_, O_2_, water, nutrients), niche differentiation must occur on rather subtle spatial, temporal or chemical differences related to resource acquisition. For example, plant species may differ in the depth or horizontal extent of greatest root activity, in the timing of resource uptake, or in preferences for different chemical forms of resources (e.g. nitrate vs ammonium vs organic nitrogen, or several different organic phosphorus compounds) (McKane *et al*., 2002; Pornon *et al*., 2007; Turner, 2008; Ashton *et al*., 2010). Such niche differentiation is also a prerequisite for complementarity in resource uptake, which has been postulated as one major mechanism underpinning the often observed positive biodiversity–productivity relationship (Loreau *et al*., 2001; Li *et al*., 2014; Tilman *et al*., 2014). Therefore, studies quantifying resource use complementarity in plant communities of different diversity levels have been increasingly performed during the last years, all relying on measurements of soil resource uptake (Kahmen *et al*., 2006; von Felten *et al*., 2009; Jesch *et al*., 2018).

The quantification of soil resource uptake by plants is not trivial. The two following points make soil resource uptake extremely dynamic in space and time which complicates any measurements of soil resource uptake:

1. It is not necessarily the presence and distribution of roots that determine water and nutrient uptake from given soil depths, but the root activity might differ among the soil horizons and is not constant in time (Gessler *et al*., 2002; Kulmatiski & Beard, 2013; Volkmann *et al*., 2016a).
2. In addition, temporal and spatial variability in root activity might differ among species and functional types (Volkmann *et al*., 2016a).

Therefore, root resource uptake data and data on root distribution and architecture are two complementary data sets with different information (e.g. Freschet *et al*., 2018). Furthermore, plant concentrations of nutrients do not necessarily reflect uptake rates due to, for example leaf turnover and other nutrient losses (cf Gessler *et al*., 1998). Uptake of different amounts or forms of nutrients cannot be derived from measurements of biomass nutrient concentrations alone. Finally, roots are difficult to identify to species or individual level in mixtures (Mommer *et al*., 2011); therefore, relating measurements of above‐ground properties to uptake of soil resources of specific roots or soil depths is not straightforward.

**Fig. 22:**
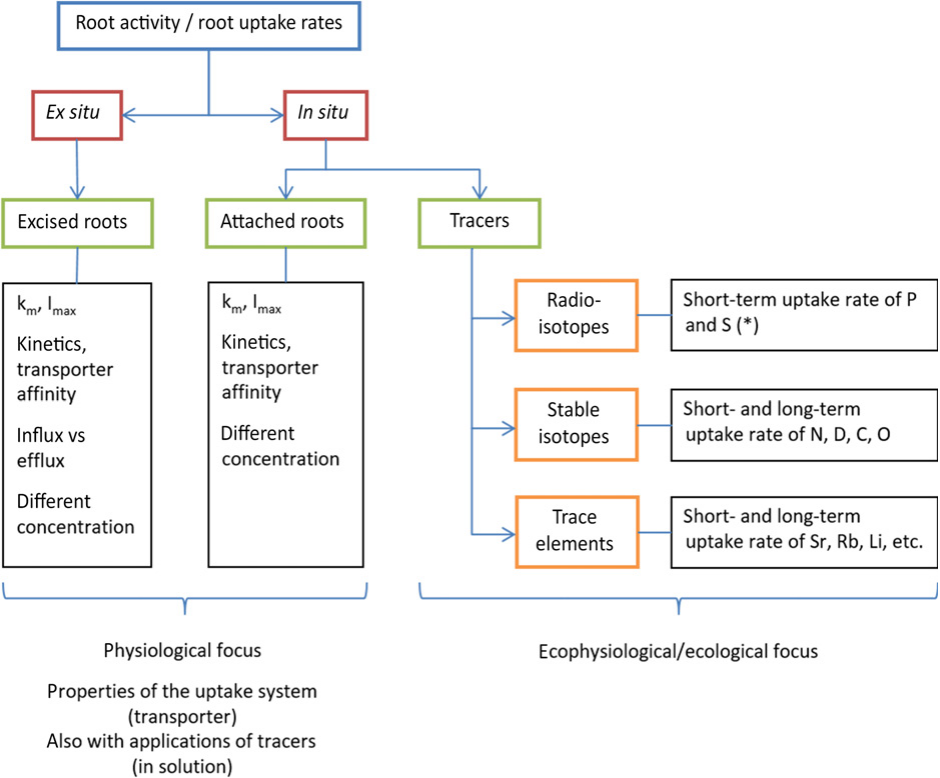
Different methodological approaches are used to quantify soil resource uptake, as a physiological root trait. Roots can be excised from the plants and incubated *ex situ* or still attached to the plant in nutrient solutions *in situ* to characterise the properties of the uptake system. Tracer addition to the soil allows estimates of nutrient uptake of plants within the soil system. *The radioisotope method is not covered in this handbook.

The quantification of soil resource uptake as a physiological trait with high plasticity comprises a challenge and several methods and procedures have been used. These often face the trade‐off between short‐term vs long‐term temporal dynamics of resource uptake (Table 4). In general, the methods can be divided into *ex situ* and *in situ* methods, and into those with a physiological focus or an ecological focus (Fig. 22). By ‘physiological focus’ we mean measurements of uptake rates under artificial and mostly stable conditions (e.g. roots suspended into a nutrient solution), as an assessment of the physiological characteristics of the root/mycorrhizal uptake system. By contrast, the ‘ecological focus’ refers to the assessment of the ‘real’ nutrient or water uptake under field conditions, which integrates various parameters, including nutrient availability (immobilisation, ion movement, leaching) or water availability (precipitation events, soil texture, season) and plant uptake capacity.

**Table 4:** Overview of the different methods to quantify soil nutrient and water uptake by plant roots.

Root activity traits	System	Focus	Method	Assessment of niche differentiation	Short description	Reference examples
Nutrient uptake traits						
Nutrient uptake kinetics (*k*_m_, *I*_max_)	*Ex situ* or *in situ* (but excavated roots)	Physiological	Tracer or measurement of nutrient depletion in solution	Yes but only indirectly	Concentration‐dependent nutrient uptake of excavated roots from an incubation solution. Uptake rate is based on fine‐root biomass, length or surface area. Natural environment of the roots (soil as a system of solid, aqueous and gaseous spaces) is changed (aqueous solution) during the assessment of uptake, mycorrhizal hyphae are destroyed	Kreuzwieser *et al.* (2000); Gessler *et al.* (2005); Lucash *et al.* (2007)
Short‐term net uptake rates	*In situ*	Ecophysiological/ecological	Tracer in natural soil	Yes	Relative tracer enrichment in plant biomass (comparison among species or functional groups). Tracer uptake from the natural soil. The uptake rate is based on plant biomass, root length or ground surface area. Processes in the soil (i.e. tracer adsorption to soil particles, tracer diffusion) and the root (transport rates) are integrated, but therefore cannot be disentangled	McKane *et al.* (2002); Kahmen *et al.* (2006); Ashton *et al.* (2010); Jesch *et al.* (2018)
Long‐term net uptake rates	*In situ*	Ecophysiological/ecological	Tracer in natural soil	Yes	Relative tracer enrichment in plant biomass (comparison among species or functional groups). Tracer uptake from the natural soil. Processes in the soil (i.e. tracer adsorption to soil particles, tracer diffusion) and the root (transport rates) are integrated and therefore cannot be disentangled	Chapin & Van Cleve (2000); Fotelli *et al.* (2004)
Water‐uptake traits						
Short‐term uptake	*In situ*	Ecophysiological/ecological	Tracer in natural soil	Yes	Relative tracer enrichment in plant biomass (comparison between species or functional groups). Tracer uptake from the natural soil. The uptake rate is based on plant biomass or ground surface area. Allows the distinction of water uptake from different soil layers (when multiple tracers are applied) but uptake rates cannot be easily determined	Grossiord *et al.* (2014); Bachmann *et al.* (2015)

While the methods for soil resource uptake described in this chapter cover the spatial scale from single roots/plants to the ecosystem level, larger scales such as the landscape scale including nutrient‐based interactions between different ecosystems within a landscape are more difficult to assess. Our lack of knowledge on that scale might call for new generations of tracer experiments where, for example the manure of cows is isotopically labelled (e.g. with ^15^N) via the fodder and the distribution of this label is tracked across landscape elements. The application of isotopic landscapes (isoscapes) allows tracking such distribution of, for example nitrogen from fertilised agricultural fields to surrounding ecosystems (e.g. Nitzsche *et al*., 2016).

There are many other traits that will add to the information on the traits described in this chapter. Amongst them are traits related to below‐ground allocation, root morphology and anatomy, the architecture of the rooting system and fine‐root distribution within the soil. While, as described above, the presence and distribution of roots does not necessarily correspond with root activity, a set of complementary root traits (e.g. Freschet *et al*., 2018) is necessary to provide a full picture of resource acquisition and its drivers.

Another set of traits that is highly complementary are physiological or transcriptome‐related traits. For water uptake, measurements of root and shoot hydraulic conductance as well as of aquaporin activity (e.g. Johnson *et al*., 2014) are highly informative. The expression of particular nutrient transporters, enzymes related to nutrient assimilation, but also of aquaporins will provide the molecular basis for the soil resource uptake traits.

### 1. Nutrient uptake kinetics (*k*_m_, *I*_max_)

Nutrient uptake kinetics describe the concentration‐dependent net uptake rates of a given nutrient by the (mycorrhizal) root. Along a concentration range expected in natural ecosystems, active uptake of the most important elements will be mediated by high‐affinity transporters. In this context, concentration‐dependent net ion uptake rates follow usually a hyperbolic function that can be characterised by two traits:

*Root maximum net ion uptake rate*, is the amount of ion accumulated per unit root biomass and time under conditions of nonlimiting nutrient concentration (typical units: µmol g^−1^ s^−1^) (frequent abbreviation: *I*_max_ or V_max_). It can also be expressed per unit root length or root surface area.

*Root Michaelis–Menten constant*, is the nutrient concentration where 50% of the maximum net ion uptake rate is observed (typical units: mmol l^−1^ or mM) (frequent abbreviation: *k*_m_).

The Michaelis–Menten constant (*k*_m_) is a measure for the affinity of a transport system for its substrate; the lower the *k*_m_ the faster nutrients can be taken up at low availability, whereas the maximum net ion uptake rate (*I*_max_) represents a potential rate at nonlimiting substrate availability that might however, not be fully expressed under *in situ* conditions. The traits *k*_m_ and *I*_max_ therefore describe fundamental properties of a transporter system as modified by abiotic (e.g. temperature) and biotic (e.g. mycorrhization) environmental factors that can also be closely linked to gene expression (Li *et al*., 2016). These traits are particularly meaningful to measure on absorptive roots or segments of roots involved in nutrient uptake (e.g. first‐order roots). They are most relevant for the acquisition of mobile soil ions (e.g. nitrate), but largely irrelevant for less mobile ions (e.g. phosphate) (Lambers & Plaxton, 2015).

In principle, the assessment of nutrient uptake kinetics allows for studying and comparing environmental impacts on the plant’s physiological performance (i.e. the transporter potential) of nutrient acquisition without needing to consider soil or microbial related constraints in nutrient supply. While widely applied in basic plant physiological studies (Kronzucker & Siddiqi, 1996; Kreuzwieser *et al*., 2000; Glass *et al*., 2002) the potential of this technique has not been fully exploited in more ecology‐related research. Gessler *et al*. (2005), for example, showed that long‐term exposure of adult beech trees to warm and dry environmental conditions led to a reduction of the maximum net nitrate uptake rate. With this assessment, the authors showed that the sensitivity of the nitrogen balance of beech towards reduced soil water availability was mainly a consequence of root physiological properties, rather than of limited microbial re‐supply of inorganic N or of changed patterns of inorganic N partitioning between soil bacteria and roots.

Roots excavated from the soil are incubated in nutrient solutions and net ion uptake rates can be either determined by accumulation of tracers (e.g. ^15^N) in the plant (e.g. Gessler *et al*., 2005) or by depletion of the nutrients in the external solution (without any need for tracers) the root is incubated in (Gessler *et al*., 1998).

While the assessment of nutrient uptake from a solution allows direct characterisation of the uptake properties of the root transporters (and is therefore not affected by soil‐related processes), there are some major points that might affect the measured uptake rates to be taken into account: the roots are removed from their natural soil substrate (which is a matrix that consists of solid, liquid and gaseous parts) into a solution, and therefore the conditions the root is exposed to are far from natural. Moreover, when excavating the roots, parts of the extramatrical mycelium of ectomycorrhizal roots is destroyed. In addition, it might be difficult to distinguish roots of plant species in mixtures. These are unavoidable shortcomings of the method, but assessments of the kinetic parameters of nutrient uptake of field‐grown plants provides important complementary information to the short‐term and long‐term uptake rates from the soil as described below. There might be an exception for phosphorus as the diffusion of inorganic phosphate in soil is the key limiting factor for uptake. Changes in kinetic parameters of the P‐uptake system in the roots generally have only small effects on the overall uptake capacity of plants (Lambers *et al*., 2006; Lambers & Plaxton, 2015).

#### a. Sampling recommendations

For the assessment of the root nutrient uptake, a two‐step sampling procedure is applied. In a first step, fine roots with root tips are excavated from the soil, washed carefully with demineralised water or with a solution that equals the solutions in the soil the roots are growing in and incubated into a nutrient solution. In a second step, either the plant material (if accumulation in the plant is assessed) or aliquots of the incubation solution are sampled (if nutrient depletion is determined).

*First sampling step*. Root tips are excavated carefully by hand or with a spatula from the soil layer of interest with extreme care, to minimise the damage done to the roots and root hairs. They are subsequently rinsed with double‐demineralised water to remove adhering soil particles and carefully dried at the surface with paper towels. There are now two options for incubation (see Lucash *et al*., 2007): (1) The cleaned roots are kept attached to the plant and incubated *in situ*; or (2) the roots are excised for *ex situ* incubation. Option (1) keeps source–sink relationships intact, and might be preferred if net uptake rates are targeted. By contrast, while (2) might be used for particular purposes such as the determination of gross influx and efflux (see e.g. Kreuzwieser *et al*., 2000), the excision might introduce artefacts (e.g. carbohydrate depletion) that affect nutrient uptake (Lucash *et al*., 2007). For (1), assessments with stable isotopes and element analogues are possible. With option (2) the uptake of elements for which no stable isotope tracer or element analogue (see Gockele *et al*., 2014) but radioisotopes are available (e.g. phosphorus) can be assessed if suitable laboratory facilities are close to the field site. Further incubation and sampling are only described for option (1).

*Incubation.* For incubation, artificial soil solutions that mimic the element composition and the pH of the soil solution under study is generally preferred. The total volume of solution depends on the amount of root tips to be incubated. An example of the composition of an artificial soil solution for beech and spruce ecosystems is given by Gessler *et al*. (1998). The target element – that is the element for which the concentration‐dependent uptake rates is assessed – is added in different concentrations either in a labelled form (e.g. ^15^N) for the tracer accumulation method, or unlabelled for the depletion method. For the uptake of organic compounds (such as amino acids) double‐labelled compounds (^13^C and ^15^N) are recommended to test if the whole molecule (and not only, for example, the split ammonium for amino acid incubations) is taken up (Warren, 2009).

*Second sampling step.* For the assessment of the nutrient depletion in the solution, aliquots are taken at different times (e.g. 0.5, 1, 1.5, 2 and 4 h) (see Gessler *et al*., 1998). Moreover, the root tips incubated are sampled to standardise the measurement per root biomass, length or surface area. For the assessment of tracer accumulation, the root tips incubated in the solution plus a part of the root outside the solution to where the tracer might have been transported (Gessler *et al*., 2002) are sampled. To avoid the loss of labelled element by acropetal tracer transport, short incubation times (e.g. 2 h) are preferred. The root tips and the transport segment are washed with artificial soil solution without tracer (Gessler *et al*., 2005).

#### b. Storage and processing

*Depletion method.* Aliquots of the incubation solution should be immediately frozen and stored at −20°C until analysis. Roots for determination of biomass or surface area can be stored in the refrigerator if the subsequent measurements are performed soon. If uptake rates are based on root dry weight (DW), root samples can also be dried at 60°C immediately.

*Tracer accumulation.* When assessing tracer accumulation, the immersed roots and the portion of roots attached to it up to several centimetres away from the immersed root can be stored separately for short‐term periods (1 d) at 4°C. This allows a straightforward assessment of the root length or surface area which is more difficult in frozen material. After measurements, the samples are dried at 60°C. Alternatively, both samples are frozen in liquid nitrogen in the field. If uptake rates are based on the FW of the roots, samples are weighed frozen and thereafter dried at 60°C.

#### c. Measurement procedure

*Depletion method.* Concentrations of ions in the incubation solutions are determined by ion‐exchange chromatography with suitable cation and anion exchange columns, or by photometric assays. The net uptake rates are calculated by linear regression analysis of the decrease in the target ion concentration vs time and based on the FW, DW or surface area of the parts of the root tips in the incubation solution. During the incubation, uptake of water by the roots is measured by weighing the solution before and after incubation, and the sampled solution aliquots and the root water uptake and sampled aliquots are taken into account when calculating the ion uptake rates.

*Tracer accumulation.* The concentrations of cation analogues such as Li, Cs, Rb and Sr in the roots can be determined with an inductively coupled plasma–optical emission spectrometer (ICP–OES) or mass spectrometer (ICP‐MS) as described in detail by Hoekstra *et al*. (2014). Stable isotope tracers in plant material are determined by isotope ratio mass spectrometry (IRMS) as described by Gessler *et al*. (2005). In both cases, control roots without tracer incubation serve to calculate the tracer accumulation during incubation. Tracer accumulation is based on the immersed roots and a few centimetres of section of roots outside the solution. Total accumulation is subsequently expressed based on the incubation time as well as on the root FW, DW, length or surface area of the parts of the roots submersed in the incubation solution.

*Calculation of I*_max_ *and K_m_*. *I*_max_ and *k*_m_ in the Michaelis–Menten model can – *sensu strictu* – only be applied to unidirectional reactions (Price & Stevens, 1989), but nutrient net uptake is a process that consists of both influx and efflux. Still, in a first approximation, net ion uptake follows such uptake kinetics and allows the fit of the Michaelis–Menten model (Kronzucker *et al*., 1995). The model can be either fitted and the parameters *I*_max_ and *k*_m_ derived via linear transformation (e.g. according to Lineweaver & Burk, 1934) and subsequent linear fitting or via the nonlinear curve fit functions available in most statistical or graphical software packages.

#### d. Future research directions

Uptake kinetics might be strongly depending on rhizosphere microbial symbionts (Kreuzwieser *et al*., 2000). In this context, methods allowing *in situ* assessments of the mycorrhizal and bacterial community together with nutrient uptake measurements are critically needed to disentangle biotic effects on the root resource acquisition potential.

### 2. Short‐term and long‐term net uptake rates

*Net uptake rate* of nutrients or water is the amount of a specific element accumulated within a plant individual per unit dry mass and time (typical units: µg g^−1^ h^−1^ or µmol g^−1^ h^−1^).

The period of accumulation can range from one or a few hours to one or a few days (short‐term net uptake rates) or from several days to months (long‐term uptake rates). Short‐term net resource uptake rate assumes no or very little element loss due to transpiration, leaching, above‐ground or below‐ground herbivory or senescence, whereas long‐term net uptake rate implies that some of the accumulated elements are disappearing from the plant during the incubation time. Long‐term uptake also integrates over changing environmental conditions, such as daily or seasonal changes in weather, or over changing biotic interactions, such as timely limited occurrence of herbivores. It will, therefore, be the preferred measure if the reaction of plants´ resource uptake to phenological changes and over longer periods is of interest. By contrast, short‐term net uptake rates might be preferable to compare different plant species or different environmental conditions, for instance. Moreover, if the direct dependencies of resource uptake, for example on temperature (e.g. Gessler *et al*., 1998), are needed as inputs for models, short‐term rates need to be determined.

Short‐term and long‐term net uptake rates can be quantified by applying either isotopic or nonisotopic tracer elements to the soil, and measuring their accumulation in plant organs, for example roots, stems or leaves (Hoekstra *et al*., 2014). The accumulation of the tracer is driven by absorptive root uptake and subsequent allocation within the plant. Net uptake rates can be expressed per above‐ground or total plant biomass, in cases of a plant or community perspective. It can also be expressed per mass or length of absorptive roots, if net uptake rates of root systems are the focus. For herbaceous plants, it may also be useful to express it per total root mass or length. If expressed per unit root length, it provides a measure of the efficiency of plant net uptake, irrespective of its root mass fraction and root morphology.

Plant species differ strongly in their spatial, temporal and chemical patterns of nutrient and water uptake rates by fine roots, allowing different species to coexist (Fitter, 1986; McKane *et al*., 2002). For example, by applying multiple tracers simultaneously and calculating net uptake rates, multidimensionality of below‐ground resource niches and resource use complementarity was determined (von Felten *et al*., 2009; Jesch *et al*., 2018). In addition, resource uptake also changes with abiotic and biotic conditions; for example along environmental gradients such as altitude (Soethe *et al*., 2006), with resource stress (Freschet *et al*., 2018), or under intraspecific vs interspecific competition (Lehmann *et al*., 2001); uptake rates are highly plastic and dynamic. The measurement of short‐term or long‐term net uptake rates helps to identify and quantify these patterns, and therefore enables to understand the drivers of root activity (Hoekstra *et al*., 2014).

As uptake of many nutrients is a regulated process, adjusted to the plant’s demands and therefore its carbon assimilation (Gessler *et al*., 2004), it is likely to relate positively to C assimilation rate in the long term. Additionally, while there is some evidence for positive correlations of long‐term net N uptake rate with root nitrogen concentration, SRL and relative growth rate (Reich *et al*., 1998), and between P uptake with root mass, root surface phosphatase activity and total root length (Fujita *et al*., 2010) the generality of such trait co‐variations between resource uptake rates and other traits is still unclear, because there are few reports in the literature. This might also be due to the fact that uptake rates are not so simple to measure (see below), and therefore are rather rare in comparison to other trait data: for example, in the global TRY database there are less than 300 observations of root N uptake, less than 80 for P uptake, and less than 50 for cation uptake (Kattge *et al*., 2011), but more than 80 000 observations for leaf N, more than 56 500 for leaf P, and more than 11 500 for leaf K content per dry mass.

#### a. Sampling, storage and measurement procedure

The quantification of short‐term net uptake rates of soil resources by root systems is based on the detection of tracers in plant biomass which had been added to the soil. A detailed review of this method can be found in Hoekstra *et al*. (2014). Tracers are substances, which naturally occur in very low quantities and are chemically equivalent to other nutrients that are studied. Three classes of tracers are used: (1) radioisotopes (e.g. ^32^P, ^33^P, ^35^S), (2) stable isotopes (e.g. ^15^N, ^34^S, ^18^O, D), and (3) trace elements (e.g. Sr^2+^, Li^+^, Rb^+^, Cs^+^), with the latter two being mostly used in recent years. The reliability of experiments to study resource‐uptake rates depends on some assumptions concerning: (1) mobility of the tracers in the soil matrix, (2) tracer‐application density, (3) tracer‐incubation time, (4) tracer‐application rates, and (5) the comparability of different tracers in multiresource studies. These assumptions cannot be discussed in detail here, and we refer to the above‐mentioned review (Hoekstra *et al*., 2014).

**Fig. 23:**
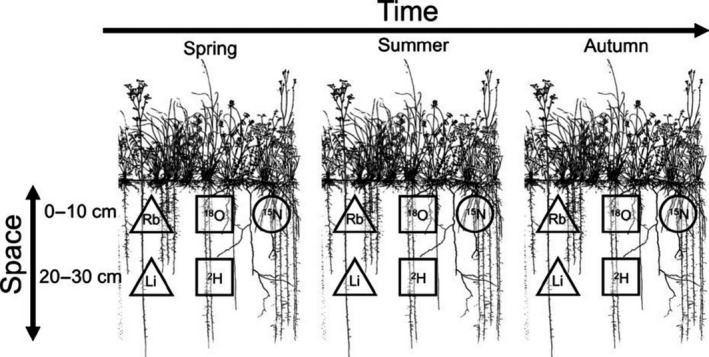
Experimental design adopting a multiple tracer study to quantify resource uptake rates along spatial (soil depth), temporal (season) and chemical (water, nitrogen, cations) niche axes (Jesch *et al*., 2018). The spatial and chemical axes are quantified in the same subplot, whereas different subplots are used to assess temporal niche differentiation. Reproduced with permission from the British Ecological Society.

In general, tracer studies to quantify resource‐uptake rates can be based on single resources, for example if the aim is to study the uptake of nitrogen under different environmental conditions (Fotelli *et al*., 2004) or to study uptake from different soil depths or at different times or of different chemical forms, using distinct plots for each depth or time step or chemical form (von Felten *et al*., 2009). In addition, multiple tracer designs can be used to simultaneously test for such differences in the same plot, avoiding environmental variability among plots, which could add noise to the uptake signal. One example of such a multiple tracer experiment is shown in Fig. 23, where resource‐uptake rates along spatial, temporal and chemical niche axes was quantified.

**Fig. 24:**
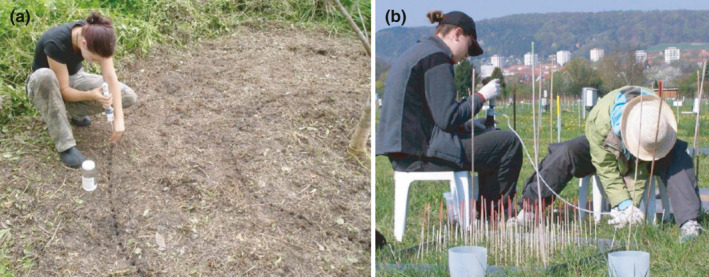
Two examples of tracer application by injection into the soil around target plants (a) or within a specific area (b). Images courtesy of M. Scherer‐Lorenzen (a) and A. Jesch (b).

Generally, the tracers are added to the soil in water solutions, either by spraying an area around target plants (e.g. Grossiord *et al*., 2014) or over larger areas (e.g. Buchmann *et al*., 1995), assuming a certain horizontal root distribution, or by injecting it into the soil at a defined depth through predrilled holes (e.g. Ashton *et al*., 2010; Jesch *et al*., 2018) (Fig. 24). For the latter approach, either rings of injection holes around target plants, or regular grids within a certain area are used to ensure an even distribution of the tracer within the soil (Fig. 24). The amount of the tracer used should be small enough to not disturb the internal water or nutrient budget of the study system, but large enough to be detectable in plant biomass after some time. This decision needs either calculation of nutrient budgets based on available data on nutrient pools in soil and plants (Buchmann *et al*., 1996), or pilot experiments to test the ideal tracer concentrations for the specific study system. For isotopic tracers, substances with a highly enriched isotope can be used (e.g. 98% enriched ^15^NH_4_^+^ or ^15^NO_3_^–^) to keep the total amount of the nutrient low. For nonisotopic trace elements, care must be taken to limit the concentrations to rather low levels, because these elements are often applied as chloride salts, involving the risk to run into chloride toxicity. In the calculation of the amount of tracer needed, several assumptions or previous knowledge must be included, such as: (1) natural abundance of the tracer in soil and in the plant material, (2) target tracer concentration in the plant material to be harvested, which depends on analytical precision (e.g. 100‰ δ^15^N), (3) biomass of the plants to be harvested, in which the tracer will be diluted, (4) soil volume soaked by the tracer solution, (5) ratio of tracer taken up by plants, immobilised by soil organisms or in the soil matrix (e.g. with high clay content), or leached to lower soil layers with less roots (e.g. with high sand content); here, a 25% uptake by plants seems to be a conservative assumption, (6) ratio of tracer allocated to target plant organ (either coarse roots, stem or leaves), and (7) the number of applications or treatments. A very good and detailed example about these calculations can be found in Kahmen *et al*. (2006). According to the large number of such assumptions to be made, typical ranges of tracer volumes or concentrations applied differ largely depending on the study system. For example, short‐term net uptake of ^15^N as ammonium or nitrate has successfully been measured in temperate grasslands with concentrations of 10 mM K^15^NO_3_ or ^15^NH_4_Cl (enrichment of 98% ^15^N), resulting in approx. 55 mg added ^15^N m^–^² in a field study (Kahmen *et al*., 2006). The amount of nonisotopic cation tracers ranged between 0.2 and 9.5 g m^–^² in the study by Hoekstra *et al*. (2014). Control plots or plants should receive the same amount of water or nutrient solution, but without the tracer element added and with the same total nutrient concentration. For example, while treatment plants/plots would receive a ^15^N‐enriched nitrate or ammonium solution, the control plants/plots would receive a nonlabelled nitrate or ammonium solution of the same N concentration.

The period between tracer application and sampling of plant organs to detect the accumulation of the tracer (incubation time) will depend on the system and plant species studied. For example, herbaceous plants grown in well watered pots will show tracer accumulation in above‐ground organs within less than an hour while large trees under dry soil conditions will need few days. Studies with herbaceous plants showed that an increase of incubation time from 24 h to 48 h increased the concentration of tracers in plant material, and that this was independent of plant species or tracers applied (Hoekstra *et al*., 2014). Biomass of the target plant parts (either roots, root crowns or leaves, twigs or stems) is harvested, dried at 60°C and the concentrations of the tracer elements are measured. Harvests at several time intervals on replicate individuals or at several locations on the same plant individual will allow for a description of the temporal uptake patterns, for example whether it follows a linear, exponential or asymptotic relationship. The plant parts being sampled to detect the accumulation of tracer elements depend on the research question, and roots, stems or leaves are usually sampled, with a clear bias towards above‐ground biomass, which can be explained mainly by practical considerations. If roots are sampled, rinsing with water is important to reduce the risk of contamination with tracer present in the rhizosphere. Sampling above‐ground parts only bears the risk that root activity might be underestimated, if the elements are not further allocated from the root system to other plant parts. For water uptake quantified by ^18^O‐labelled or D‐labelled water, either roots, stem xylem water (woody plants) or water extracted from the root crown, that is the part between the above‐ground and below‐ground organs of herbaceous plants, are usually taken, because they show the least variability in the isotopic signal as post‐uptake fractionation in transpiring organs is avoided (Barnard *et al*., 2006). For studies quantifying net uptake rates of nutrients, the period with the greatest nutrient demand might be chosen, that is during fastest leaf production in herbaceous plants, or a few weeks after first leaf emergence in trees (Albaugh *et al*., 2012).

Xylem water or sampled organs for water isotope analysis in roots or root crowns must be frozen in liquid nitrogen or at −20°C for subsequent cryogenic water extraction. During extraction, the plant sample is heated (80°C) under vacuum, and evaporated water is trapped via freezing in a liquid nitrogen cold trap (Ehleringer *et al*., 2000). After defrosting, ^18^O is analysed on the liquid water sample. Samples for nutrient analyses should be transported in cooling boxes from the field to the laboratory, and then oven dried (e.g. at 60°C until constant weight), milled and analysed for tracer element concentrations. Standard chemical analyses are then used to quantify tracer concentrations in the plants (e.g. IRMS; atom absorption spectrometry (AAS); ICP–OES).

##### Calculation of net uptake rates

The calculation of tracer net uptake as a measure of root activity is a critical part, and several slightly different approaches can be found in the published literature, ranging from comparison of tracer concentrations in biomass of different species or points in time (e.g. Fitter, 1986), to rather sophisticated calculations involving nutrient pool sizes in soil and plants, as well as mineralisation rates or tracer diffusion radius in the soil (Kahmen *et al*., 2006). Most comparisons involve the calculation of tracer excess in treated plants compared with nontreated plants, or background concentration of tracers before labelling, by subtracting the tracer concentration of control (nonlabelled, or before labelling) plants to the tracer concentration of labelled plants (Hoekstra *et al*., 2014). However, to account for differences in plant standing biomass, and to calculate total resource uptake of a whole plant (or plant community), tracer concentrations of each part sampled are preferably multiplied by the biomass of these parts, and summed up (and then expressed per unit ground area in cases of a plant community) to derive tracer contents (g or g m^−2^). The total amount of tracer taken up can then be further expressed per unit biomass (e.g. per plant or root dry mass, μg ^15^N g^−1^), or for roots also per root length (μg ^15^N m^−1^). For nonisotopic tracers, units are given as content per dry mass (g g^−1^ or mol g^−1^). As rates refer to uptake during a specific period, these values should be divided by the respective time (e.g. μg ^15^N g^−1^ h^−1^). The use of different units critically depends on the research question (e.g. a focus on uptake from shallow vs deep soil layers, or comparison of uptake rates of co‐existing species with different abundances), as discussed in detail in Hoekstra *et al*. (2014).

If the aim is to study spatial, temporal or chemical niche differentiation or resource complementarity among co‐existing plants, then the net uptake rates from different depths, at different times or of different chemical elements should be compared among species. This could include calculations of similarity between resource‐uptake rates and available resource pools (McKane *et al*., 2002), or resource niche breadth or overlap (von Felten *et al*., 2009; Jesch *et al*., 2018).

#### b. Future research directions

Short‐term net uptake rates of soil resources measured *in situ* are not easy to assess in a highly standardised way, because, depending on the research question and study system, different assumptions have to be made (Hoekstra *et al*., 2014). A major issue in this respect is the rather low comparability of different chemical tracers used in multiple tracer studies or across studies, because of the different chemical properties influencing mobility within the soil, root uptake physiology and allocation within plants. Therefore, site‐specific correction factors should be developed that account for plant parts (e.g. root vs stem vs leaf) and plant species (Gockele *et al*., 2014).

There are additional partially complementary methods that allow study of water and nutrient uptake, but a main constraint is the difficulty to use them in ecological experiments, especially under field settings. One example is the assessment of ammonium or nitrate uptake applying ion‐selective microelectrodes (e.g. Henriksen *et al*., 1992). This technique also allows the localisation of the nutrient uptake along the root axis, but the uptake measurements require laboratory settings. Spatial mapping of nutrient uptake can also be achieved via radioactive tracers (e.g. ^32^P) and autoradiography imaging (e.g. Rubio *et al*., 2004), but the application of radioactivity prevents field studies.

Only recently, nondestructive real‐time measurements of water uptake of the vegetation have been applied, combining the detection of the isotopic signal of water isotopologues, that is water molecules of different hydrogen and/or oxygen isotopic composition, in different soil layers and in plant‐transpired or xylem‐transported water, together with water flux measurements (Volkmann *et al*., 2016a,b). With the new generation of field‐deployable water isotope laser spectrometers, this technique provides the possibility to assess the water use of plants and ecosystems in detail, and also to understand short‐term dynamics during drought or rainfall events. Moreover, minimal invasive sampling of plants for different metabolites has been applied successfully with micro‐dialyses systems (Pretti *et al*., 2014) and soil nutrient concentration can also be determined with this technique (Inselsbacher & Näsholm, 2012). A combination of the assessment of the origin of water uptake and therefore the soil layer with root activity and total water fluxes as performed by Volkmann *et al*. (2016a) with high temporal resolution probing of soil and xylem nutrients with microdialysis would potentially allow calculation of nutrient uptake rates for different species from xylem concentration and mass flow. Moreover, assuming that water uptake activity is related to uptake of highly mobile nutrients such as nitrate, the depth of nutrient uptake (taking also into account the relationship between xylem concentration of a given nutrient and the concentration distribution in the soil) might also be studied. However, it needs to be taken into account that some nutrients (and their assimilates, for example amino acids assimilated from nitrate and ammonium) are not only unidirectionally transported in acropetal direction, but cycle through the whole plant (Gessler *et al*., 2004).

## 19 Mycorrhizal associations

Mycorrhiza, the term combining the Greek words ‘*mycor*’ (µνκησ) – fungus and ‘*rhiza*’ (ριζα) – root, is a symbiotic association between plant roots and fungi, where plants provide the fungi with photosynthetically derived carbohydrates and lipids and fungi provide plants with essential nutrients and water (Smith & Read, 2008; Treseder, 2013; van der Heijden *et al*., 2015; Luginbuehl *et al*., 2017). It is widely recognised that mycorrhizal associations play a key role in plant nutrition as well as in the functioning of terrestrial ecosystems (van der Heijden *et al*., 2015). The nature of this carbon‐for‐nutrients exchange relationship may vary from parasitism to truly mutualistic relations depending on light and nutrient availability in a particular ecosystem (Karst *et al*., 2008; Hoeksema *et al*., 2010).

Mycorrhizas strongly affect biogeochemical cycles (Veresoglou *et al*., 2012a; Soudzilovskaia *et al*., 2015b; Averill & Hawkes, 2016), soil formation and structure (Rillig & Mummey, 2006; Leifheit *et al*., 2014), and plant community composition (van der Heijden *et al*., 1998; Klironomos *et al*., 2011; Elumeeva *et al*., 2018) through several complementary mechanisms. Most of the mycorrhizal fungi build extensive mycelial networks in soils (Leake *et al*., 2004), except, for example, the fungi forming ericoid mycorrhizas and the contact‐type of the ectomycorrhizal associations (Agerer, 2001; Smith & Read, 2008). Although the carbon stocks and enzymatic activities of these networks are strongly mediated by traits of individual mycorrhizal fungal species that form the network (Koide & Malcolm, 2009; Clemmensen *et al*., 2015; Treseder & Lennon, 2015), evidence is rapidly growing that altogether mycorrhizal fungi themselves constitute an important carbon source in the soil (Leake *et al*., 2004; Clemmensen *et al*., 2013). Mycorrhizas also enhance soil aggregation through hyphal exudation, physical bounding of soil aggregates and through increasing hydrophobicity of surfaces (Rillig & Mummey, 2006; Leifheit *et al*., 2014; Rillig *et al*., 2015). Moreover, through the exchange of nutrients and carbon mycorrhizal fungi affect important aspects of plant life strategies, such as carbon allocation (Veresoglou *et al*., 2012b), growth rate (Karst *et al*., 2008; Treseder, 2013; Elumeeva *et al*., 2018), litter quality (Cornelissen *et al*., 2001; Langley & Hungate, 2003; but see Koele *et al*., 2012) and decomposition (Elumeeva *et al*., 2018). Ultimately, this results in a strong control of mycorrhizal symbiosis on community composition (van der Heijden *et al*., 1998; Klironomos *et al*., 2011; Elumeeva *et al*., 2018).

While a complete understanding of the role of mycorrhizas in functioning of terrestrial ecosystems and biogeochemical cycles requires a comprehensive analysis of both root and fungal traits, we only focus here on traits related to the root–fungi association and do not include traits more specifically measured on the mycelium. In this synthesis, we focus on the presence and functioning of mycorrhizal symbiosis by addressing sequentially what type of mycorrhizal association the plant has, how intensively plant roots are colonised by the mycorrhizal fungi, and which fungal species form the mycorrhizal partnership with the plant.

### 1. Mycorrhizal association type

*Mycorrhizal association type* is a classification of mycorrhizal association based on the identity of mycorrhizal partners, as well as morphological features of the symbiosis (categories: arbuscular mycorrhizal, ectomycorrhizal, ericoid mycorrhizal, orchid mycorrhizal, nonmycorrhizal and combinations thereof).

There are four widely recognised types of mycorrhizal association: arbuscular mycorrhizal (AM, 72% of all terrestrial plant species), ectomycorrhizal (ECM, 2.0% of plant species), ericoid mycorrhizal (ERM, 1.5% of plant species), and orchid mycorrhizal (OR, 10% of plant species) (Brundrett & Tedersoo, 2018). Arbuscular mycorrhiza is most often defined by the identity of the fungi forming the symbiosis, and presence of typical tree‐like fungal structures (arbuscules) inside the root. Ericoid and orchid mycorrhiza are most often defined by the taxonomic identity of the plant species. Ectomycorrhiza is most often defined by the morphology of the symbiosis. It is important to bear in mind that there is yet no broadly accepted definition of what a mycorrhizal type is, and which features of the symbiosis should be used to differentiate between mycorrhizal types (Brundrett & Tedersoo, 2019; Bueno *et al*., 2019). Some plant species do not form mycorrhizal associations, whereas others might be facultatively mycorrhizal. To date, facultative mycorrhization has been reported only for plant species able to form arbuscular mycorrhizas (Moora, 2014; Brundrett & Tedersoo, 2018). Among all vascular plants, only 8% are completely nonmycorrhizal (NM), while 7% have inconsistent NM–AM associations, which means that these plants are able to grow without fungal partners (Brundrett & Tedersoo, 2018). For such plant species, their mycorrhizal status can vary among plant individuals and must be assessed for each site examined in a study.

**Fig. 25:**
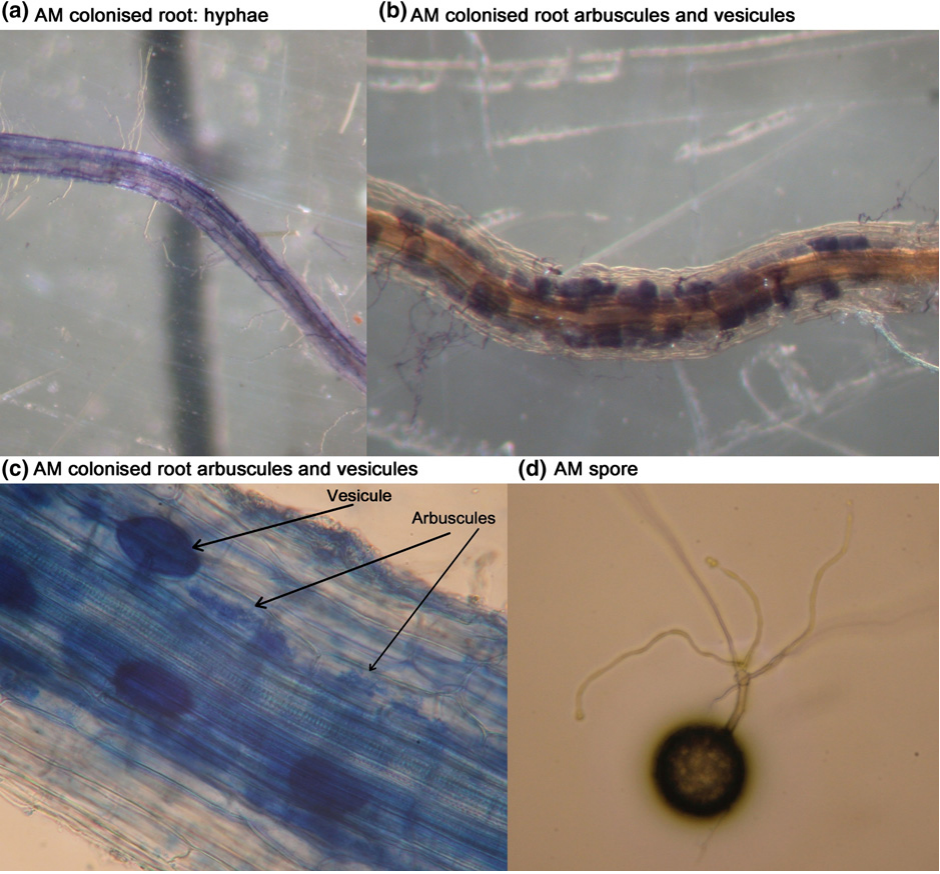
(a) Roots of *Molinia caerulea* colonised by arbuscular mycorrhizal (AM) fungi (species unknown) with visible hyphae; and (b) arbuscules and vesicules; captures with stereomicroscope (×3–5 magnification). (c) Wheat roots (*Triticum aestivum* L.) colonised by AM fungi *Rhizophagus irregularis* with visible hyphae, arbuscules and vesicules; and (d) spores and extraradical (i.e. outside of roots) mycelium of *Rhizophagus irregularis*. Note that vesicules (c) are very similar to fungal spores, but the latter can be distinguished because they are typically round and not connected to the hyphal network. Images courtesy of M. Bakker (a, b, stereomicroscope, ×3–5 magnification); and M. Rebeca Cosme (c, d, compound microscope, ×200 magnification).

Arbuscular mycorrhizal symbiosis is globally most widespread, both geographically (Read, 1991; Soudzilovskaia *et al*., 2019) and taxonomically (Brundrett, 2009; Brundrett & Tedersoo, 2018). Plant species that form AM represent the majority of the families of vascular plants (Brundrett & Tedersoo, 2018). Arbuscular mycorrhiza is widely recognised as the symbiosis formed by fungi that belong to the phylum Mucoromycota, of which the majority of fungal taxa that are able to form AM are included in the subphylum Glomeromycotina (former phylum Glomeromycota; Spatafora *et al*., 2016). Arbuscular mycorrhizal fungi penetrate into the root cells and form specific structures, called arbuscules and vesicules, within the root cells (Fig. 25). Arbuscular mycorrhizal fungi provide their host plants with phosphorus and water and enable pathogen protection. There is some evidence that AM fungi are able to provide nitrogen to their hosts as well (Hodge *et al*., 2001; Govindarajulu *et al*., 2005), but the magnitude of this supply is limited at the ecosystem level (van der Heijden *et al*., 2015).

**Fig. 26:**
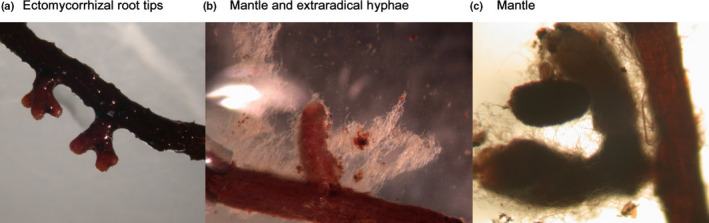
(a) Ectomycorrhizal fungi (EMF) structures on roots of *Pinus pinaster* Aiton showing ectomycorrhizal fungi (ECM) root tips; (b) mantle and extraradical hyphae; and (c) mantle. Images courtesy of C. Guérin and M. Bakker; stereomicroscope, ×3–5 magnification.

Ectomycorrhiza is the second most widespread mycorrhizal type in terms of geography (Read, 1991; Soudzilovskaia *et al*., 2019). However, taxonomically, the range of plants able to form ectomycorrhiza is limited to 2% of plant species, within few families of gymnosperms and angiosperms (Brundrett, 2009). By contrast, there are many fungal species able to form the ectomycorrhizal association, belonging to nonrelated lineages in the phyla *Ascomycota* and *Basidiomycota*. The ECM fungi form a Hartig net of hyphae surrounding the root cortex cells, and a hyphal sheath, also called mantle, covering the root tips, both features typically used to identify ECM (Fig. 26). Ectomycorrhizal fungi provide their host plants with all macronutrients and micronutrients (Smith & Read, 2008) and water.

In addition to the traditionally recognised ECM, three other types of mycorrhiza are recognised as special types of ECM. Arbutoid and Pyroloid mycorrhizas are formed by *Basidiomycetes* fungi and plants that belong, respectively, to the genera *Arbutus*, subfamily Arbutoideae and some species of the *Pyrolaceae* family of the *Ericales* order. The morphological structures of the arbutoid mycorrhizas are similar to those of other ECM: the fungi create a sheath or mantle that covers the root tips and an intercellular Hartig net, typically in the outer layer of root cells. Arbutoid and pyroloid mycorrhizal fungi forming these associations penetrate the outer cortical cells, and form coils within the cells. Such intracellular coils, along with the mantle sheath and Hartig net are the diagnostic features of arbutoid and pyroloid mycorrhizas.

The third special type of ectomycorrhiza is a monotropoid mycorrhiza. It is formed by plants that belong to the *Monotropoideae* section of the family *Ericaceae* that lost their photosynthetic capacity and live as achlorophyllous plants on the forest floor. Unlike other mycorrhizal plants these species obtain both carbon and nutrients from their fungal partners, parasitising on mycorrhizal mycelia supported by other plants (Manoharachary *et al*., 2002). This association is formed by fungal species that belong to the genera *Tricholoma*, *Russula* and *Rhizopogon*. Morphologically, this type of mycorrhiza is similar to ECM: fungi form fungal sheaths and Hartig nets on root tips. However, in addition, the fungi penetrate into the tangential walls of the host plant's root cortical cells forming fungal pegs that are enveloped by epidermal cell wall material and plasma membrane (Peterson & Massicotte, 2004). In contrast with ericoid and arbutoid mycorrhizas, monotropoid fungi forming mycorrhizal associations do not penetrate the plant cell walls (Manoharachary *et al*., 2002; Smith & Read, 2008).

Given the distinct mode of plant nutrient supply enabled by AM and ECM, these two most widespread types of mycorrhizas play contrasting roles in ecosystem functioning. According to the mycorrhizal‐associated nutrient economy (MANE) hypothesis (Phillips *et al*., 2013), vegetation dominated by AM and ECM plants are characterised by different carbon and nutrient cycles, in line with the different mycelial morphologies and enzymatic capacities of AM and ECM symbionts. As AM fungi have limited or no abilities to depolymerise organic macromolecules, they primarily acquire inorganic nutrients mobilised by saprotrophs. Conversely, most ECM fungi can, to different extents (Lindahl & Tunlid, 2015; Zak *et al*., 2019), produce enzymes involved in breaking down organic compounds and therefore have earlier access to organic nutrients than AM fungi. As a result, AM‐dominated vegetation displays a mainly inorganic nutrient economy as a result of the fast decomposition of high quality litter pools and high rates of carbon and nitrogen mineralisation. By contrast, ECM‐dominated stands are largely characterised by an organic nutrient economy, fuelled by low‐quality litter pools and showing slow rates of carbon and nitrogen turnover, which results in limited release of inorganic nutrients (Phillips *et al*., 2013).

The ericoid mycorrhizal type is a third widespread type of mycorrhiza, occurring mostly in tundra, boreal forest, Mediterranean areas, and South Africa (Read, 1991; Soudzilovskaia *et al*., 2019). This type of association is formed exclusively by the plant members of the Ericaceae family, in association with Ascomycota fungi or by Basidiomycetes in the Sebacinales order of Agaricomycetes. The fungi colonise the plant roots in a manner similar to that of AM fungi. They penetrate the root cells, and form hyphal structures (coils) within cells. However, in contrast with AM, each individual cell is colonised by hyphae stretching from the root surface.

Orchid mycorrhiza is formed exclusively by plants that belong to the *Orchidaceae* family, and mostly by fungi from the *Basidiomycota* division, most particularly from the genus *Rhizoctonia* (Brundrett & Tedersoo, 2018). Orchid mycorrhizas are widespread as well, but due to scarcity of the host plants they are never abundant (Jacquemyn *et al*., 2017; Selosse *et al*., 2017). In this type of mycorrhiza, fungi also form hyphal coil structures (called pelotons) within the root cells. Fungi penetrate the root cells from neighbouring cells or from the root surface. Orchid seeds are very small (*c*. 0.1–1 mg) and contain little nutrients. Therefore, seedlings of all orchid plants have a period when they are entirely dependent on their mycorrhizal partners for nutrient uptake. Moreover, the majority of orchid plants does not germinate before they get infected by an appropriate fungus. Some orchid plants are mycotrophic.

Finally, there is no consensus among researchers whether structures that are formed by dark septate endophyte (DSE) fungi that form mycorrhiza‐like associations with vascular plants should be interpreted as mycorrhizas (Jumpponen & Trappe, 1998; Jumpponen, 2001; Newsham, 2011). The nature of physiological relationships between plants and DSE fungi, as well as the ecological role of DSE, are still poorly understood (Mandyam & Jumpponen, 2005). DSEs are globally spread, but most abundant in cold and cool regions (Newsham, 2011; Ruotsalainen, 2018). These fungi have been found in *c*. 600 plant species within 114 families (Jumpponen & Trappe, 1998). Fungi known to date that form DSE mycorrhiza belong to *Ascomycota*. The DSE fungi have darkly pigmented septate hyphae that penetrate the root cell and form so‐called *microsclerotia*, intracellular groups of hyphae having thick‐walled cells. Often DSE fungi co‐occur together with the recognised types of mycorrhiza discussed above (Jumpponen & Trappe, 1998).

Although the majority of plants can form only one type of mycorrhizal association, and this type is plant‐species dependent (Wang & Qiu, 2006; Smith & Read, 2008; Brundrett & Tedersoo, 2018), some species are able to simultaneously or time‐sequentially form two or even more types of mycorrhiza (Wang & Qiu, 2006; Akhmetzhanova *et al*., 2012; Brundrett & Tedersoo, 2018). Currently, several large databases describe plant‐species mycorrhizal association types (Wang & Qiu, 2006; Akhmetzhanova *et al*., 2012; Hempel *et al*., 2013) that can be consulted for the expected type of mycorrhizal association of a plant species. However, recent assessments have discovered some errors in these listings, mostly due to inclusion of older reports, based on (at that time) poorly developed techniques of mycorrhizal assessments (Brundrett & Tedersoo, 2019). This is particularly true for plants for which double colonisation is expected, and for particular groups of *Ericales* (Brundrett & Tedersoo, 2018, 2019). The recent work of Tedersoo and Brundrett (Tedersoo & Brundrett, 2017; Tedersoo, 2017a,b) highlights plant genera for which it is especially useful to check the mycorrhizal type(s) directly on roots instead of relying on previous assessments. Many of these potential errors are acknowledged and highlighted in the recently published new FungalRoot database (Soudzilovskaia *et al*., 2020). However, formal inspection of a mycorrhizal type is generally recommended. Finally, it is important to consider that only *c*. 14 000 plant species have been properly examined for mycorrhizal types (Brundrett, 2009; Soudzilovskaia *et al*., 2020).

#### a. Sampling recommendations

See section **VI. Experimentation and sampling in laboratory and field**.

#### b. Storage and processing

To preserve root material for microscopic mycorrhizal identification, the roots should be dried completely (60°C for 48 h) or immersed into 60–95% (v/v) ethanol or methanol. In the latter case, the roots in ethanol/methanol should be stored at 4°C. Formalin‐based preservations have been commonly used, but is not recommended because of its toxicity.

#### c. Measurement procedure

##### Root staining

The preserved root samples should be soaked in deionised water for 8–10 h and rinsed three times to remove ethanol/methanol. before staining, roots should be cleared in 10% (v/v) KOH in a water bath (70°C, 30 min 7 h), to remove natural pigments (tannins, polyphenols) and cell content. The time of cleaning is species‐dependent and is to be established experimentally, depending on the degree of root pigmentation. Heavily lignified or pigmented roots may require a subsequent bleaching with 3.5% of alkaline hydrogen peroxide H_2_O_2_ or sodium hypochlorite (NaCl) in a water bath (70°C, *c*. 30 min). For ECM roots, bleaching is sometimes needed to remove the excess of secondary compounds camouflaging the Hartig net, which can be determined when assessing the roots under a stereomicroscope. Also, the bleaching time is species‐dependent and needs to be established experimentally to achieve a balance between the absence of pigments and quality of staining of mycorrhizal structures. After clearing (and eventual bleaching) roots should be briefly (0.5 min) acidified with 1% (v/v) HCl or acetic acid, being immersed into a new acid solution (HCl or acetic acid), to improve staining efficiency. Roots should be stained shortly after bleaching.

Various staining techniques are available for visualising mycorrhizal structures. We recommend the protocol proposed by Vierheilig *et al*. (1998) and further developed by Walker (2005). This comprises immersing the roots into a tube with a blue ink (e.g. ink of Parker, which proved to work well) in 1% (v/v) HCL, and placing the tube into a water bath at 70°C for 30 min. Alternatively, roots can be stained with 0.05% (v/v) trypan blue (in a 1 : 1 : 1 mixture of lactic acid, glycerol, and deionised water) for 15–30 min at 70°C (Koske & Gemma, 1989), but is not recommended here as it is potentially carcinogenic. Subsequently, the roots should be de‐stained in a lactic acid glycerol solution for 10 min to 8 h (here also the duration is species dependent). If needed, the stained roots could be preserved in the lactic acid glycerol for a period up to 3 yr before microscopic examination. Preservation in lactic acid glycerol de‐stains the roots but not the fungal structures, improving the visibility of mycorrhizal structures.

##### Microscope visualisation

Assessment of the type of mycorrhizal association is best conducted on root tips under a stereomicroscope to detect ECM, and subsequently on root sections along and across the roots under a compound microscope to establish the presence of other mycorrhizas. Using a stereomicroscope (×5–10 magnification), roots can be examined in Petri dishes with water to keep roots moist. Roots can be moved with tweezers, but keep in mind that roots may be fragile due to the clearing and staining process. Often ECM can be detected on unstained roots, but to visualise the Hartig net, which is a diagnostic feature of ECM, clearing and staining is advisable. Moreover, staining is essential for plants that show only minimal differences in branching and thickness of colonised vs uncolonised roots.

For AM and orchid mycorrhizas and presence of DSE fungi, we recommend using an optical microscope (*c*. ×200 magnification). For AM, the roots should be arranged on microscopic slides and examined for the presence of fungal hyphae, vesicules and arbuscules (Fig. 25). The observation of fungal hyphae only is not enough to validate the presence of AM fungi. Detecting ericoid, arbutoid, pyroloid and monotropoid mycorrhizas requires making cross‐sections (i.e. transverse section, performed with a blade) of roots to assess the presence of coils and fungal hyphae penetrating the cells and the intracellular space. See also section **XIII. Root anatomy** for a protocol on making root cross‐sections.

Root fragments can be mounted on a microscopic slide with lactic acid glycerol solution, which is covered by a transparent lid. If the slides need to be preserved, they could be sealed with an ordinary transparent nail lacquer.

#### d. Future research directions

There is increasing evidence that the mechanism and magnitude of mycorrhizas’ impacts on ecosystem functioning depends on the mycorrhizal type involved, but also on differences among mycorrhizal fungi within each type (Veresoglou *et al*., 2012a; Phillips *et al*., 2013; Averill *et al*., 2014; Lindahl & Tunlid, 2015; Soudzilovskaia *et al*., 2015b; Terrer *et al*., 2016). Therefore, a better understanding of the identity of plant and fungi involved in mycorrhizal associations at local, regional and global levels (e.g. Soudzilovskaia *et al*., 2019) would be a first step to understand the role of mycorrhizas in global biogeochemical cycles. Furthermore, Rillig (2004) framed the mycorrhizal impacts on ecosystems functioning as *direct* (via presence and functioning of mycorrhizal fungi in soil) and *indirect* (via mechanisms associated with plant nutrition and fitness). Recent findings demonstrate that these pathways are tightly connected, mostly via the coupling of the carbon and nitrogen pathways (Terrer *et al*., 2016, 2018; Lin *et al*., 2017; Treseder *et al*., 2018), but little information is known of the mechanisms implying interactions between soils, plants and fungi that underlie these connections.

### 2. Root mycorrhizal colonisation intensity

*Root mycorrhizal colonisation intensity* is the degree of completeness of root association with mycorrhizal fungi (typical units: %). The method used to estimate root mycorrhizal colonisation intensity of ECM differs from this used for AM, ericoid, arbutoid, orchid and DSE fungi and results are therefore not comparable between the two methods. For ECM, root mycorrhizal colonisation intensity is generally represented by the percentage of root tips colonised by ECM fungi. For other mycorrhizal types, root mycorrhizal colonisation intensity represents the percentage length of roots infected by mycorrhizal fungi.

It can be measured on roots of the first three orders, although most often researchers use only the first‐order roots, given the prevalence of mycorrhizal structures in young roots. Therefore the exact root order(s) examined should always be reported when presenting data on mycorrhizal colonisation.

Although it is widely recognised that the intensity of mycorrhizal colonisation is a measure of the intimacy of relationship between plants and fungi, the exact relationship between root colonisation intensity and plant function is still poorly understood. For AM, it has been shown, however, that an increase in intensity of root colonisation by fungi positively correlates with the efficiency of plant phosphorus uptake and relative growth rate (Treseder, 2013). Root mycorrhizal colonisation intensity in the field has also been linked to plant foraging strategies (e.g. Eissenstat *et al*., 2015) and plant growth rate in *ex situ*, unfavourable conditions for mycorrhization (e.g. Elumeeva *et al*., 2018). For ECM plants, it has been demonstrated that colonised roots show lower decomposition rates, than noncolonised roots, and likely to be contributing to soil carbon accumulation (Langley & Hungate, 2003) and more generally that ECM directly and indirectly modify the biochemical composition and amount of soil organic matter (Zak *et al*., 2019). Nonetheless, we note that the composition of fungal community colonising roots (see section **XIX. 3. Root mycorrhizal fungal community composition**) is likely to be as important as the mycorrhizal type.

#### a. Sampling recommendations

See section **VI. Experimentation and sampling in laboratory and field**.

#### b. Storage and processing

See section **XIX. 1. Mycorrhizal root type**. In addition, note that to accurately estimate the percentage of root length that is colonised, unbranched pieces of roots are required, or the length of branches should be taken into account when root length is calculated. We advise to cut into pieces of *c*. 1 cm, to avoid a branching pattern. To count the presence and absence of ECM root tips, a stereomicroscope with ×10 magnification is recommended.

#### c. Measurement procedure

##### Arbuscular, ericoid, arbutoid, orchid and DSE mycorrhizas

**Fig. 27:**
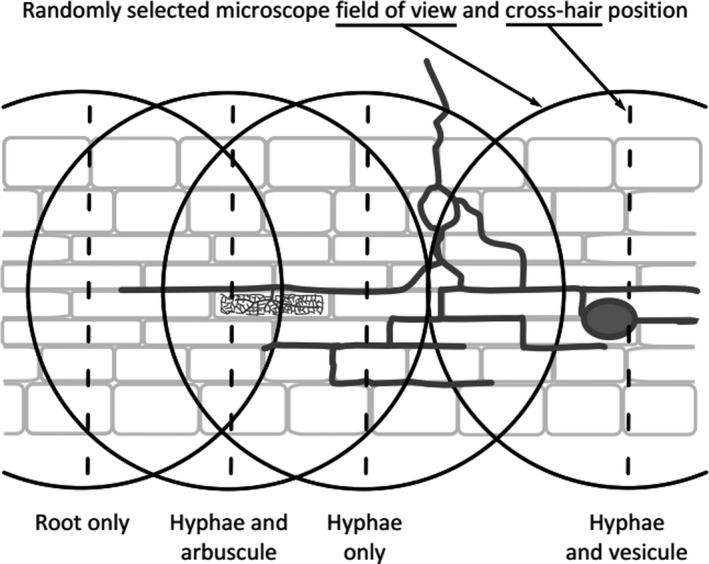
Microscopic examination of roots to quantify arbuscular mycorrhizas (AM) (adapted from Brundrett *et al*., 1996).

By far the most popular method to examine root colonisation intensity by these types of mycorrhizal fungi is the root intersection method proposed by McGonigle *et al*. (1990). It encompasses examination of the fraction (%) of root length colonised by mycorrhizal fungi. Root colonisation by AM, ericoid, arbutoid, orchid and DSE fungi should be examined by moving the microscopic field view with constant distance between passes, of *c*. 0.1 mm, and observing each intersection of root and vertical cross‐hair eyepiece (Fig. 27) for at least 100–150 intersections (Giovannetti & Mosse, 1980). The absence or presence of fungal structures (i.e. hyphae, arbuscules, vesicules, spores for arbuscular mycorrhizas; coils for ericoid and arbutoid mycorrhizas, pelotons for orchid mycorrhizas; microsclerotia for DSE mycorrhizas) at each intersection should be marked. The percentage colonised intersects of the total number of examined intersects provides information on intensity of fungal root colonisation.

##### Ectomycorrhizas and monotropoid mycorrhizas

Root tips colonised by ECM and monotropoid mycorrhizas are covered by a hyphal mantel and contain an active Hartig net zone. The number of these colonised root tips as a percentage of the total number of root tips, or the length of the root zone containing mycorrhizal tips compared with the total first‐order root length is typically used to quantify the intensity of the ectomycorrhizal and monotropoid mycorrhizal associations.

#### d. Future research directions

The extent to which root mycorrhizal colonisation intensity contributes to plant vitality, growth rate and growth strategies is still poorly understood (Treseder, 2013; Elumeeva *et al*., 2018). Understanding these relationships is complicated by the variability of mycorrhizal colonisation intensity in laboratory conditions. Nonetheless, intraspecific variation in root mycorrhizal colonisation intensity is not larger than that of other plant traits (Soudzilovskaia *et al*., 2015a), suggesting that, in natural conditions, the intensity of root mycorrhizal colonisation is potentially valuable in interspecific trait comparisons (Violle *et al*., 2007).

### 3. Root mycorrhizal fungal community composition

*Root mycorrhizal fungal community composition* is the identity of fungal species colonising the root.

Within a given mycorrhizal type, roots of the same plant species can be colonised by multiple fungal species. Also, the identities of mycorrhizal fungal species in plant roots can control competitive interactions between plant species, with distinct plant–fungal combinations being stronger than others (e.g. Scheublin *et al*., 2007). There is a widely accepted presumption that not all mycorrhizal fungal associations are equally beneficial for plant nutrition and pest protection (van der Heijden *et al*., 2015). Similarly, mycorrhizal fungi, especially so ECM, vary in the impact of their extraradical mycelium on biogeochemical processes (Lindahl & Tunlid, 2015; Zak *et al*., 2019). However, the mechanisms underlying these phenomenon as well as their ecological consequences still need to be investigated further. Therefore, examination of fungal community composition within roots is an important tool to understand the ecological impacts of root fungal diversity on functioning of ecosystems.

#### a. Sampling recommendations

See section **VI. Experimentation and sampling in laboratory and field**.

#### b. Storage and processing

To preserve the roots for DNA extractions of fungal material to analyse root fungal community composition, the roots samples are preferably freeze dried, but can also be stored at −20°C.

#### c. Measurement procedure

Ectomycorrhizal root tips, or root segments for other types of mycorrhiza, should be subjected to extraction of total genomic DNA or in some cases RNA. The extraction can be done using standardised plant polymerase chain reaction (PCR) kits, following the manufacturer’s instructions. Subsequently, the regions relevant as markers for the fungi of the mycorrhizal group of interest should be PCR amplified. The selection of regions for this amplifications is a topic of debate in current literature (Lindahl *et al*., 2013). Options include ITS1 and ITS2 for ECM (Nilsson *et al*., 2019), and ITS2, SSU4, SSU9 and LSU3 for AM (Krüger *et al*., 2009). Given the rapid development of this field, we advise the reader to consult the most up‐to‐date literature to select a suitable primer set. Sequences of mycorrhizal fungi vs those of other root‐associated fungi should next be separated using reference databases. More details on the fungal community analysis by high‐throughput sequencing and on the available platforms can be found in reviews by Nilsson *et al*. (2019) and Castaño *et al*. (2020).

#### d. Future research directions

We still need more precise understanding of how the identity of mycorrhizal fungi in associations within plant roots link to the overall soil microbial composition, plant community composition and soil processes. Fast development of new molecular techniques allows more and more accurate disentangling of the microbial community composition and linking community structure to function, via profiling of microbial traits such as, for instance, enzymatic activities. Quantitative assessments of fungal community composition using novel molecular techniques, such as droplet‐digital PCR (ddPCR; Hindson *et al*., 2011, 2013), can inform us on how individual mycorrhizal fungi or their functional groups affect biogeochemical cycling and ecosystem structures in quantitative terms. Also, while it has been demonstrated that individual AM and ECM fungi can have different strategies towards colonisation of roots and soil (Agerer, 2001; Hart *et al*., 2001; Hart & Reader, 2002; Lilleskov *et al*., 2011), we need to better understand to what extent these strategies are complementary and how they relate to soil processes as well as plant nutrition.

## 20 Nitrogen‐fixing symbioses

Thousands of plant species have the ability to form symbioses with nitrogen (N)‐fixing bacteria (Doyle, 2016; Tedersoo *et al*., 2018), representing eight origins of the symbiosis, all potentially derived from one cryptic innovation over 100 Ma (Werner *et al*., 2014). These eight origins represent a diversity of plant and bacterial symbiont identities, nodule morphologies and strategies of host regulation. Yet, among these N‐fixing plant species (i.e. N‐fixers) the fundamental relationship between plant and symbiont is consistent: plant‐derived carbon compounds are allocated to bacteria in specialised root structures (broadly referred to as nodules), which regulate oxygen for nitrogenase enzymatic activity, and the fixed N is then assimilated into organic N compounds and translocated throughout the plant (Pawlowski & Bergman, 2007; Lambers & Oliveira, 2019). Beyond the nodule‐forming symbiosis, the degree to which endophytic associations are prevalent across plant species or how much N they contribute to the host plant and ecosystem are unclear. For some plant species, free‐living or associative N‐fixing bacteria in the rhizosphere can fix a substantial amount of N (e.g. Roley *et al*., 2018), but it remains unresolved if these associations are true symbioses. Here, we focus on the N‐fixing symbiosis housed in specialised root structures, which we collectively refer to as nodules, as they represent a root trait and a quantifiable process, with widely known significance for the plant and ecosystem.

The N‐fixation symbiosis evolved among legumes (Fabaceae) and *Parasponia* species (Cannabaceae) with ‘rhizobia’ bacteria (*α*‐proteobacteria and *β*‐proteobacteria), in numerous plant families (e.g. Betulaceae, Rosaceae, Rhamnaceae) with actinorhizal bacteria (i.e. *Frankia*; Doyle, 2016), and in some members of the Cycadophyta with cyanobacterial symbionts (Lindblad, 2009; Tedersoo *et al*., 2018). Across these association types, there are diverse nodule morphologies, relating to differences in growth form and organ structure, that occur across biomes (Lindblad, 2009; Sprent, 2009; Doyle, 2016). For example, among rhizobial N‐fixing plants, nodules are either determinate or indeterminate in their growth form (Sprent, 2009). Actinorhizal nodules are composed of lobes (rhizothamnia), which can be elongated or coralloid, of which only the apical portions contain the N‐fixing symbiont (Huss‐Danell, 1997). Most rhizobial and actinorhizal nodules are perennial (Huss‐Danell, 1997; Sprent, 2005), but rhizobial nodules can also be ephemeral if soil conditions become unfavourable (e.g. Batterman *et al*., 2013b). Among cycads, the N‐fixing symbiosis forms within perennial, coralloid roots, rather than conspicuous nodules and these root structures are formed by the plant before colonisation by cyanobacteria (Lindblad, 2009).

The N‐fixation symbiosis can have profound effects on natural and managed ecosystems by supplying additional N and affecting plant growth rates, with consequences at community and ecosystem scales (Boring & Swank, 1984; Binkley *et al*., 1992; Von Holle *et al*., 2006; Batterman *et al*., 2013a). The N‐fixing symbiont allows plants access to the vast pool of atmospheric N_2_, which may explain the higher concentration of N in their foliage (Cornelissen *et al*., 1997; Fyllas *et al*., 2009; Kurokawa *et al*., 2010; Adams *et al*., 2016) and fine roots (Freschet *et al*., 2017), and higher growth rates among N‐fixing leguminous trees vs nonfixing trees (Batterman *et al*., 2013a; Menge & Chazdon, 2016; Taylor & Menge, 2018; but see Lai *et al*., 2018). However, many of these generalisations are based on N‐fixing legumes, and it remains unclear whether and how consistently such patterns extend to actinorhizal and cyanobacterial N‐fixing species. Access to atmospheric N_2_ can improve the competitive success of N‐fixers relative to nonfixers, particularly in N‐poor soils (Luo *et al*., 2014; Wurzburger & Miniat, 2014). Once N‐fixing plants die or shed litter, their fixed N enters the soil system, having broader effects on community and ecosystem processes. For example, N‐fixers can lead to the accumulation of soil N stocks (Boring & Swank, 1984), and increase the rates of nitrification (Montagnini *et al*., 1986), even decades after the N‐fixer has been removed (Von Holle *et al*., 2013). Invasive N‐fixing plants can alter ecosystem properties through their enrichment of N and facilitate future invasions of exotic plant species (Vitousek & Walker, 1989; Von Holle *et al*., 2006). In agroecosystems, several N‐fixing legumes are of high economical and societal value, as they provide a nutritious food source and/or improved ecosystem functioning as a cover crop (Azani *et al*., 2017).

The ecological significance of N fixation strongly depends on how the symbiosis is regulated by the plant (i.e. strategies of fixation). For some plant species, such as tropical leguminous trees (Barron *et al*., 2011) and some herbaceous legumes (Menge *et al*., 2015; Ament *et al*., 2018), the symbiosis is upregulated or downregulated by the plant based on soil N supply and plant N demand (facultative *sensu* Menge & Hedin, 2009). For other plant species, such as actinorhizal temperate or boreal trees and shrubs, the symbiosis is less responsive to changing N conditions (obligate *sensu* Menge & Hedin, 2009). These contrasting fixation strategies between tropical fixers and extratropical fixers effectively regulate the amount and timing of N fixation over the course of plant succession following disturbance, and appear to have evolved in response to the N cycling conditions dictated by climate (Sheffer *et al*., 2015). However, fixation strategies are not consistent within association types (i.e. within rhizobial or actinorhizal N‐fixers), and are likely to persist along a spectrum from obligate to facultative (Menge *et al*., 2015). This spectrum of strategies can even extend to over‐regulation when plants fix less N than they need (Menge *et al*., 2015). As a result of this variation in fixation strategy, the degree to which plants reduce the activity of their N‐fixing symbiont under increasing N supply can vary widely, among species of both rhizobial and actinorhizal fixers (Binkley *et al*., 1994; Menge *et al*., 2015; Ament *et al*., 2018). In addition to N as a regulating force, the N‐fixation symbiosis can also be constrained by limiting resources including nutrients (e.g. phosphorus or molybdenum; Wurzburger & Hedin, 2016), light (Taylor & Menge, 2018) and water (Minucci *et al*., 2017), making this root trait highly dynamic within and across species of plants. Constraint by temperature is of specific interest, despite the observation that many herbaceous fixers can grow in cold conditions at high latitudes or altitudes. The dynamics of N fixation mean that observations and quantifications of the N‐fixation trait require careful consideration of the environmental context in which a plant is sampled.

### 1. Nitrogen‐fixation ability and association type

*Nitrogen‐fixation ability* is the genetic capacity of a plant species to form N‐fixing association (categories: N‐fixing, non‐N‐fixing).

*Nitrogen‐fixation association type* of a plant species is the type of N‐fixing association the plant may form (categories: rhizobial, actinorhizal and cyanobacterial).

The association type of N‐fixers should be regarded as a potential for the symbiosis to form, and should not be assumed for all plants in all ecosystems, given the complexity of fixation strategies and the potential for resource constraints on the symbiosis (see above).

#### a. Assessment of N‐fixation ability and association type

The three association types – rhizobial, actinorhizal and cyanobacterial – are strongly conserved at the plant species level and can be predicted from previous reports (Tedersoo *et al*., 2018).

#### b. Future research directions

Although we have confirmed that some plant species have the potential to form N‐fixing symbioses, many species that have the potential to be N‐fixers have not been examined. Future research is needed to determine the status of these plant species. For example, of the 745 genera of legumes, about a third have not been rigorously tested for their potential to fix N (Afkhami *et al*., 2018). Phylogenetic analyses can help with inferences about the N‐fixation status of the untested species or genera (Azani *et al*., 2017; Afkhami *et al*., 2018; Tedersoo *et al*., 2018), since fixation ability is generally phylogenetically constrained (with a few exceptions; Sprent, 2009). Beyond nodulating plants, additional research is needed to examine whether different types of N‐fixation symbioses exist. For example, recent research has discovered N‐fixing bacteria within plant organs including roots, stems or foliage (Burbano *et al*., 2015; Moyes *et al*., 2016; Roley *et al*., 2018), but it is unclear how widespread these associations are, beyond a few species of plants, or how much N they supply to the plants and ecosystems.

### 2. Nodule investment

*Nodule investment* is the ratio of the root nodule dry mass to the total plant dry mass (typical units: mg g^−1^). It represents the proportion of biomass plants invest in nodules relative to other plant parts.

The presence of nodules reveals the potential for a plant to symbiotically fix N, which can be particularly relevant to assess for plants that are facultative N‐fixers (i.e. they may not form the symbiosis under all conditions) or where limiting resources might constrain the expression of the symbiosis. The degree of nodule investment can indicate plant demand for N, where a greater investment in nodules leads to a greater potential for fixation per unit plant biomass. However, nodule presence in itself does not always equate to N fixation (e.g. Menge *et al*., 2015), and nodule investment differs from absolute nodule biomass. Nodule investment is a useful metric to compare plant strategies and per‐biomass fixation potential, whereas absolute nodule biomass may give a better estimate of fixation potential at the ecosystem level.

#### a. Sampling recommendations

See section **VI. Experimentation and sampling in laboratory and field**. Sampling should be designed to capture the investment plants make in nodule biomass. In glasshouse and field studies of small plants, an optimal sampling design would harvest the whole plant so that all nodule and total plant biomass can be quantified. For larger field‐sampled plants or where plants cannot be fully harvested, nodule biomass can be extrapolated from soil cores or excavated areas to a per plant basis by sampling within a specified radius of the plant stem. The depth of nodule sampling with soil cores will depend on the plant species and ecosystem type, and should be determined before field sampling. For example, in moist temperate and tropical ecosystems, most nodules tend to grow in the top 15 cm of soil (Boring & Wayne, 1984; Barron *et al*., 2011) but, in arid systems, nodules can be concentrated at 50–60 cm (Kummerow *et al*., 1978) and even deeper than 1 m (Virginia *et al*., 1986). The sampling radius around individual plants should also be determined before sampling, as root systems vary in their horizontal distributions. The smaller the sampling radius, the higher the probability of encountering nodules among randomly distributed soil cores, but there is a greater chance of missing root nodules further from the plant, leading to an underestimate of total nodule mass per plant. However, the larger the sampling radius, the longer the amount of time needed to examine the soil volume for nodules. By quantifying nodule biomass among sampled soil cores of a given area, investigators can extrapolate total plant nodule mass supported by a plant. To calculate nodule investment for large woody plants, above‐ground or whole‐plant biomass can be estimated from measures of stem diameter and height using allometric relationships (e.g. Chave *et al*., 2005).

For large trees, for which it is difficult to quantify total plant and nodule biomass (because allometric relationships have not been determined for the species, or nodulation is challenging to quantify across the entire root system), an alternative approach is to quantify nodule biomass per unit area (e.g. Barron *et al*., 2011; Batterman *et al*., 2013; Wurzburger & Hedin, 2016). Another alternative is adaptive cluster sampling (ACS), which involves sampling soil cores across a landscape using a stratified approach (Sullivan *et al*., 2014; Winbourne *et al*., 2018). This approach is not conducive to quantify nodule investment per plant, but is useful for ecosystem‐level measurements of nodulation when plant identity and potential to form the symbiosis are unknown.

#### b. Storage and processing

See above sections on **Field** or **Laboratory experiment and sampling**. Soil samples and nodules should be kept cool and sealed in plastic bags and assessed within 1 wk to ensure that nodule biomass does not decompose. Soil cores should be carefully extracted from the soil, and all soil in the core should be examined for nodules because nodules can break off roots when soil is disturbed. Nodule processing can be done in the field, however laboratory‐based processing with proper lighting is preferred, as some nodules are small and easily overlooked.

#### c. Measurement procedure

Only active nodules (those likely to fix N) should be counted in the nodule investment metric. For legumes, nodules can be checked for activity by cutting open nodules and verifying a moist and pink interior, which signifies the presence of leghaemoglobin. Determining nodule viability is more challenging for actinorhizal and cyanobacterial nodules, as activity is often concentrated in the apical lobes of nodules. In these cases, nodules can be classified as inactive if they are dry and brittle. If nodules are not further assessed for their fixation rates (see section **XX. 3. N‐fixation rate**), cutting open and assessing all nodules improves the precision of this procedure to confirm viability and the potential for N fixation. Nodules used for measurement of fixation rates (as below) can be cut open after analysis. Nodules should be oven dried at 60°C to a constant weight along with plant biomass to calculate plant nodule investment, as the ratio of nodule biomass (mg) to plant biomass (g).

#### d. Future research directions

Future research should examine whether nodule investment scales predictably with plant biomass and/or size, and how scaling factors vary among woody and herbaceous N‐fixers. In addition, we still poorly understand where nodules occur along root systems, including what soil depth, horizontal distance from the stem, and what root orders produce nodules. Such characteristics are likely to vary both among and within N‐fixation association types and plant species.

### 3. N‐fixation rate

*N‐fixation rate* is the amount of N originating from N_2_ fixed per unit plant dry mass and time (typical units: mg N kg^−1^ yr^−1^). In situations where plant biomass cannot be measured, or if an ecosystem‐level rate is desired, fixation rates can be expressed on an area basis (kg N ha^−1^ yr^−1^).

Quantifying the rate of N fixation identifies the functional significance of nodulation and provides a means for scaling up fixation rates from individual plants to the ecosystem. Assessing the response of N‐fixation rate to additions of N can help to identify the N‐fixation strategy of individual N‐fixers. Furthermore, the response of N fixation to other treatments (i.e. water, light and other nutrients) can help elucidate controls on the fixation process.

There are four common approaches to quantify N‐fixation rate. The first approach is to conduct acetylene reduction assays (ARAs) with root nodules. This approach is inexpensive and rapid but has several difficulties and can only provide a quantitative measure of fixation if appropriately conducted and calibrated with ^15^N_2_ incubations. A second method, and one that we advocate instead of ARAs, is to rely solely on ^15^N_2_ incorporation into nodule biomass to quantify rates of fixation. A third method involves a ^15^N pool‐dilution approach to quantify the percentage of N derived from the atmosphere after applying low levels of ^15^N‐enriched N to soil. A fourth method is to calculate N derived from the atmosphere using the natural abundance of ^15^N in tissues of N‐fixers and nonfixer reference plants. These third and fourth approaches must be combined with measures of productivity and tissue N concentrations to allow for estimates of N fixation rate. All four methods have limitations and require special considerations. The appropriate approach for a given study may depend on a variety of factors, including the question, ecosystem, timing of experiment or observation and/or resources available to the researcher (see Shearer & Kohl, 1986; Unkovich & Pate, 2000).

#### a. Sampling recommendations

See section **VI. Experimentation and sampling in laboratory and field**. Measurement of fixation rates (using ARAs or ^15^N_2_ incorporation) can be done on nodules collected in soil cores, as described above or on nodules found on roots excavated from the soil specifically for the purpose of measuring rates. Note that all measurements should be done immediately. Because of the dependency between photosynthesis and fixation, maintaining light intensities during measures of fixation with ARA or ^15^N_2_ incubations is critical, especially for cyanobacterial N‐fixers. For the ^15^N natural abundance and pool‐dilution approach (see below) nodules need not be sampled and, rather, plant foliar or whole‐plant biomass samples are used to quantify the percentage of plant N derived from the atmosphere, along with measurement of plant productivity and plant N concentration allow for estimation of fixation rate on a plant or area basis.

#### b. Storage and processing

Nodules should be attached to several cm of root fragments without intensive cleaning for both ARAs and ^15^N_2_ incorporation (e.g. by carefully brushing with a small paintbrush to remove the bulk of soil) because N‐fixation rates are negatively affected by nodule disturbance. Furthermore, excavated nodules should be incubated immediately for ARAs because acetylene reduction rates decline rapidly over time.

#### c. Measurement procedure

##### Acetylene reduction assay (ARA)

ARAs are rapid and inexpensive assays that rely on the ability of nitrogenase to reduce acetylene (C_2_H_2_) to ethylene (C_2_H_4_) as a proxy for N fixation. The technique has been the subject of much debate (Vessey, 1994), and many researchers are opposed to this method (Minchin *et al*., 1994). Yet it continues to be used (Myrold *et al*., 1999; Carlsson & Huss‐Danell, 2003) and can be appropriate if the following limitations are considered (Unkovich & Pate, 2000). First, the disturbance associated with sampling nodules in the field can reduce the activity of nitrogenase (Minchin *et al*., 1986), which suggests that short‐term fixation measures (ARA and ^15^N_2_ incorporation) underestimate true fixation rates. The second issue is acetylene‐induced decline in fixation rate, which has been observed in some, but not all, rhizobial N‐fixers (Minchin *et al*., 1983) and actinorhizal fixers (Schwintzer & Tjepkema, 1997), and has been attributed to an increased resistance of O_2_ diffusion, which reduces the rate of adenosine triphosphate production and, therefore, electron transport to nitrogenase. Therefore, the main debate with ARAs stems from its inaccuracy in quantifying electron flux. This problem can be minimised by the use of flow‐through systems to quantify predecline rates of ethylene production (Minchin *et al*., 1986) or using maximum rates in closed‐chamber incubations (i.e. less than 3 min; Myrold *et al*., 1999). However, as many researchers seek to quantify the rate of N fixation, not electron flux in itself, the use of ^15^N_2_ incorporation in conjunction with ARAs can provide a conversion factor to N‐fixation rate that avoids a reliance on electron flux. Furthermore, it is necessary to empirically determine C_2_H_2_ : N_2_ conversion factors because nitrogenase also produces H_2_, and the proportion of electrons allocated to H_2_ production can vary among species resulting in conversion factors that vary widely from the originally theorised ratio of 3 : 1 or 4 : 1 (Hardy *et al*., 1973; Witty & Minchin, 1988).

ARAs have been described in detail by others (Myrold *et al*., 1999), so here we provide an abbreviated description, with a focus on root nodules. Ideally, gas‐tight, flow‐through chambers that contain the entire plant or below‐ground system of a plant, would allow ARAs to be conducted in real time and with no destruction to the plant. However, this method requires substantial laboratory infrastructure and is unrealistic for field‐grown plants. For extracting nodules from plants, we make a few recommendations to minimise the known pitfalls of ARAs. For example, nodules should be excavated with care and incubated immediately because acetylene reduction rates decline rapidly over time. Nodules should be attached to several cm of root fragments and carefully brushed to remove soil (with a small paintbrush) to minimise disturbance to nodules, and placed in *c*. 100–250 cm^3^ gas‐tight chambers, such as canning jars, where 10% of the headspace is replaced with acetylene (via a gas‐tight septum or port to remove and replace gas). The quantity of nodule biomass used in each incubation will depend on the size of the chamber, the duration of the incubation and activity of the nodule. A relatively large amount of nodule biomass (*c*. 0.5 g dried nodule biomass) may be necessary to detect ethylene production over short time scales.

Acetylene can be produced by reacting calcium carbide (CaC_2_) with water (caution: acetylene is a flammable gas) or obtained in pressurised form. Because nitrogenase is temperature sensitive, chambers should be maintained at ambient soil temperature to quantify more realistic field activity. Chamber headspace should be sampled at least two times at 5–15 min intervals over a period of 20–30 min. The length of incubation should be determined based on trials to avoid capturing the decline of acetylene reduction over time. Some advocate that incubations should last no more than 3 min to capture the predecline rates of ethylene production (Myrold *et al*., 1999). Gas samples are stable if stored in gas‐tight evacuated vials until analysis with a gas chromatograph equipped with a flame ionisation detector. Butyl rubber septa are preferred since other types of rubber can generate ethylene. After background contamination of ethylene and nodule or root‐produced ethylene is accounted for with control (no nodules with acetylene) and blank (with nodules, no acetylene) samples, the rate of ethylene production over time can be quantified for each incubation. After cleaning and drying nodules (60°C to a constant weight) nodule dry mass is measured and the rate of acetylene reduction (e.g. µmol g^−1^ h^−1^) can be calculated by the following equation:

(Eqn 12) $$\begin{array}{l} \mathrm{Acetylene}\;\mathrm{reduction}\;\mathrm{activity}\;\\ \;=\;\frac{\mathrm{Rate}\;\mathrm{of}\;\mathrm{ethylene}\;\mathrm{production}\;\times \;\mathrm{headspace}\;\mathrm{volume}}{\mathrm{Nodule}\;\mathrm{mass}} \end{array}$$

To convert acetylene reduction activity to a rate of N fixation, incubations with ^15^N_2_ should be performed on replicate nodule samples from the same plant or location, but where acetylene is replaced with ^15^N_2_ of a high atom per cent enrichment (i.e. 98%). Ideally, investigators would use the same chamber conditions, incubation times and nodule masses as the ARAs to account for any inconsistencies in rates due to incubation duration. Labelled gas can be costly and difficult to ship to remote field sites, and measuring N isotope ratios in plants is also costly. After incubations, nodules should be dried at 60°C to a constant weight and ground to a fine powder before analysis of N isotopes with a mass spectrometer. Nodules used for ARAs, or other nodules that were not enriched in ^15^N_2_ should be used to determine natural abundance of δ^15^N to calculate atom per cent enrichment of incubated nodules. Care should be taken to avoid cross‐contamination of samples. To determine an empirical ratio of ethylene to fixed N_2_, the moles of produced ethylene are divided by the moles of N_2_ incorporated into nodules.

##### ^15^N_2_ incorporation

The method of ^15^N_2_ incorporation into nodule biomass (described above for the conversion of ARA to N fixation) can also be performed in lieu of ARA. This method is more precise and does not suffer some of the drawbacks of ARAs, but may be subject to disturbance‐induced reductions in fixation rate, requires access to isotopically labelled ^15^N_2_ gas and is, therefore, substantially more costly.

##### ^15^N pool dilution

This approach requires more time investment, but has some benefits, because it more directly measures fixed N incorporation into plant biomass, integrates N fixation over a longer time (i.e. months or years), and avoids the pitfalls of acetylene reduction (i.e. potential declining rate over nodule incubation and disturbance of nodules) and the difficulty of transporting ^15^N_2_ gas to remote field sites. The downside of the pool dilution approach is that it can be more challenging to conduct on large woody shrubs and trees, as it may take a longer amount of time for ^15^N applications to sufficiently enrich large plants that have a substantial reserve pool of N (Danso *et al*., 1992; Myrold *et al*., 1999; Yelenik *et al*., 2013). Nonetheless, the method is still effective for many types of glasshouse and field studies.

The method involves enriching the soil N pool of a field plot or glasshouse pot with a sufficient amount of ^15^N contained in urea or inorganic N to ensure that soil‐based N sources have a different N isotope signature than the atmosphere. Careful consideration of the amount of N applied can prevent a fertilisation effect, which may cause facultative fixers to down‐regulate fixation, thereby leading to underestimates. For example, ^15^N applied at an enrichment of 5–20 atom per cent and a rate of 0.4–1 kg ha^−1^ has been deemed sufficient in some studies of woody plants (Danso *et al*., 1992; Myrold *et al*., 1999; Yelenik *et al*., 2013), but it is critical to consider that the total amount of N needed to achieve soil enrichment may be much greater when using N sources with a low atom per cent. Investigators should carefully select a nonfixing reference plant species similar in rooting habit and mycorrhizal association type as the N‐fixer in question to minimise the potential that the reference plant is accessing a different soil nitrogen source or has differential isotopic fractionation at the root–mycorrhizal interface when compared with the focal plant. In glasshouse studies, ideal reference species should be selected, but in field studies, investigators must select species that already exist at a given field site. As the reference plant cannot fix N, their foliar δ^15^N represents the δ^15^N in the available soil N pool. Foliar or whole‐plant samples of the N‐fixer and nonfixer should be collected after a sufficient amount of time for the applied ^15^N to enrich the available N pool. This could be a few months when plants are grown in pots in a glasshouse experiment, or it could be months or years in the field. The time required for this process can be estimated based on N uptake rates or N demands of plants, the pool of available soil N and the applied amount of N. Sampled biomass should be dried at 60°C to a constant weight, and ground to a fine powder for analysis on a mass spectrometer. The atom % enrichment (AE) of these organs can then be applied to the following equation to calculate the percent of plant N derived from the atmosphere (%Ndfa):

(Eqn 13) %Ndfa=N15AEreference-N15AENfixerN15AEreference×100

To calculate the total amount of N fixed on a plant basis, the fraction of N derived from the atmosphere can be multiplied by the rate of plant biomass N production (e.g. g N yr^−1^). The rate of plant biomass production can be determined by quantifying biomass growth using stem and height measures and allometric equations combined with measures of root productivity. Rates of biomass production can then be multiplied by plant N concentrations to calculate biomass N production. In field studies, by considering the density of plants per unit area, N fixation can also be expressed on an area basis.

##### ^15^N natural abundance

The natural abundance method relies on natural differences in soil and atmosphere δ^15^N to calculate the proportion of N from these two sources in N‐fixer biomass. The benefit of this method is that it does not require the addition of ^15^N and confounding fertilisation effects. However, the downside is that it lacks sensitivity and can be unreliable in many ecosystems where: (1) the δ^15^N of available soil N is too similar to the atmosphere to accurately partition N sources (Shearer & Kohl, 1986; Danso *et al*., 1992), (2) plants access different nitrogen pools with different signatures, or (3) where differential isotopic fractionation occurs at the plant–mycorrhizal interface across species. In these cases, it would be difficult to determine whether the lack of isotopic difference between N‐fixers and reference plants is truly due to a lack of fixation or some other factor. Nonetheless, this method has been useful, particularly in agricultural settings and paired with other methods. The procedure to sample biomass and calculate %Ndfa is the same as above for ^15^N pool dilution, and also requires measures of biomass N production to estimate N‐fixation rate on an area basis (see above).

#### d. Future research directions

Future research should examine why fixation rates vary among and within nodules, and how this varies across the morphological types of nodules found among the N‐fixation association types. Furthermore, it remains poorly understood how N‐fixation rates within the same plant species can vary across different bacterial species and how different plant–microbial partnerships may modulate responses to environmental conditions, such as differences in soil nutrients and water availability.

## 21 Root tip morphology and elongation

The apical zone of the root is a very important structure that is often called the ‘root tip’, including the very tip (apex) with its cap and meristem, and the young part, typically several centimetres long, distal to the last emerged lateral roots. This particular zone concentrates several developmental processes: root elongation through cell division and extension, cell differentiation from meristematic to mature cells, development of root hairs, initiation and development of lateral primordia. The anatomical structure which is gradually developed and differentiated in this zone is called the primary structure of the root (Esau, 1977). Beyond all these major developmental aspects, this zone has also a particular impact from a functional point of view, because of the important exchange fluxes with the environment, mainly through respiration, water and mineral uptake and exudation (Bidel *et al*., 2000; Nguyen, 2003; Laporte *et al*., 2013). Exchange functions are increased by root hairs, which are specialised subtypes of the epidermal cells (see section **XXII. Root hair morphology and development**).

Despite this prime importance of root tips in root system development and functioning, they are rarely considered explicitly in ecological studies, probably because of the methodological difficulties related to their observation. Indeed, they are particularly fragile, usually not lignified, and they tend to maintain an intimate contact with the surrounding soil that can be increased by sticky exudates and root hairs (McCully, 1999; Hinsinger *et al*., 2006).

Although we focus here mainly on quantitative traits, we would like to mention first that simple and qualitative observations can also be done on root tips either to characterise soil constraints or the growth status of individual roots. For example, various authors (Justin & Armstrong, 1987; Konôpka *et al*., 2009; Bengough *et al*., 2011) have pointed out the impact of soil strength and hypoxia on the shape of root apices. Moreover, the tortuosity of root tips, as well as longitudinal variations of diameter (prayer bead‐like roots), also reflect mechanical constraints. In many tree species (e.g. among Pinaceae and Rosaceae) the apical roots quickly change their colour from white to yellow and brown with ageing (Peterson *et al*., 1999), so that the number and length of white tips were used to evaluate temporal variations of growth activity (e.g. Head, 1966; Peterson *et al*., 1999; see section **XVI. Root dynamics**).

One of the main soil factors that affects root growth is the mechanical resistance of the soil to deformation. Measurements from a seedling root elongation assay in soil samples from 59 UK agricultural fields found that, even when the soil is relatively wet, root elongation was slowed by more than 50% in most samples, mainly as a result of the mechanical impedance of the soil (Bengough *et al*., 2011; Valentine *et al*., 2012). The effects of mechanical impedance on root growth can be seen by changes in several traits at the individual root tip scale. Moreover, such changes in growth at the root meristem‐scale have important consequences at the whole root‐system scale. For instance, the RLD distribution may change substantially, especially if a compacted layer limits the maximum depth in the profile to which roots can penetrate. A shallower root distribution may limit crop yield if sufficient water and nutrients are not available within the smaller volume of soil explored by the roots.

Individual root tips undergo morphological changes as a result of mechanical stress, with thickening of root diameter, shortening of the hairless zone of bare root tips, and sometimes distortion of the shape of the root tip (Atwell, 1990; Schmidt *et al*., 2013; Colombi *et al*., 2017). Root tip morphological changes underlying root responses to mechanical stress have been reviewed thoroughly by Potocka and Szymanowska‐Pułka (2018). It is often unclear whether morphological changes in root tip growth are merely a consequence of mechanical stress, or an adaptation that will confer benefit for the root tip in terms of soil penetration. For example, species with thicker diameters may be better able to penetrate compacted soil layers to depth in the field (Materechera *et al*., 1992), possibly due to a decrease in the peak mechanical stress at the tip of the root apex (Kirby & Bengough, 2002; Bengough, 2012), and possibly due to increased bending stiffness of thicker roots enabling roots to penetrate localised compacted layers (Clark *et al*., 2008). Quantitative trait loci associated with penetration of hard wax layers have been successfully identified in screening studies on rice (Price *et al*., 2000), suggesting this is a heritable trait.

We describe four quantitative traits measured on root tips: elongation rate as well as root tip diameter, length of the apical nonbranching zone, and length of apical hairless zone, the latter three providing complementary information on root elongation. Addressing root elongation is a major issue in modelling of the RSA and more generally in root ecology studies, because it represents an important investment of plants to fulfil root functions and it contributes to define major root traits at the plant level. For example, rooting depth and root lateral extension mainly depend on the root elongation of main roots, while RLD depends on both elongation and branching rates. Moreover, obtaining direct measurements on the dynamics of root elongation in soils remains very tricky and root elongation is usually measured on windows interfaces, instead of the plain soil.

The traits that we propose can be used either directly to analyse variations among plant genotypes or environmental conditions and to establish relevant relationships with local soil conditions (e.g. Bécel *et al*., 2012; Schmidt *et al*., 2013) or they can be used to estimate the values of parameters included in models dedicated to the simulation of the dynamics of the RSA (Leitner *et al*., 2010; Schnepf *et al*., 2012; Pagès *et al*., 2014; Postma *et al*., 2017). The traits are conceived and measured at the individual root level. However, for various objectives, it is possible to aggregate these data at larger scales or to study the distributions of traits. Simulation models can be used to make such integration and upscaling of analytical data (Pagès & Picon‐Cochard, 2014).

### 1. Root tip diameter

*Root tip diameter* is the diameter of the root in the zone where the root is nearly cylindrical, that is right after the apical cone (typical units: mm) (frequent abbreviation: Drt).

This diameter, often called apical diameter, is firstly an indicator of the amount of biomass which is required to elongate the root, as biomass per unit of length is proportional to the squared diameter, for a cylinder‐shaped root with a given RTD. Moreover, the diameter of the root tip was often shown to be positively related to root elongation (Wilcox, 1968; Wilson & Horsley, 1970; Coutts, 1987; Cahn *et al*., 1989; Pagès, 1995; Lecompte *et al*., 2005; Bui *et al*., 2015). At the individual root level, correlations were obtained between this diameter and elongation rate or length in many different species, including gymnosperms and angiosperm trees and herbaceous plants belonging to various families. These empirical relationships were generally interpreted considering that apical diameter is intimately related to the size of the apical meristem (e.g. Barlow & Rathfelder, 1984). As this size is approximately proportional to the number of meristematic cells, it is logically an essential determinant of elongation rate, at least in uniform environmental conditions. Pagès (1995) and Thaler & Pagès (1996, 1998) discussed this important general relationship and proposed to use it to model the potential elongation rate of each individual root as a function of its apical diameter.

Thickening of roots in response to mechanical stress is also associated with slower root elongation meaning that, although roots may successfully cross strong layers of soil, the rate of growth to depth may also be slowed (Bengough *et al*., 2006). Certain root tip responses to mechanical impedance, although potentially beneficial for root penetration, currently can only be measured in small controlled experimental systems. These include direct measurement of root forces (e.g. Bizet *et al*., 2016; Clark *et al*., 1999), increased exudation rates associated with mechanical impedance (Barber & Gunn, 1974; Boeuf‐Tremblay *et al*., 1995), and the rate of border cell production (Iijima *et al*., 2003). Most studies investigating root responses to mechanical stresses have been in small controlled systems using seedlings, largely because soil strength depends strongly on soil water content, decreasing following rainfall and increasing greatly as the soil dries (Bengough *et al*., 2006).

Not only elongation rate but also growth duration and longevity were shown to depend on apical diameter (Cahn *et al*., 1989; Varney *et al*., 1991; Eissenstat *et al*., 2000). Within the whole root system, the finest roots usually have a determinate growth pattern, with an elongation activity lasting only a few days (e.g. Varney & McCully, 1991). The relationship between growth duration and diameter has been studied for example by Cahn *et al*. (1989) for maize, by Eissenstat *et al*. (2000) for fruit trees and by Pagès (1995) for young oak trees. In maize and oak, elongation of the finest roots lasted less than 3 d. Conversely, the thick roots (sometimes referred to as pioneer roots or macrorhiza) have a long‐lasting growth, some of them exhibiting an indeterminate growth pattern. Thanks to their rapid growth rate and indeterminate growth pattern, these thick roots extend the colonised volume of soil.

Additionally, root apical diameter, as representative of the size of the meristem, was shown to be a predictor for the intensity of the gravitropic behaviour for several species among both dicots and monocots (Coutts, 1987; Le Roux & Pagès, 1996; Jourdan *et al*., 2000; Wu *et al*., 2015). Wider meristems typically showed quicker reorientations than thinner ones. Such relationships between the tip diameter and gravitropic intensity have been observed within species, but they should not be used for interspecific comparisons. A marked gravitropism for the thick roots can be seen as a useful and consistent feature to achieve their role of root system extension towards given directions (depth, horizontal direction).

Therefore, the diameter of the root tip is both a prime component of the root economy, and it is an essential trait to predict individual root growth attributes, especially elongation rate, growth duration and eventually final length. However, these relationships between diameter and growth attributes are different – and therefore must be calibrated – for the different species and environmental conditions. Moreover, both the range of apical diameters and average diameters exhibit strong interspecific variations (Gu *et al*., 2014; Kong *et al*., 2014; Pagès, 2014, 2016; Valverde‐Barrantes *et al*., 2015, 2017; Pagès & Kervella, 2018).

#### a. Sampling recommendations

See section **VI. Experimentation and sampling in laboratory and field**. The main difficulty for measuring the traits on root tips is to harvest the roots very carefully, because root tips are the most fragile parts of the roots. In some species, they are brittle and easily snap. In pot experiments, this harvest can be much easier, providing that the soil or substrate can be gently separated from the roots (see section **VI. 2. b. Substrate types**). In the field, we recommend to extract monoliths with sufficient dimensions (at least 20 cm wide). Soil cores obtained by augers are more convenient and quicker to extract, but they are not suitable for these observations because they are too small. The probability of obtaining intact root tips decreases a lot with decreasing size of the soil sample, and also if the sample edges have to be twisted a lot to enable extraction.

#### b. Storage and processing

See section **VII. Root washing, sorting and storage**. Another important step is washing the roots from the embedding soil. To maximise efficiency and minimise damage to the root tips, the samples should be immersed in large baths of water, over finely meshed sieves for periods of up to several hours. Combining the use of salt and soap in the bathwater, as well as maintaining a gentle water movement, greatly facilitates the washing process. Salt and soap synergistically disperse the soil aggregates; a difficult task if the soil contains clay and organic matter. Neither should be used if chemical analyses of root tips (e.g. N concentration) are also planned.

#### c. Measurement procedure

**Fig. 28:**
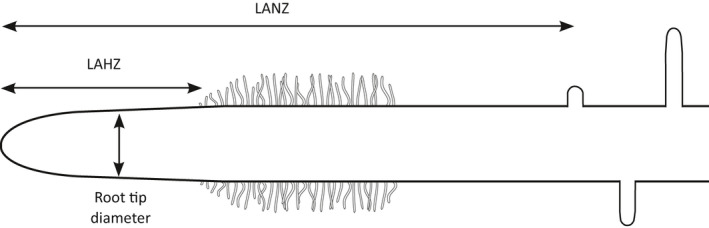
Schematic view of a root tip and suggested measurements. LANZ, length of apical nonbranched zone; LAHZ, length of apical hairless zone. Redrawn from Pagès *et al*. (2010).

Root tip measurements (Fig. 28) can be made using a binocular microscope or a desktop scanner together with an image analysis software.

##### Binocular microscope

A binocular microscope with eyepiece graticule and a magnification factor of *c*. ×30 can be used. The calibration of the graticule scale using a stage micrometre or similar, should always be checked at the start of each series of measurements. Alternatively, excised root tips may be placed on the square grid of either an 'England Finder' microscope slide, or a piece of damp graph‐paper, and the diameter of the root measured at 0.5 mm or 1 mm intervals along the root tip. It is important to avoid drying the root tips by misting with water from a hand‐sprayer at regular intervals and keeping roots on damp blotting paper under a polythene sheet between measurements.

##### Imaging at high resolution

For imaging, roots are spread in a film of water contained in a transparent plastic or glass container. Using a scanner with transparency mode is strongly recommended. The transparent mode of the scanner allows seeing the root hairs as well as internal structures in the root, depending on the transparency of roots. The sharpness and contrast of obtained images are decisive for subsequent measurements. The resolution must be sufficient (usually between 1200 and 3200 dpi) depending on the fineness of roots. Compressed image file formats (e.g. jpg) are convenient to keep a high level of detail while reducing the file size, however they are not as precise as noncompressed formats (e.g. tiff). Working with grey level images is useful to limit their size. Nonetheless, sometimes, more detailed colours (coded on 24 bits) can be useful to measure particular traits, but images are slower to process when pixels are coded using a larger number of bits.

Quantitative observations can be undertaken with various pieces of software. For example, imagej (http://rsbweb.nih.gov/ij/) is a free and open‐source software that is very convenient to make a large panel of measurements (e.g. diameter, length, surface and colour). With ImageJ, the ‘straight line’ tool can be used to measure root tip diameter.

##### Selecting the healthy root tips

It is easy to visually evaluate the quality of root tip images under a binocular microscope or on a computer monitor, and to evaluate the state of the sampled root tips, to select healthy root samples, that is those which were growing or just recently stopped. At this stage, it is easy to identify and discard those which are broken and those which are degraded by the extraction procedure or by the normal processes of ageing and necrosis into the soil. When the sampled roots have grown in dense soils, they usually exhibit the mechanical constraint by local thickenings accompanied by high densities of root hairs (Konôpka *et al*., 2009; Bengough *et al*., 2011). These zones of local thickening must be noted separately as it is an indication that soil strength is limiting the elongation of these root tips, and causing an increase in their diameter.

### 2. Root elongation rate

*Root elongation rate* is the length of root produced per unit of time (typical units: mm^−1^ d^−1^).

*Length of apical nonbranched zone* is the distance from the apex to the most distal lateral root longer than 0.5 mm (typical units: mm^−1^) (frequent abbreviation: length of apical nonbranched zone (LANZ)).

*Length of apical hairless zone* is the distance from the tip of the apex to the base of the closest emerging root hairs (typical units: mm) (frequent abbreviation: length of apical hairless zone (LAHZ)).

Root elongation involves cell division in the apical meristem and cell elongation in its proximal vicinity. The apical generation of new cells has the important implication of creating an age gradient along the roots, with the youngest cells being at the distal end and the oldest at the proximal base. Some functional properties of the roots are tightly linked to this age gradient, and so to the longitudinal position along the root.

Within the root system, elongation activity is highly variable from one root to the other in both intensity and duration, leading to considerable differences in length between the roots. Some roots exhibit an indeterminate growth pattern during the whole growing season and therefore define the root system extension, while a large majority are determinate and stop growing after a period of some days. Even on annual plants, such as sunflower, pea, maize or rice, the indeterminate roots (also called pioneer roots, main roots, framework roots or macrorhiza) are often longer than 1 m, while the shortest roots reach only a few millimetres. The usually large number of determinate roots (also called short roots or brachyrhiza) makes their contribution important to define total root length and distribution of RLD. Therefore, the measurement of root elongation rate must be done on well defined root categories (see section **IV. Below‐ground plant entities and root classifications**).

In most species, the indeterminate roots, have the important role of developing lateral roots. This process is usually organised temporally and spatially, since most lateral roots are initiated near the apex, develop into lateral primordia during several days, and eventually emerge as lateral roots. This organisation of branching has been called ‘acropetal branching’, because laterals tend to appear towards the apex. Several authors have observed a tight positive correlation between the LANZ (distance from the apex to the distal most) and the elongation rate of the mother root (Pagès & Serra, 1994; Pellerin & Tabourel, 1995; Lecompte *et al*., 2001). This relationship stems from the fact that lateral root initiation is very close to the apex and lateral root emergence appears on tissues having a relatively fixed age (A_e_) within a given species. Typical values of A_e_ range between 3 and 6 d, depending on species (Pagès *et al*., 2010). Therefore, the LANZ reflects the elongation of the mother root during the period of time 0–A_e_. It could be used (Pagès *et al*., 2010; Schmidt *et al*., 2013) as a trait to measure the root elongation during this period (with a mean elongation rate given by the value LANZ/A_e_). Therefore, the location where laterals emerge serves as a landmark along the developmental sequence of the root tip. The trait LANZ can be valuably used on roots which potentially have a long‐lasting elongation and a regular acropetal branching. Providing that the relationship between LANZ and elongation rate has been calibrated to determine the A_e_ value, this trait gives a simple way to estimate elongation rates in soil conditions. However, the trait LANZ cannot be used on the finest roots of the studied species, because their growth is determinate and their branching pattern is usually more erratic.

Following the same principle as for LANZ, the LAHZ (also referred to as length of hairless root tip or distance between apex and root hairs) of excavated primary roots from both laboratory and field has been found to correlate linearly with the root elongation rate in cereals (Watt *et al*., 2003; Pagès *et al*., 2010; Schmidt *et al*., 2013). It is a complementary trait which gives a more instantaneous estimate of elongation rate than LANZ, typically during the last one or two d before sampling. Similar to LANZ, this trait is typically measured on pioneer roots. This trait can be used when sufficient root hairs are systematically produced by the considered species under the prevailing conditions. As root hairs are particularly fine structures, very precise and somewhat tedious observations are necessary for evaluating the trait. It can be done using a binocular microscope or on high quality scanner images. Similar to LANZ, LAHZ can vary from one species to the other and therefore must be calibrated for each species. Additionally, LAHZ can be used in a simpler qualitative way, to distinguish the roots that were stopped from those that were growing. The stopped roots exhibit root hairs very close to the tip, and sometimes root hairs even cover the very tip.

The measurement of LANZ and LAHZ makes it possible to study the elongation rate variations in the soil, in relation to several factors, such as the diversity of roots in single root systems, the phenological stage of the plants, or the local soil characteristics around the investigated roots. Measurements of these traits on different types of roots will bear different outcomes. In cereals there may well be differences in the growth and stress responses of seminal and nodal roots, and even between nodal roots that emerge from different whorls (Gregory, 1986). Focusing on pioneer roots is particularly useful to characterise the penetration of the root system to depth, while the focus on absorptive roots would give indication on the capacity of roots to respond to fluctuating resource availability (e.g. Eissenstat *et al*., 2015). As root tip morphology is strongly influenced by soil mechanical strength and other soil physical properties, measurements of penetrometer resistance, water content at field capacity, and soil dry bulk density are particularly important to characterise the context of these trait measurements (see Smith & Mullins, 1991). Indeed, one of the root responses to mechanical impedance is a decrease in the rate of cell expansion of cells in the elongation zone that are furthest from the root tip (Bengough *et al*., 2006).

#### a. Sampling recommendations

Approaches to study or estimate root elongation rates can be divided into three main groups; direct observations and indirect approaches. Direct observations are techniques in which the researcher directly views or images individual roots in the soil environment. The most common direct approach today is the minirhizotron technique which uses clear tubes installed into the soil through which the soil environment and roots may be repeatedly imaged overtime using a scanner or camera. Indirect approaches involve the excavation of roots and the measurement of morphological variables on the root tips. Regarding the indirect approach, we consider both *in situ* sampling and experimental manipulation and control of soil conditions for root growth.

##### Minirhizotron

See section **VI. 3. b. Overview of root observation methods**.

##### *In situ* sampling

See section **XXI. 1. Root tip diameter**.

##### *Ex situ* experimentation

Plants can be grown in tubes of sieved and repacked soil, or in soil that has been sampled from the field by pushing the tube into the soil. Roots are grown for periods of time ranging from a single day to several months, depending on the size of the tube. It is crucial that the initial packing of the soil is controlled carefully. To provide a uniform growing environment the soil should be sieved when relatively dry (easiest when air dry), normally to < 2 mm, to provide a uniform environment at the root tip scale. Soil should then be rewet to a known water content where the soil will stick back together (generally slightly drier than the plastic limit). The soil should be equilibrated in a sealed double‐wrapped polythene bag for 24 h in the dark, and then repacked by compressing the soil in layers of appropriate thickness while scarifying each layer surface to create a column of as uniform density as possible. To apply the compressive stress it is usual to either compress to a known dry mass per unit volume using a hydraulic press or ram, or to pack with a constant energy using a proctor hammer. If a tube longer than *c*. 20 cm is being used, then it is often helpful to use a thin polythene liner to aid withdrawal of the soil and root system at the end of the experiment, or to grow roots in a tube that has been split vertically and can be opened.

If the growth period is only 1 or 2 d, with a nontranspiring seedling, then the tube may be simply covered with polythene and kept at constant temperature in a growth room. For longer periods it will be necessary to maintain the water content of the tube to be as constant as practicable. If the soil cores are more than 10 cm deep, surface watering may lead to a significant gradient in water content along the tube, and therefore variation in soil strength. Helpful approaches might include watering daily to prevent the soil hydraulic conductivity decreasing, bringing the whole tube back to approximately field capacity by immersing it once a week, or watering along the tube by injecting water or using a porous wick of some form.

#### b. Storage and processing

Root tips will continue to grow after extraction from the soil, with elongation rates of up to 1 mm h^−1^ being typical of intact crop plants at 20°C. This may mean that the root tip properties become determined by conditions after harvest, rather than when the root was growing encased in soil, and so it is important to stop the growth of the roots by excising them and chilling to between 1 and 4°C, potentially with the use of a preservative to kill the roots and prevent microbial growth. Ethanol is a commonly used and recommended preservative for morphological analyses at a concentration of > 50% (see section **VII. Root washing, sorting and storage**). Excised root tips may be stored conveniently in Eppendorf‐style tubes. Root tips stored in preservative may be kept safely in the fridge for a period of weeks before analysis and it may be appropriate to transfer roots back into distilled water before measurements are made.

#### c. Measurement procedure

##### Minirhizotron

See section **VI. 3. b. Overview of root observation methods**.

##### *In situ* sampling and *ex situ* experimentation

See section **XXI. 1. Root tip diameter**. The use of an image analysis software on scanned images is particularly recommended if root tips are distorted such that the distance along the centreline of the root must be measured.

The LANZ and LAHZ are measured as the length from the apex to the most distal lateral root longer than 0.5 mm and the length from the tip of the apex to the base of the closest root hair, respectively (Fig. 28). *Ex situ* measurements, by incubating roots for a known amount of time also allow direct estimations of root elongation rate. This is made by subtracting the initial root length from the root length at harvest and expressing it per unit time.

#### d. Future research directions

When using LANZ or LAHZ to estimate root elongation rate, it is assumed that the age of the mother root tissues on which lateral roots emerge or root hairs elongate is fixed. To date, this assumption has been evaluated in a limited number of situations (Lecompte *et al*., 2001; Pagès *et al*., 2010; Schmidt *et al*., 2013) and additional studies on other species and conditions are required to extend this verification.

As we need to better understand root growth in field soil where root growth is often limited by mechanical strength in macro‐porous heterogeneous environment, comparing root elongation rates across a range of soil densities would be a powerful tool to compare the capacities of different root species and genotypes to penetrate soils and improve soil physical properties.

Laser ablation is now enabling rapid phenotyping of traits related to cellular morphology in roots, although most studies have been performed on older tissues far behind the root apex (e.g. Chimungu *et al*., 2015). The decreasing cost and increased resolution and processing speed of X‐ray microtomography systems has also great potential for studying root growth *in situ* with the ability to resolve root hairs in X‐ray synchrotron imaging of roots in narrow tubes of soil (Koebernick *et al*., 2017). However, although X‐ray microtomography can quantify the complex patterns of macropores present in field soil (Jassogne *et al*., 2007), we need to further develop methods to resolve and track the growth of relatively fine‐root material in large cores of heterogeneous field soils.

## 22 Root hair morphology and development

**Fig. 29:**
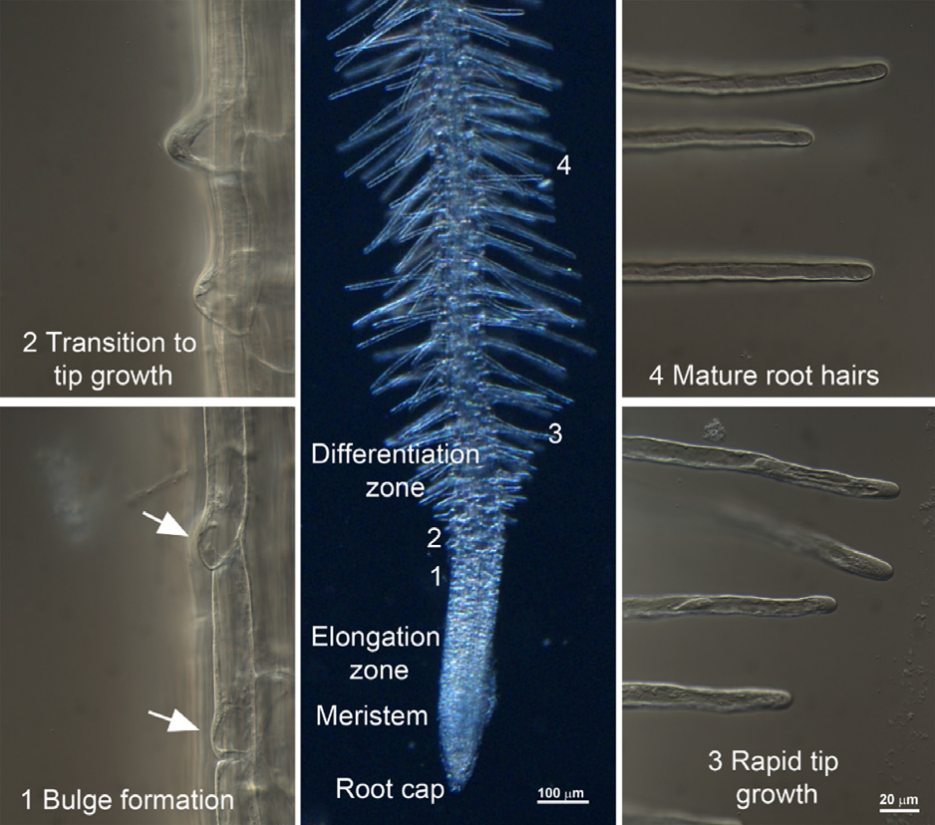
Stages of root hair development in *Arabidopsis thaliana*. Root hairs emerge in the root differentiation zone (numbers 1–4 of the middle panel) and progressively increase in length the further they are from the root cap. Numbers in the outer panel are high magnification differential interference contrast micrographs of root hairs corresponding to the numbers in the middle panel.

Root hairs are tubular projections that form on epidermal cells of the root that improve plant nutrient and water uptake. Although root hairs typically emerge in the root differentiation zone (Fig. 29), the fate of the cells that give rise to root hairs is already specified at an early stage of plant development (i.e. during embryogenesis and post‐embryonic root growth) (Schiefelbein *et al*., 2009). Our understanding of the molecular and cellular bases underlying root hair development has come mostly from studies of the model plant *Arabidopsis thaliana* (Bibikova & Gilroy, 2002; Grierson *et al*., 2014). In *Arabidopsis*, a suite of transcription factors interacting within a network of positive and negative feedback loops specifies whether a particular root epidermal cell forms a root hair or remains hairless. This process of root hair cell specification also relies on continuous positional cues provided by underlying cortical cells as the root develops (Schiefelbein *et al*., 2014). The situation is different in some grasses such as *Brachypodium distachyon*, *Hordeum vulgare* and *Oryza sativa*. In these species, root hair cell specification also appears to involve developmentally programmed symmetric or asymmetric epidermal cell divisions (Marzec *et al*., 2013, 2015; Dolan, 2017).

Although the genetic circuitry and positional signalling cues that instruct a root epidermal cell to form a root hair differs among plant species (Wang & Schiefelbein, 2014; Dolan, 2017), the molecular machinery that regulates the later stages of root hair development is highly conserved (Grierson *et al*., 2014). After emergence, root hairs attain their cylindrical shape via tip growth, a process that involves the targeted trafficking of membranes and vesicles carrying cell wall building blocks to the cell apex (Rounds & Bezanilla, 2013).

The utility of root hairs for basic plant genetics and cell biology research is well established (Bibikova & Gilroy, 2002; Grierson *et al*., 2014; Bascom *et al*., 2018). However, their importance as a target trait for plant breeding and ecological applications is only beginning to be realised. Early numerical simulations of the influence of root hairs on soil resource uptake focused on the relatively immobile nutrient phosphate. They demonstrated that root hairs increase the effective radius of the root, therefore allowing access to phosphate beyond the depletion zone of the root surface (Itoh & Barber, 1983). This conceptual model continues to guide root hair research, and is supported by work in trees showing an inverse relationship of mycorrhizal and root hair abundance among species that indicates root hairs and mycorrhizas may have some functions in common. Baylis (1975) proposed that among tree species, those with a greater reliance on mycorrhizas constitutively express lesser degrees of root hair density and length. This hypothesis was validated in a set of tropical trees (St John, 1980). Given the known importance of mycorrhizal hyphae for exploring soil for access to phosphorus and water, this inverse relationship in trees provides support for the importance of root hairs for accessing the same resources as mycorrhizal hyphae. It is now increasingly recognised that root hairs have substantial influence not only on phosphorus, but also nitrogen and water uptake (Robinson & Rorison, 1987; Carminati *et al*., 2017b).

In addition to their roles in soil resource uptake, root hairs are an important driver of rhizosheath and rhizosphere formation (York *et al*., 2016). Scanning electron microscopy revealed the intimate association of soil particles with root hairs (Hinsinger *et al*., 2003). The soil particles are further agglutinated by secretion of mucigel from both roots and microbes, which along with a mesh of root hairs leads to the formation of a rhizosheath that is often visible when roots are removed from bulk soil (James *et al*., 2016). While the rhizosheath may be viewed as the component of the rhizosphere most physically associated with roots and root hairs, the rhizosphere can extend several millimetres into the soil due to exudation of hundreds of chemicals, uptake of nutrients and water, influences on gas concentrations and soil processes such as diffusion, ion exchange, and microbial activities (York *et al*., 2016).

The location of root hairs along the root surface makes them highly amenable to microscopic observation allowing researchers to ask a range of basic questions on mechanisms underlying polarised growth in plants, cell fate determination, cellular signalling and organelle/membrane dynamics (Grierson *et al*., 2014). Nevertheless, the root hair traits that are most valuable for plant breeding applications and studies linking root hair functions with plant performance are root hair length and density (Brown *et al*., 2013). Additionally, root hair rhizosheath size is increasingly thought to adequately represent the overall influence of root hairs, particularly in acidic soils (Delhaize *et al*., 2012; Delhaize *et al*., 2015; James *et al*., 2016), and root hair lifespan is critical to assess the temporal scale of influence of root hairs (Shane *et al*., 2011; Brown *et al*., 2013).

### 1. Root hair length and root hair density

*Root hair length* is the length of fully grown root hairs (typical units: µm or mm).

*Root hair density* is the number of root hairs per unit root length (typical units: mm^−1^). It can also be expressed per root surface area (mm^−2^).

Root hair length and density are generally considered as two complementary facets of the same root functions, therefore we propose studying them as a functional module (York *et al*., 2013). Greater root hair length generally extends the zone of influence of the root in the soil and its reach to less mobile or spatially isolated soil resources, whereas root hair density increases the intensity of the root hair effect on the same aspects. For example, in simulation studies, increased root hair length and density increased the total depletion volume, but increased density eventually leads to substantial competition among root hairs and decreased phosphorus acquisition efficiency (Ma *et al*., 2001b). This efficiency will be especially important if root hairs have substantial construction or maintenance costs, yet empirical studies have not discovered such an investment cost (Bates & Lynch, 2000). Given these interactions of root hair length and density, some studies have explicitly combined them into one trait such as the root hair factor (RHF), which is derived by multiplying length and density (Nestler *et al*., 2016).

Root hairs have been shown to proliferate in conditions of low phosphate availability (Ma *et al*., 2001a; Zhu *et al*., 2005), and their presence can offer a competitive advantage in low P conditions (Bates & Lynch, 2001). Therefore, the case for the utility of root hairs in phosphorus uptake is strong (Heuer *et al*., 2017). The influence of root hairs on nitrogen uptake is somewhat less clear. Nitrate is rather mobile in soil so root hair length is usually considered a minor adaptation to nitrogen shortage (Forde & Lorenzo, 2001). A few studies reported nonetheless common positive responses of root hair length to nitrogen deficiency among herbaceous species (Boot & Mensink, 1990; Freschet *et al*., 2018) and demonstrated a possible link with increased plant growth (Robinson & Rorison, 1987).

Regarding water, older work using microphotometers, demonstrated that root hairs are capable of water absorption (Rosene, 1943). This role in water uptake is partially confirmed in studies using magnetic resonance imaging and numerical modelling (Segal *et al*., 2008). Recent conceptual work suggests that root hairs may support liquid bridges by connecting roots across soil pore air space to films of water bound to soil particles (Carminati *et al*., 2017a), which would also give more access to nutrients dissolved in that water. Further empirical work led to a proposal that root hairs reduce the sharp decline of matric potential near the root epidermis and therefore prevent hydraulic failure (Carminati *et al*., 2017b). More research is needed to fully understand the role of root hairs in water uptake.

In common bean, root hair length and density were shown to positively correlate to acid (H^+^) exudation in a segregating mapping population which consists of related individuals sharing the same two parents and often displays substantial phenotypic variation for traits of interest (Yan *et al*., 2004). By comparing wild‐type barley with mutants lacking root hairs, Holz *et al*. (2018) demonstrated that the wild‐type plants had a greater carbon deposition, consistent with the release of carbon‐based exudates by root hairs. Last, due to root hairs bringing roots in intimate contact with the soil, root hairs aid plant anchorage and penetration of high‐strength soil (Haling *et al*., 2013).

Root hair measurements in annual species have typically been made on axile roots (e.g. tap, basal or shoot‐borne roots). However, a mature annual root system usually includes second‐order or third‐order lateral roots. These lateral roots have to be considered when making root hair measurements to better evaluate the contribution of root hairs at the whole root system scale. In fact, limiting measurements of root hair length and density to the axile roots in rice showed that these parameters can lead to overestimation of root hair influence on phosphate uptake, especially if data from nutrient solution‐grown plants is extrapolated to the field (Nestler *et al*., 2016). Conversely, in perennial species, measurements of root hairs have focused on stream‐based first‐order or fine roots (those roots most topologically distal from the plant stem) that are believed to be more active in nutrient acquisition. Therefore, we would recommend sampling several root orders, classes, and locations based on distance from root tip to develop informed protocols for a particular species and experiment.

#### a. Sampling and experimental recommendations

See section **VI. Experimentation and sampling in laboratory and field**. For plant breeding applications, several methods for high‐throughput root hair measurements have been developed using crop plants such as wheat, rice, barley and soybean. These methods include growing plants on germination paper, gel‐based media, hydro/aeroponics and sand : vermiculite mixtures in containers (Delhaize *et al*., 2012; Qiao & Libault, 2013; Dyachok *et al*., 2016; Horn *et al*., 2016; Nestler *et al*., 2016). As for germination paper, hydroponics, aeroponics or gel‐based media protocols require minimal washing of the roots to acquire images of root hairs, enabling more rapid sampling (Qiao & Libault, 2013; Horn *et al*., 2016). One could argue, however, that these artificial systems might not accurately reflect root hair traits under field conditions (Nestler *et al*., 2016). As such, screening methods in artificial substrates can be complemented with measurements of root hairs from plants grown in containers or rhizoboxes containing real soils (Nestler *et al*., 2016). In the field, extraction of roots using shovels or soil corers should be done with the greatest care. A disadvantage of screening plants grown in real soils is the increased collapsing and breaking of delicate root hairs, leading to underestimation of root hair length and density.

**Fig. 30:**
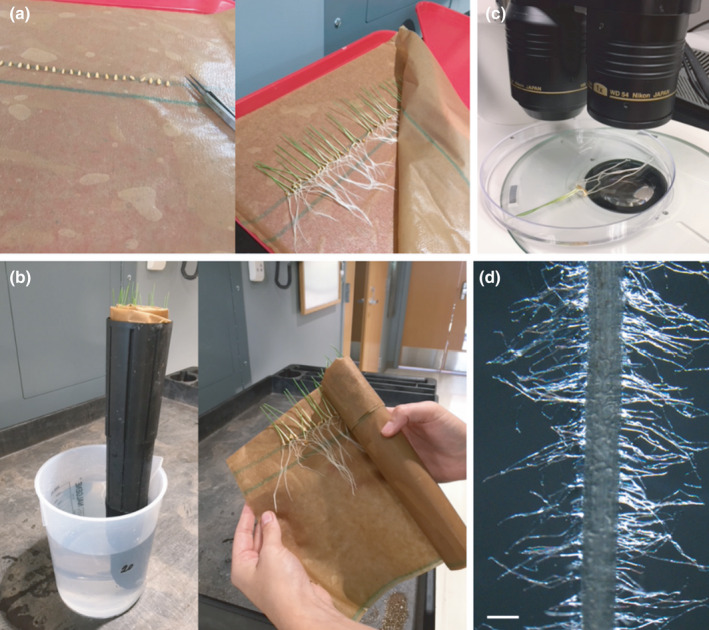
Protocols for phenotyping root hairs in a large number of plants. (a) Imbibed wheat seeds are aligned in a row on moist germination paper placed on top of a tray; or (b) the paper can be rolled and inserted into cones. (c) After 10 d of vertical growth, root hairs are ready to be imaged with a stereomicroscope. (d) Representative images of unstained wheat root hairs for length and density measurements. Bar, 500 µm.

**Fig. 31:**
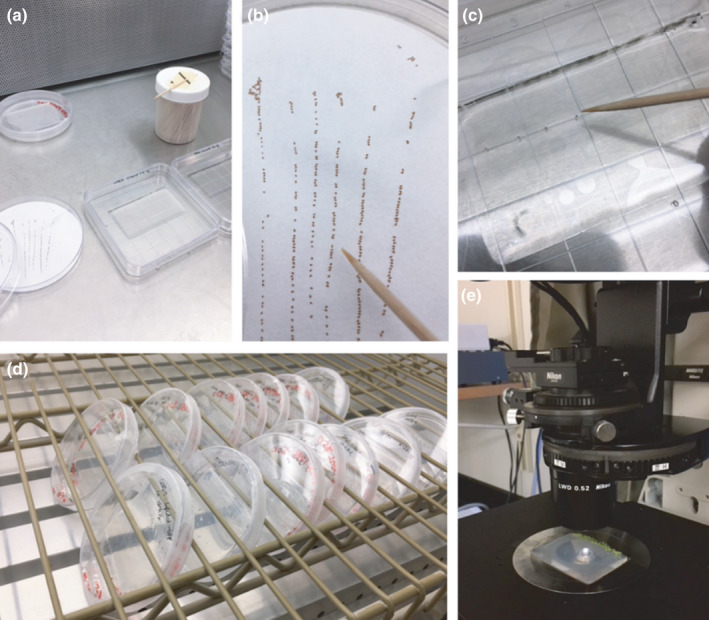
Protocol for preparing *Arabidopsis* seedlings for live root hair imaging. (a) All material for handling seeds is sterilised and prepared in a laminar flow hood. (b, c) Sterilised seeds are picked individually with a pointed toothpick and planted on nutrient‐supplemented gel layered onto a coverslips. (d) Coverslips with seeds are placed inside Petri dishes and kept at an inclined angle in a growth chamber. (e) After 3–5 d, the coverslips with seedlings can be transferred directly onto a stage of a microscope for image acquisition.

For methods using germination paper, seeds are first imbibed in water for a few hours to overnight depending on the plant species. The harder the seed coat and the larger the seed, a longer imbibition time might be needed. Species with harder seed coats will also require chemical or physical scarification. After seed imbibition or scarification, germination paper is placed on a flat surface such as a cafeteria tray and the paper moistened with water. Seeds are then planted by aligning them in a row over the moistened paper (Fig. 30a). After planting, a second layer of germination paper is used to cover the seeds. Seeds are secured by pushing down gently on the germination paper used to cover the seed. Several trays containing the planted seed can be stacked vertically or germination paper can be rolled and secured in a cone (Dyachok *et al*., 2016). In both cases, trays or cones are positioned vertically on containers with water (Fig. 30b). This will allow moisture to rise and prevent germination paper from drying while allowing roots to grow down toward the gravity vector. Although such a set‐up will keep paper moist, it is important to monitor the status of the paper as the seedlings develop. Depending on the plant species, seedlings are ready for root hair measurements when axial roots are *c*. 3‐10 cm long. In wheat and maize seedlings, for example, the suitable axial root length in which root hair measurements can be done range from 3–6 d after germination. Alternatively, a gel‐based method using cut cellophane discs placed on top of the gel can be used to phenotype seedling root hairs in square Petri plates. Surface‐sterilised seeds are planted on the cut edge of the cellophane, plates sealed with Micropore tape and plates positioned at an inclined angle (see Fig. 31) so the primary roots grow down over the cellophane for subsequent root hair imaging with a stereomicroscope (Horn *et al*., 2016).

#### b. Storage and processing

See section **VII. Root washing, sorting and storage**. To wash roots free of soil, we recommend carefully soaking roots in dish detergent until soil softens and begins to fall away, and then gently wash to remove remaining soil. These washed roots can either be imaged immediately as described below or stored in ethanol (concentration > 50%) in a refrigerator (4°C) for months.

#### c. Measurement procedure

Images of unstained (Fig. 30c) or toluidine blue‐stained (Vejchasarn *et al*., 2016) root hairs are acquired using a stereomicroscope. The staining step is performed by dipping the root for a few seconds into the colourant (e.g. 0.5% toluidine blue) followed by a series of water rinses. This step increases contrast in stereomicroscope systems that do not have the optics to enable clear visualisation of unstained root hairs. A minimal magnification of ×40 enables the distinguishment of the base of the root hair from its tip, which is important for accurate length measurements. Downstream length measurements are made in ImageJ using the ‘segmented line’ tool (Schneider *et al*., 2012). For density measurements, the number of root hairs that occupy a known area of the root surface (e.g. on the visible half of the root) can be manually counted from digital images.

The number of measurements for obtaining reliable root hair trait values also depends on whether plants were sampled in the field or grown in the laboratory. Because environmental conditions in laboratory‐grown plants are better controlled, fewer measurements are needed. For live root hair measurements in *Arabidopsis* growing on gel‐based medium, counting in total 200 root hairs from 5–10 independent seedlings (*c*. 20 root hairs per seedling) is usually sufficient (Kwon *et al*., 2018). For field‐grown plants, the more root hair measurements acquired the better. We recommend measurements of 50 root hairs from at least three regions within one root sample. It is also important to carefully record the root type from which root hair measurements are taken to get a more accurate representation of root hair contributions at the whole root system level (Nestler *et al*., 2016).

Root hairs emerge from the root differentiation zone and this process is strongly influenced by the environment (e.g. water and nutrient availability, soil type/compaction) and plant species. In *Arabidopsis* seedlings grown in gels, root hairs emerge at *c*. 300 µm from the root tip (Fig. 29). Maximum root hair length is typically reached at *c*. 500 µm to 1 mm from the root tip. Therefore, to obtain information on root hair length and density of fully grown hairs, measurements should be obtained a few millimetres away from the root tip, generally along the region of the root in which root hairs have attained their maximum length.

Although most of the protocols for acquiring root hair measurements from live plants have been developed for *Arabidopsis*, they should be applicable for other plant species after a few refinements. Moreover, these protocols are not only applicable to obtain measurements from root hairs but can also be used for studies on the primary root (Srivastava *et al*., 2011). Using these protocols, the type of measurements that can be acquired include root hair growth rate, root hair length and a range of cellular processes such as tip‐focused signalling events (e.g. cytoplasmic calcium) and organelle dynamics (Kwon *et al*., 2018).

#### d. Future research directions

More needs to be known about the potential costs of root hairs in root economics, which may be negligible in terms of resource invested but nonnegligible with regard to other aspects such as root defence against pathogens. Additionally, the roles that root hairs play in not only increasing effective root radius, but also creating hydraulic bridges and rhizosheaths need further refinement, along with the effects of uptake of all soil resources.

Protocols that facilitate removal of soil without damaging root hairs would allow more field work to include this important trait. Imaging methodology that does not require staining and that maintains high contrast between root hairs and background would benefit automated root hair image analysis. In recent years, technology to nondestructively image root hairs in soils has been developed. The most widely utilised *in situ* method that provides sufficient resolution is synchrotron radiation computed tomography (SR‐CT). This technology has been used to acquire 3D images of root hairs in the soil. Such information was then applied to build mathematical models for describing a range of root functions including patterns of phosphate extraction by root hairs and impacts of root hairs on soil structure (Keyes *et al*., 2013; Daly *et al*., 2016; Koebernick *et al*., 2017). While SR‐CT has tremendous potential for understanding root physiology in real soils, this method is still low throughput and therefore has limited utility as a screening tool.

### 2. Root hair development

*Root hair growth rate* is the length of root hair built per unit of time (typical units: µm min^−1^).

*Root hair lifespan* is the time between root hair initiation and its death (typical units: d).

The timing of root hair growth may be an important consideration for the adaptive role of root hairs. For example, the time to emergence after cells elongate following tip growth may determine the location of the first root hairs from the tip (see section **XXI**. **2. Root elongation rate**). This and the extension rate into soil may influence the ability to acquire water and nutrients. Root hair lifespan has been rarely measured. However, simulation results indicate that increasing lifespan can contribute to specific P uptake similar to increasing root hair length, ranging from 0 to 10 d (Brown *et al*., 2013), depending on the assumed diffusion coefficients construction costs, and changes in uptake capacity with age. Further simulation results constrain the usefulness of increasing lifespan because, for phosphate, depletion would be maximised after 4 d and therefore the utility of root hairs greatly reduced (Jungk, 2001). In fact, this may explain the limits observed for root hair lifespan, and if root hairs have a minimal cost, plants may perform better with at least 5 d of root hair lifespan in general. Nonetheless, benefits of longer root hair lifespan may be further prolonged regarding the uptake of more mobile nutrients.

#### Growing plants for accurate analysis

The most important factor to consider when using root hairs for cell biological applications is to ensure their viability during the entire data acquisition session. Root hair development can be divided into four phases: bulge formation, transition to tip growth, rapid tip growth and maturation (Duckett *et al*., 1994; Bibikova & Gilroy, 2002; Grierson *et al*., 2014). Being familiar with these stages of development can inform researchers about the general health and viability of the root hair cell used in data acquisition (Kwon *et al*., 2018). In *Arabidopsis* seedlings, progress of a single root hair can be readily observed as it transitions through these four developmental phases (Fig. 29). However, to be able to routinely obtain reliable measurements during these developmental transitions, a system that minimises excessive handling of the seedlings, especially in small plants such as *Arabidopsis*, is required.

In *Arabidopsis*, one method used for keeping root hairs viable for live imaging is to plant seeds directly on coverslips coated with half‐strength Murashige & Skoog (½MS) nutrient‐supplemented gel‐based media (Fig. 31). Nutrients and sugars (typically 1% sucrose) to support plant growth are mixed with agar (0.5–0.8%) or other gelling agents of choice (Dyachok *et al*., 2016). After autoclaving, the solution of MS salts, sucrose and agar is poured onto the surface of a coverslip before the gel polymerises (Fig. 31a). Sterilised seeds are picked individually with pointed toothpicks and pushed gently into the polymerised gel until the seed touches the coverslip. The coverslip with the planted seed is placed inside a square or round Petri plate and positioned at an inclined angle in a temperature/light‐controlled growth chamber (recommended 23°C/120 µmol m^−2^ s^−1^) before acquiring data (Fig. 31b,c). Placing the Petri plates at an inclined angle will enable roots to grow downward and along the surface of the coverslip to keep many root hairs growing at a single focal plane (Figs 29, 31d). After 4–5 d, seedlings are ready for microscopic observation. The entire coverslip containing the seedlings can be transferred directly onto the stage of a microscope to minimise excessive handling of the fragile seedlings during measurement (Fig. 31e).

Alternatively, some researchers transfer seedlings onto glass‐bottom culture dishes and cover them with a small volume of medium (0.7% agarose + MS salts). To allow the seedlings to recover from handling, culture dishes are kept in a growth chamber for at least one d before root hair measurement (Park & Nebenführ, 2013). Recently, a microfluidic platform was developed to minimise disturbance to the seedlings and keep the root hairs at the same optical plane as the primary root. This way, root hair growth and organelle dynamics can be quantified while preventing drifting of the cell along the *z*‐axis (Aufrecht *et al*., 2017).

#### b. Measurement procedure

There are many types of root hair measurements that can be made on living plants. From the set‐up described above (Fig. 31), one can obtain data on root hair growth rates (µm min^−1^) or growth rate kinetics. To obtain detailed growth rate kinetics, imaging root hairs at ×100–200 with an inverted compound microscope works best (Fig. 31e). Various systems that enable time‐lapse acquisition of root hair images are available. Root hair growth rates can be obtained from time‐lapse movies by measuring the displacement of the root hair tip with imagej at each time point. The interval selected depends on the biological question of interest. For instance, root hairs are known to exhibit oscillatory growth (Monshausen *et al*., 2007). If one is interested in correlating root hair oscillatory growth with dynamics of root hair cell components, then time intervals of 1 s or less should be considered (Kwon *et al*., 2018). If only root hair growth rate is desired, one can readily select actively growing root hairs based on their length (i.e. typically between 50–100 µm) and distance from the root tip. For such measurements, root hair images are acquired at time zero and then after 1 h. The displacement of the root hair tip is measured and divided by the elapsed time. Under a compound microscope, growing root hairs are characterised by having a dense cytoplasm at the tip. In fully grown root hairs, one can see the vacuole protrude to the tip (Fig. 29). For laboratory‐grown plants, acquiring growth rates from 10 independent plants and 20 root hairs per plant should produce reliable values.

#### c. Future research directions

Detailed analysis of living root hairs as they develop is useful for understanding fundamental plant cellular and developmental processes. The protocol on live root hair imaging in gel‐based media described is low throughput and therefore have limited utility for screening a large number of samples. However, this is due to the fact that tools developed therefore far are tailored more toward addressing basic cell biological and genetic questions. Future methodological advances could make this trait valuable for ecological applications. For example, data on the developmental stage of root hairs that are most efficient for water and nutrient uptake is lacking. New genetically encoded sensors for cellular phosphate should help address this problem (Mukherjee *et al*., 2015). Root hairs contribute to secretion of compounds that attract beneficial microbes or add organic matter to soils. Methods to image secretion and cellular dynamics as they occur in soils would be a valuable addition to the live root hair imaging toolkit.

### 3. Rhizosheath size

*Rhizosheath size* is the weight of soil attached to the root after gently shaking off nonadhering soil per unit root length (typical units: g m^−1^).

Recently, the role of root hairs in stabilising the rhizosheath has become more clear. Even after root hair death their cell walls continue to bind soil particles to the root (Shane *et al*., 2011). Rhizosheath size may therefore have consequences for plant water and nutrient acquisition in drying soil, as well as a range of ecosystem properties such as soil interparticle cohesion. Additionally, over the past decade, this understanding has led to measuring the rhizosheath as a proxy for root hair length and/or density, which has been validated in wheat, barley, and several perennial grasses (Haling *et al*., 2010; Brown *et al*., 2012; Delhaize *et al*., 2012; George *et al*., 2014). Here we suggest that any application of the rhizosheath proxy methods first requires a validation by regressing rhizosheath mass against root hair length and density. This validation could be done before large‐scale evaluations, or could be achieved by measuring a subset of the material for root hairs. Most of the studies have used axile roots in annual crop species. Therefore, further work is needed to understand the application in woody species or perennials, on more distal root orders. The method is expected to work in soils with moderate water content, with inconsistent results when too dry or too wet. Rhizosheaths may be harder to form in very clayey soils or in organic soils, limiting the comparability of this trait across soil types.

#### a. Sampling recommendations

See section **VI. Experimentation and sampling in laboratory and field**.

#### b. Storage and processing

If not measured immediately, roots should be stored in plastic bags and kept cold until processing, but no more than a few hours.

#### c. Measurement procedure

Rhizosheath sampling can be conducted on both pot and field‐grown plant individuals (George *et al*., 2014). Once extracted from the soil, roots are gently shaken to let the bulk soil fall away from the roots. In some instances, large soil clumps (e.g. several centimetres wide) may need to be gently loosened by a gentle pressure between two fingers. The root system that remains may be covered in adhering soil, taken as the rhizosheath. The FW of the root system and rhizosheath are determined. Subsequently, roots are gently washed free of soil to not damage root hairs, patted dry with tissue paper, and then the cleaned root system is weighed again. The difference in mass between the sheathed roots and the cleaned roots is taken to be the rhizosheath mass. Whereas some methodologies have skipped the cleaning and reweighing step after finding that the root FW often comprises only 5% of the combined rhizosheath and root mass, which is possibly too inaccurate for general use across a wide range of plant species (Delhaize *et al*., 2015). Similarly, estimating the rhizosheath DW may be advisable to obtain estimates across soils of contrasting water content. This can be done by cleaning the sheathed roots with a small amount of distilled water above containers of known weight and allowing the water to evaporate to estimate the remaining rhizosheath soil DW.

The root length is measured subsequently as described under section **XII. 2. Specific root length**, and the specific rhizosheath mass is expressed as g m^−1^. Validating the relationship of specific rhizosheath mass to root hair length and density is generally accomplished by excising a short root segment at least 2–6 cm away from the root tip from the gently washed roots and imaging under a microscope. Root hair length and density are measured as described under section **XXII. 1. Root hair length and root hair density**. The specific rhizosheath mass is regressed against the root hair measures, and coefficients of determination (R^2^) in the range 0.4–0.8 have been found for the intraspecific variation of the crop species cited above.

#### d. Future research directions

Although good correlations of root hair length (and in some cases density) have been found with rhizosheath size, the additive and synergistic effects of root hair length, density, mucilage production and possibly other parameters are poorly understood. In these protocols, measuring percent carbon or quantifying mucilage within the rhizosheath along with measuring root hair length and density would allow more advanced multiple regression models to be fit to quantify the contributions of each. As a proxy for root hair traits, the utility of the rhizosheath is partially understood, however the rhizosheath may have other additional properties, such as protecting the root from drying soils, which also need consideration.

## 23 Root decomposition

Fine roots are the below‐ground plant organs with the highest production and turnover rates (Eissenstat *et al*., 2000; Gill & Jackson, 2000; McCormack *et al*., 2015a). Furthermore, because roots die and decompose in close proximity to minerals, soil organisms, and soil organic matter (SOM), root‐derived C contributes disproportionately to SOM formation and dynamics (Schmidt *et al*., 2011). For example, in boreal forests, up to 70–84% of the soil C stock may originate from roots and associated mycorrhizal fungi (Clemmensen *et al*., 2013; Kyaschenko *et al*., 2019). In addition, below‐ground NPP is a significant fraction of global NPP, with fine‐root production and turnover equal to *c*. 22% of total NPP (McCormack *et al*., 2015a). Therefore, below‐ground organ decomposition is a major contributor to C and nutrient cycling in ecosystems and predicting soil C and nutrient dynamics requires consideration of below‐ground traits and decomposition processes (Bardgett *et al*., 2014). Yet, despite its importance, decomposition of below‐ground organs is much less studied than that of leaf litter, yielding variable conclusions regarding root decomposition rates and trait control over decomposition (e.g. Silver & Miya, 2001; Hobbie *et al*., 2010; Goebel *et al*., 2011; Sun *et al*., 2013b; Xiong *et al*., 2013; Ma *et al*., 2016).

Decomposition of roots is influenced by broadly similar factors to that of above‐ground litter, including tissue chemistry, soil nutrient availability, soil biota and soil abiotic conditions (Silver & Miya, 2001; T. Sun *et al*., 2016). However, because of their unique morphology, physiology, symbiotic associations and location within the soil profile, roots are likely to show unique relationships with these factors. Furthermore, the contribution of roots to SOM may change with soil depth, because of changes in soil and microbial properties (Bödeker *et al*., 2016; T. Sun *et al*., 2016; Kyaschenko *et al*., 2019), in contrast with leaf‐litter decomposition, which mostly occurs on the soil surface and therefore may be more affected by photodegradation (Austin *et al*., 2016). Evidence is conflicting whether the traits that influence decomposition are similar for leaf litter and roots. Meta‐analyses of decomposition of bulk fine roots indicate that initial concentrations of calcium and N are important controls of fine‐root decomposition (Silver & Miya, 2001; See *et al*., 2019). However, in recent studies of root tips (first‐order roots), aspects of C chemistry (nonstructural carbohydrates and tannins) were the primary litter chemistry drivers of decomposition (Sun *et al*., 2018). Root tannins were also found to influence fungal necromass decomposition (Adamczyk *et al*., 2019a).

Root decomposition is uniquely influenced by endophytic, mycorrhizal, and saprotrophic soil microbial communities (Kohout *et al*., 2018). For example, fungal colonisation, especially by mycorrhizal fungi, can influence decomposition of fine roots by competing with saprotrophic decomposers for resources and by ensheathing roots and therefore affecting their chemical composition (Langley *et al*., 2006; Fernandez & Koide, 2012; Frey, 2019). Mycorrhizal fungi may directly influence measured decomposition rates as they may account for a significant mass fraction of root litter that cannot be separated from dead root tissue. Root‐derived organic matter can be taken up and transformed into microbial residues by decomposing bacteria and fungi and further stabilised through sorption and incorporation into aggregates and other stable SOM forms (Adamczyk *et al*., 2019b). Decomposing root detritus is also important in soil N dynamics, as a source of N from root tissue and the chitin of associated mycorrhizal fungi and because root‐derived organic matter can prime decomposition and release of N from SOM (Schmidt *et al*., 2011); conversely, decomposing roots can be a sink for N (Kyaschenko *et al*., 2019) and contribute to the formation of stable SOM‐N (Adamczyk *et al*., 2019b).

Measuring root decomposition poses numerous methodological challenges related to temporal and spatial heterogeneity. Many of these challenges are similar to those posed by measuring above‐ground decomposition, but some are unique to roots. Root decomposition experiments are usually conducted over short time periods (less than 2 yr, Silver & Miya, 2001; See *et al*., 2019), which may be inadequate to fully characterise what can be a relatively slow process (Berg, 1984; Parton *et al*., 2007; Harmon *et al*., 2009; Freschet *et al*., 2013). Therefore, long‐term studies (at least 4 yr *in situ*) are needed to disentangle various controls on initial vs late stages of decomposition. Isotopes may be used to measure root decomposition over longer time scales (Kyaschenko *et al*., 2019). At the same time, characterisation of decomposition dynamics requires sequential harvests at intervals that are sufficient in number and frequency to allow model‐fitting of decomposition dynamics (Cornwell & Weedon, 2014). Selecting an incubation period and sequence of harvests that match the natural seasonality of litter production and climate is necessary to accurately characterise seasonal patterns and annual rates of decomposition (Quested *et al*., 2005). For example, in environments with strong seasonality and slow decomposition rates, it is important to include 1‐yr incubation time steps to encompass the full suite of the environmental conditions occurring throughout the year. Most ecosystems experience strong spatial (horizontal and vertical) heterogeneity in temperature and precipitation, in the quantity and quality of litters both on the ground surface and at different depths in the soil profile below‐ground, and in the composition of their decomposer communities (Schmidt & Lipson, 2004; Bardgett & Wardle, 2010). Establishing replicate blocks that allow characterising both within and among site variation, is therefore important to adequately represent ecosystem complexity (Bradford *et al*., 2017).

Measuring root decomposition is also difficult because of heterogeneity within root systems. In decomposition studies, fine roots are often treated as a single category, traditionally defined by their diameter, most often arbitrarily set at ≤ 2 mm. However, root classification based on either branching order or on function (transport vs absorptive roots) may be more meaningful for understanding organic matter dynamics than classifications based on size class (Pregitzer *et al*., 2002; McCormack *et al*., 2015a). Most distal root orders generally have shorter lifespans and therefore contribute disproportionately to root turnover and production of root necromass (Chen & Brassard, 2013). Furthermore, regardless of their diameter, different root branching orders differ significantly in their tissue chemistry (Pregitzer *et al*., 2002; Fan & Guo, 2010; Goebel *et al*., 2011; Xiong *et al*., 2013; Ma *et al*., 2016), which is highly relevant for understanding fine‐root decomposition and its contribution to soil C and nutrient dynamics. Absorptive roots (often the lowest three root orders) also differ consistently from transport roots in characteristics relevant to decomposition, such as lignin : N ratio, condensed tannin concentration, and mycorrhizal colonisation (McCormack *et al*., 2015a). Therefore, quantifying the impact of root decomposition at the ecosystem scale requires understanding root order‐specific biomass, turnover, and decomposition rates and their relationships with traits.

### 1. Root litter mass loss rate and dynamics

*Root litter mass loss rate* is the dry mass lost by roots per initial dry mass of roots per unit time during some incubation period (typical units: g g^−1^ yr^−1^).

Most studies of root decomposition use the litter bag technique, which has several advantages: (1) litter bag construction does not require collection or study of roots in monospecific stands to compare among species and therefore allows for more flexibility in designing experiments for species comparisons; and (2) litter bags facilitate studies of decomposition rates (and their relationships to traits) by root order or diameter class. However, it is worth noting that the litter bag technique, while relatively convenient and inexpensive, may misrepresent *in situ* fine‐root decay rates by altering the preexisting relationships among roots, soil, and the rhizosphere decomposer community as well as aspects of the environment (Fahey & Arthur, 1994; Dornbush *et al*., 2002; Li *et al*., 2015). For example, root litter bags are often constructed of fine mesh that, while preventing growth of roots into the bag and loss of roots from the bag, also prevents most soil fauna from contributing to root decomposition. In addition, litter bags are likely to minimise the influence of rhizosphere processes such as exudation over decomposition of newly dead roots. Finally, the decomposition of dead roots that remain incorporated in a living mycorrhizal mycelium may differ from roots that have been removed, dried and reintroduced to soil in a litter bag, as some mycorrhizal taxa (e.g. ericoid mycorrhizal taxa) may switch into saprotrophic mode after the root dies (Kohout *et al*., 2018; Martino *et al*., 2018).

Several alternative methods can be used to estimate root decomposition rates, including trenched plots, buried pots, tethered roots and the intact‐core method. In the trenched plot method, a trench is dug around an area of ground that is weeded of live plants, to isolate it from new root inputs and to kill existing roots, whose *in situ* decay is subsequently followed through sequential coring and root biomass estimation (Silver & Vogt, 1993). The buried pot method encloses roots and soil in an inverted buried pot, minimising root in‐growth, with coarse mesh covering the pot opening to allow access to invertebrates and other decomposers, so that root mass loss can be determined on enclosed roots (Gijsman *et al*., 1997). The tethered root approach has been used to follow decomposition of fine and coarse woody roots by measuring mass loss rates of the tethered root samples in the field (Fahey *et al*., 1988; Fahey & Arthur, 1994). The intact‐core technique involves collecting cores near individual plants that have been killed recently (e.g. because of above‐ground harvest or girdling) to isolate roots of a single species, reinstalling cores (fit with mesh caps and bottoms) in a site of interest, and harvesting cores at intervals to follow the change in the mass of roots of different size classes. This method requires no *a priori* root processing, maintains *in situ* decomposition conditions, and retains some natural rhizosphere associations, although the influence of live plants on rhizosphere processes ceases (Dornbush *et al*., 2002) and root death may have bypassed natural senescence (including nutrient and C resorption processes). Unlike litter bags, the intact‐core method does not measure mass of dead roots in cores before their deployment, but instead compares biomass of roots in cores after they are harvested to root mass in cores collected at the time of core construction and deployment. Yet another approach combines minirhizotrons with sequential coring to measure root turnover, death rate, mass of live roots, and mass of dead roots, and uses a mass‐balance approach to estimate decomposition rate of roots *in situ* (Li *et al*., 2020). This method has the advantage of estimating decomposition of dead roots that have intact associations with soil and live roots. It is most accurate in monospecific stands because of the large amount of effort needed to obtain the necessary species‐specific parameters in mixed stands (Li *et al*., 2020). The very limited comparisons among different methods suggest that litter bags may lead to estimates of slower root decomposition rates than measurements that leave roots more intact (Dornbush *et al*., 2002; Sun *et al*., 2013a). However, few studies have directly compared different methods of determining root decomposition rates (Fahey *et al*., 1988; Fahey & Arthur, 1994; Dornbush *et al*., 2002).

For root litter bags and tethered roots, the majority of effort is required up front during the collection of roots. Both the trenching and intact‐core methods require a substantial time investment for sample processing upon collection of cores (Dornbush *et al*., 2002; Sun *et al*., 2013a). The buried pot method requires time investment for root collection as well as for isolating roots from collected pots. The combined minirhizotron‐sequential core method requires intensive image and root core processing. Readers should consider using alternative methods such as trenching, intact cores, and minirhizotron‐sequential coring methods when research questions are focused on obtaining realistic rates of *in situ* decomposition for developing ecosystem budgets or for parameterising or validating models. Conversely, if questions relate more to understanding the influence of traits and soil characteristics on decomposition rates or differences among species, litter bags might be more appropriate. We encourage comparisons of litter bags and methods that are less disruptive of *in situ* conditions to develop quantitative estimates of relationships among methods to facilitate the integration of the relatively abundant litter bag data into models. Below, we provide detailed recommendations regarding root sampling, storage, and processing and litter bag construction for the litter bag technique because its use is so common, and refer readers to other papers for details regarding methods for measuring decomposition of intact roots (e.g. Dornbush *et al*., 2002; Li *et al*., 2020).

#### a. Sampling recommendations

Researchers using root litter bags can control whether they study live or senesced roots, although standardising the degree of root death is difficult (Freschet *et al*., 2010), as root death is less discrete than leaf senescence. Most litter bag studies use freshly killed live roots as substrates. However, the physiological and chemical characteristics of live roots differ from those that experience a prolonged senescence period during which they gradually lose function (Eissenstat & Volder, 2005). Although retranslocation was long thought to be negligible in roots, evidence is growing that roots retranslocate significant amount of nutrients upon senescence, and that root retranslocation efficiency rates vary considerably among species, but on average are less than for leaves (Freschet *et al*., 2010; Brant & Chen, 2015). Accordingly, some studies have used newly dead roots to allow any retranslocation of nutrients to occur before study (e.g. Freschet *et al*., 2010). In studies using trenching and intact cores, the researcher has less control over the substrates that are decomposing, which are likely to include some combination of freshly killed, senescing and dead roots. Whether roots are infected with mycorrhizas also has the potential to influence the rates of root decomposition (Langley *et al*., 2006; Koide *et al*., 2011); for example, ericoid mycorrhizas are able to switch to saprotrophic capabilities upon root death (Martino *et al*., 2018). How mycorrhizas may influence retranslocation is uncertain.

If comparisons among species are desired, roots must be identified to the species level, either by collecting roots from monospecific stands or pots, or by tracing a root to its parent plant. For the latter, one method is to establish plots at a relatively close distance to an individual stem and excavate the surface soil around the plant to expose lateral roots. Roots can be carefully excavated from soil and traced back to the stem for species identification.

#### b. Storage and processing

Once collected, handling and storage of sampled roots will depend on the nature of the study to be conducted. For most purposes, roots should be refrigerated if they can be processed within a few days or frozen for later processing if sorting by root order is desired. For studies of decomposition by root order, roots of each species should be dissected into different branch orders with the most distal root tips labelled as first order (following Pregitzer *et al*., 2002), which cannot be done accurately on air‐dried roots. Studies of root size classes will require sorting roots into appropriate classes. Once root orders or size classes are isolated, roots should be air‐dried or oven‐dried at moderate temperature (e.g. 50°C) to avoid altering root chemistry. Ratios of oven‐dry (65°C) to air‐ or 50°C‐dry material should be developed using subsets of material, to allow comparison with harvested mass.

#### c. Measurement procedure

##### Litter bag construction

Mesh size should be selected to maximise colonisation by soil microbes and fauna, and contact with surrounding soil, while preventing loss of roots to soil or in‐growth of new roots into bags. Diameter of first‐order roots can vary considerably. For instance, in one study of subtropical woody species, first‐order root diameter ranged 73–1010 µm among species (Kong *et al*., 2014), so most litter bag studies of root decomposition will require a mesh size of *c*. ≤ 50 µm. Because fungal hyphal diameters are on the order of 5 µm (Iotti *et al*., 2002; Ordaz *et al*., 2012), whereas soil microfauna range in size up to 100 µm, a mesh that will prevent root loss and in‐growth will exclude mesofauna and macrofauna (100–2000 µm and > 2000 µm, respectively), but allow access to microbes, and some, but not all, microfauna. A potential alternative is the use of litterbags with distinct faces, the bottom face (preventing root loss) using finer mesh size than the upper one (allowing fauna movement), although this is best suited for common‐garden studies where all vegetation has been removed as the use of a coarser mesh on the upper surface may allow root in‐growth into the bags, and is not appropriate if bags are buried (and root litter bags often are). Bag dimensions should be chosen to allow roots to be packed at densities that are similar to those occurring naturally in the soil, as overly dense packing of roots may impede microbial access to roots and alter the microenvironment.

##### Litter bag set‐up, harvesting and processing

Litter bags containing air‐dried root material should be deployed in the soil where roots would normally decompose, at depths appropriate to the research questions being addressed. For instance, for general estimations of ecosystem organic matter decay rates, deployment depths should match depths where the majority of root turnover and microbial activity occurs, often the surface horizon. Litter bags can be inserted vertically in the soil or at a 45° angle to maximise contact with soil and flowing water. Harvest intervals and study duration will depend upon the expected mass loss, which in turn will depend on factors such as climate (especially seasonality), the soil environment and the root substrates themselves. For instance, harvest intervals will need to be more frequent in warm, mesic ecosystems where mass loss occurs more rapidly. Upon harvest, decomposed root litter should be removed from the litter bags, dried (65°C), and weighed. A root cleaning step may be necessary depending on the mesh size. If litterbag mesh size is ≤ 50 µm, little contamination from surrounding organic matter may be expected. However, as the mesh size increases, there will be increasing contamination by soil particles. In such instances, sorting out roots from SOM and brushing off soil from the root (using hands or a light brush) would be recommended. The use of water can lead to underestimation of the mass of root remaining, while not using water will have the opposite effect, so the degree to which roots should be cleaned should be considered carefully and be consistent among samples. Subsequent ashing may also be necessary to account for contamination by mineral soil particles or carbonate precipitation. In noncalcareous soils, determination of C concentration and expression of decomposition on a C remaining basis can account for contamination.

##### Calculations

If multiple harvests are possible, mass (or C) remaining can be fit against time to various decay models (Wieder & Lang, 1982; Cornwell & Weedon, 2014), including models that assume discrete pools that decay at different exponential rates vs reactivity continuum models that use a probability distribution to describe a continuum of decomposition rates (Koehler & Tranvik, 2015). The choice of decomposition index or analytical method of gross decomposition measurements can influence the inference of relative species decomposability. Expressing results in terms of percentage mass loss restricts interpretation to the time‐scale of the incubation. The alternative, expressing decomposability in terms of rate parameters of decay models requires explicit commitment to a certain model of decomposition dynamics, justified either by previous assumptions, or by collecting multiple observations and statistically comparing different decay models. Providing further details regarding these alternatives is beyond the scope of this handbook, however one common approach is to select (using an information‐theoretic approach, Burnham & Anderson, 2002) among discrete‐pool single exponential (*X* = *e* ^–^ *^k^S^t^*), double exponential (*X* = *Ce* ^–^ *^k^_1t_* + (1 − *C*)*e^–k^_2t_*), and asymptotic (*X* = *A* + *(*1 − *A)e^–k^_A_^t^*) decomposition models, where *X* is the proportion of initial mass remaining at time *t* and *k_S_* is the decomposition constant in the single exponential model; *C* is the fraction of the initial mass that decomposes with decomposition rate *k_1_*, while the remaining fraction (1 − *C*) decomposes with rate *k_2_* in the double exponential model; and *A* is the fraction of the initial mass with a decomposition rate of zero (i.e. the asymptote), while the remaining fraction (1 − *A*) decomposes with rate *k_A_* in the asymptotic model.

#### d. Future research directions

To better link root turnover, litter decomposition rate and soil C and nutrient cycling, decomposition studies based on roots with well defined lifespan (e.g. sorted by root order or between absorptive vs transport roots) are needed. Also, new methodology to accurately measure decomposition rates of naturally dead roots is required as most studies currently use fresh roots owing the many obstacles in collecting nondecomposed, naturally senesced roots. Methods that estimate root decomposition *in situ* (i.e. without removing roots from the soil biotic and abiotic context) are promising (Li *et al*., 2020).

Given that the small mesh size of litter bags used for measuring fine‐root decomposition excludes all mesofauna and macrofauna, which are important processors of detritus in terrestrial ecosystems (Hättenschwiler & Gasser, 2005; Hobbie *et al*., 2006; Handa *et al*., 2014), further studies are needed to determine their role in the decomposition of roots differing in decomposability (Fujii *et al*., 2018) and to test whether root litter bag studies systematically underestimate root decomposition rates. Similarly, although mycorrhizal fungi are known to influence plant litter decomposition, there is still considerable uncertainty regarding how mycorrhizal colonisation affects fine‐root decomposition (Langley *et al*., 2006; Koide *et al*., 2011; Beidler & Pritchard, 2017). Future studies that directly compare the decomposition of roots and mycorrhizal fungi and other methods to study the influence of mycorrhizal fungi on decomposition are a high priority, given their importance for C sequestration and nutrient cycling and their roles as substrates for decomposition that are physically connected to roots, as agents of decomposition, and through their interactions with saprotrophs (Lindahl & Tunlid, 2015; Martino *et al*., 2018; Frey, 2019).

Most studies to date have focused on the early and middle stages of litter decomposition to describe litter decomposition rate and dynamics. However, while initial litter decomposition rate provides reasonably good estimates for the whole decomposition process of many leaf litters, this may not be true for slow‐decomposing litters (e.g. Freschet *et al*., 2012b; Kyaschenko *et al*., 2019), such as root litter. Furthermore, the drivers of root decomposition in its early stages can be different from those in the later stages of decomposition. Therefore, a stronger focus on the later phases of the decomposition process is needed to understand the links between litter chemistry, environmental conditions and ecosystem functions, such as nutrient cycling and SOM formation.

Finally, many studies indicate that root decomposition proceeds considerably slower than leaf‐litter decomposition (Gholz *et al*., 2000; Freschet *et al*., 2013; Sun *et al*., 2018). Meta‐analyses of decomposition studies using bulk fine roots indicate that root and leaf litter decomposition are correlated across species, although more so across than within ecosystems (Freschet *et al*., 2013). Nevertheless, comparative studies of above and below‐ground decomposition are relatively rare and should be a focus of future research.

### 2. Root litter nutrient release

*Root litter nutrient release rate* is the mass of nutrients lost (or gained) by roots per initial mass of nutrients per unit time during some incubation period (typical units: yr^−1^).

*Maximum nutrient immobilisation* is the difference between the litter nutrient mass when reaching its peak during decomposition and the initial litter nutrient mass, per initial litter mass (typical units: g N g^−1^ litter).

As roots decompose, they lose not only C (to CO_2_ through microbial respiration) but also nutrients. If nutrients limit C use by decomposers because they are present in low concentrations in litter, decomposers may take up nutrients from the environment (soil, throughfall, or N‐fixation for N) and incorporate them into their biomass. Because litter and decomposers growing on it are generally measured together, such litter exhibits a period of immobilisation – an absolute increase in the amount of nutrient in litter – that lasts until the C : nutrient ratio narrows to the point that C becomes limiting, and decomposers release nutrients from litter (Manzoni *et al*., 2008). Therefore, as with above‐ground litter, the dynamics of nutrient release from decomposing roots depend on whether those nutrients are limiting to decomposers in fresh litter and are present in the roots as organic molecules (e.g. N, P and S) that require decomposition to be released or as ions (K, Mg, etc.) whose release depends on their potential to be exchanged at cation‐exchange sites and leached (Staaf & Berg, 1981).

Although it has been suggested that roots immobilise relatively little N (Parton *et al*., 2007; Kyaschenko *et al*., 2019), the dynamics of nutrient release from decomposing root litter may, similarly to leaf litter, be related to the initial concentration of those nutrients. For example, in a comparison among 14 tree species, roots with lower initial N concentrations immobilised more N than those with higher root N concentrations, whereas roots with the highest initial N concentrations began releasing N immediately upon decomposing (Hobbie *et al*., 2010). However, this relationship might be more complex depending on the type of N compounds contained in litter and the root order considered (Xiong *et al*., 2013). For instance, in a study using a branch‐order classification (T. Sun *et al*., unpublished) root N loss increased significantly with increasing order among the first five branch orders across 40 co‐occurring temperate woody species over 4 yr exposure in field, even though lower order roots had higher nutrient concentrations than higher‐order roots. Root C loss, however, explained 71% of the variation of N loss across all species. The slower rates of N release in lower order roots, despite higher initial N concentrations, probably occurred because their root N is mainly in recalcitrant forms and release of most N occurs only when the recalcitrant C fractions are utilised by decomposer communities. Finally, while root litter nutrient release can potentially increase the availability of this nutrient for soil microorganisms and plant roots, the actual fate of released nutrients will vary dramatically among different types of soils (Schimel & Bennett, 2004; Hobbie, 2015).

#### a. Sampling recommendations

See section **XXIII. 1. Root litter mass loss rate and dynamics**. Note that advances in identifying and collecting naturally senesced litter are desired for characterising nutrient dynamics during root decay, as any retranslocation could alter those dynamics.

#### b. Storage and processing

See section **XXIII. 1. Root litter mass loss rate and dynamics**.

#### c. Measurement procedure

##### Litter bag construction, set‐up, harvesting and processing

See section **XXIII. 1. Root litter mass loss rate and dynamics**. To determine root litter nutrient release, initial nutrient concentrations will need to be determined on subsamples of initial roots. Upon harvest, roots should be dried at temperatures ≤ 65°C to avoid volatilising N. Details regarding analyses of nutrients in roots are described under section **XIV. Root chemistry**.

##### Calculations

The proportion of the initial nutrients remaining at any time point, *t*, is equal to *N_t_*/*N_0_*, where *N_0_* is the initial nutrient pool (determined by multiplying the initial litter mass by the initial mass‐based nutrient concentration) and *N_t_* is the nutrient pool at time *t* (determined by multiplying the litter mass at time *t* by its mass‐based nutrient concentration). The maximum immobilisation can be calculated at (*N_t_* − *N_0_*)/*M_0_*, where *t* is the time at which *N* is at its maximum and *M_0_* is the initial litter mass. It is often informative to plot *N_t_*/*N_0_* against *M_t_*/*M_0_*, where *M_t_* is mass remaining at time *t*, to understand whether differences among root substrates (e.g. species, root order) in the dynamics and rate of nutrient release relate to aspects other than differences in rates of mass loss, such as aspects of substrate chemistry, or environmental factors unrelated to mass loss (e.g. Parton *et al*., 2007).

#### d. Future research directions

The range of research directions identified for litter decomposition rate generally applies to root litter nutrient release and immobilisation. In addition, the interplay between root nutrient release and mass loss are still poorly understood, as well as their respective effects on soil functioning.

## Author contributions

GTF initiated and coordinated the handbook project. GTF wrote section I. EG and GTF wrote section II with input from CR. GTF wrote section III. LP, MLM, GTF and BR wrote section IV. EG and CMI wrote section V. CR, HP, CMI, JAP, MLM and TSA wrote section VI, with input from GTF. CR, CMI, MLM and TSA wrote section VII, with input from a range of authors. JK and JHCC wrote section VIII. CMI, HP, VS and JK wrote section IX, with input from GTF. LP and JAP wrote section X, with input from GTF. LM, AW and LHC wrote section XI. PR and OJV‐B wrote section XII, with input from GTF, LHC and LR. MZ, LHC and ABZ wrote section XIII, with input from MW and a range of authors. IB and NT wrote section XIV, with input from HP, MGdM and SJ. AS wrote section XV. MLM and CPC wrote section XVI. ICM and BR wrote section XVII. AG and MS‐L wrote section XVIII. NAS wrote section XIX. NW wrote section XX, with input from SAB. LP and AGB wrote section XXI. LMY and EBB wrote section XXII. TS and SEH wrote section XXIII, with input from GTF. GTF reviewed and edited all sections, with help from HL, MW and LR, and a range of authors, including AGB, BR, CR, EG, HP, IB, ICM, JHCC, LHC, LMY and OJV‐B.
